# Omaveloxolone functions as a molecular crowbar-like degrader of STING to alleviate aging-related inflammation

**DOI:** 10.1038/s41467-026-77760-1

**Published:** 2026-09-17

**Authors:** Jingyi Zhou, Xinyi Ma, Bing Jiang, Zisheng Fan, Penghua Li, Qi Dai, Yajie Wang, Min Wu, Qibang Sui, Ning Ma, Ruirui Yang, Haiwei Shen, Jingyi Meng, Manlin Huang, Zhiming Ge, Jiusheng Lin, Dan Teng, Mingyue Zheng, Sulin Zhang

**Affiliations:** 1 School of Pharmaceutical Science and Technology, Hangzhou Institute for Advanced Study, University of Chinese Academy of Sciences, Hangzhou, China; 2 Drug Discovery and Design Center, State Key Laboratory of Drug Research, Shanghai Institute of Materia Medica, Chinese Academy of Sciences, Shanghai, China; 3 School of Chinese Materia Medica, Nanjing University of Chinese Medicine, Nanjing, China; 4 Department of Translational Medicine Platform, The Affiliated Hospital of Hangzhou Normal University, Hangzhou, China; 5 University of Chinese Academy of Sciences, Beijing, China; 6 School of Pharmacy, Nanchang University, Nanchang, China; 7 Key Laboratory of Marine Drugs, Chinese Ministry of Education, School of Medicine and Pharmacy, Ocean University of China, Qingdao, China

**Keywords:** Pharmacology, Proteolysis, Ageing, Inflammatory diseases, Pattern recognition receptors

## Abstract

Aberrant activation of the STING signaling pathway is involved with aging-related inflammation, yet no STING-targeting therapeutics are clinically approved. Here, we identify Omaveloxolone, an FDA-approved drug for Friedreich’s ataxia, as a potent STING degrader (DC_50_ = 118 nM, D_max_ = 98.6%) via high-throughput screening. Omaveloxolone binds non-covalently to the base of the STING ligand-binding domain (LBD) with high affinity (*K*_D_ = 21.4 nM) and exhibits crowbar-like behavior consistent with STING conformational destabilization: it hinders LBD closure, increases hydrophobic exposure, and reduces thermal stability. This conformational perturbation is proposed to enhance recognition by the E3 ubiquitin ligase HUWE1, leading to K27-linked ubiquitination at STING lysines K236/K347/K370 and proteasomal degradation. In vivo, Omaveloxolone depletes STING, suppresses downstream inflammatory signaling, and ameliorates aging-related inflammatory phenotypes in aged male mice. Because Omaveloxolone is also an established NRF2 activator, these in vivo benefits likely reflect dual modulation of inflammatory and antioxidant pathways. This study implicates Omaveloxolone as a molecular crowbar-like STING degrader and provides a mechanistic framework for targeting STING via conformational perturbation.

## Abstract (web summary)

Aging-related inflammation can have excessive activation of the STING pathway as some of its features. Here the authors look at the function of Omaveloxolone and show this compound binds to the base of the STING ligand-binding domain and destabilises STING leading to STING K27-linked ubiquitination and proteasomal degradation and show Omaveloxolone reducing aging associated inflammation in aged male mice.

## Introduction

The stimulator of interferon genes (STING) pathway functions as a master regulator of innate immunity, which is activated upon binding of STING to the second messenger 2’,3’-cGAMP—generated by cGAS following detection of cytosolic DNA^1–5^. Upon cGAMP engagement, STING translocates from the endoplasmic reticulum to the Golgi apparatus, triggering activation of TBK1-IRF3 and NF-κB signaling cascades, culminating in the induction of type I interferons (IFNs) and proinflammatory cytokines^6–9^. Aberrant STING activation has been linked to a spectrum of inflammatory disorders^3^. For instance, loss-of-function mutations in the DNase *TREX1* result in cytosolic DNA accumulation and pathological STING signaling, underpinning the pathogenesis of autoinflammatory diseases such as Aicardi-Goutières syndrome (AGS), systemic lupus erythematosus (SLE), and familial chilblain lupus (FCL)^10–13^. Additionally, gain-of-function (GOF) mutations in STING (e.g., V155M, N154S) induce ligand-independent constitutive activation, leading to STING-associated vasculopathy with onset in infancy (SAVI)^14–17^.

Emerging evidence establishes STING as a key mediator of aging-associated inflammation. Cellular senescence, a hallmark of aging^18,19^, generates micronuclei and chromosomal fragments that activate the cGAS-STING-TBK1-IRF3 axis, thereby promoting the production senescence-associated secretory phenotype (SASP) factors (e.g., CXCL10, IL-6)^20–22^. Furthermore, STING coordinates responses to oxidative stress, radiation, and inflammatory stimuli to drive senescence^21,23^. Additionally, the activation of the non-canonical STING-ERK-eIF2α pathway has also been shown to enhance SASP expression^24^. Within the central nervous system, microglial STING-dependent IFN responses have been implicated in neuronal loss and neurodegeneration^25–27^. Thus, STING inhibition holds therapeutic potential for addressing chronic inflammation and aging-related pathologies.

To date, several STING inhibitors have been described. Compounds such as ASTIN C, SN-011, and Ginkgetin^28–32^ function through direct binding to the STING ligand binding pocket, whereas others including C-176 and H-151^33–35^ suppress STING activation via inhibition of palmitoylation or oligomerization. Notably, Ginkgetin—a biflavonoid component of Ginkgo biloba extract—has been shown in our studies to downregulate senescence marker expression and ameliorate inflammatory and pathological phenotypes in aging mouse models^30^. H-151, a prototypical STING inhibitor, abrogates STING activation through covalent modification of the critical cysteine residue Cys91, exhibiting potent inhibitory activity^33^. It has been widely utilized in studies investigating aging-related inflammation and neurodegenerative diseases^26,27,36,37^. However, no STING inhibitors have yet been approved for clinical use, and none have advanced to clinical trials.

In recent years, targeted protein degradation (TPD) has emerged as a transformative therapeutic strategy, with several agents advancing to clinical trials or receiving regulatory approval^38–40^. The field is nominated by two main modalities: PROTACs and molecular glues^41–43^. PROTACs function as bifunctional scaffolds that co-recruit E3 ubiquitin ligases and target proteins, facilitating ternary complex formation to trigger ubiquitination and proteasomal degradation of the target.

Several STING-targeting PROTAC degraders have been reported. In 2022, Liu et al. reported the first-in-class STING PROTAC, SP23 (DC_50_ = 3.2 μM, *D*_max_ = 86%), which exerted significant anti-inflammatory effects in a cisplatin-induced acute kidney injury mouse model^44^. In 2023, Zhu et al. developed UNC9036 (DC_50_ = 227 nM, *D*_max_ = 91.7%), a STING PROTAC constructed from the STING agonist diABZI and the VHL ligand VH032^45^. That same year, Luo et al. designed SD02 (DC_50_ = 530 nM, *D*_max_ = 56.7%), a covalent STING PROTAC that selectively engages Tyr residues of STING via an N-sulfonyl pyridone warhead^46^. However, UNC9036 and SD02 exhibit molecular weights exceeding 1400 Da, a property that may compromise their drug-like profiles. Subsequently, Nakamura et al. developed a low-molecular-weight, dual-covalent STING PROTAC (compound 2) based on the STING inhibitor C-170 and a minimal covalent moiety of the E3 ligase RNF126, achieving 75% STING degradation at 3 µM with sustained activity^47^.

Molecular glues, in contrast, promote protein degradation by allosterically remodeling interaction interfaces between E3 ligases and their substrates^48^. In 2024, Novartis BioMedical Research reported AK59, the first STING molecular glue degrader, which recruits the E3 ligase HERC4 to form a HERC4-AK59-STING ternary complex and mediates STING degradation with micromolar potency^49^.

More recently, a “molecular crowbar” mechanism has been described in TPD, wherein small molecules directly bind and destabilize target proteins, rendering them susceptible to E3 ligase recognition and proteasomal degradation—an innovative strategy distinct from established modalities^50^. To date, however, only one molecular crowbar degrader has been reported, which targets Pin1 through covalent binding, leaving this mechanism largely unexplored for other proteins.

Collectively, these advances highlight the feasibility of STING degradation but also underscore critical limitations of existing agents—including excessive molecular weight, suboptimal drug-like properties, and insufficient degradation efficacy—that hinder clinical translation. This underscores the pressing need for STING degraders with enhanced potency and improved drug-like profiles.

The Oleanolic acid derivative RTA 408 (Omaveloxolone) is an FDA-approved therapeutic for Friedreich’s ataxia (FA) in adolescents and adults. FA, an autosomal recessive neurodegenerative disorder, stems from GAA trinucleotide repeat expansions in the *FXN* gene encoding frataxin^51,52^. Clinically, it typically presents in childhood or adolescence, characterized by cerebellar ataxia, impaired motor coordination, and cardiomyopathy. Initially developed as an NRF2 agonist with confirmed blood-brain barrier permeability^53–56^, the precise therapeutic mechanism of Omaveloxolone in FA remains incompletely characterized.

Beyond its approved FA indication, Omaveloxolone is under evaluation in multiple clinical trials, including melanoma (NCT02259231), mitochondrial myopathy (NCT02255422), and post-ophthalmic surgery inflammation (NCT02065375). Importantly, no clinical or preclinical studies have thus far explored its therapeutic potential in aging-related inflammation.

In this study, we show that Omaveloxolone acts as a STING-targeting molecular crowbar-like degrader. It binds with high affinity (*K*_D_ = 21.4 nM) to the base of STING ligand-binding pocket via non-covalent interactions. This binding event destabilizes STING conformation, enhancing its recognition by the E3 ubiquitin ligase HUWE1 and ultimately promoting STING ubiquitination and degradation. In vivo, Omaveloxolone effectively depletes STING protein and ameliorates aging-related inflammatory phenotypes. These findings not only establish that Omaveloxolone acts as a molecular crowbar-like degrader, but also extend its potential therapeutic utility to aging-associated inflammatory diseases.

## Results

### Discovery of potential STING degraders via flow cytometry-based screening

**Fig. 1:**
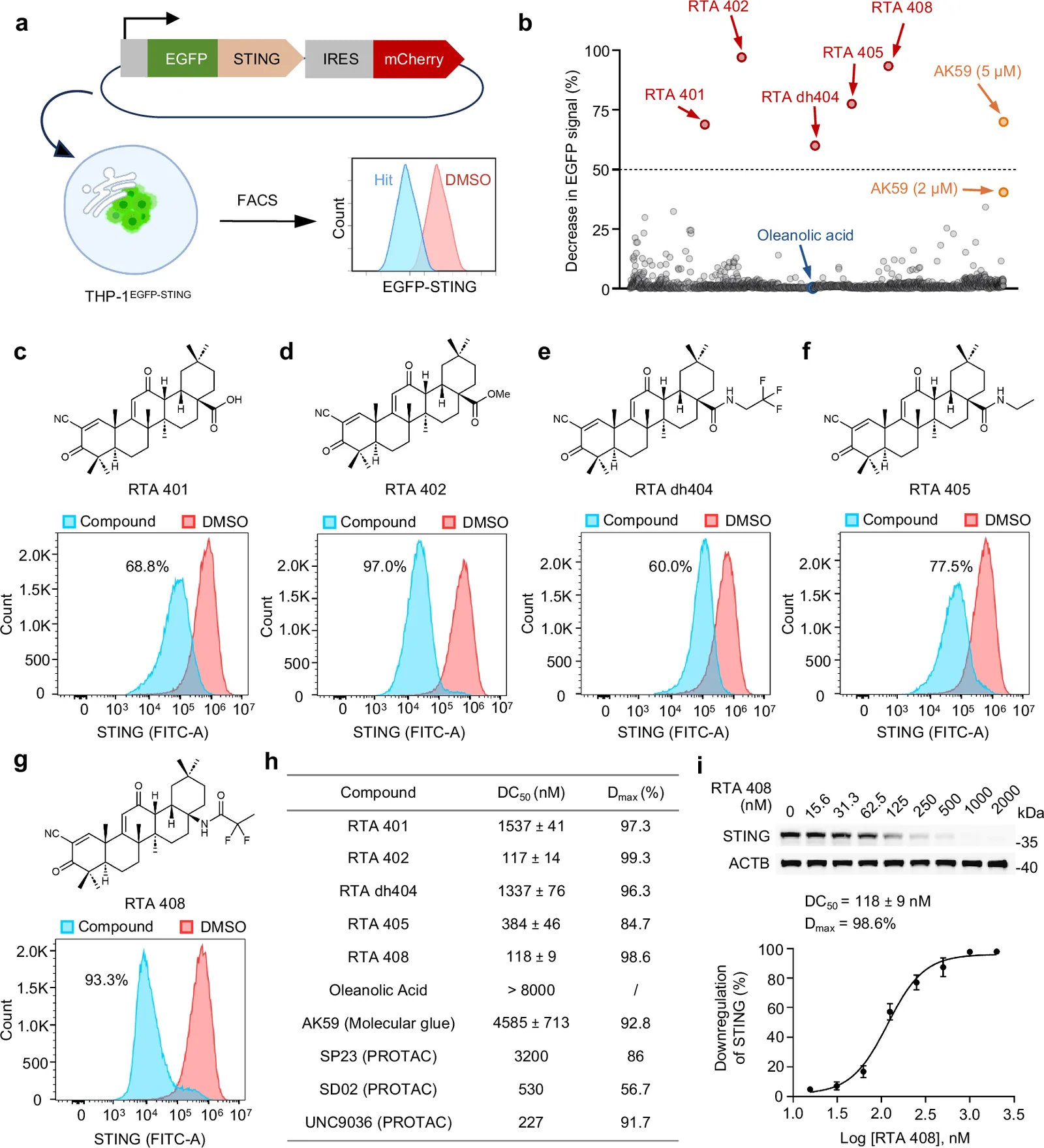
Discovery of potential STING degraders via flow cytometry-based screening. **a**, Schematic workflow of the flow cytometry-based screening strategy. The schematic was assembled by the authors using elements created in BioRender; Zhou, J. (2026) https://BioRender.com/29gmzuu. **b**, The effects of approximately 3,700 natural products and their derivatives on STING downregulation in THP-1^EGFP-STING^ cells at 2 µM (12 h treatment) were analyzed using the flow cytometry-based screening. AK59 was used as positive control. **c–g**, Chemical structures of RTA 401, RTA 402, RTA dh404, RTA 405, RTA 408, and their ability to downregulate STING at 2 µM (12 h treatment) in THP-1^EGFP-STING^ cells, as analyzed by the flow cytometry-based screening. Gating strategy for these panels is shown in Supplementary Fig. 10. **h**, Overview of DC_50_ and *D*_max_ values of indicated compounds. Data for the RTA series (401, 402, dh404, 405, and 408), Oleanolic acid, and AK59 were measured by western blot in THP-1 cells; data for SP23, SD02 (measured in THP-1 cells), and UNC9036 (measured in Caki-1 cells) were obtained from published research. **i**, Western blot analysis of STING protein levels in THP-1 cells treated with indicated concentrations of RTA 408 for 24 h. DC_50_ refers to half-maximal downregulation concentration; *D*_max_ refers to maximum downregulation efficacy. DC_50_ values were determined by fitting a dose-response curve (variable slope) generated from quantitative data through nonlinear regression analysis using Graphpad Prism 8.0.1 (*n* = 3 independent experiments, mean ± SD). Source data are provided as a Source Data file.

To identify compounds with STING degradation activity, we developed a flow cytometry screening system using a dual-reporter THP-1 cell line (THP-1^EGFP-STING^) stably expressing EGFP-STING-IRES-mCherry (Fig. 1a). This system enables real-time monitoring of intracellular STING protein levels via EGFP fluorescence, with mCherry signal serving as an internal control for high-throughput screening of candidate degraders. Natural products, owing to their structural diversity, bioactivities, and favorable biocompatibility, represent a valuable source for the discovery of targeted protein degraders^57,58^. Utilizing our flow cytometry-based screening system, we screened approximately 3700 compounds from our in-house library of natural products and their derivatives, with the reported STING molecular glue degrader AK59 used as a positive control. Western blot analysis confirmed STING downregulation effect of AK59 (DC_50_ = 4.59 μM, *D*_max_ = 92.8%; Supplementary Fig. 1a), consistent with its reported efficacy. Our flow cytometry screening reproduced this effect, showing dose-responsive STING reduction of AK59 (40.5% at 2 μM; 69.9% at 5 μM; Fig. 1b), thereby validating the reliability of our screening approach. To identify STING degraders outperforming AK59 while minimizing potential false negatives due to the hook effect at higher concentrations, we selected 2 μM as the optimal screening concentration.

This screen identified several Oleanolic acid derivatives—including RTA 401, 402, dh404, 405, and 408—that markedly reduced STING protein levels (60%–97% reduction) at 2 μM, surpassing the performance of AK59 (Fig. 1b–g and Supplementary Fig. 1b). However, Oleanolic acid itself showed no STING-downregulating activity (Fig. 1b, Supplementary Fig. 1c, d). Western blot analysis further confirmed the concentration-dependent STING downregulation induced by these derivatives (Fig. 1h, i and Supplementary Fig. 1e–h). Among them, RTA 402 (DC_50_ = 117 nM, *D*_max_ = 99.3%) and RTA 408 (DC_50_ = 118 nM, *D*_max_ = 98.6%) emerged as top candidates, substantially outperforming reported degraders including molecular glue AK59, PROTACs SP23, and SD02 (Fig. 1h). Notably, RTA 408, also known as Omaveloxolone, is an FDA-approved drug with a well-characterized safety profile, demonstrating obviously lower cytotoxicity than RTA 402 (Supplementary Fig. 1i). Given its high potency, reduced cytotoxicity, and favorable clinical characteristics, Omaveloxolone was selected for further investigation.

### Omaveloxolone mediates STING degradation via the ubiquitin-proteasome pathway

**Fig. 2:**
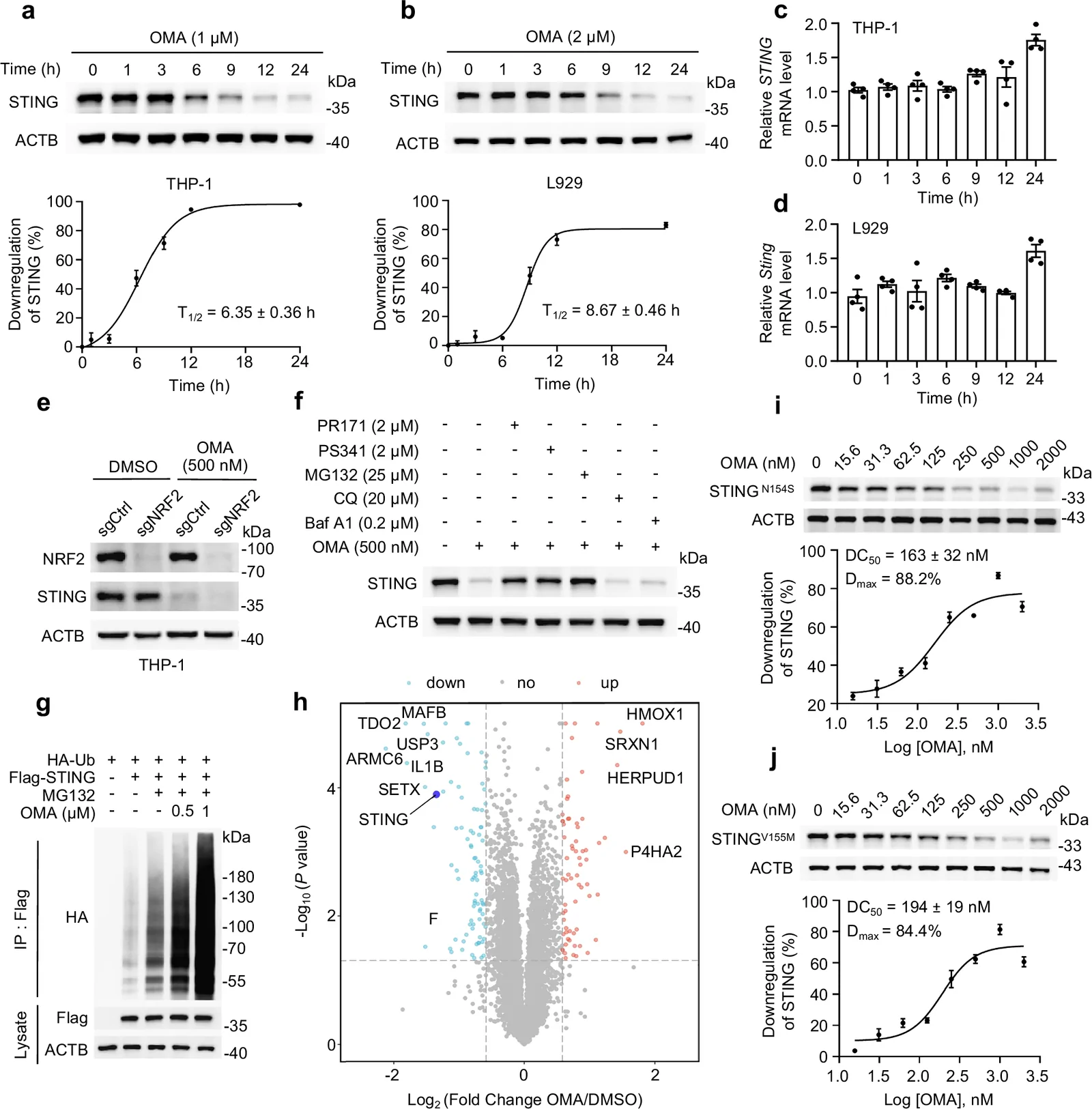
Omaveloxolone mediates STING degradation via the ubiquitin-proteasome pathway. Time-course analysis of STING protein degradation in human THP-1 cells (**a**) and murine L929 cells (**b**) following treatment with OMA at indicated concentrations. STING protein levels were assessed by western blot. RT-qPCR analysis of STING mRNA levels in THP-1 (**c**) and L929 (**d**) cells treated with OMA (1 μM for THP-1, 2 μM for L929) at indicated time points (*n* = 4, two technical replicates from two independent experiments, mean ± SEM). **e** Western blot analysis of STING and NRF2 protein levels in *NRF2*-knockout THP-1 cells treated with DMSO or OMA (500 nM) for 24 h. Data are representative of three independent experiments with similar results. **f** Effects of proteasome inhibitors or lysosomal inhibitors on OMA-induced STING downregulation. THP-1 cells were pretreated with proteasome inhibitors (PR171, PS341, MG-132) or lysosomal inhibitors (CQ, BafA1) for 1 h, followed by 6 h OMA (500 nM) treatment. STING protein levels were assessed by Western blot. Data are representative of three independent experiments with similar results. **g** Effects of OMA on STING ubiquitination. HEK293T cells were co-transfected with Flag-STING and HA-Ub plasmids. After pretreatment with MG-132 (5 μM) for 1 h, cells were treated with OMA (0.5 μM or 1 μM) for 24 h. Ubiquitinated Flag-STING was detected by immunoprecipitation followed by Western blot. Lysate samples were run on separate gels with ACTB as loading control. Data are representative of three independent experiments with similar results. **h** Proteomic analysis of THP-1 cells treated with 500 nM OMA for 12 h. The volcano plot displays differentially expressed proteins compared to the DMSO control. Statistical analysis was conducted using an unpaired two-sided Student’s *t*-test with raw unadjusted *P* values. Western blot analysis of OMA-induced degradation on STING^N154S^ (**i**) and STING^V155M^ (**j**). THP-1 cells expressing STING mutants N154S or V155M were treated with OMA at indicated concentrations for 24 h. OMA denotes Omaveloxolone; T_1/2_ refers to half-life of STING protein; DC_50_ refers to half-maximal downregulation concentration; *D*_max_ refers to maximum downregulation efficacy. T_1/2_ and DC_50_ values were determined by fitting a dose-response curve (variable slope) generated from quantitative data through nonlinear regression analysis using Graphpad Prism 8.0.1 (*n* = 3 independent experiments, mean ± SD). Source data are provided as a Source Data file.

To investigate the mechanism underlying Omaveloxolone-induced STING downregulation, we first characterized the temporal kinetics of STING protein reduction and its effects on *STING* mRNA levels during equivalent treatment periods. Our results demonstrated that Omaveloxolone treatment rapidly reduced STING protein levels (THP-1 T_1/2_ = 6.35 h, L929 T_1/2_ = 8.67 h), achieving maximal downregulation at 12 h that persisted for at least 24 h (Fig. 2a, b). RT-qPCR analysis revealed no reduction in *STING* mRNA levels throughout 24 h of Omaveloxolone treatment (Fig. 2c, d), suggesting that the mechanism of Omaveloxolone-induced STING protein downregulation is independent of transcriptional regulation.

Omaveloxolone is known as an NRF2 activator. Prior research reported that it can suppress *STING* transcription in murine bone marrow-derived macrophages (BMMs) through activation of NRF2^59^. Notably, this research demonstrated that Omaveloxolone suppresses *STING* transcription in BMMs after prolonged treatment (≥48 h), whereas it showed no reduction in *STING* mRNA levels within 24 h. This observation aligns with our findings in THP-1 cells, where Omaveloxolone treatment for 24 h similarly did not reduce *STING* mRNA expression (Fig. 2c, d).

To further investigate whether Omaveloxolone-induced STING protein downregulation is dependent on NRF2 in THP-1 cells, we generated an *NRF2*-knockout THP-1 cell line. The results showed that Omaveloxolone treatment for 12 h still significantly reduced STING protein levels in the absence of NRF2 (Fig. 2e), confirming that its mechanism of STING downregulation is independent of NRF2.

We next explored the underlying mechanism of Omaveloxolone’s STING-downregulating effect, with a particular focus on the ubiquitin-proteasomal and autophagy-lysosomal degradation pathways. In THP-1 cells, treatment of proteasome inhibitors (PR171, PS341, MG132), but not lysosomal inhibitors (Chloroquine (CQ), Bafilomycin A1 (Baf A1)), prevented Omaveloxolone-induced STING degradation (Fig. 2f), implicating the involvement of the ubiquitin-proteasome system. This observation was further confirmed in HEK293T cells co-expressing ubiquitin and STING, where Omaveloxolone promoted robust, dose-dependent STING ubiquitination (Fig. 2g). Proteomic analysis demonstrated that Omaveloxolone exerts a certain degree of selective degradation effect on STING (Fig. 2h and Supplementary Fig. 2a). Given that GOF STING mutations (e.g., N154S, V155M) drive spontaneous pathway activation and are linked to autoinflammatory disorders like SAVI and FCL, we established THP-1 cell lines stably expressing these mutants. Western blot analysis demonstrated that Omaveloxolone effectively degraded STING^N154S^ and STING^V155M^, with DC_50_ values of 163 nM and 194 nM, respectively (Fig. 2i, j), underscoring its therapeutic potential for treating diseases associated with GOF STING mutations.

In summary, Omaveloxolone degrades STING via the ubiquitin-proteasome pathway, achieving maximal efficacy by 12 h, with equivalent activity against pathogenic GOF STING mutants.

### Omaveloxolone binds non-covalently to the STING LBD with high affinity and reduces STING protein thermal stability

**Fig. 3:**
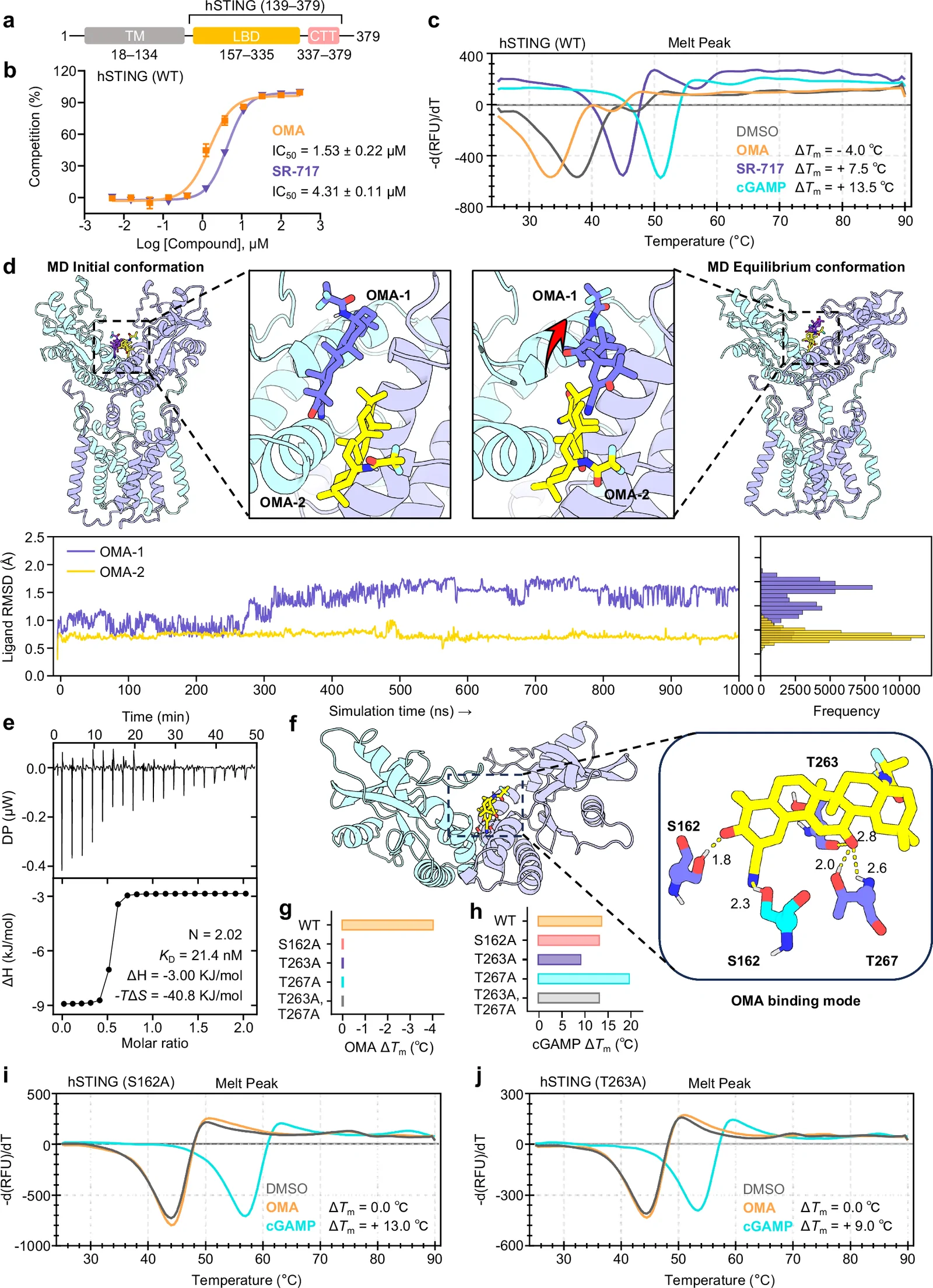
Omaveloxolone directly binds to STING LBD and reduces the thermal stability of STING protein. **a** Schematic representation of purified human STING (residues 139–379). **b** Competitive binding of OMA or SR-717 to hSTING^WT^ measured by FP assay. **c** Effects of OMA (1 μM), SR-717 (50 μM), and cGAMP (50 μM) on the melting temperature of hSTING^WT^ (2 μM) determined by PTS assay. **d** MD simulation analysis of OMA binding to the STING dimer. In the initial model, two OMA molecules (OMA-1 and OMA-2) are bound symmetrically (top left). During the simulation, one molecule (OMA-1) gradually dissociates from its original binding site and loses stable interactions with STING (top right), suggesting that OMA preferentially binds in a monovalent mode. **e** ITC measurement for the binding between OMA and hSTING^WT^. **f** Binding mode analysis of OMA with STING, highlighting key residues that form stable hydrogen bonds with the ligand. Overview of Δ*T*_m_ values of the hSTING^WT^ and its mutants (S162A, T263A, T267A, and T263A/T267A) induced by OMA (1 μM) (**g**) and cGAMP (50 μM) (**h**). Effects of OMA (1 μM) and cGAMP (50 μM) on the melting temperature of hSTING^S162A^ (2 μM) (**i**) and hSTING^T263A^ (2 μM) (**j**) determined by PTS assay. OMA denotes Omaveloxolone; IC_50_ refers to half-maximal inhibitory concentration; Δ*T*_m_ refers to the shift of protein melting temperature. IC_50_ values were determined by fitting the dose-response curves (variable slope) through nonlinear regression analysis using Graphpad Prism 8.0.1 (*n* = 4 independent biological replicates, mean ± SD). Source data are provided as a Source Data file.

To investigate how Omaveloxolone mediates STING degradation, we first examined whether it directly interacts with STING. Leveraging our experience in developing STING modulators, we used a fluorescence polarization (FP) assay with 5-FAM-labeled diABZI—a STING agonist—as a probe to evaluate Omaveloxolone’s ability to competitively bind the hSTING LBD^60–64^. Omaveloxolone competitively bound hSTING LBD with an IC_50_ of 1.53 μM (Fig. 3a, b). As a positive control, the STING agonist SR-717^65^ exhibited an IC_50_ of 4.31 μM, confirming that Omaveloxolone binds to hSTING LBD.

To confirm this interaction, we performed a protein thermal shift (PTS) assay, which demonstrated that Omaveloxolone markedly modulates the thermal stability of the hSTING (Fig. 3c), suggesting direct binding. Notably, unlike STING agonists SR-717 (Δ*T*ₘ = +7.5 °C) and cGAMP (Δ*T*ₘ = +13.5 °C), which increase STING thermal stability, Omaveloxolone significantly decreased it (Δ*T*ₘ = –4.0 °C) (Fig. 3c).

Given the presence of an α-cyanoacryloyl electrophilic warhead in Omaveloxolone—a moiety known to undergo Michael addition with cysteine residues—we therefore investigated whether it forms a covalent bond with STING. However, mass spectrometry analysis detected no covalent adducts (Supplementary Fig. 2b, c), indicating a non-covalent binding mode.

To characterize the binding mode, we performed extensive microsecond-scale molecular dynamics (MD) simulations. Since STING ligands such as SR-717^49^ and MSA-2^66^ often adopt a dual-ligand binding configuration, we first simulated a STING-Omaveloxolone complex with two ligand molecules. Only one molecule remained stably anchored at the bottom of the STING LBD; the second dissociated after ~300 ns (Fig. 3d), suggesting a preference for monovalent binding.

We validated this by isothermal titration calorimetry (ITC), in which STING protein was titrated into Omaveloxolone. This quantified a high-affinity interaction (dissociation constant, *K*_D_ = 21.4 nM) with a binding stoichiometry of 2:1 (two STING molecules per one Omaveloxolone molecule) (Fig. 3e)—indicating one Omaveloxolone molecule per STING dimer, consistent with MD simulations.

To refine the binding mode, we clustered equilibrated MD trajectories (last 200 ns) to identify representative poses. These revealed stable hydrogen bonds between Omaveloxolone and key residues at the STING LBD base: S162, T263, and T267 (Fig. 3f), implicating these as critical anchoring sites.

We validated this finding by mutating S162, T263, or T267 to alanine in hSTING LBD and assessing their thermal stability via PTS assay. These mutations abrogated Omaveloxolone-induced destabilization (Fig. 3g, i, j and Supplementary Fig. 2d, e) but did not affect cGAMP-mediated thermal stabilization (Fig. 3h–j and Supplementary Fig. 2d, e), consistent with published evidence that these residues are not critical for cGAMP binding^67^.

Further analysis highlighted that the carbonyl group on Omaveloxolone’s C-ring mediates hydrogen bonds with T263 and T267 (Fig. 3f). In contrast, Oleanolic acid—a structural analog lacking this carbonyl group—failed to compete with diABZI for STING LBD binding (Supplementary Fig. 2f, g), had no effect on STING thermal stability (Supplementary Fig. 2h), and could not stably anchor in the ligand-binding pocket in MD simulations (Supplementary Fig. 2i, j). These observations explained why Oleanolic acid lacks STING-degrading activity (Supplementary Fig. 1c, d).

Collectively, these data demonstrated that Omaveloxolone anchors to the base of the STING LBD pocket via specific hydrogen bonds with S162, T263, and T267, thereby destabilizing STING.

### Omaveloxolone destabilizes STING conformation via a molecular crowbar-like mechanism

**Fig. 4:**
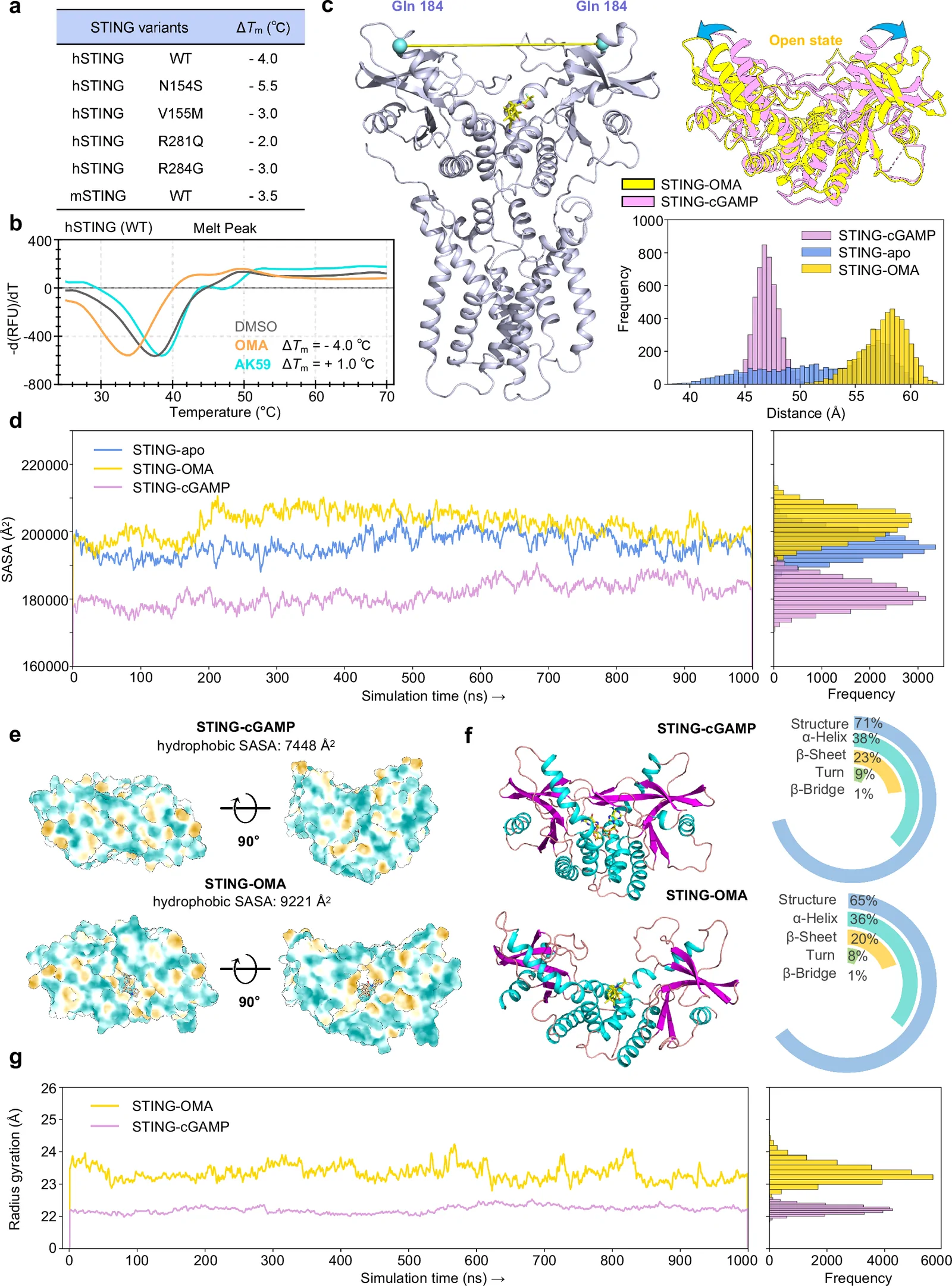
Omaveloxolone functions via a molecular crowbar-like mechanism to destabilize STING protein conformation. **a** Overview of Δ*T*_m_ values of the hSTING^WT^ (2 μM) and its disease-associated variants (N154S, V155M, R281Q, R284G) (2 μM), along with mSTING^WT^ (2 μM), induced by OMA (1 μM). **b** Effects of OMA (1 μM) and AK59 (1 μM) on the melting temperature of hSTING^WT^ (2 μM) determined by PTS assay. **c** Measurement of the Cα-Cα distance between Gln184 residues during MD simulations was used to evaluate the effect of different ligands on the open conformation of the STING LBD. Compared to the apo and cGAMP-bound systems, OMA binding more effectively stabilized the open state of the LBD. **d** Distribution of SASA across different systems during MD simulations. The STING-OMA complex exhibited a significantly higher SASA than control groups, indicating increased surface exposure upon OMA binding. **e** Hydrophobic surface analysis of representative stable conformations from STING-cGAMP and STING-OMA simulations. Hydrophobic and hydrophilic regions are shown in yellow and cyan, respectively. The hydrophobic surface area of the OMA-bound STING (9221 Å²) was markedly greater than that of the cGAMP-bound complex (7448 Å²). **f** Secondary structure analysis revealed that OMA binding substantially reduced the proportion of ordered structures compared to the cGAMP-bound STING, suggesting ligand-induced destabilization of secondary structure. **g** Radius of gyration (Rg) analysis showed that OMA binding increased both the mean Rg and its fluctuations, indicating a more relaxed and less stable overall protein conformation. OMA denotes Omaveloxolone; Δ*T*_m_ refers to the shift of protein melting temperature. Source data are provided as a Source Data file.

We previously observed that Omaveloxolone markedly reduces the thermal stability of hSTING^WT^. To extend this observation, we assessed its impact on the thermal stability across multiple human GOF STING mutants (hSTING N154S, V155M, R281Q, and R284G) and mouse STING (mSTING). Omaveloxolone consistently decreased thermal stability in all tested STING proteins (Fig. 4a and Supplementary Fig. 3a–e). In contrast, the molecular glue degrader AK59 increased STING thermal stability (Fig. 4b), a striking difference indicating Omaveloxolone likely promotes STING degradation via a mechanism distinct from AK59.

The molecular crowbar mechanism in TPD is defined by small molecules that directly bind target proteins, inducing conformational destabilization and reduced thermal stability to facilitate recognition by E3 ubiquitin ligases and subsequent proteasomal degradation^50^. To evaluate whether Omaveloxolone fits this paradigm, we performed extensive microsecond-scale MD simulations of hSTING-ligand complex. Relative to STING bound to the endogenous ligand cGAMP or the apo state, Omaveloxolone strongly inhibited closure of the STING LBD, maintaining a more open conformation (Fig. 4c). This conformational shift significantly increased solvent-accessible surface area (SASA; Fig. 4d) and exposed additional hydrophobic regions (Fig. 4e). Notably, the hydrophobic surface area of Omaveloxolone-bound STING reached 9221 Å²—substantially higher than the 7448 Å² observed in the cGAMP-bound state—indicating a more relaxed conformation with enhanced hydrophobic exposure, which may facilitate recognition by the ubiquitin-proteasome system. Further analysis revealed that Omaveloxolone binding disrupted the secondary structural stability of the “lid” subdomain within the STING LBD (Fig. 4f) and increased both the radius of gyration (Rg) and its fluctuation (Fig. 4g), consistent with overall conformational loosening and destabilization. HDX-MS analysis further supported the conformational perturbation observed in MD simulations, revealing increased deuterium uptake in several regions within the STING LBD upon Omaveloxolone binding (Supplementary Fig. 3f–j). Collectively, these findings indicate that Omaveloxolone compromises STING conformational stability by hindering LBD closure, increasing hydrophobic exposure, and perturbing local secondary structure organization, consistent with a molecular crowbar-like mode of degradation driven by conformational perturbation. These findings provide structural and dynamic insights into Omaveloxolone’s function as a STING degrader.

Our team previously synthesized a library of Oleanolic acid derivatives and identified multiple Omaveloxolone analogs that competitively bind the STING ligand-binding pocket (via competition with the agonist diABZI) with comparable affinities (Table 1 and Supplementary Fig. 12). Despite similar binding capacities, these analogs exhibited substantially divergent STING-degrading ability. This activity correlated tightly with their ability to destabilize STING thermal stability: only analogs that significantly reduced STING stability displayed potent STING-degrading activity (Table 1 and Supplementary Fig. 4a–c). These observations indicate that STING binding alone is insufficient for degradation; instead, the combination of binding and induction of conformational instability is critical.

**Table 1:** STING degradation activity, binding activity, and destabilization activity of Omaveloxolone analogs

Compound	*R*^1^	*R*^2^	DC_50_ (nM)	*D*_max_ (%)	IC_50_ (μM)	Δ*T*_m_ (°C)
Omaveloxolone	CN		118 ± 9	98.6	1.53 ± 0.22	–4.0
RTA 402	CN		117 ± 14	99.3	6.53 ± 0.44	–4.0
RTA 405	CN		384 ± 46	84.7	4.41 ± 0.07	–4.0
LPH-A33	CN		222 ± 23	98.2	2.60 ± 0.27	–5.0
LPH-A40	CN		169 ± 8	98.2	2.71 ± 0.19	–4.0
LPH-A43	CN		351 ± 44	96.0	3.04 ± 0.16	–5.0
D1-1	CN		>1000	/	4.60 ± 0.69	–1.0
D5-1	CN		>1000	/	1.95 ± 0.09	–0.5
D6-1	CN		>1000	/	2.36 ± 0.20	–1.0
D6-2	CN		>1000	/	1.69 ± 0.46	–0.5
LPH-A107	CHO		>1000	/	3.33 ± 0.24	–1.0
LPH-A134	CN		>1000	/	3.24 ± 0.26	–1.0
D17			>1000	/	3.56 ± 0.27	–0.5

DC_50_ (half-maximal degradation concentration) values were determined in THP-1 cells and were presented as mean ± SD from three independent experiments. *D*_max_ represents the maximal degradation efficacy of indicated compounds. IC_50_ (half-maximal inhibitory concentration) values were measured using FP assay and were presented as mean ± SD from four independent biological replicates, reflecting the compounds’ competitive binding to STING LBD. Δ*T*ₘ indicates the change in thermal stability (melting temperature) of hSTING (2 μM) in the presence of the indicated compounds (1 μM).

To dissect why analogs that bind to STING but lack degradation effects are deficient in degrading activity, we performed MD simulations using the STING-Omaveloxolone complex as a reference, focusing on two representative inactive compounds (D5-1 and LPH-A134). Simulations revealed that these analogs bound at positions shifted upward relative to Omaveloxolone, deviating from the critical anchoring region at the LBD base (Supplementary Figs. 4d and 5a, b). Consequently, the LBD tended to reclose and failed to maintain the open conformation induced by Omaveloxolone (Supplementary Fig. 4e), without marked increases in hydrophobic exposure or conformational destabilization. Further binding-mode analyses showed that D5-1 and LPH-A134 adopt upward-shifted poses within the STING LBD pocket and form additional contacts, including hydrogen bonding with D237 in the upper β-sheet lid region (Supplementary Fig. 5c, d). These upper-pocket interactions, together with conserved contacts mediated by the shared core scaffold, may partially offset the loss of deep LBD-base anchoring interactions and help preserve their binding affinity. However, this altered binding geometry appears less effective in inducing the pose-dependent conformational perturbation required for productive crowbar-like destabilization. In particular, increased engagement with the upper lid region and reduced deep anchoring are consistent with the observed tendency of the LBD to reclose, rather than sustaining the open and destabilized conformation induced by Omaveloxolone. Thus, apparent pocket occupancy alone does not determine degradation activity; productive degradation requires a binding geometry capable of coupling ligand engagement to LBD destabilization.

In summary, Omaveloxolone engages the STING ligand-binding pocket and induces conformational instability and hydrophobic exposure, supporting a molecular crowbar-like model for STING ubiquitination and proteasomal degradation.

### Omaveloxolone promotes STING ubiquitination and degradation via the E3 ubiquitin ligase HUWE1

**Fig. 5:**
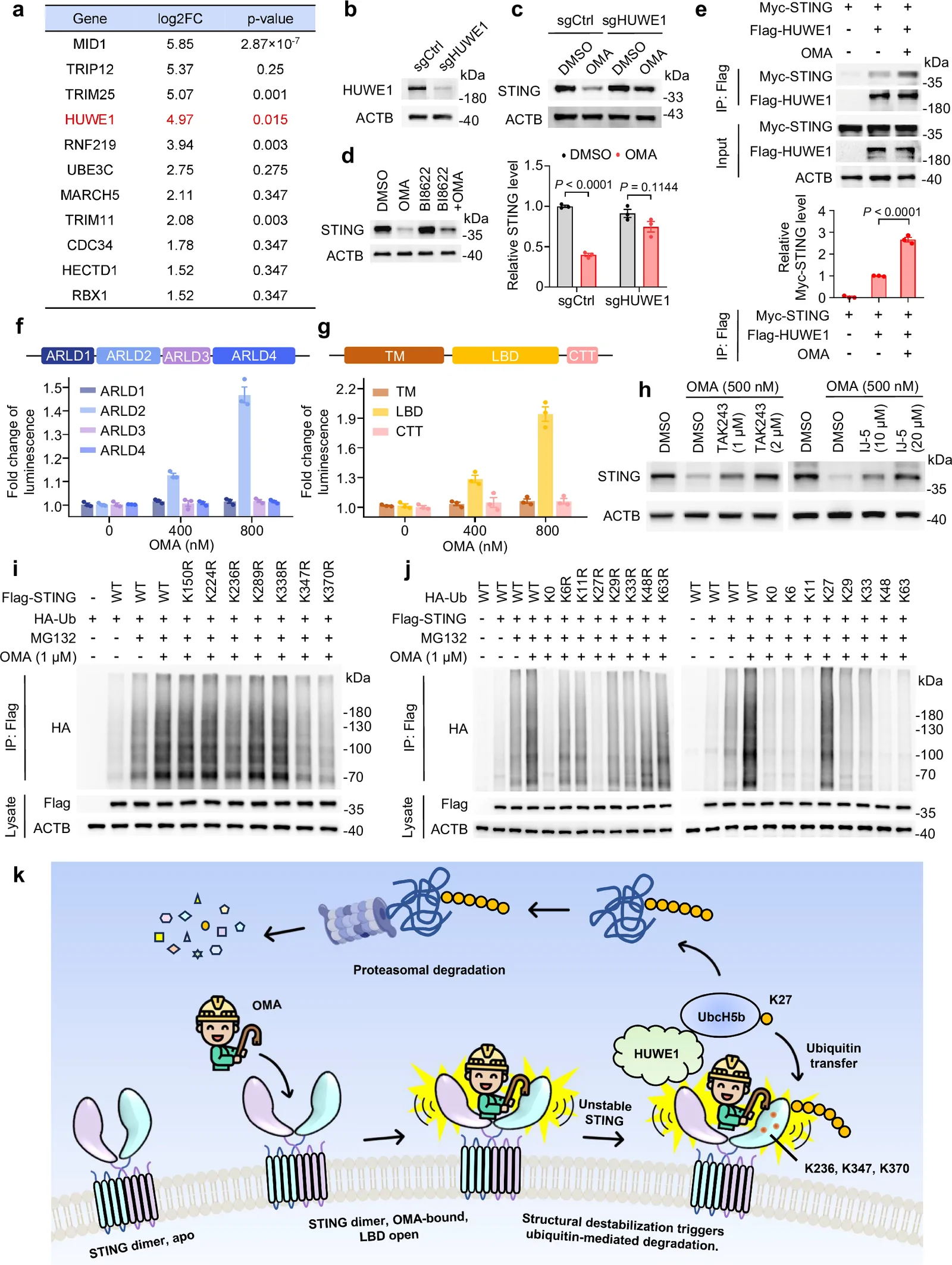
Omaveloxolone promotes STING ubiquitination and degradation via the E3 ubiquitin ligase HUWE1. **a** IP-MS screening identified candidate E3 ligases for OMA-induced STING degradation. **b** Western blot analysis of HUWE1 protein levels in THP-1 cells transduced with control or *HUWE1*-targeting sgRNA. Data are representative of three independent experiments with similar results. **c** STING protein levels in WT or *HUWE1*-knockout THP-1 cells treated with OMA (350 nM, 12 h), assessed by western blot. Quantitative analysis of STING levels is presented below (*n* = 3 independent experiments, mean ± SEM). **d** STING protein levels in THP-1 cells pretreated with HUWE1 inhibitor BI8622 (20 μM, 1 h) followed by OMA (500 nM, 6 h) treatment, assessed by western blot. Data are representative of three independent experiments with similar results. **e** Co-IP assay detecting the interaction between STING and HUWE1. HEK293T cells were transfected with Myc-STING and Flag-HUWE1 plasmids for 24 h. After pretreatment with PR171 (2 μM) for 1 h, cells were treated with OMA (350 nM) for 6 h. Lysate samples were run on separate gels with ACTB as loading control. Quantitative analysis of Myc-STING levels is presented below (*n* = 3 independent experiments, mean ± SEM). **f** NanoBiT assay evaluating the OMA-induced interactions between full-length STING-LgBiT and HUWE1-SmBiT truncations (ARLD1, ARLD2, ARLD3, ARLD4) in HEK293T cells (*n* = 3 independent biological replicates, mean ± SEM). **g** NanoBiT assay evaluating the OMA-induced interactions between HUWE1-ARLD2-SmBiT and STING-LgBiT truncations (TM, LBD, CTT) in HEK293T cells (*n* = 3 independent biological replicates, mean ± SEM). **h** Western blot analysis of STING degradation in THP-1 cells pretreated with UBA1 inhibitor TAK-243 or UbcH5b inhibitor IJ-5 for 1 h, followed by OMA treatment for 6 h. Data are representative of three independent experiments with similar results. **i**, Effects of lysine-to-arginine point mutations in STING (Flag-STING-KxR, x = 150/224/236/289/338/347/370) on OMA-induced STING ubiquitination. Lysate samples were run on separate gels with ACTB as loading control. Data are representative of three independent experiments with similar results. **j** Effects of lysine mutations in ubiquitin on OMA-induced STING ubiquitination. Each lysine residue in ubiquitin was mutated to arginine individually (HA-Ub-KxR, x = 6, 11, 27, 29, 33, 48, 63) (left panel) or only one specific lysine residue was preserved while all others were mutated to arginine (HA-Ub-Kx) (right panel) for ubiquitination analysis. Lysate samples were run on separate gels with ACTB as a loading control. Data are representative of three independent experiments with similar results. **k** Schematic illustration of the mechanism by which Omaveloxolone degrades STING. The schematic was assembled by the authors using elements created by Figdraw.com and crowbar icon by Eucalyp: Flaticon.com. OMA denotes Omaveloxolone. *P* values were calculated using two-sided unpaired Student’s *t*-tests. Source data are provided as a Source Data file.

Molecular crowbar can destabilize target protein conformations, rendering them more susceptible to recognition by E3 ubiquitin ligases^50^. To identify the specific E3 ligase responsible for Omaveloxolone-induced STING degradation, we performed immunoprecipitation coupled with mass spectrometry (IP-MS) screening. Comparative analysis revealed four E3 ligases—MID1, TRIM25, HUWE1, and RNF219—that were significantly enriched in Omaveloxolone-treated samples relative to DMSO controls (Log2FC > 3, *P* < 0.05) (Fig. 5a). Further sgRNA-mediated knockout of the candidate E3 ubiquitin ligases in THP-1 cells demonstrated that only HUWE1 deletion significantly blocked Omaveloxolone-induced STING degradation (Fig. 5b, c), whereas knockout of the other three candidate ligases had no obvious effect (Supplementary Fig. 6a–f). This specificity was further confirmed by pharmacological inhibition of HUWE1 using BI8622, which effectively reversed Omaveloxolone-induced STING degradation (Fig. 5d). These findings together confirmed HUWE1 as the key E3 ubiquitin ligase mediating STING degradation induced by Omaveloxolone.

To determine whether Omaveloxolone promotes the interaction between STING and HUWE1, we performed co-immunoprecipitation (Co-IP) assays. While a weak basal interaction between STING and HUWE1 was detectable under control conditions, Omaveloxolone treatment markedly enhanced this interaction (Fig. 5e). This observation was further corroborated by the NanoBiT complementation assay, which specifically demonstrated enhanced binding between the HUWE1 ARLD2 domain and STING LBD upon Omaveloxolone treatment (Fig. 5f, g). Moreover, microscale thermophoresis (MST) assays excluded direct binding between Omaveloxolone and HUWE1 (Supplementary Fig. 6g), indicating that Omaveloxolone promotes STING-HUWE1 interaction by binding to STING rather than HUWE1. These findings collectively demonstrated that Omaveloxolone facilitates STING recognition by HUWE1, consistent with a molecular crowbar-like model in which target protein destabilization promotes E3 ligase engagement. Previous studies have shown that the E1 ubiquitin-activating enzyme UBA1 and the E2 ubiquitin-conjugating enzyme UbcH5b are involved in HUWE1-mediated substrate ubiquitination^68–70^. Therefore, we further investigated whether these upstream components are also required for Omaveloxolone-induced STING degradation. Treatment of UBA1 inhibitor TAK-243 or UbcH5b inhibitor IJ-5 effectively abrogated Omaveloxolone-induced STING degradation (Fig. 5h), confirming that Omaveloxolone promotes STING ubiquitin-proteasome degradation through the canonical UBA1-UbcH5b-HUWE1 E1-E2-E3 cascade.

The degradation fate of ubiquitinated proteins is critically determined by both the specific lysine modification sites and the ubiquitin linkage types^71,72^. To characterize Omaveloxolone-induced STING ubiquitination, we performed comprehensive mutational analysis of seven lysine residues on STING (K150, K224, K236, K289, K338, K347, and K370) by substituting each with arginine. Among these mutations, only K236R, K347R, and K370R significantly attenuated STING ubiquitination (Fig. 5i), indicating that these three lysine residues are key sites for Omaveloxolone-induced STING ubiquitination. To further define the ubiquitin chain linkage type, we employed a series of ubiquitin mutants in which only one lysine residue was retained. Notably, the K27R mutant completely abrogated ubiquitination, while robust STING ubiquitination was observed exclusively with the K27-only ubiquitin mutant (HA-Ub-K27) (Fig. 5j). Consistently, endogenous ubiquitination assays in THP-1 cells revealed a K27-linked ubiquitin signal on endogenous STING (Supplementary Fig. 6h), further supporting that Omaveloxolone induces K27-linked polyubiquitination. Collectively, these results demonstrate that Omaveloxolone preferentially promotes K27-linked polyubiquitin chain assembly on STING, primarily at K236, K347, and K370.

In summary, the data support a model in which Omaveloxolone binds to the ligand-binding domain of STING, destabilizes its conformation, and facilitates recognition by the E3 ubiquitin ligase HUWE1. This interaction leads to K27-linked polyubiquitination at K236, K347, and K370, ultimately resulting in STING degradation by the proteasome (Fig. 5k).

### Omaveloxolone effectively degrades STING and inhibits the activation of downstream pathways both in vitro and in vivo

**Fig. 6:**
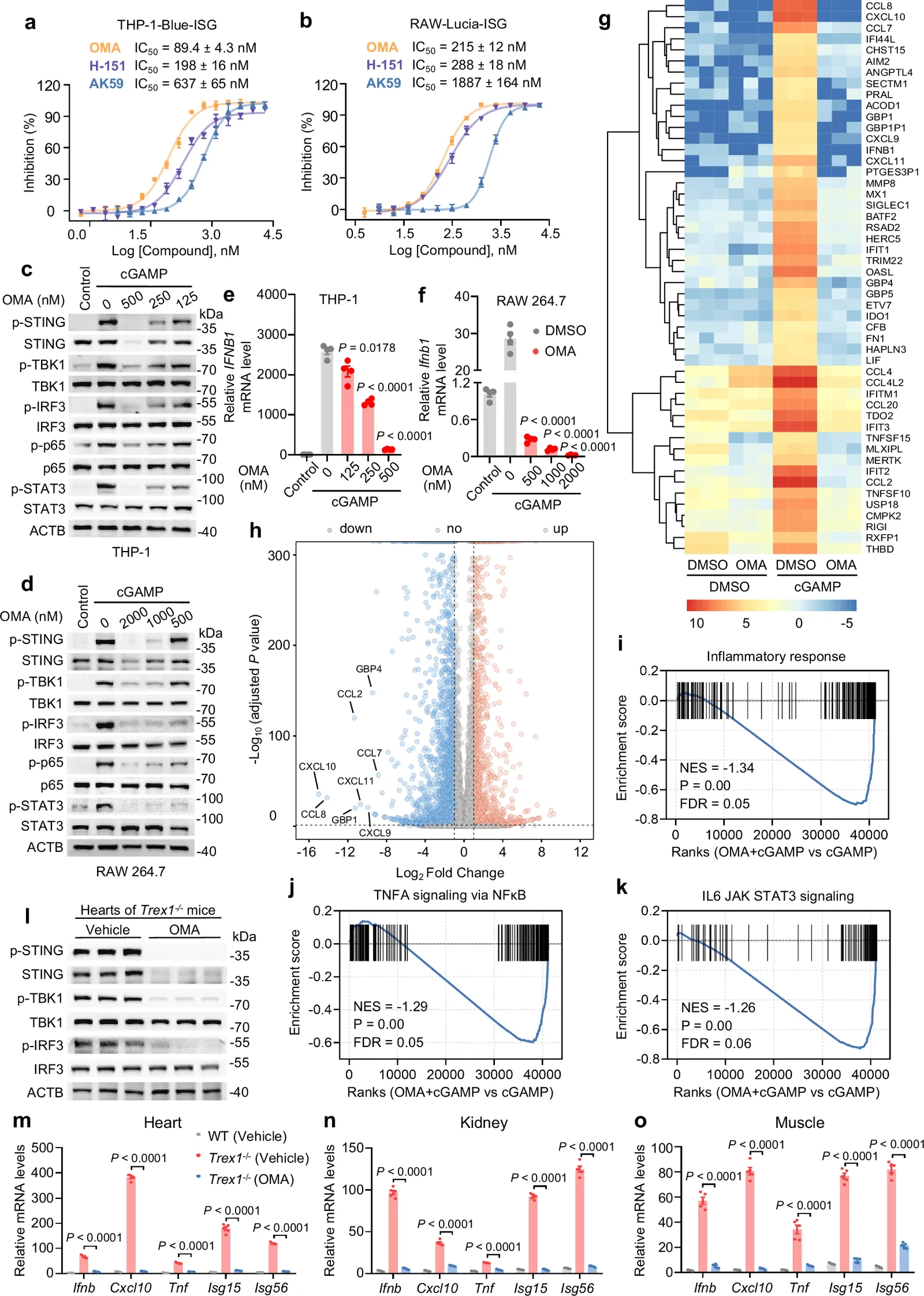
Omaveloxolone effectively degrades STING and inhibits the activation of downstream pathways both in vitro and in vivo. **a** Inhibition of cGAMP-induced STING signaling by OMA, H-151 and AK59 in THP-1-Blue-ISG-derived macrophages. Cells were treated with compounds for 12 h, followed by cGAMP (5 μM) stimulation for 6 h (*n* = 4 independent biological replicates, mean ± SD). **b** Inhibition of cGAMP-induced STING signaling by OMA, H-151 and AK59 in RAW-Lucia ISG cells. Cells were treated with compounds for 12 h, followed by cGAMP (10 μM) stimulation for 6 h (*n* = 3 independent biological replicates, mean ± SD). OMA dose-dependently reduced STING protein levels and the phosphorylation of STING, TBK1, IRF3, p65, and STAT3 in THP-1-derived macrophages (**c**) and RAW 264.7 cells (**d**). Cells were treated with indicated concentrations of OMA for 12 h, followed by cGAMP (5 μM for THP-1, 10 μM for RAW 264.7) stimulation for 2 h. Data are representative of three independent experiments with similar results. RT-qPCR analysis of *IFNB1* mRNA levels in THP-1-derived macrophages (**e**) and RAW 264.7 cells (**f**) treated with indicated concentrations of OMA for 12 h, followed by cGAMP stimulation (5 μM for THP-1, 10 μM for RAW 264.7) for 6 h (*n* = 4, two technical replicates from two independent experiments, mean ± SEM). *P* values were calculated using two-sided unpaired Student’s *t*-tests. Transcriptomic analysis of THP-1-derived macrophages treated with DMSO or OMA (500 nM) for 12 h, then stimulated with cGAMP (5 μM) or DMSO for 6 h. Differential gene expression is visualized via heatmap (**g**) and volcano plot (**h**). Statistical significance was assessed with the two-sided Wald test, and *P* values were adjusted for multiple comparisons using the Benjamini-Hochberg procedure. **i–k**, Gene set enrichment analysis (GSEA) comparing OMA + cGAMP co-treated group with cGAMP alone-treated group. OMA treatment significantly suppressed gene sets associated with the inflammatory response (**i**), TNFA signaling via NF-κB (**j**), and IL6-JAK-STAT3 signaling (**k**). Nominal *P* values and Benjamini-Hochberg-adjusted FDR values are provided. **l** OMA treatment (10 mg kg^-1^) reduces STING protein levels and phosphorylation of STING, TBK1 and IRF3 in heart tissues of *Trex1*^‒/‒^ C57BL/6 mice. Data are representative of three mice per group with similar results. RT-qPCR analysis of *Ifnb1*, *Cxcl10*, *Tnf*, *Isg15*, and *Isg56* mRNA levels in heart (**m**), kidney (**n**), and muscle (**o**) from mice. *Trex1*^‒/‒^ C57BL/6 mice were intraperitoneally administered OMA (10 mg kg^−1^) or vehicle for 21 days. Wild-type C57BL/6 mice received vehicle injections as controls (*n* = 5 mice/group, mean ± SEM). *P* values were calculated using two-sided unpaired Student’s *t*-tests. OMA denotes Omaveloxolone; IC_50_ refers to half-maximal inhibitory concentration. IC_50_ values were determined by fitting the dose-response curves (variable slope) through nonlinear regression analysis using Graphpad Prism 8.0.1. Source data are provided as a Source Data file.

Building on Omaveloxolone’s established role as a potent molecular crowbar-like degrader of STING, we comprehensively assessed its ability to degrade STING and suppress STING pathway activation in vitro and in vivo. Reporter gene assays revealed that Omaveloxolone inhibits cGAMP-induced activation of the STING signaling with promising potency (THP-1 IC_50_ = 89.4 nM, RAW 264.7 IC_50_ = 215 nM), surpassing the reported STING inhibitor H-151 and molecular glue degrader AK59 (Fig. 6a, b). Mechanistic characterization revealed that Omaveloxolone treatment led to dose-dependent suppression of critical downstream phosphorylation events (TBK1, IRF3, p65, STAT3) under cGAMP stimulation (Fig. 6c, d), accompanied by dose-dependent downregulation of STING downstream inflammatory genes such as *IFNB1* and *CXCL10* (Fig. 6e, f and Supplementary Fig. 7a, b). Transcriptomic profiling further uncovered Omaveloxolone’s broad attenuation of cGAMP-induced expression of interferons, interferon-stimulated genes (ISGs), chemokines, and pro-inflammatory cytokines (Fig. 6g, h). These findings were corroborated by gene set enrichment analysis (GSEA), which demonstrated marked suppression of key inflammatory gene signatures, including “Inflammatory Response”, “TNFA Signaling via NF-κB”, and “IL6-JAK-STAT3” gene sets (Fig. 6i–k). In summary, as a STING degrader, Omaveloxolone effectively suppresses STING pathway activation at the cellular level, outperforming previously reported inhibitors H-151 and AK59.

Given the potent STING-degrading and pathway-suppressing activity of Omaveloxolone at the cellular level, we further evaluated its in vivo pharmacological efficacy using a *Trex1*^-/-^ mouse model. *Trex1*-deficient mice lack cytosolic DNA exonuclease activity, resulting in sustained activation of the STING pathway and systemic inflammatory phenotypes. This model is therefore considered ideal for assessing the in vivo efficacy of STING inhibitors or degraders^73^. Upon treatment with Omaveloxolone, a significant reduction in STING protein levels was observed in the heart tissue of *Trex1*^-/-^ mice, along with marked decreases in phosphorylation of downstream effectors TBK1 and IRF3 (Fig. 6l). Moreover, Omaveloxolone significantly suppressed the mRNA expression of pro-inflammatory cytokines and ISGs, including *Ifnb1*, *Cxcl10*, *Tnf*, *Isg15*, and *Isg56*, in heart, kidney, and muscle tissues (Fig. 6m–o).

Taken together, our findings demonstrated that Omaveloxolone potently degrades STING and suppresses its downstream signaling both in vitro and in vivo, highlighting its therapeutic potential.

### Omaveloxolone attenuates aging-related inflammation in vivo

Inflammation is a key biological hallmark of aging and a central driver of aging-related tissue damage and disease. Emerging evidence indicates that aberrant activation of the STING signaling pathway plays a pivotal role in aging-associated inflammation and neurodegeneration^19,25–27^. Based on these insights, we explored the therapeutic potential of Omaveloxolone in mitigating aging-related inflammation and brain aging.

The ability of Omaveloxolone to counteract aging-related inflammation was first evaluated in Doxorubicin (Dox)- or ionizing radiation (IR)-induced senescence models using wild-type (WT) and STING-KO THP-1 and RAW 264.7 cells, as Dox and IR are well-established inducers of cellular senescence that recapitulate key features of aging-related inflammation, including SASP factor upregulation and pro-inflammatory cytokine secretion^30,74,75^. Omaveloxolone treatment significantly alleviated senescence-related inflammation, as evidenced by reduced mRNA levels of senescence markers (*P16*, *P21*) and pro-inflammatory SASP factors (*IL6*, *IL1B*), as well as a decreased proportion of senescence-associated β-galactosidase (SA-β-Gal)-positive cells (Supplementary Fig. 8a–d). In contrast, such effects were substantially diminished in STING-KO THP-1 and RAW 264.7 cells (Supplementary Fig. 8e–h), supporting that the ability of Omaveloxolone to ameliorate cellular senescence-associated inflammation is largely STING-dependent in vitro.

**Fig. 7:**
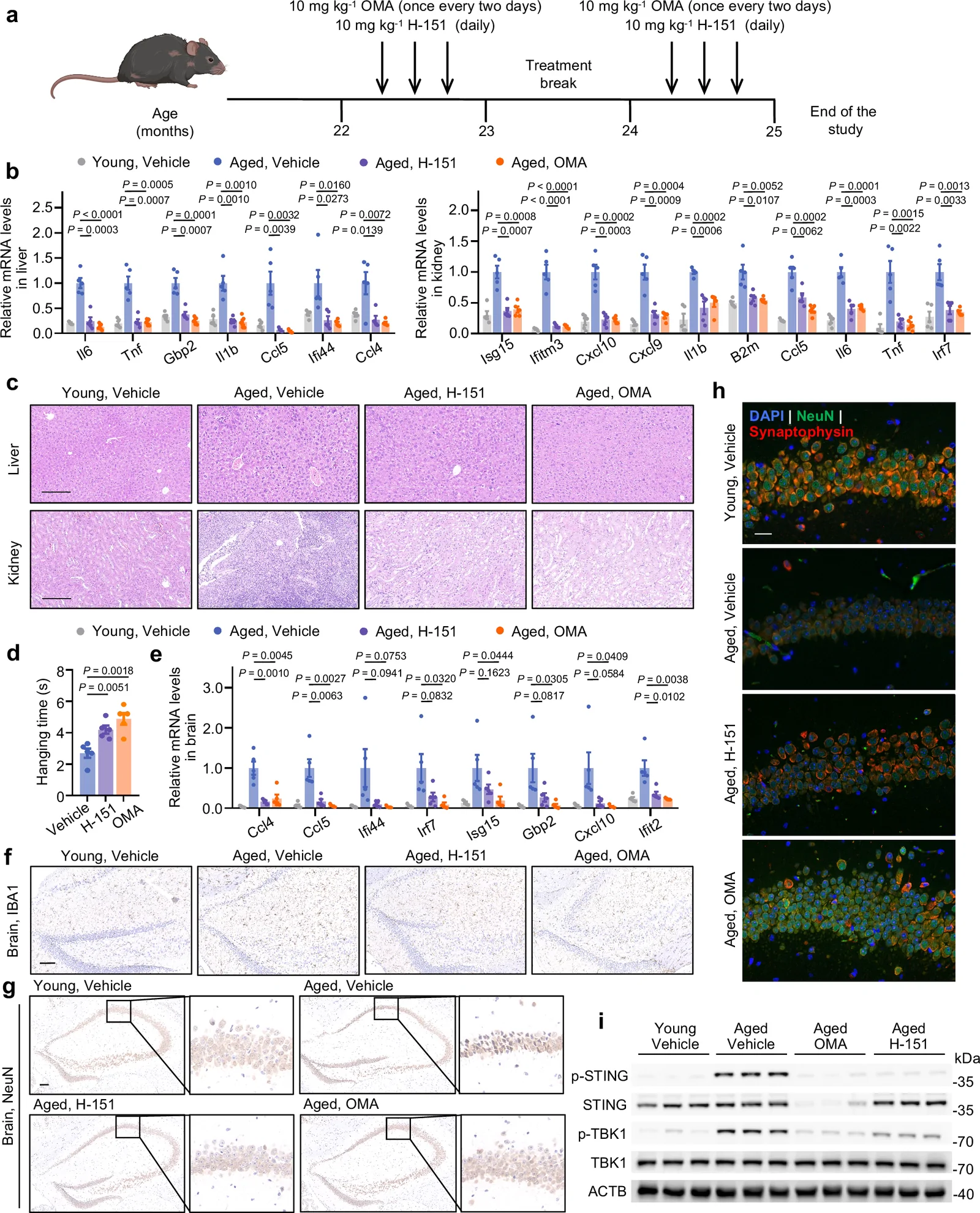
Omaveloxolone attenuates aging-related inflammation in vivo. **a** Schematic diagram of the treatment protocol in aged mice. The schematic was assembled by the authors using elements created in BioRender; Zhou, J. (2026) https://BioRender.com/29gmzuu. **b** RT-qPCR analysis of proinflammatory cytokines, chemokines, and ISGs in the liver and kidney of young mice, aged mice, and aged mice intraperitoneally administered H-151 or OMA (*n* = 5 mice/group). **c** H&E staining analysis of liver and kidney of young mice, aged mice, and aged mice intraperitoneally administered H-151 or OMA. Scale bars, 200 μm; representative of three mice per group with similar results. **d** Exercise capacity was assessed at the end of the study using an inverted grip-hanging test with time to fall off recorded (*n* = 5 mice/group). **e** RT-qPCR analysis of proinflammatory cytokines, chemokines, and ISGs in the brain of young, aged, and aged mice intraperitoneally administered H-151 or OMA (*n* = 5 mice/group). Representative images of hippocampal IBA1^+^ cells (**f**), NeuN^+^ cells (**g**), and synaptophysin intensity (**h**) in the CA1 region from brain sections of young mice, aged mice, aged mice intraperitoneally administered H-151 or OMA. Scale bars, 100 μm (**f**, **g**), 50 μm (**h**); representative of three mice per group with similar results. **i**, Western blot analysis of STING protein levels and phosphorylated STING and TBK1 in brain lysates from young mice, aged mice, and aged mice intraperitoneally administered H-151 or OMA (*n* = 3 independent biological replicates). OMA denotes Omaveloxolone. Data were presented as mean ± SEM. *P* values were calculated using two-sided unpaired Student’s *t*-tests. Source data are provided as a Source Data file.

We further conducted a long-term in vivo efficacy study in naturally aged mice starting at 22 months of age. Mice in the treatment group received intraperitoneal injections of Omaveloxolone (10 mg kg^−1^) every other day for one month, followed by a one-month treatment interval, and then resumed dosing until 25 months of age. The blood-brain barrier-permeable STING inhibitor H-151^26,27^ (10 mg kg^−1^, daily intraperitoneal injection) was used as a reference (Fig. 7a). Body weight monitoring throughout this chronic treatment period revealed no significant differences between the Omaveloxolone-treated and vehicle control groups (Supplementary Fig. 9), indicating the favorable tolerability of Omaveloxolone at the therapeutic dose. Compared to young mice, aged mice exhibited markedly elevated levels of pro-inflammatory cytokines, chemokines, and ISGs, along with increased inflammatory cell infiltration in the liver and kidney tissues (Fig. 7b, c). Treatment with either Omaveloxolone or H-151 significantly reduced these inflammatory markers (Fig. 7b, c). Moreover, both compounds improved physical function in aged mice, as evidenced by significantly enhanced muscle grip strength (Fig. 7d). Given that both Omaveloxolone and H-151 can penetrate the blood-brain barrier and distribute within brain tissue^26,27,36,37,54–56^, we further assessed inflammation levels in the mouse brain. The results showed that Omaveloxolone and H-151 significantly reduced various inflammatory factors, chemokines, and ISGs in the brain tissues of aged mice (Fig. 7e). Collectively, these findings demonstrated that Omaveloxolone effectively attenuates aging-related inflammation in both the peripheral and central nervous systems while improving physical function in aged mice.

Aberrant STING activation-induced inflammation and IFN signaling are important drivers of brain aging^19,25–27^. Therefore, we further examined the effect of Omaveloxolone on brain aging in aged mice. Histopathological analysis of the hippocampus revealed increased microgliosis characterized by increased IBA1^+^ cell density in aged mice, which was significantly attenuated by treatment with Omaveloxolone or H-151 (Fig. 7f). Moreover, Omaveloxolone prevented loss of neurons characterized by reduced NeuN^+^ cell counts in the hippocampal CA1 region of aged mice and increased local expression of synaptic activity marker Synaptophysin (Fig. 7g, h). Whole-brain lysate analysis showed that aged mice had significantly elevated levels of p-STING and p-TBK1 compared to young mice, these levels were reduced by both Omaveloxolone and H-151 treatments (Fig. 7i). Notably, Omaveloxolone reduced STING protein levels in the brains of aged mice, whereas H-151 had no significant effect on STING protein abundance (Fig. 7i). Collectively, these results demonstrated that STING signaling is activated in the brains of aged mice, and Omaveloxolone may alleviate brain aging by promoting STING protein degradation in brain tissue.

## Discussion

Targeted protein degradation has established itself as a transformative approach in modern drug discovery, enabling the selective elimination of pathogenic proteins. Among emerging strategies, molecular crowbars function through direct binding to the target protein, exerting a crowbar-like effect that disrupts secondary structure, induces conformational destabilization, and exposes hydrophobic regions—triggering recognition and degradation by the ubiquitin-proteasome system^50^.

This mechanism stands in stark contrast to established paradigms: PROTACs and certain molecular glues rely on bifunctional molecules to bridge E3 ubiquitin ligases and target proteins, forming stable ternary complexes to mediate ubiquitination; other molecular glues induce conformational changes in E3 ligases to recruit substrates^41–43^. In contrast, molecular crowbar degraders act directly on the target protein, inducing conformational disruption and hydrophobic exposure to facilitate E3 ligase recognition—representing a fundamentally distinct protein degradation strategy.

To date, this field remains nascent, with only one molecular crowbar degrader reported: a covalent agent targeting the pancreatic cancer-associated protein Pin1^50^. This molecule forms a covalent adduct with Pin1’s Cys113, inserting like a crowbar into the binding pocket to destabilize the short D-helix, induce unfolding, and expose hydrophobic residues, thereby triggering ubiquitination and degradation across multiple human cancer cell lines. Importantly, critical knowledge gaps persist: the universality of the molecular crowbar mechanism for other targets is unproven, and applicability to non-covalently binding molecules—distinct from the covalent Pin1 degrader—has yet to be explored.

In this study, we identified Omaveloxolone—an FDA-approved drug for Friedreich’s ataxia—as a potent STING degrader, with DC_50_ of 118 nM and *D*_max_ of 98.6%. Mechanistic analyses confirmed the absence of covalent adducts between Omaveloxolone and STING, while its degradation mode is consistent with defining characteristics of the molecular crowbar-like mechanism: it binds to a critical region at the base of STING’s ligand-binding pocket, induces conformational loosening and exposure of hydrophobic surfaces, and enhances recognition by the E3 ubiquitin ligase HUWE1. This interaction triggers K27-linked polyubiquitination of STING at K236, K347, and K370, ultimately leading to proteasomal degradation. These findings expand the target repertoire of potential molecular crowbar degraders and support that non-covalent binding can mediate this degradation mechanism. While we provide experimental and computational evidence for Omaveloxolone’s binding and destabilizing effect on STING, the current lack of a high-resolution Omaveloxolone-STING complex structure represents a limitation of this study, and future structural biology efforts will be required to further define the precise binding mode.

Compared to reported STING PROTACs (i.e., UNC903, SD02, SP23), Omaveloxolone demonstrates superior degradation potency, lower molecular weight, and effective activity against SAVI-associated STING mutants N154S and V155M (DC_50_ = 163 nM and 194 nM; *D*_max_ = 88.2% and 84.4%, respectively), supporting its therapeutic potential for autoinflammatory diseases driven by STING GOF mutations. Relative to Novartis’ first-in-class STING molecular glue degrader AK59, Omaveloxolone offers enhanced degradation efficacy and benefits from well-established clinical safety profiles, underscoring its promising translational potential.

Notably, Omaveloxolone is well characterized as an NRF2 activator that covalently modifies KEAP1 to stabilize NRF2 and subsequently induce transcription of canonical antioxidant target genes. In this study, we identified Omaveloxolone as a STING degrader. Mechanistically, Omaveloxolone-induced STING degradation occurs at the proteasomal level and is independent of NRF2 (Fig. 2e). Importantly, proteomic analysis in STING-KO-THP-1 cells revealed that Omaveloxolone still robustly upregulates the canonical NRF2 downstream targets HMOX1 and SRXN1 to levels comparable to those in WT cells, confirming that NRF2 activation does not rely on STING expression or degradation (Fig. 2h and Supplementary Fig. 2a). Collectively, Omaveloxolone acts as a dual-function modulator capable of independently regulating both the NRF2 and STING signaling axes.

This dual-target property may provide pharmacological complementarity for two core pathological drivers of aging. Aging is characterized by chronic low‑grade inflammation (“inflammaging”), to which STING contributes substantially^26,76^, as well as by impaired antioxidant capacity, which is largely governed by NRF2^77–79^. Omaveloxolone can simultaneously modulate both aging-associated pathway dysregulations: on one hand, it directly degrades STING, blocking inflammatory signaling cascades at their source; on the other hand, it activates NRF2, enhancing cellular antioxidant defense. Such a dual regulatory strategy combining anti-inflammatory and antioxidant effects may address two interconnected pathogenic pillars of inflammaging and enable functional synergy between the two pathways.

Although our cellular senescence models indicate that Omaveloxolone modulates cellular senescence-related inflammation primarily in a STING-dependent manner (Supplementary Fig. 8), the in vivo microenvironment of naturally aged mice is more complex, where STING-driven hyperinflammation coexists with impaired NRF2-mediated antioxidant capacity. Therefore, we cannot rule out the possibility that the in vivo protective effects of Omaveloxolone against aging-related inflammation may also be partially attributed to NRF2 activation. This represents a limitation of the present study regarding the in vivo pharmacological mechanism.

Our proteomic profiling in THP-1 cells further revealed that Omaveloxolone concurrently downregulates several other proteins with established functions in immune regulation and nucleic acid metabolism, including TDO2^80^, USP3^81^, ARMC6^82^, IL1B^83^, SETX^84^, and MAFB^85,86^ (Fig. 2h). Subsequent proteomic analysis using STING-KO THP-1 cells demonstrated distinct regulatory patterns for these targets. The Omaveloxolone-induced downregulation of ARMC6, SETX, and USP3 remained significant in STING-KO THP-1 cells (Supplementary Fig. 2a), indicating that their downregulation occurs largely through STING-independent mechanisms. In contrast, the downregulation of MAFB, TDO2, and IL1B was abolished upon STING deletion, demonstrating that the suppression of these three proteins is STING-dependent. Notably, IL1B serves as a canonical pro-inflammatory downstream effector of NF-κB signaling^83^, while TDO2 functions as a key immunomodulatory enzyme induced by inflammatory stress^80^. Pro-inflammatory cytokines, including IL1B can induce TDO2 expression^87^, and TDO2-derived metabolites can in turn modulate inflammatory signaling^88^. The coordinated STING-dependent downregulation of these two factors may collectively contribute to the protective effects of Omaveloxolone against aging-related inflammation.

Currently, Omaveloxolone is FDA-approved for Friedreich’s ataxia in patients aged 16 years and above, and is being evaluated in multiple clinical trials. However, studies exploring its utility in aging-related diseases remain limited. In our work, we demonstrate that Omaveloxolone effectively mitigates aging-associated inflammation in both the peripheral and central nervous system of mouse models, ameliorates brain aging phenotypes, and enhances physical performance. These results support Omaveloxolone’s potential utility as a therapeutic for aging-associated disorders and provide a critical preclinical foundation for expanding its clinical indications.

In summary, this study has made three interconnected and significant advancements. First, it reveals Omaveloxolone as a potent STING degrader, extending its therapeutic potential to aging-related inflammatory diseases and holding promise for further expanding its clinical indications. Second, it uncovers the mechanism by which Omaveloxolone degrades STING as a molecular crowbar-like degrader, providing a mechanistic framework for targeting STING through conformational perturbation. Third, it expands the target range of molecular crowbar degraders, and more importantly, supports the possibility that non-covalent binding can mediate the molecular crowbar-like degradation mechanism, which represents a key breakthrough in this field.

## Methods

### Compounds

Omaveloxolone (HY-12212), cGAMP (HY-100564A), RTA 401 (HY-14909), RTA 402 (HY-13324), RTA dh404 (HY-112671), RTA 405 (HY-12213), Oleanonic acid (HY-N0156), AK59 (HY-162563), H-151 (HY-112693), SR-717 (HY-131454), TAK-243 (HY-100487), IJ-5 (HY-124670), BI8622 (HY-120929), PR171 (HY-10455), PS341 (HY-10227), MG132 (HY-13259), Chloroquine (HY-17589A), Bafilomycin A1 (HY-100558) used in this article were purchased from MedChemExpress (MCE).

Omaveloxolone analogs (LPH-A33, LPH-A40, LPH-A43, LPH-A107, LPH-A134, D1-1, D5-1, D6-1, D6-2, and D17) were synthesized in-house. All final compounds were characterized by ^1^H NMR, ^13^C NMR, and HRMS, and their purity (≥95%) was verified by HPLC. The synthetic routes (Supplementary Figs. 13–17) and ^1^H NMR, ^13^C NMR, HRMS, and HPLC spectra of all final compounds are shown in the Supporting Information.

### Cell lines

THP-1 human myeloid leukemia mononuclear cells (TIB-202) were obtained from American Type Culture Collection. RAW 264.7 mouse macrophage cells (SCSP-5036) were obtained from Cell Bank of the Chinese Academy of Sciences. HEK293T human embryonic kidney cells (CL-0005) and L929 mouse fibroblast cells (CL-0137) were obtained from Procell Life Science & Technology Co., Ltd. The cell lines THP-1-Blue ISG (thp-isg), THP-1-KO-STING (thpd-kostg), RAW-Lucia ISG (rawl-isg), RAW-KO-STING (rawl-kostg) were purchased from InvivoGen. THP-1, THP-1-Blue ISG, and THP-1-KO-STING cells were cultured in RPMI 1640 medium (Meilunbio, MA0215) supplemented with 10% fetal bovine serum (FBS, MeilunBio, PWL001), 1% penicillin-streptomycin (MeilunBio, MA0110) and 0.05 mM β-mercaptoethanol (Thermo Fisher Scientific, 21985023). HEK293T, RAW 264.7, RAW-Lucia ISG, and RAW-KO-STING cells were cultured in DMEM medium (Meilunbio, MA0212) supplemented with 10% FBS and 1% penicillin-streptomycin. L929 cells were cultured in MEM medium (BasalMedia, L540KJ) containing 10% FBS and 1% penicillin-streptomycin. THP-1-derived macrophages were generated by treating THP-1 cells with phorbol 12-myristate 13-acetate (PMA, 100 ng mL^−1^). To generate THP-1 cells expressing EGFP-STING, STING^N154S^, or STING^V155M^, THP-1-KO-STING cells were transduced with lentiviral plasmids encoding EGFP-STING-IRES-mCherry, STING^N154S^, or STING^V155M^, followed by selection with Puromycin (MeilunBio, MB2005-1). Gene knockout THP-1 cell lines were established by lentiviral transduction with plasmids containing target-specific sgRNAs (Synthesized by Synbio Technologies (Suzhou) Co., Ltd. Sequences provided in Supplementary Data 1). STING-KO cells were validated by Western blot (Supplementary Fig. 11). All cell lines were maintained at 37 °C in a humidified atmosphere containing 5% CO_2_.

### Flow cytometry

THP-1^EGFP-STING^ cells were treated with indicated compounds for 12 h. Following treatment, cells were washed and resuspended with phosphate-buffered saline (PBS) for flow cytometry analysis. Samples were acquired using a NovoCyte 3000 flow cytometer (Agilent), with EGFP-STING signals detected in the FITC channel (excitation, 488 nm; emission, 530 nm) and mCherry fluorescence measured in the PE-Texas Red channel (excitation, 488 nm; emission, 615 nm). The gating strategy for cell population analysis was detailed in Supplementary Fig. 10. Data analysis was performed using FlowJo 10.6.2 software.

### Western blot

Cells were collected, washed with PBS, and lysed on ice using RIPA lysis buffer (Beyotime, P0013C) containing protease inhibitor (Bimake, B14001) and phosphatase inhibitor (Bimake, B15001). After centrifugation (15,000 × *g*, 15 min, 4 °C), the supernatant protein concentration was quantified using a BCA assay kit (Thermo Fisher Scientific, 23225). Protein samples were denatured by heating with SDS-PAGE loading buffer (Beyotime, P0015L) at 100 °C for 5 min. Western blot analysis was performed according to standard procedures. Antibodies used included anti-p-STING (Ser366) (Cell Signaling Technology, CST, 50907), anti-p-STING (Ser365) (CST, 72971), anti-STING (CST, 13647), anti-p-TBK1 (Ser172) (CST, 5483), anti-TBK1 (CST, 3504), anti-p-IRF3 (Ser396) (CST, 29047), anti-IRF3 (CST, 11904), anti-NF-κB p-p65 (Ser536) (CST, 3033), anti-NF-κB p65 (CST, 8242), anti-p-STAT3 (Tyr705) (CST, 9145), anti-STAT3 (CST, 12640), anti-NRF2 (CST, 12721), anti-HA-Tag (CST, 3724), anti-Flag-Tag (CST, 2368), anti-Myc-Tag (CST, 2278), anti-K27-linkage specific ubiquitin (Abcam, ab181537), anti-β-Actin (CST, 4970), anti-GAPDH (CST, 2118), anti-β-Tubulin (Starter, S0B0238), anti-HUWE1 (CST, 5695), anti-TRIM25 (CST, 13773), anti-MID1 (Immunoway, YT2760), anti-RNF219 (Proteintech, 24988-1-AP), HRP-conjugated anti-rabbit IgG (Promega, W4011), HRP-conjugated anti-mouse IgG (Promega, W4021). A detailed information list of all the antibodies used is provided in Supplementary Data 2. Uncropped and unprocessed scans of blots are available in the Source Data file.

### Cytotoxicity assay

Cells were seeded in 96-well culture plates at a density of 5 × 10^4^ cells/well and treated with the indicated compounds for 24 h in a final volume of 100 μL. Following treatment, cell viability was assessed using the CellTiter-Glo Luminescent Cell Viability Assay (Promega, G7572) according to the manufacturer’s protocol. Luminescence was quantified using a Tecan Spark multifunctional microplate reader.

### Real-time quantitative PCR (RT-qPCR)

Total RNA was isolated from cells or tissues using RNA-easy Isolation Reagent (Vazyme, R701-01). Reverse transcription was performed using 2 μg total RNA with HiScript III qRT SuperMix (Vazyme, R323-01) according to the manufacturer’s protocol. RT-qPCR was performed using ChamQ SYBR qPCR Master Mix (Vazyme, Q331-03) on a QuantStudio 5 Real-Time PCR System (Applied Biosystems). Primers were synthesized by Synbio Technologies (Suzhou) Co., Ltd. Sequences are provided in Supplementary Data 1.

### Protein expression and purification

The DNA sequences encoding hSTING (residues 139–379) and mSTING (residues 144–378) were cloned into pET-28a vectors containing an N-terminal 6×His-SUMO tag. The plasmids were transformed into *Escherichia coli* BL21 (DE3) competent cells for protein expression. The single transformants were grown in Luria-Bertani (LB) medium at 37 °C until OD_600_ reached 0.6-0.7. Protein expression was induced with 0.2 mM isopropyl β-D-1-thiogalactopyranoside (IPTG) at 16 °C for 16 h. Cells were harvested and lysed by sonification in lysis buffer (20 mM HEPES, pH 7.4, 200 mM NaCl, 10 mM imidazole, 1 mM TCEP, 1 mM PMSF). After centrifugation and removal of cell debris, the supernatant was loaded onto Histrap HP columns (Cytiva) and then eluted with a gradient of imidazole up to 300 mM. The eluted protein was incubated with ULP1 protease at 4 °C overnight to cleave the SUMO tag, followed by further purification using a Superdex 75 Increase 10/300 GL column (Cytiva) pre-equilibrated with buffer containing 20 mM HEPES (pH 7.4) and 200 mM NaCl. Purified proteins were concentrated, aliquoted, flash-frozen in liquid nitrogen, and stored at −80 °C. All mutant proteins were purified using the same protocol. Plasmids were synthesized by Synbio Technologies (Suzhou) Co., Ltd.

### Fluorescence polarization (FP) assay

The competitive binding of test compounds to STING proteins was determined by FP assay. Serially diluted compounds were transferred into the black 384-well microplates (Corning, 3575). STING proteins (400 nM) in assay buffer (20 mM HEPES, pH 7.4) were added to each well and incubated for 30 min at room temperature. The fluorescent tracer 5-FAM-labeled diABZI (360 nM final concentration) was then added with a final reaction volume of 40 μL per well, followed by an additional 1 h incubation at room temperature. FP values were measured using a Tecan Spark multifunctional microplate reader (excitation, 485 nm; emission, 535 nm).

### Protein thermal shift (PTS) assay

The thermal stability of STING proteins was determined by PTS assay. STING proteins (2 μM) were incubated with test compounds and 5× SYPRO Orange (Merck, S5692) in assay buffer (20 mM HEPES, pH 7.4, 200 mM NaCl) with a final reaction volume of 20 μL in 96-well low-profile PCR plates (DN Biotech, 5371012). Samples were subjected to a temperature gradient from 25 to 90 °C using the CFX96 Real-Time PCR Detection System (Bio-Rad). The fluorescence signals were continuously tracked and recorded. The *T*_m_ values were calculated by the CFX Manager 3.1 software (Bio-Rad).

### Isothermal titration calorimetry (ITC)

ITC experiment was performed at 25 °C using a MicroCal PEAQ-ITC instrument (Malvern). STING protein (100 μM) was loaded into the syringe, while Omaveloxolone (10 μM) was placed in the sample cell. All components were diluted in ITC buffer (20 mM HEPES, pH 7.4, 200 mM NaCl) with consistent DMSO concentrations. A total of 20 injections were conducted with the first injection of 0.4 μL/0.8 s followed by 19 injections of 2 μL/4 s at 150 s intervals. Data processing was performed using the MicroCal PEAQ-ITC Analysis software.

### Proteomics

Sample preparation: THP-1 and STING-KO-THP-1 cells were treated with Omaveloxolone (500 nM) or DMSO for 12 h (three independent biological replicates per group). Following treatment, cells were washed three times with PBS and lysed in SDT buffer (2% SDS (w/v), 0.1 M DTT, 0.1 M Tris, pH 7.6). Lysates were heat-denatured (100 °C, 10 min), then centrifuged (15,000 × *g*, 15 min, 4 °C) to remove insoluble debris. The cell lysates were incubated for 10 min at 95 °C and centrifuged at 15,000 × *g* for 15 min. The supernatants were collected, and protein concentration was determined by tryptophan fluorescence emission assay as reported^89^. One hundred micrograms of proteins from each sample were alkylated (350 mM iodoacetamide, 45 min, 25 °C in the dark). Solvent precipitation SP3 (SP4) captures aggregated protein for proteomics sample clean-up^90^. One thousand micrograms inert glass beads (water suspended) were added in bead/protein ratio of 10:1 (w/w), then add 4 volumes of anhydrous acetonitrile and centrifuge to remove 90%–95% of the supernatant, wash with 80% ethanol for 3 times and dried in vacuum. Subsequently, trypsin was added to a 1:100 enzyme: protein ratio and digested overnight at 37 °C. The next day, 10% trifluoroacetic acid was added to stop the enzyme digestion, and the pH of the solution was adjusted to about 3.0. About 20 μg of peptides from each sample were desalted for LC-MS/MS analysis.

LC-MS/MS analysis: The DIA analysis was performed on hybrid TIMS quadrupole TOF mass spectrometer (Bruker timsTOF Pro) platform connected to an online nanoflow U3000 HPLC system (Thermo Fisher Scientific). For each sample, 100 ng of peptides were resolved using 0.1% formic acid and separated using a home-made reversed-phase C18 column (20 cm × 75 µm, ReproSil-Pur C18-AQ, 1.9 µm resin, Dr. Maisch GmbH, Germany) using a 60 min gradient at 300 nL/min. Mobile phase A consisted of 0.1% (v/v) formic acid in H_2_O and Mobile phase B consisted of 0.1% (v/v) formic acid in 80% acetonitrile. Liquid chromatography was coupled online to a hybrid TIMS quadrupole TOF mass spectrometer (Bruker timsTOF Pro) via a CaptiveSpray nano-electrospray ion source. The source parameters were: 1500 V of Capillary voltage, 3.0 L/min of dry gas, and 180 °C of dry temperature. To perform data-independent acquisition, samples were acquired with a diaPASEF method106 consisting of 14 cycles including a total of 28 mass width windows (25 Da width, from 452 to 1152 Da) with 4 mobility windows each, making a total of 56 windows covering the ion mobility range (1/K0) from 0.76 to 1.29 V s/cm2.

Database searching and data analysis: Raw data were processed using DIA-NN software (version 1.9.2)^91^ against the Uniprot human proteome database, with precursor FDR at 1%. Carbamidomethyl were selected as fixed modifications and N-terminal methionine excision enabled. Peptide length range was 7–30 and one missing cleavage site was allowed. Log level was set as 5. Quantification strategy was Any LC (high accuracy).

### Immunoprecipitation-mass spectrometry (IP-MS)

HEK293T cells were transfected with Flag-STING plasmid using PolyJet transfection reagent (SignaGen, SL100688) following the manufacturer’s protocol. 24 h post transfection, cells were treated with Omaveloxolone (1 μM) or DMSO control for an additional 24 h (three independent biological replicates per group). Cells were then harvested, washed three times with PBS, and lysed using IP lysis buffer (Beyotime, P0013). Lysates were cleared by centrifugation, and protein concentrations were determined using a BCA assay kit. Flag-STING and its interacting proteins were immunoprecipitated using anti-Flag magnetic beads (Selleck, B26102).

Samples were digested with trypsin (1:50, enzyme to protein in 50 mM NH_4_HCO_3_) by incubating at 37 °C for 16 h, and supernatant was collected, desalted, and dried using Speed-Vac. The LC-MS/MS analysis was performed on a Q-Exactive HF mass spectrometer (Thermo Fisher Scientific) connected to an online nanoflow EASY nLC1000 HPLC system (Thermo Fisher Scientific). Approximately 1 μg peptides of each sample was resolved in 0.1% formic acid and loaded onto a self-packed column (75 μm × 200 mm, 3 μm ReproSil C18 beads, Dr. Maisch GmbH, Germany). Then the peptides were separated with a 60 min gradient at a flow rate of 300 nL min^−1^ using solvent A (0.1% formic acid in H_2_O) and solvent B (0.1% formic acid in acetonitrile). The 60 min gradient was set as follows: 2%–4% B in 1 min; 4%–26% B in 42 min; 26%–32% B in 5 min; 32%–90% B in 2 min; 90% B for 9 min. The spray voltage was set at 2.0 kV in positive ion mode and the capillary was set at 300 °C. Data-dependent acquisition was performed with Xcalibur software. The full MS resolution was set as 60,000 for full MS scan (300–1700 m/z) and 15,000 for MS2 scan. The automatic gain control (AGC) targets of the ion trap were set to 3 e6 for full MS scan and 1 e5 for MS2 scan. The maximum ion injection time was set to 20 ms for full MS scan, 100 ms for MS2 scan. The normalized collision energy (NCE) was set at NCE 27% and the dynamic exclusion time was 30 s. Top 10 data-dependent MS2 scans were acquired by high-energy collision dissociation (HCD). The isolation window was set to 1.6 m/z.

Raw mass spectrometry data were analyzed using MaxQuant software version 1.6.17.0 against the UniProt Human database with a false discovery rate (FDR) < 0.01 at the levels of peptides and proteins. Other settings were set for peptide identification and quantification: Carbamidomethyl (C) as a fixed modification; oxidized methionine (M) and protein N-acetylation as variable modification; trypsin/P as the digestive enzyme.

### Mass spectrometry

For protein-ligand interaction analysis, STING protein (100 μM) was incubated with Omaveloxolone (1 mM) or DMSO control in assay buffer (20 mM HEPES, pH 7.4, 200 mM NaCl) and at 4 °C overnight. Intact protein was analyzed using an Agilent 1290 Infinity II LC coupled with a 6545 QTOF mass spectrometer (Agilent) as reported^92^.

### Protein structure preparation

To obtain a complete and reliable three-dimensional structure of STING for subsequent molecular simulations, several high-resolution crystal structures of human STING were retrieved from the Protein Data Bank (PDB), including entries 6NT5, 6MXE, and 8FLM. Based on the canonical human STING protein sequence from the UniProt database (UniProtKB: Q86WV6), all non-native point mutations in the crystal structures were reverted to the wild-type sequence. To reconstruct missing segments, homology modeling was performed using the Build Homology Model module in the Schrödinger Suite. Among the generated models, the conformation with the lowest energy was selected to represent the full-length STING structure. The resulting structure was then processed with the Protein Preparation Wizard^93^. Protonation states of all titratable residues, especially histidines, were carefully evaluated and adjusted. Hydrogen atoms were subsequently added under physiological pH conditions (pH 7.4) using the PROPKA tool, and the hydrogen-bonding network was optimized. A restrained energy minimization step was performed to relieve steric clashes and ensure structural stability. The final model provided a robust structural basis for downstream molecular docking, molecular dynamics simulations, and binding mechanism investigations.

### Molecular docking

Molecular docking was performed using the Glide module in the Schrödinger 2023 software suite to evaluate the interactions between Omaveloxolone and the STING protein. Previous studies have indicated that certain inhibitors targeting the LBD of STING can adopt a dual-ligand binding mode. To explore whether Omaveloxolone could adopt a similar binding mode, we used the crystal structure of STING (PDB ID: 6MXE) as a template, with the co-crystallized ligand (Compound 18) defining the reference binding pocket. The first Omaveloxolone molecule was docked and retained in the pocket, after which the second molecule was docked in the presence of the first ligand. The resulting dual-ligand complex was subjected to restrained minimization before MD simulations. Ligand structures were constructed in Maestro and subjected to conformational sampling using the integrated low-energy conformer search tool. During grid generation, a cubic docking grid of 18.0 × 18.0 × 18.0 Å³ was centered on the centroid of Compound 18 to ensure full coverage of the potential binding region. Flexible ligand docking was performed, and resulting poses were scored and ranked using GlideScore. The top-ranked pose was selected as the starting structure for subsequent MD simulations.

### MD simulation preparation

In this study, a lipid bilayer composed of DCPC was constructed using the CHARMM-GUI^94^ web server to model the conformational behavior of the STING protein in a membrane environment. The protein–ligand complex was pre-aligned according to the standard membrane orientation provided by the Orientations of Proteins in Membranes (OPM) database to ensure a physiologically relevant spatial configuration within the lipid bilayer. During system assembly, the protein-ligand complex was embedded into the pre-equilibrated DCPC bilayer, and explicit solvent was added using the TIP3P water model to create a fully solvated environment. The resulting system was enclosed in a cubic simulation box, and sufficient Na⁺ and Cl⁻ ions were added to maintain charge neutrality and achieve a physiological salt concentration of 0.15 M. All system components—including protein, lipids, water molecules, and ions—were parameterized using the CHARMM36 force field to ensure accurate representation of molecular interactions and dynamics. Ligand topologies and parameters were generated using the CHARMM General Force Field (CGenFF)^95^, ensuring force field compatibility across the entire system. This preparation provided a robust computational foundation for subsequent molecular dynamics simulations and mechanistic analysis of protein–ligand interactions.

### MD simulations

All MD simulations in this study were performed using GROMACS 2022.4^96^. Initially, each system underwent 5,000 steps of energy minimization using the steepest descent algorithm to eliminate unfavorable atomic contacts and reduce system potential energy, thereby providing a stable starting point for subsequent simulations. Following energy minimization, a six-step equilibration protocol was employed. During this process, position restraints were applied to the protein, lipid molecules, water, and ions at varying levels to gradually bring the system to thermodynamic equilibrium while preserving structural integrity. The equilibration included two stages under the constant volume and temperature ensemble (NVT), during which the system temperature was gradually increased from 0 to 300 K, followed by four stages under the constant pressure and temperature ensemble (NPT) to allow full adaptation to environmental pressure and volume changes. Upon completion of system equilibration, 1000 ns of unrestrained production MD simulations were performed for each system under the NPT ensemble, ensuring stable temperature and pressure control and enabling adequate conformational sampling. To comprehensively evaluate the binding behavior between Omaveloxolone and the STING protein, five representative STING-ligand complex systems were constructed and simulated, with detailed system compositions summarized in Supplementary Data 3. To enhance the statistical reliability of the simulations, each system was independently simulated in duplicate using different random velocity seeds, ensuring reproducibility and robustness of the results.

### Hydrogen-deuterium exchange mass spectrometry (HDX-MS)

STING protein was incubated with or without Omaveloxolone at a protein-to-ligand molar ratio of 1:50 for 1 h at room temperature (four technical replicates per group). Hydrogen-deuterium exchange was initiated by diluting the samples into deuterated buffer. The exchange reactions were then quenched at 0, 5, 10, 60, and 600 s by adding ice-cold quench buffer (4 M guanidine hydrochloride, 0.2 M citric acid, 0.2 M TCEP) to a final pH of 2.5. Quenched samples were immediately injected onto immobilized pepsin column (2.1 × 30 mm^2^) at a flow rate of 45 μL min^−1^ with 0.1% formic acid in H_2_O at 4 °C. Peptide fragments were collected on an Acclaim PepMap300 C18 column (5 μm, 1.0 × 15 mm, Thermo Fisher Scientific) for desalting with 0.1% formic acid in H_2_O, and then separated on an ACQUITY UPLC Peptide CSH C18 column (130 Å, 1.7 μm, 1 × 50 mm^2^, Waters) at a flow rate of 45 μL min^−1^ with an acetonitrile gradient (2% to 80% over 12 min). Mass spectra were acquired on an Orbitrap Fusion mass spectrometer (Thermo Fisher Scientific) in positive ion mode over a mass range of m/z 300–1500. The full MS resolution was set to 60,000, and MS2 resolution was set to 15,000. The total acquisition time for each LC-MS run was 15 min. To identify peptides, a spectral database was built using non-deuterated (MS1/MS2) data with BioPharm Finder (version 2.0). Deuteration levels were calculated using HDExaminer (version 2.4.1). A summary table of HDX-MS experimental parameters is provided (Supplementary Data 4).

### Co-immunoprecipitation (Co-IP)

To assess STING ubiquitination, HEK293T cells were co-transfected with Flag-STING (wild-type or mutant) and HA-Ub (wild-type or mutant) plasmids at a 1:1 ratio using PolyJet transfection reagent (SignaGen) according to the manufacturer’s instructions. After 24 h of transfection, cells were pretreated with MG-132 (5 μM) for 1 h, followed by treatment with Omaveloxolone (0.5 μM or 1 μM) for an additional 24 h. For protein-protein interaction studies, HEK293T cells were transfected with Myc-STING and Flag-HUWE1 plasmids under identical conditions and subsequently treated with 350 nM Omaveloxolone for 6 h. Cells were lysed in IP lysis buffer (Beyotime, P0013) supplemented with protease inhibitor. Lysates were cleared by centrifugation, and protein concentrations were quantified using BCA assay kit. For immunoprecipitation, lysates were incubated with anti-Flag magnetic beads (Selleck, B26102) overnight at 4 °C with gentle rotation. For endogenous STING ubiquitination analysis, THP-1 cells were pretreated with 2 μM PR171 for 1 h, followed by treatment with Omaveloxolone (1 μM) or DMSO for 8 h. A total of 2 × 10^7^ cells were collected and boiled in PBS containing 1% SDS supplemented with 5 mM N-ethylmaleimide (NEM). The lysates were diluted 10-fold with IP lysis buffer (Beyotime, P0013) supplemented with protease inhibitor. After centrifugation for clarification, protein concentrations were determined using a BCA protein assay kit. For endogenous immunoprecipitation, protein A/G magnetic beads (Selleck, B23201) were pre-incubated with STING antibody (CST, 13647) at a dilution of 1:50 for 1.5 h at room temperature. The pre-coupled beads were then incubated with cell lysates overnight at 4 °C with gentle rotation. After extensive washes, bound proteins were eluted under denaturing conditions by boiling in 2× SDS loading buffer and analyzed by western blot.

### NanoBiT complementation assay

The interaction between STING and HUWE1 was analyzed using the NanoBiT protein complementation system. Full-length STING and its truncations (TM, LBD, CTT) were cloned into the LgBiT TK-neo Flexi vector (Promega) to generate N-terminal LgBiT-tagged constructs. Similarly, HUWE1 truncations (ARLD1, ARLD2, ARLD3, ARLD4) were cloned into the pFN35K SmBiT TK-neo Flexi vector (Promega) to create N-terminal SmBiT-tagged constructs. HEK293T cells were transfected with full-length STING-LgBiT and ARLD1/2/3/4-SmBiT, or ARLD2-SmBiT and TM/LBD/CTT-LgBiT using PolyJet transfection reagent. After 24 h transfection, cells were treated with Omaveloxolone (400 nM or 800 nM) or DMSO for 24 h. Protein-protein interactions were assessed using the Nano-Glo Luciferase Assay System (Promega, N1110) according to the manufacturer’s protocol. Luminescence was measured using a PerkinElmer EnVision multifunctional microplate reader.

### Microscale thermophoresis (MST) assay

MST assays were performed using Monolith NT.115 instrument (Nano Temper). HEK293T cells were collected 24 h after transfection with EGFP-tagged HUWE1 and lysed in IP lysis buffer (Beyotime, P0013) supplemented with protease inhibitor (Bimake, B14001) and phosphatase inhibitor (Bimake, B15001). The supernatant of cell lysates was used as the fluorescent protein source. Omaveloxolone was serially diluted in the lysate, followed by incubation at room temperature for 15 min. Samples were loaded into standard MST capillaries (NanoTemper, MO-K022-SP). Measurements were performed at 40% excitation power using the MO Control software. The cold and hot fluorescence signals were recorded at −1–0 s and 4–5 s, respectively. Binding curves and *K*_d_ values were fitted and calculated using the MO Affinity Analysis software.

### SEAP and luciferase reporter assay

For SEAP reporter assay, THP-1-Blue-ISG-derived macrophages were plated into 96-well plates at a density of 5 × 10^4^ cells/well. Cells were treated with serially diluted compounds for 12 h and subsequently stimulated with 5 μM cGAMP for 6 h. Secreted embryonic alkaline phosphatase (SEAP) activity was quantified using the QUANTI-Blue kit (InvivoGen, rep-qbs2) according to the manufacturer’s protocol, with absorbance measured at 650 nm using a Tecan Spark multifunctional microplate reader. For the luciferase reporter assay, RAW-Lucia ISG cells were seeded in 96-well plates at a density of 1 × 10^4^ cells/well and cultured overnight. Cells were treated with serially diluted compounds for 12 h and subsequently stimulated with 10 μM cGAMP for 6 h. Luciferase activity was assessed using the QUANTI-Luc kit (InvivoGen, rep-qlc2) following the manufacturer’s protocol, with luminescence signals detected using a Tecan Spark multifunctional microplate reader.

### RNA sequencing

THP-1-derived macrophages were treated with Omaveloxolone (500 nM) or DMSO for 12 h. Where indicated, cells were subsequently stimulated with 5 μM cGAMP for an additional 6 h (three independent biological replicates per group). Total RNA was extracted using RNA isolater Total RNA Extraction Reagent (Vazyme, R401-01). Subsequent sample quality inspection, RNA sequencing, and differential gene expression analysis were completed by Majorbio Bio-Pharm Technology Co., Ltd. (Shanghai, China).

### Senescence-associated β-galactosidase (SA-β-Gal) staining

SA-β-Gal staining was performed using a commercial SA-β-Gal staining kit (Beyotime, C0602) following the manufacturer’s instructions. Briefly, cells were washed with PBS and fixed with the kit-provided fixation solution at room temperature for 15 min. After being washed by PBS for three times, cells were incubated with SA-β-Gal working solution at 37 °C. Staining images were acquired using a Zeiss LSM 900 confocal microscope.

### Animals

All animal studies were conducted in compliance with the guidelines approved by the Institutional Animal Care and Use Committees (IACUC) of Shanghai Institute of Materia Medica, Chinese Academy of Sciences (IACUC Issue NO. 2025-04-ZMY-12). Wild-type (WT) C57BL/6 mice were purchased from Beijing Huafukang Biotechnology Co., Ltd. *Trex1*^+/‒^ C57BL/6 mice were kindly provided by Prof. Yichuan Xiao’s laboratory at Shanghai Institute of Nutrition and Health, Chinese Academy of Sciences. *Trex1*^‒/‒^ C57BL/6 mice were generated by further mating the male and female *Trex1*^+/‒^ mice. For compound administration, a solution containing 5% DMSO, 40% PEG300, and 5% Tween-80 in double-distilled water was used. Mice were housed in a specific-pathogen-free (SPF) facility with free access to pellet food and water. The housing environment was maintained at 21 ± 2 °C with 50% ± 10% relative humidity under a 12-h light/dark cycle in plastic cages. Mice were euthanized by CO_2_ asphyxiation.

### Trex1^‒/‒^ mouse model study

To assess the therapeutic efficacy of Omaveloxolone in *Trex1*^‒/‒^ mouse model, *Trex1*^‒/‒^ C57BL/6 mice of 4-7 weeks old were administered with Omaveloxolone (10 mg kg^‒1^) or vehicle control via intraperitoneal injection for 21 days, with WT mice serving as controls (*n* = 5 mice/group). Following the treatment period, mice were euthanized, and tissues were collected for subsequent analysis by western blot and RT-qPCR. Sex-based analysis was not conducted, as sex was not incorporated as an experimental variable in the original study design.

### Aging mouse model study

Twenty-two-months-old male wild-type C57BL/6 mice were injected intraperitoneally with Omaveloxolone (10 mg kg^‒1^) once every two days or H-151 (10 mg kg^‒1^) daily for one month (*n* = 5 mice/group). Following a one-month treatment break, the same dosing regimen was administered for an additional month. The hanging grid test was performed at the end of experiment. Briefly, mice were placed on a wire grid, which was then inverted. The hanging time was measured from the moment of complete suspension until the mouse released its grip onto a padded surface. Mice were subsequently euthanized, and tissues were harvested for comprehensive analysis, including western blot, RT-qPCR, H&E staining, immunohistochemistry, and immunofluorescence analysis. H&E staining, immunohistochemistry and immunofluorescence analysis were completed by Servicebio Technology Co., Ltd. (Wuhan, China).

### Reporting summary

Further information on research design is available in the Nature Portfolio Reporting Summary linked to this article.

## Supplementary information

Supplementary Information

Description of Additional Supplementary Files

Supplementary Data 1

Supplementary Data 2

Supplementary Data 3

Supplementary Data 4

Reporting Summary

Transparent Peer Review file

## Source data

Source Data

## Data Availability

All data are available in the main text or the supplementary materials. The RNA-seq data generated in this study have been deposited in the Genome Sequence Archive (GSA) database under accession code HRA015016. The proteomic data generated in this study have been deposited in a ProteomeXchange partner repository under accession code PXD071456. Source data are provided with this paper.
