# Supplementary material for: Omaveloxolone functions as a molecular crowbar-like degrader of STING to alleviate aging-related inflammation
Source: Nat Commun. 2026 Sep 17;17:11202. doi: 10.1038/s41467-026-77760-1 (PMC13719992; doi:10.1038/s41467-026-77760-1)
Supplement: Supplementary file 1 [file 41467_2026_77760_MOESM1_ESM.pdf]

Supporting Information for:

Omaveloxolone functions as a molecular crowbar-like degrader of STING to alleviate aging-related inflammation

Authors: Jingyi Zhou, Xinyi Ma, Bing Jiang, Zisheng Fan, Penghua Li, Qi Dai, Yajie Wang, Min Wu, Qibang Sui, Ning Ma, Ruirui Yang, Haiwei Shen, Jingyi Meng, Manlin Huang, Zhiming Ge, Jiusheng Lin, Dan Teng, Mingyue Zheng, Sulin Zhang

Table of contents

Supplementary Figures 3

Supplementary Fig. 1 Evaluation of STING-downregulating activity or cytotoxicity of the indicated compounds. 3

Supplementary Fig. 2 Binding mode analysis of Omaveloxolone with STING protein. 5

Supplementary Fig. 3 Omaveloxolone destabilizes STING and disease-associated STING variants. 7

Supplementary Fig. 4 STING degradation activity, binding activity and destabilization activity of Omaveloxolone analogs. 8

Supplementary Fig. 5 Upward-shifted binding poses of inactive Omaveloxolone analogs in the STING LBD. 10

Supplementary Fig. 6 Omaveloxolone destabilizes STING, facilitates HUWE1-mediated recognition, and induces K27-linked polyubiquitination of STING. 11

Supplementary Fig. 7 Omaveloxolone inhibits STING pathway downstream gene expression. 12

Supplementary Fig. 8 Omaveloxolone alleviates cellular senescence and senescence-related inflammation in a STING-dependent manner. 13

Supplementary Fig. 9 Body weight trajectories during chronic OMA treatment in aged mice. 15

Supplementary Fig. 10 Flow cytometry gating strategy. 15

Supplementary Fig. 11 Western blot analysis of STING protein levels in WT and STING-KO THP-1/RAW 264.7 cells. 15

Supplementary Fig. 12 Chemical structures of all tested compounds in Table 1. 16

Supplementary Methods Synthetic details and spectra 17

Supplementary Fig. 13 The synthetic route of LPH-A33, LPH-A40, LPH-A43 and LPH-A134. 17

Supplementary Fig. 14 The synthetic route of LPH-A107. 30

Supplementary Fig. 15 The synthetic route of D1-1, D5-1 and D6-2. 34

Supplementary Fig. 16 The synthetic route of D6-1. 41

Supplementary Fig. 17 The synthetic route of D17. 47

Supplementary Figures

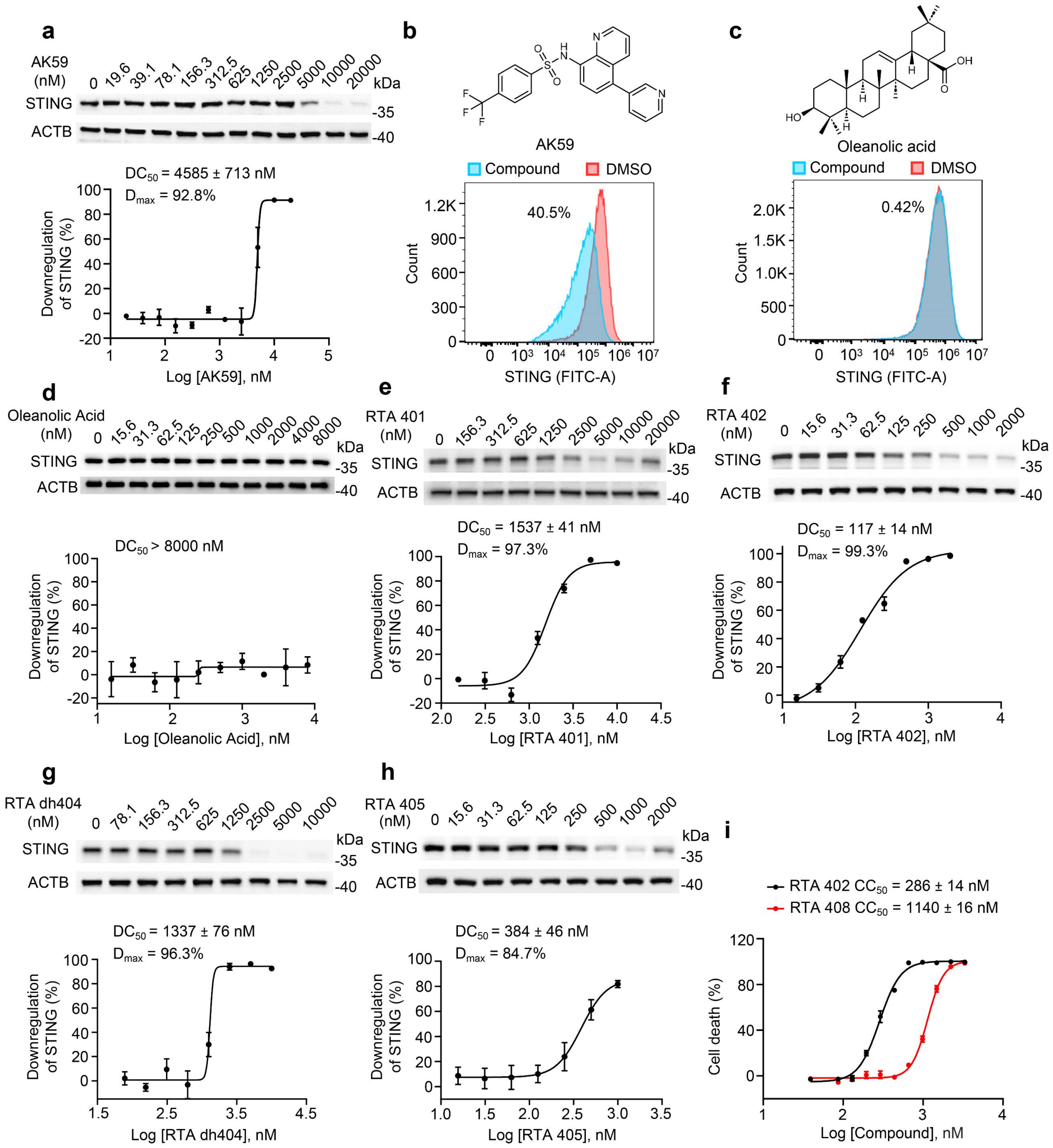

Supplementary Fig. 1 Evaluation of STING-downregulating activity or cytotoxicity of the indicated compounds.

a, Western blot analysis of STING protein levels in THP-1 cells treated with indicated concentrations of AK59 for 24 h. **b,c,** Chemical structures of AK59 and Oleanolic acid, and their ability to downregulate STING at 2 μM (12 h treatment) in THP-1$^{EGFP\text{-}STING}$ cells, as analyzed by the flow cytometry-based screening. Gating strategy for these panels is shown in Supplementary Fig. 10. **d–h,** Western blot analysis of STING protein levels in THP-1 cells treated with the indicated concentrations of Oleanolic acid (**d**), RTA 401 (**e**), RTA 402 (**f**), RTA dh404 (**g**), or RTA 405 (**h**) for

24 h. **i,** Cytotoxicity of RTA 402 and RTA 408 in THP-1 cells after 24 h treatment. DC_{50} refers to half-maximal downregulation concentration; D_{max} refers to maximum downregulation efficacy; CC_{50} refers to half-maximal cytotoxic concentration. DC_{50} and CC_{50} values were determined by fitting the dose-response curves (variable slope) through nonlinear regression analysis using Graphpad Prism 8.0.1 (n = 3 independent experiments, mean ± SD). Source data are provided as a Source Data file.

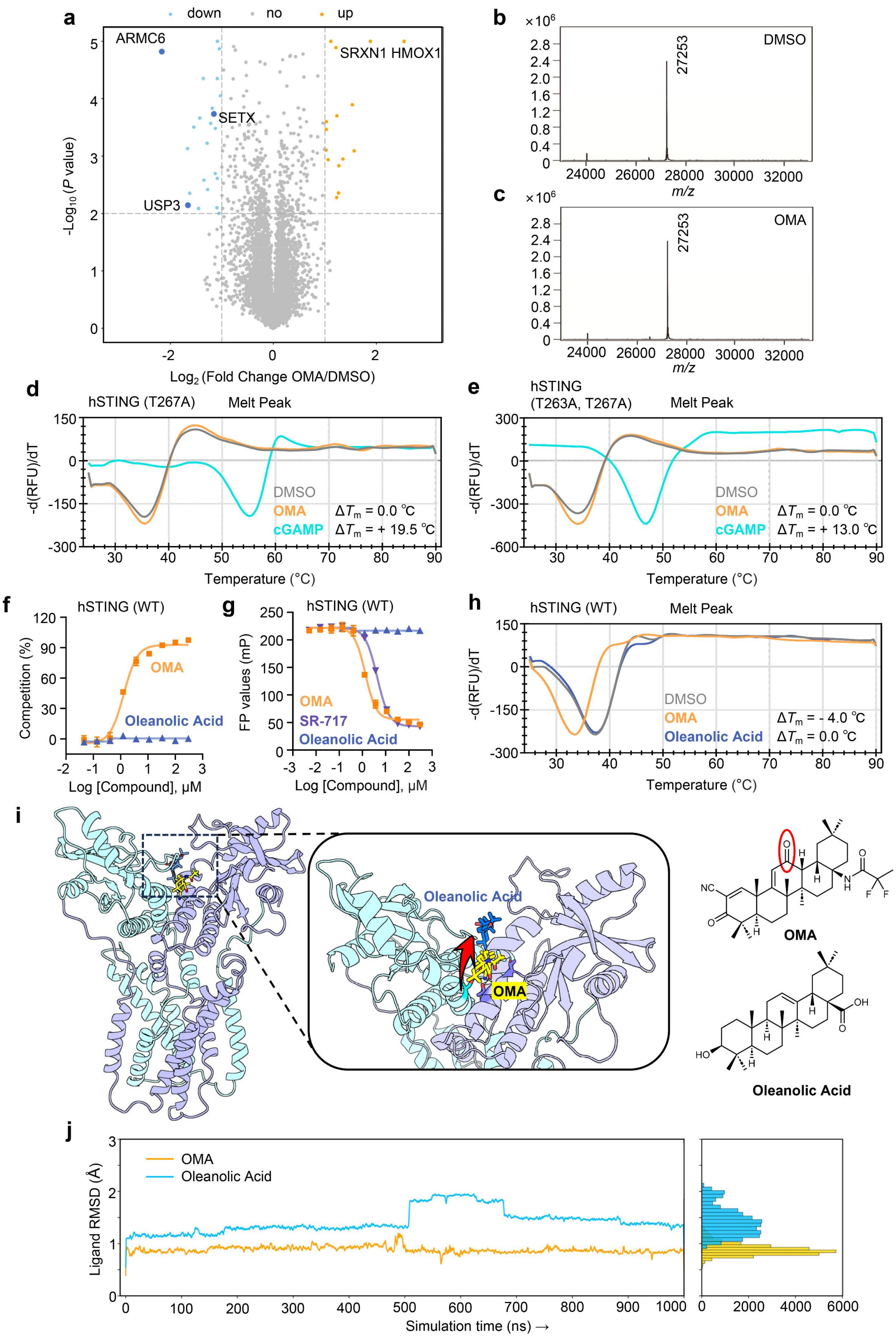

Supplementary Fig. 2 Binding mode analysis of Omaveloxolone with STING protein.

a, Proteomic analysis of STING-KO-THP-1 cells treated with 500 nM OMA for 12 h. The volcano plot displays differentially expressed proteins compared to the DMSO control. Statistical analysis was conducted using an unpaired two-sided Student's *t*-test with raw unadjusted *P* values. **b,c,** Mass

spectrometry analysis of $hSTING^{WT}$ protein incubated with DMSO (**b**) or OMA (**c**), showing no evidence of covalent modification. **d,e,** Effects of OMA (1 μM) and cGAMP (50 μM) on the melting temperature of $hSTING^{T267A}$ (2 μM) (**d**) and $hSTING^{T263A, T267A}$ (2 μM) (**e**) determined by PTS assay. **f,** Competitive binding of OMA and Oleanolic acid to $hSTING^{WT}$ measured by FP assay (n = 4 independent biological replicates, mean ± SD). **g,** FP values from competitive binding assays of OMA, SR-717, and Oleanolic acid to $hSTING^{WT}$ (n = 4 independent biological replicates, mean ± SD). **h,** Effects of OMA (1 μM) and Oleanolic acid (1 μM) on the melting temperature of $hSTING^{WT}$ (2 μM) determined by PTS assay. **i,** Superimposition of representative binding conformations from the stable phase of MD simulations for OMA and Oleanolic acid. Oleanolic acid, lacking the carbonyl group necessary for hydrogen bonding with T263 and T267, gradually dissociates from the STING binding pocket. **j,** RMSD distribution of OMA and Oleanolic acid during MD simulations. OMA denotes Omaveloxolone; ΔT_m refers to the shift of protein melting temperature. Source data are provided as a Source Data file.

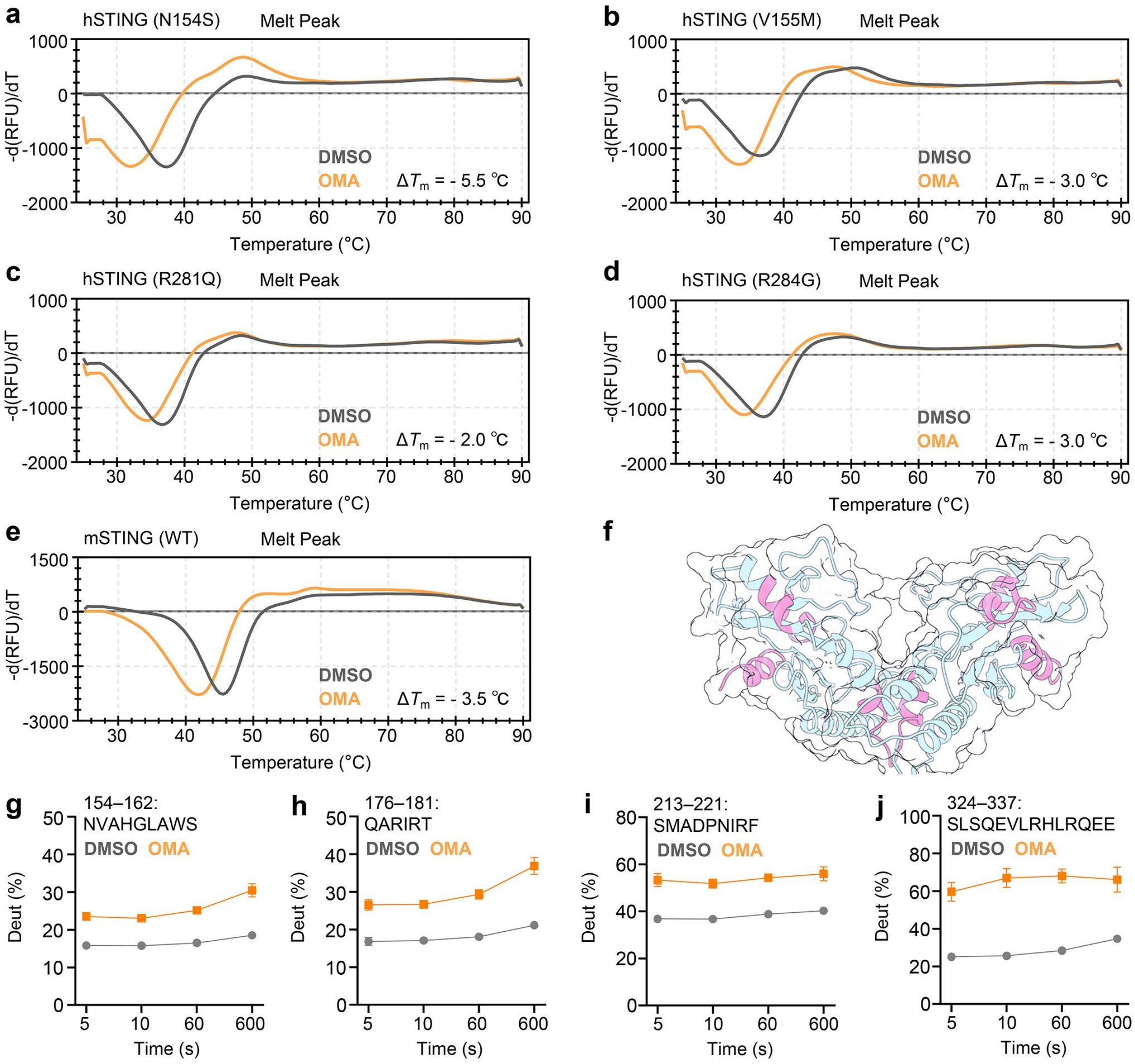

Supplementary Fig. 3 Omaveloxolone destabilizes STING and disease-associated STING variants.

a–e, Effects of OMA (1 µM) on the melting temperature of STING proteins, including hSTING variants N154S (2 µM) (**a**), V155M (2 µM) (**b**), R281Q (2 µM) (**c**), R284G (2 µM) (**d**), and mSTING (2 µM) (**e**), as determined by PTS assay. **f,** Peptide segments in the STING LBD showed significantly increased deuterium uptake upon OMA treatment. These segments are mapped onto the STING LBD dimer structure and highlighted in magenta, with the remaining structure shown in light cyan. **g–j,** Deuterium uptake plots for the peptide regions highlighted in **f** (n = 4 technical replicates; data are presented as mean ± SD). OMA denotes Omaveloxolone, ΔT_m refers to the shift of protein melting temperature. Source data are provided as a Source Data file.

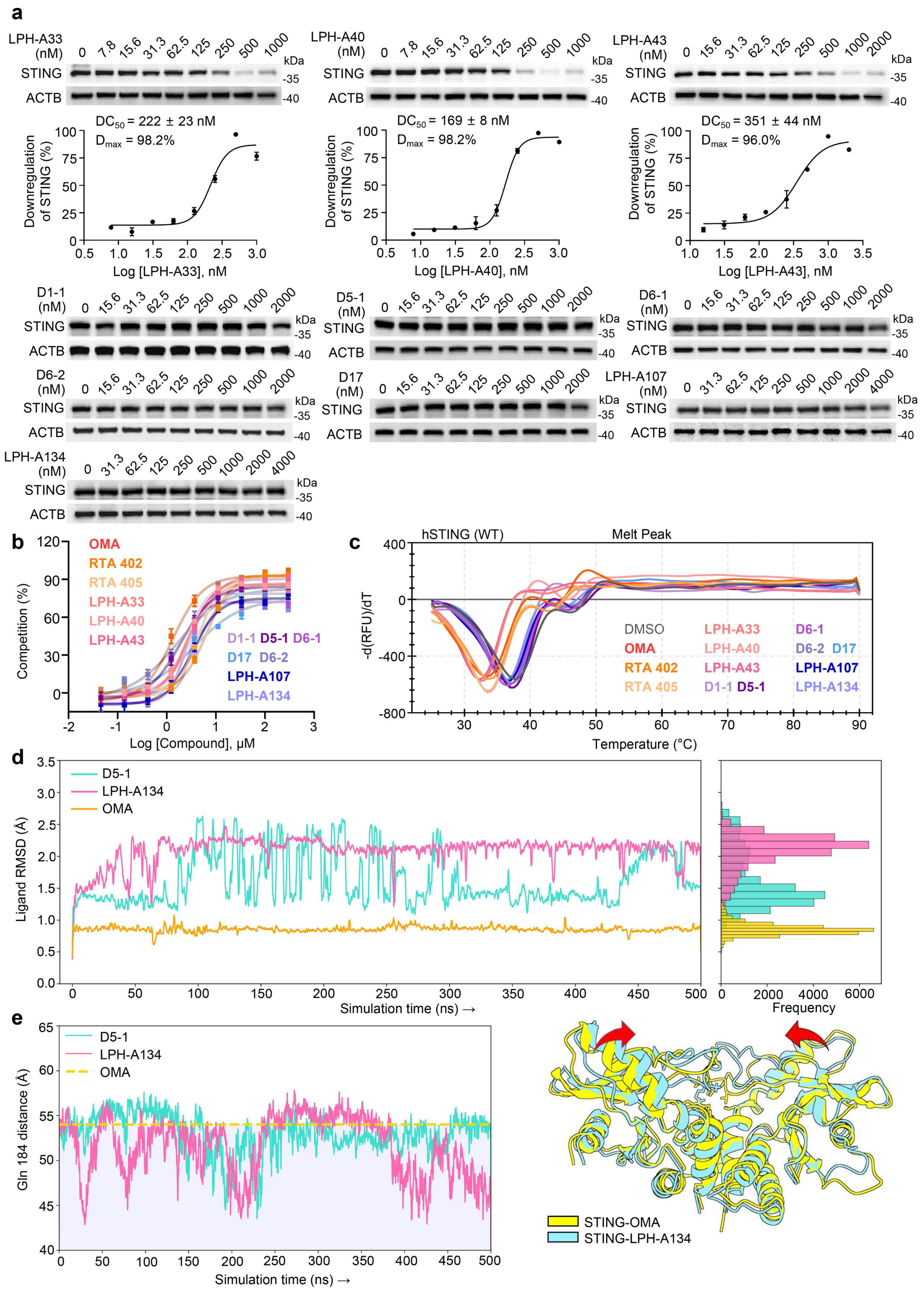

Supplementary Fig. 4 STING degradation activity, binding activity and destabilization activity of Omaveloxolone analogs.

a, Western blot analysis of STING protein levels in THP-1 cells treated with the indicated concentrations of Omaveloxolone analogs for 24 h. Data are representative of three independent

experiments with similar results. **b,** Competitive binding of Omaveloxolone analogs to $hSTING^{WT}$ measured by FP assay (n = 4 independent biological replicates, mean ± SD). **c,** Effects of Omaveloxolone analogs (1 μM) on the melting temperature of $hSTING^{WT}$ (2 μM) determined by PTS assay. **d,** RMSD analysis of representative non-degradative compounds D5-1 and LPH-A134 during MD simulations. Compared to OMA, both compounds gradually dissociate from the initial binding site, indicating their inability to stably anchor to the critical bottom region of the STING LBD. **e,** LBD opening analysis shows that, unlike the OMA-bound system, D5-1 and LPH-A134 fail to maintain an open LBD conformation and instead exhibit a marked tendency toward structural closure. OMA denotes Omaveloxolone; DC_{50} refers to half-maximal downregulation concentration; D_{max} refers to maximum downregulation efficacy. DC_{50} values were determined by fitting a dose-response curve (variable slope) generated from quantitative data through nonlinear regression analysis using Graphpad Prism 8.0.1 (n = 3 independent experiments, mean ± SD). Source data are provided as a Source Data file.

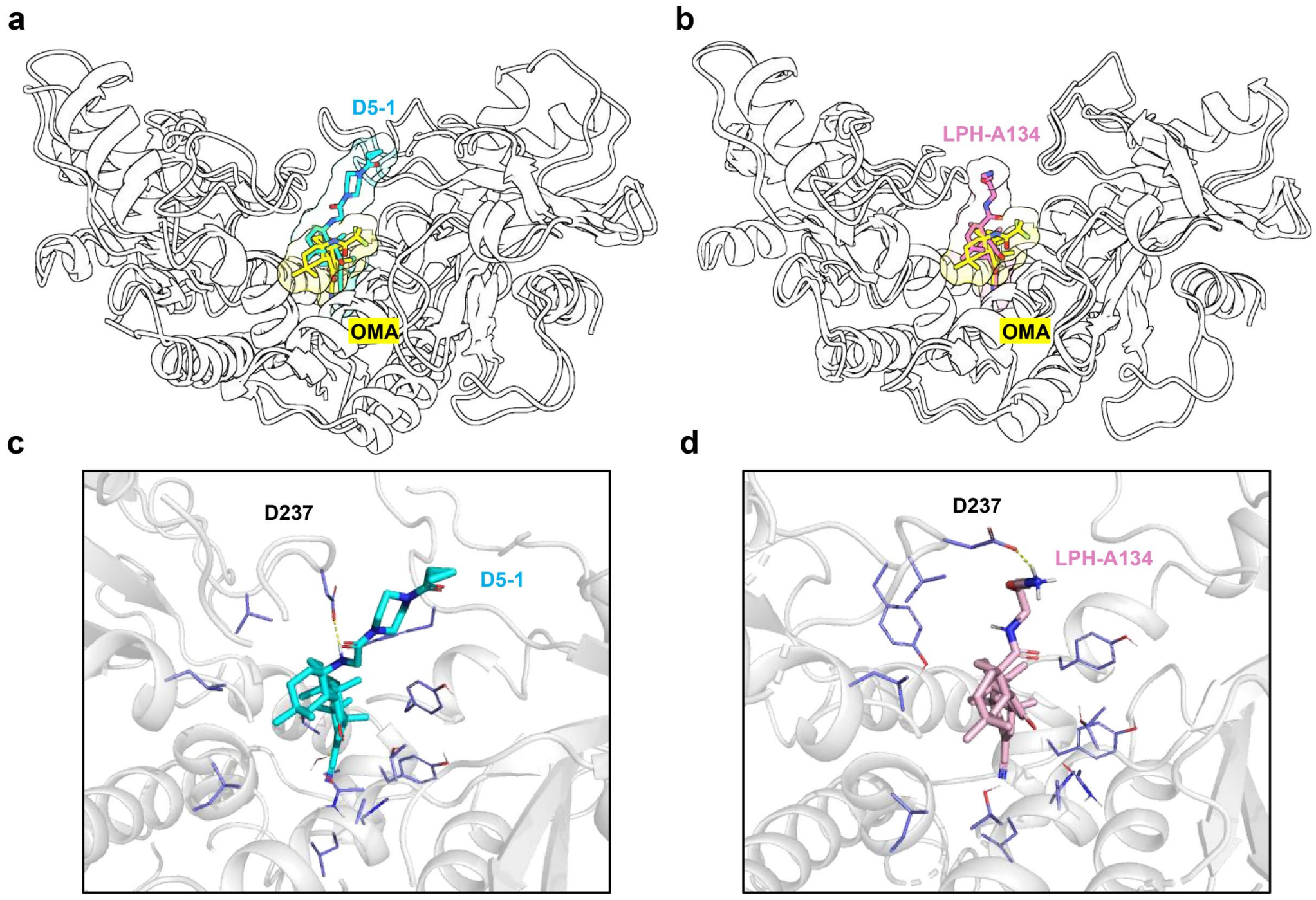

Supplementary Fig. 5 Upward-shifted binding poses of inactive Omaveloxolone analogs in the STING LBD.

a, Superposition of representative ligand poses from the stable MD simulation stage of the D5-1-STING complex (blue) and the OMA-STING complex (yellow). **b,** Superposition of representative ligand poses from the stable MD simulation stage of the LPH-A134-STING complex (magenta) and the OMA-STING complex (yellow). **c,d,** Binding mode analysis of D5-1 (**c**) and LPH-A134 (**d**) in the STING LBD. OMA denotes Omaveloxolone.

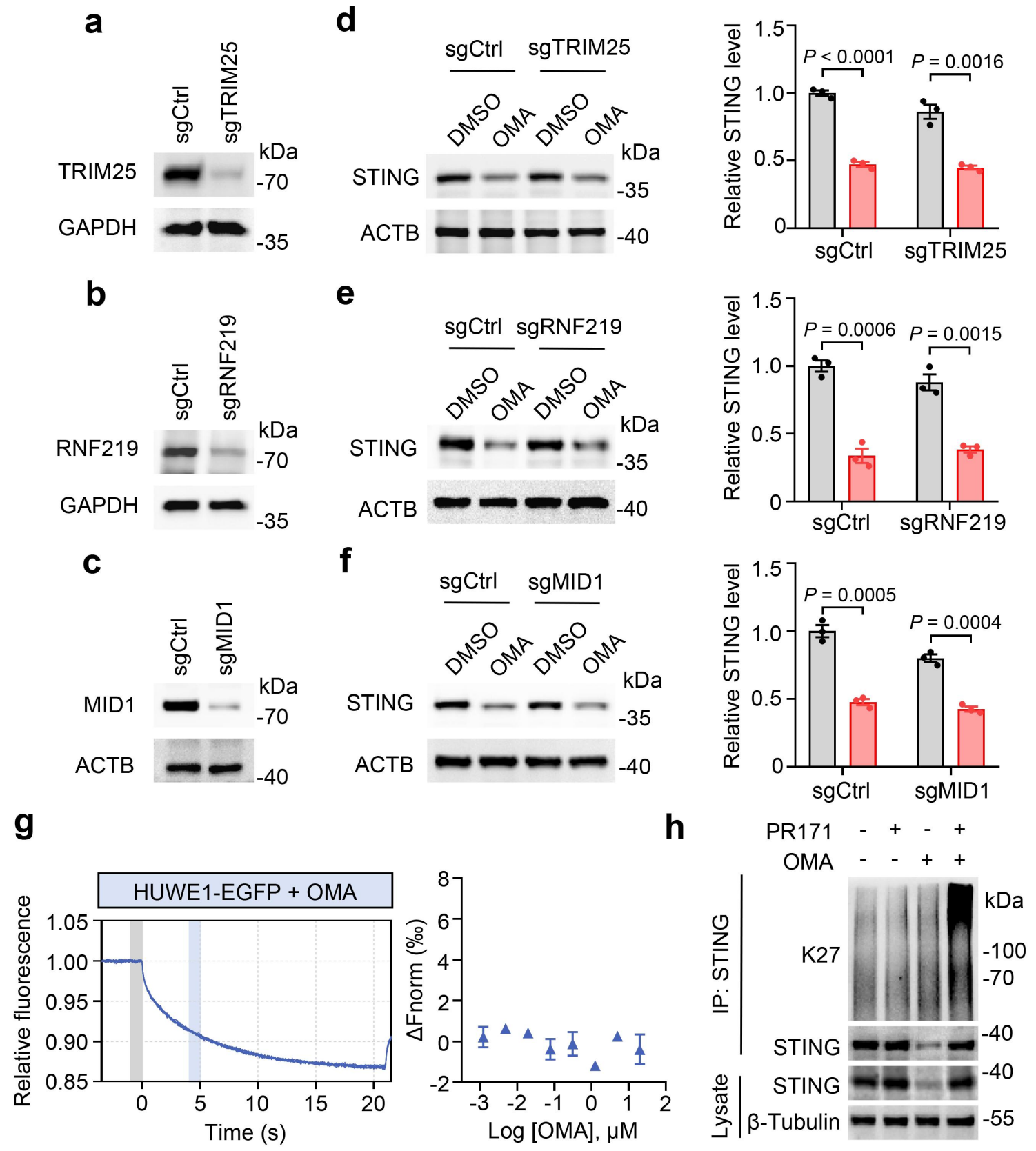

Supplementary Fig. 6 Omaveloxolone destabilizes STING, facilitates HUWE1-mediated recognition, and induces K27-linked polyubiquitination of STING.

a–c, Western blot analysis of TRIM25 (**a**), RNF219 (**b**), and MID1 (**c**) protein levels in THP-1 cells transduced with control sgRNA or gene-specific sgRNAs. **d–f,** Western blot analysis of the STING-degradation effect of OMA in THP-1 cells transduced with control sgRNA or gene-specific sgRNAs targeting *TRIM25* (**d**), *RNF219* (**e**), or *MID1* (**f**). Quantitative analysis of STING protein levels is presented in the right panel (n = 3 independent experiments, mean ± SEM). **g,** MST analysis reveals no direct binding between OMA and HUWE1. Representative MST traces (left panel) and dose-response curve (right panel) reveal no detectable binding (n = 3 independent biological replicates, mean ± SEM). **h,** OMA induces K27-linked polyubiquitination of endogenous STING. THP-1 cells were pretreated with PR171 (2 μM) for 1 h followed by OMA (1 μM) for 8 h. Endogenous STING was immunoprecipitated and probed with K27 linkage-specific ubiquitin antibodies. Lysate samples were run on separate gels with β-Tubulin as loading control. Data are representative of three independent experiments with similar results. OMA denotes Omaveloxolone. P values were calculated using two-sided unpaired Student's t-tests. Source data are provided as a Source Data file.

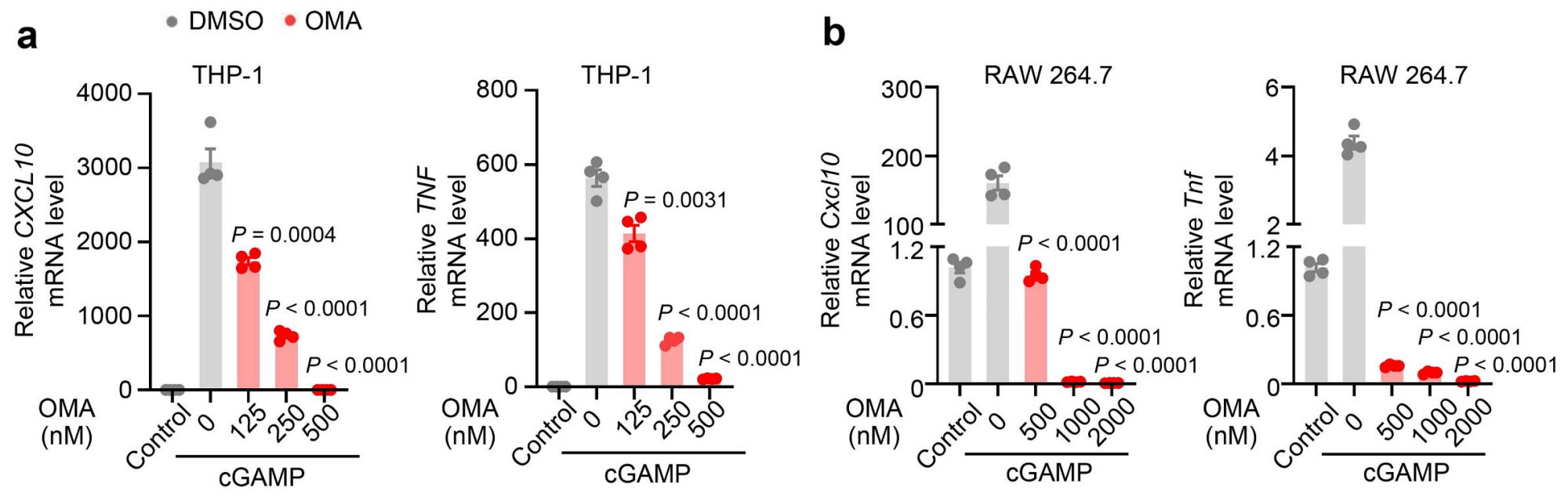

Supplementary Fig. 7 Omaveloxolone inhibits STING pathway downstream gene expression. **a,b,** RT-qPCR analysis of *CXCL10* and *TNF* mRNA levels in THP-1 (**a**) and RAW 264.7 (**b**) cells pretreated with indicated concentrations of OMA for 12 h, followed by cGAMP stimulation (5 μM for THP-1, 10 μM for RAW 264.7) for 6 h (n = 4, two technical replicates from two independent experiments, mean ± SEM). OMA denotes Omaveloxolone. P values were calculated using two-sided unpaired Student's t-tests. Source data are provided as a Source Data file.

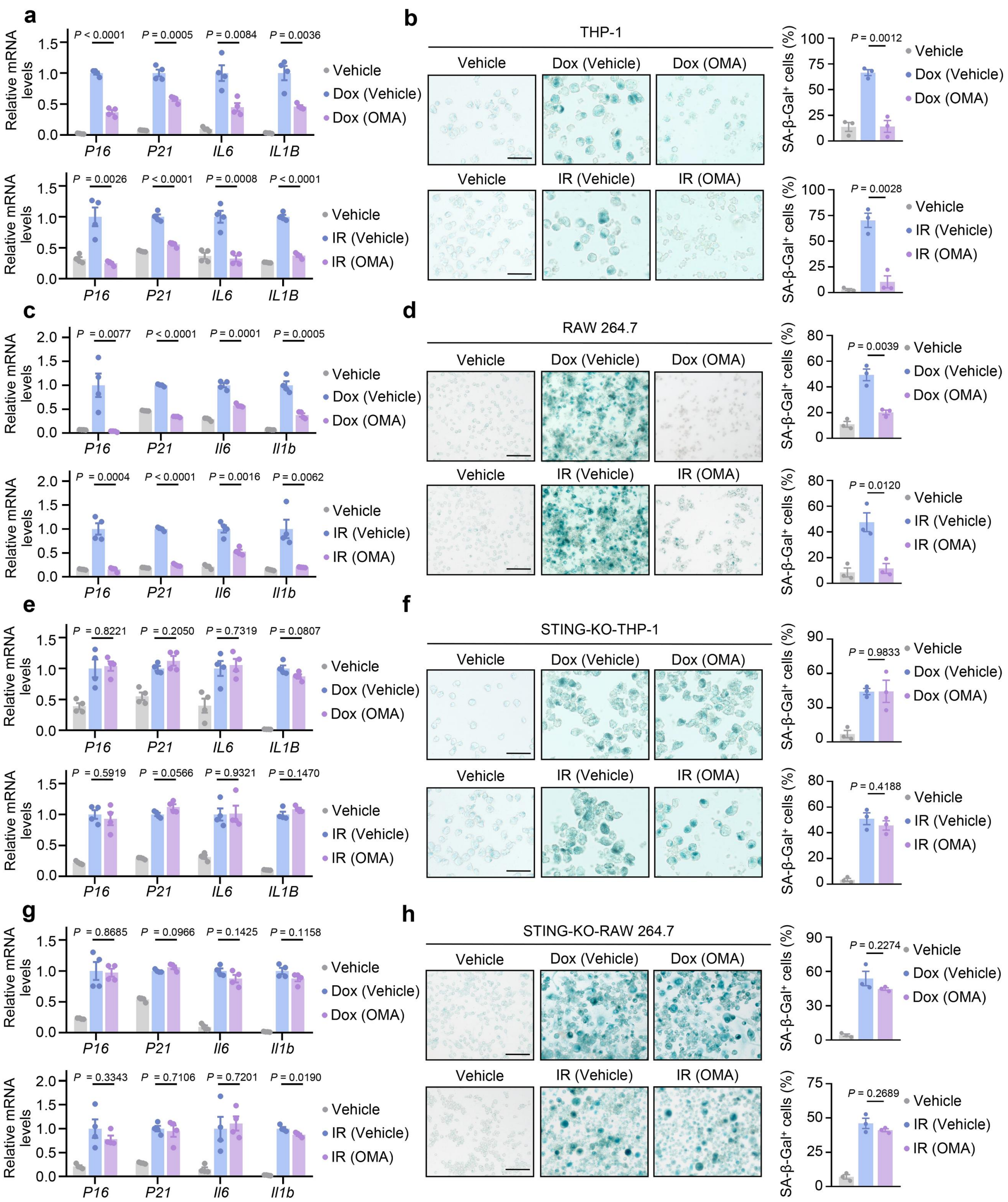

Supplementary Fig. 8 Omaveloxolone alleviates cellular senescence and senescence-related inflammation in a STING-dependent manner.

a,b, THP-1 cells were treated with 200 nM Dox for 24 h followed by 4 days of culture, or irradiated with 7.5 Gy X-ray and cultured for 7-9 days, to induce cellular senescence. Cells were then treated with 500 nM OMA for 24 h. The mRNA levels of senescence markers (*P16*, *P21*) and pro-inflammatory SASP factors (*IL6*, *IL1B*) were measured by RT-qPCR ($n = 4$, two technical replicates from two independent experiments, mean ± SEM) (**a**). Cellular senescence was assessed by SA-β-Gal staining; representative images and quantifications are shown (scale bar, 50 μm; n = 3

independent biological replicates, mean ± SEM) (**b**). **c,d,** RAW 264.7 cells were treated with 200 nM Dox for 24 h followed by 2 days of culture, or irradiated with 6 Gy X-ray and cultured for 4-6 days, to induce cellular senescence. Cells were then treated with 1 μM OMA for 24 h. The mRNA levels of *P16*, *P21*, *Il6*, and *Il1b* were measured by RT-qPCR ($n = 4$, two technical replicates from two independent experiments, mean ± SEM) (**c**). Representative SA-β-Gal staining images and quantifications are presented (scale bar, 100 μm; $n = 3$ independent biological replicates, mean ± SEM) (**d**). **e,f,** STING-KO-THP-1 cells were subjected to senescence induction as in **a,b**, followed by 500 nM OMA treatment for 24 h. The mRNA levels of *P16*, *P21*, *IL6*, and *IL1B* were measured by RT-qPCR ($n = 4$, two technical replicates from two independent experiments, mean ± SEM) (**e**). Representative SA-β-Gal staining images and quantifications are presented (scale bar, 50 μm; $n = 3$ independent biological replicates, mean ± SEM) (**f**). **g,h**, STING-KO-RAW 264.7 cells were subjected to senescence induction as in **c,d**, followed by 1 μM OMA treatment for 24 h. The mRNA levels of *P16*, *P21*, *Il6*, and *Il1b* were measured by RT-qPCR ($n = 4$, two technical replicates from two independent experiments, mean ± SEM) (**g**). Representative SA-β-Gal staining images and quantifications are presented (scale bar, 100 μm; $n = 3$ independent biological replicates, mean ± SEM) (**h**). OMA denotes Omaveloxolone. *P* values were calculated using two-sided unpaired Student's *t*-tests. Source data are provided as a Source Data file.

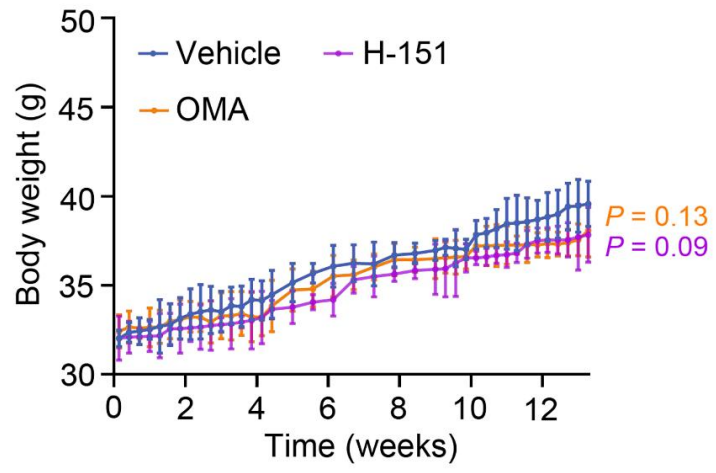

Supplementary Fig. 9 Body weight trajectories during chronic OMA treatment in aged mice. 22-month-old wild-type C57BL/6 mice were injected with OMA (i.p. 10 mg kg^{-1}) once every two days or H-151 (i.p. 10 mg kg^{-1}) daily for one month. The treatment regimen consisted of one month of dosing, followed by a one-month treatment-free interval, and then a second month of the same dosing schedule. Body weights were recorded every two days during treatment periods and every four days during the interval (n = 5 mice/group, mean ± SD). OMA denotes Omaveloxolone. P values were calculated using two-sided unpaired Student's t-tests. Source data are provided as a Source Data file.

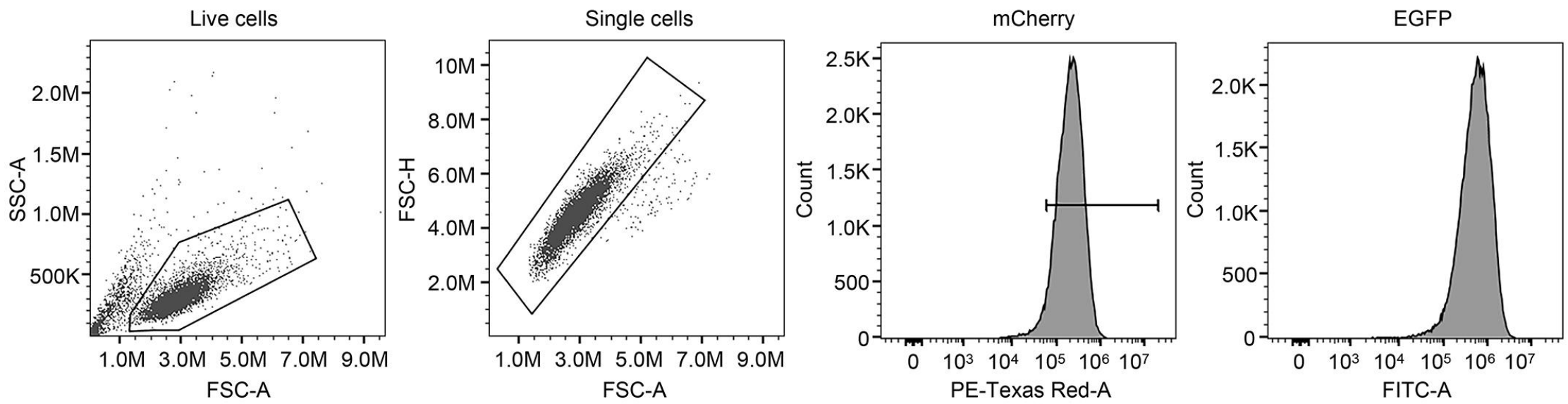

Supplementary Fig. 10 Flow cytometry gating strategy.

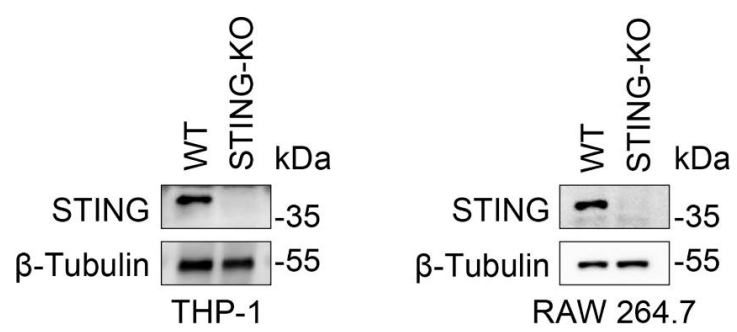

Supplementary Fig. 11 Western blot analysis of STING protein levels in WT and STING-KO THP-1/RAW 264.7 cells.

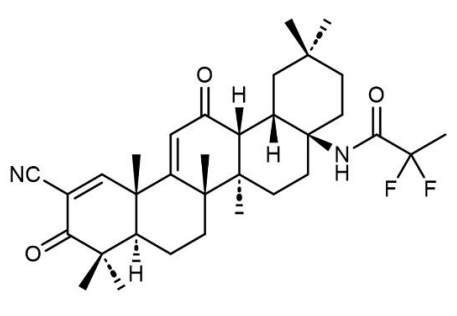

Omaveloxolone

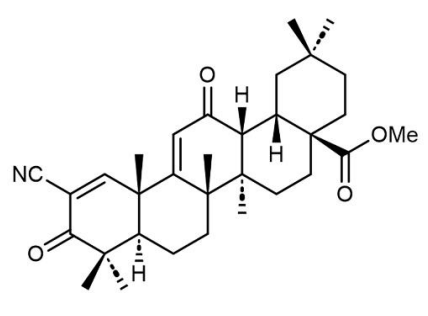

RTA 402

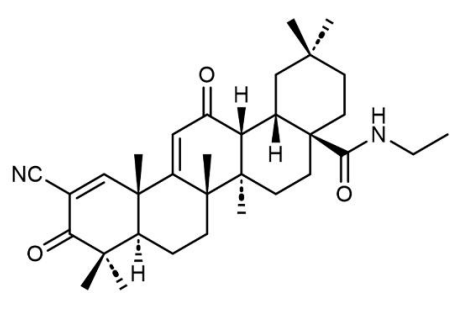

RTA 405

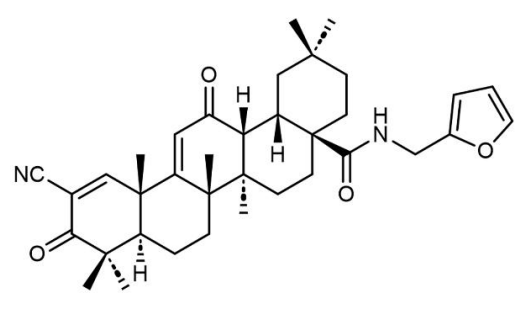

LPH-A33

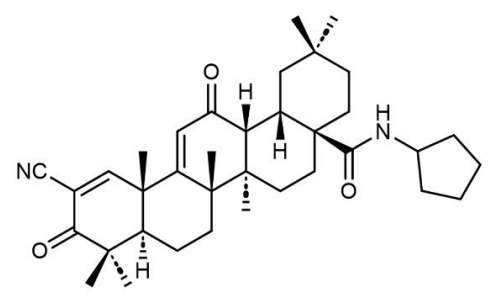

LPH-A40

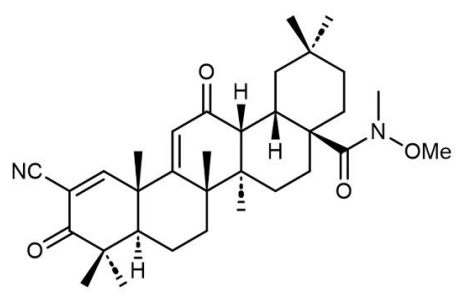

LPH-A43

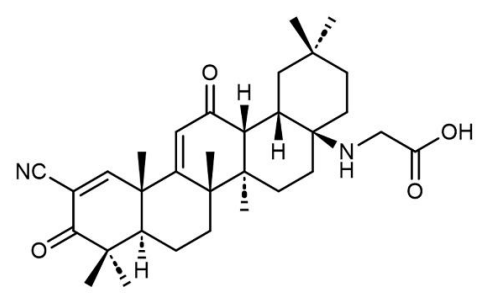

D1-1

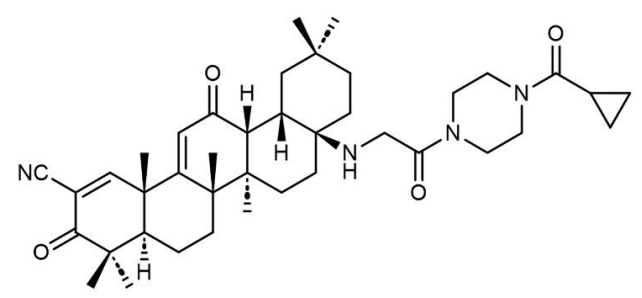

D5-1

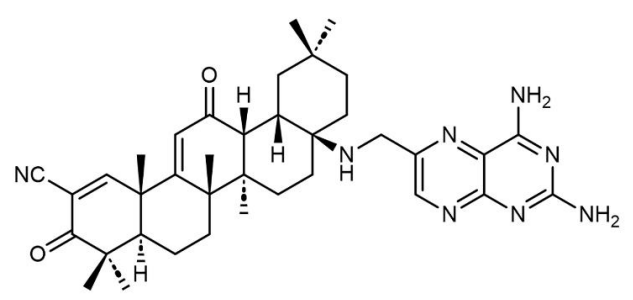

D6-1

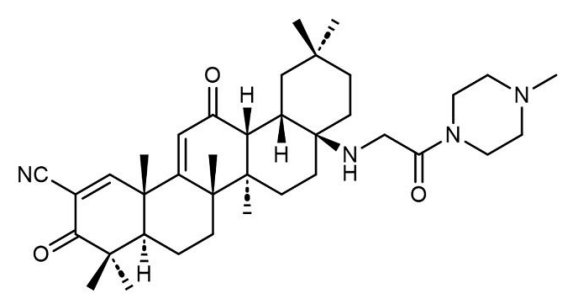

D6-2

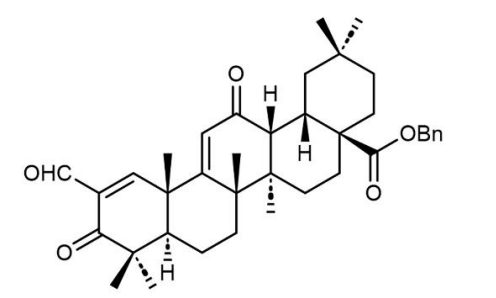

LPH-A107

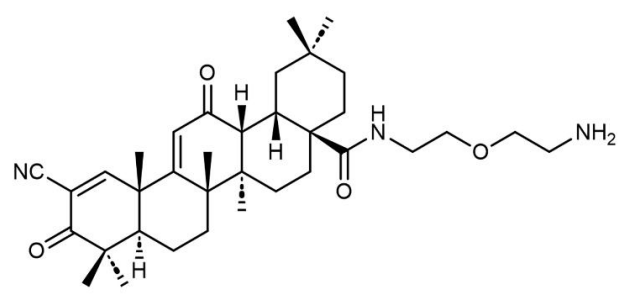

LPH-A134

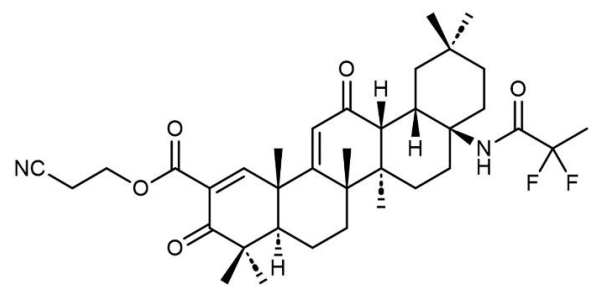

D17

Supplementary Fig. 12 Chemical structures of all tested compounds in Table 1.

Supplementary Methods Synthetic details and spectra

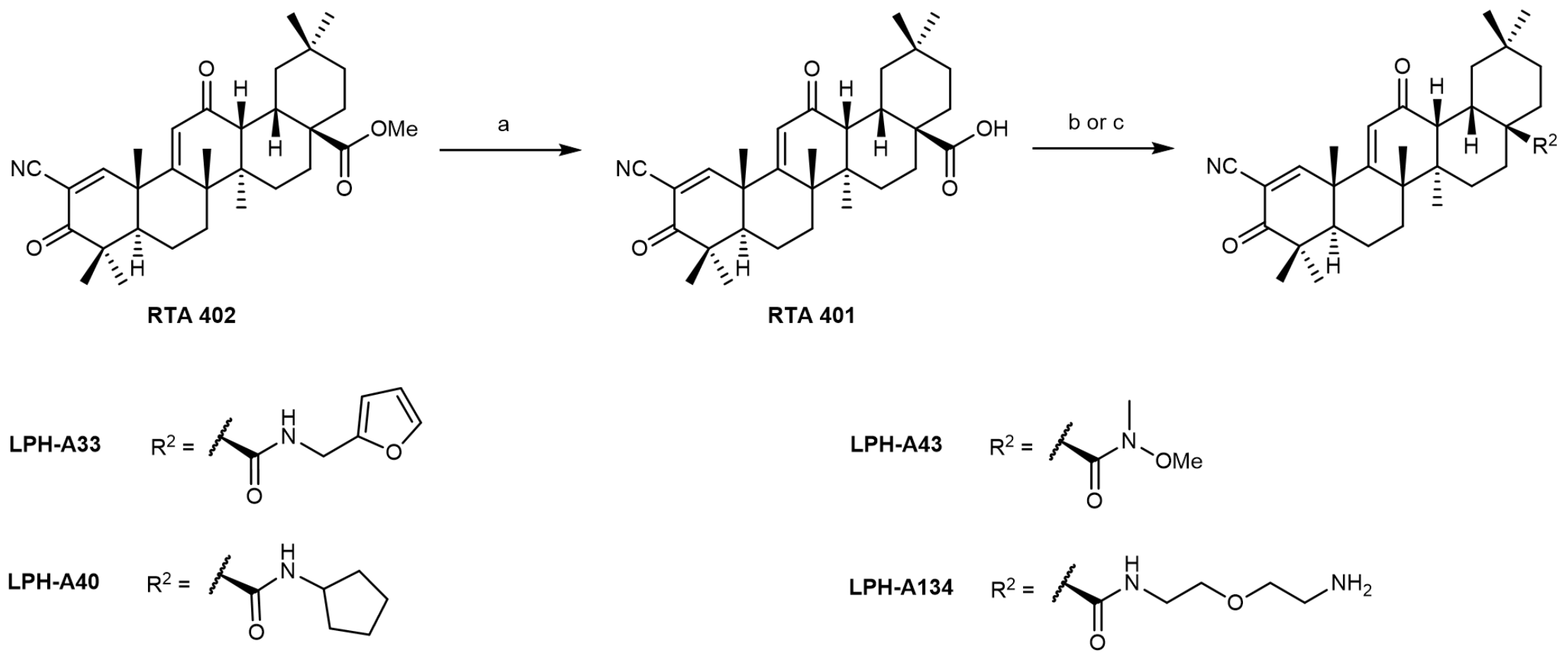

Supplementary Fig. 13 The synthetic route of LPH-A33, LPH-A40, LPH-A43 and LPH-A134. Reagent and conditions: (a) LiI, DMF, 155 °C; (b) RNH_2, PyBop, DIPEA, DMF; (c) RNH, $(COCl)_2$, Et_3N, CH_2Cl_2.

Method A: To a solution of **RTA 401** (1.00 mmol) in dry *N,N*-dimethylformamide (DMF, 0.1 M) were added 1H-benzotriazol-1-yloxytripyrrolidinophosphonium hexafluorophosphate (PyBOP, 1.50 mmol) and *N,N*-diisopropylethylamine (DIPEA, 3.00 mmol). The reaction mixture was stirred at room temperature for 15 min, followed by addition of amines (1.20 mmol). The reaction was stirred at room temperature until TLC analysis indicated completion, the mixture was diluted with EtOAc. The organic layer was washed successively with water, brine, and dried over Na_2SO_4. The solvent was removed under reduced pressure to afford the crude product. The final product was isolated by flash column chromatography.

Method B: RTA 401 (1.00 mmol) was dissolved in dry dichloromethane (2.0 mL), and oxalyl chloride (10.00 mmol) was added dropwise slowly under stirring. The mixture was stirred at room temperature for 1 h. After TLC analysis confirmed reaction completion, the solvent was removed under reduced pressure to afford a white solid. The white solid was redissolved in dry dichloromethane (0.1 M), and an amine substrate (2.00 mmol) and a base (2.00 mmol) were added under stirring. The reaction was allowed to proceed at room temperature overnight. Upon confirmation of reaction completion by TLC monitoring, the target compound was purified by flash

column chromatography.

The synthesis of LPH-A33

The title compound was synthesized with **Method A** from **RTA 401** (55.0 mg, 0.11 mmol) and Furfurylamine (12 μL, 0.13 mmol). **LPH-A33** was obtained as a white solid (51.0 mg, 80%). ^{1}H NMR (400 MHz, $CDCl_3$) δ 8.06 (s, 1H), 7.29–7.26 (m, 1H), 6.31–6.22 (m, 2H), 6.20 (d, J = 3.2 Hz, 1H), 5.96 (s, 1H), 4.43 (d, J = 5.5 Hz, 2H), 3.02 (d, J = 4.5 Hz, 1H), 2.90 (d, J = 13.3 Hz, 1H), 1.97 (td, J = 13.7, 3.8 Hz, 1H), 1.85–1.47 (m, 10H), 1.45 (s, 3H), 1.36–1.24 (m, 3H), 1.23 (s, 3H), 1.18 (s, 3H), 1.14 (s, 3H), 0.98 (s, 3H), 0.97 (s, 3H), 0.88 (s, 3H); ^{13}C NMR (101 MHz, $CDCl_3$) δ 199.16, 196.70, 176.97, 168.85, 166.01, 151.86, 142.06, 124.01, 114.60, 114.53, 110.55, 107.57, 49.49, 47.78, 46.64, 45.91, 45.10, 42.65, 42.21, 36.54, 36.14, 34.67, 34.04, 33.37, 31.94, 31.74, 30.71, 27.78, 27.07, 26.67, 24.72, 23.20, 23.14, 21.81, 21.65, 18.33. HRMS (ESI) m/z: $[M + H]^+$ calcd for $C_{36}H_{47}N_2O_4$ 571.3530, found 571.3535.

^{1}H NMR (400 MHz, CDCl$_3$) of LPH-A33

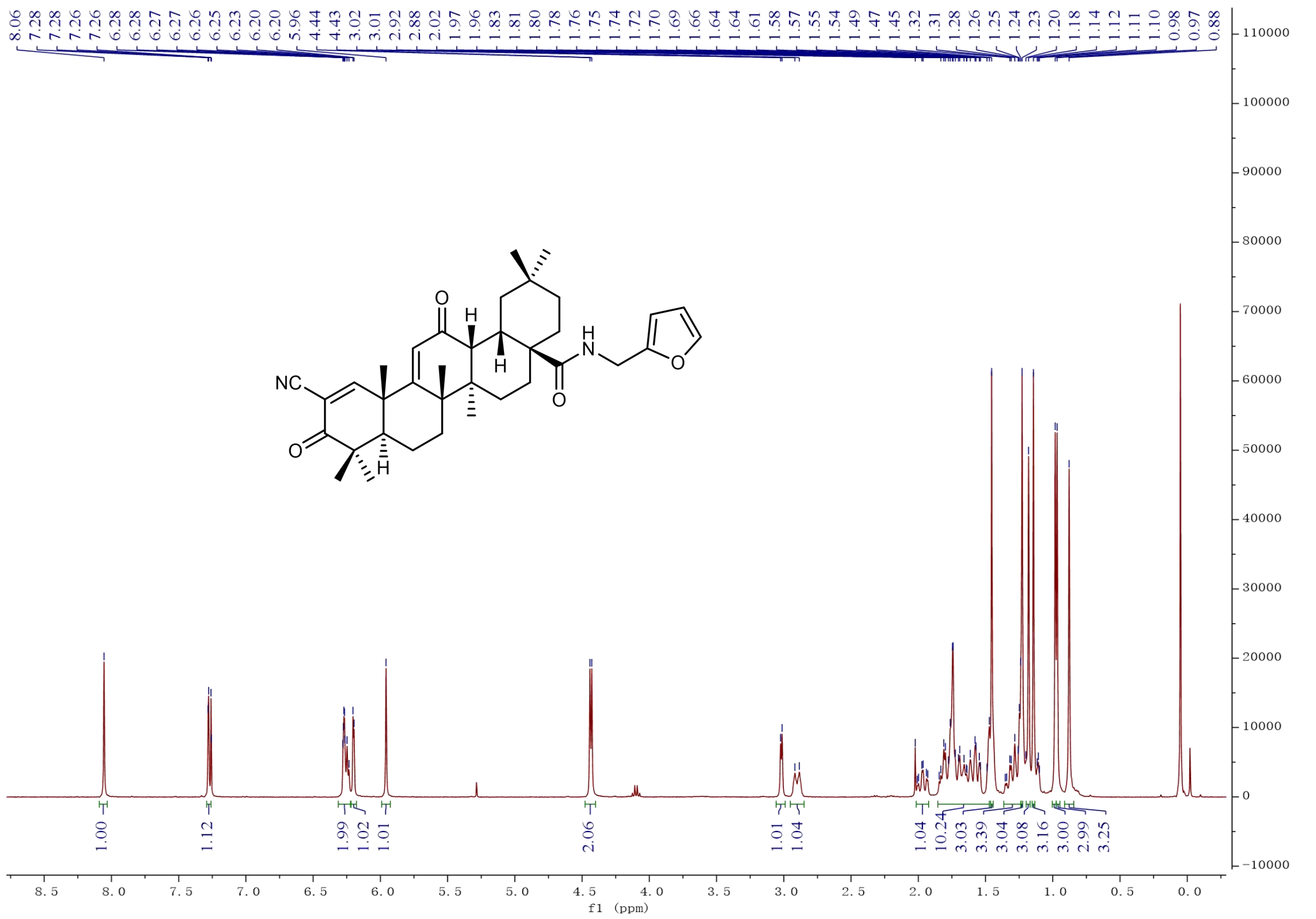

^{13}C NMR (101 MHz, CDCl$_3$) of LPH-A33

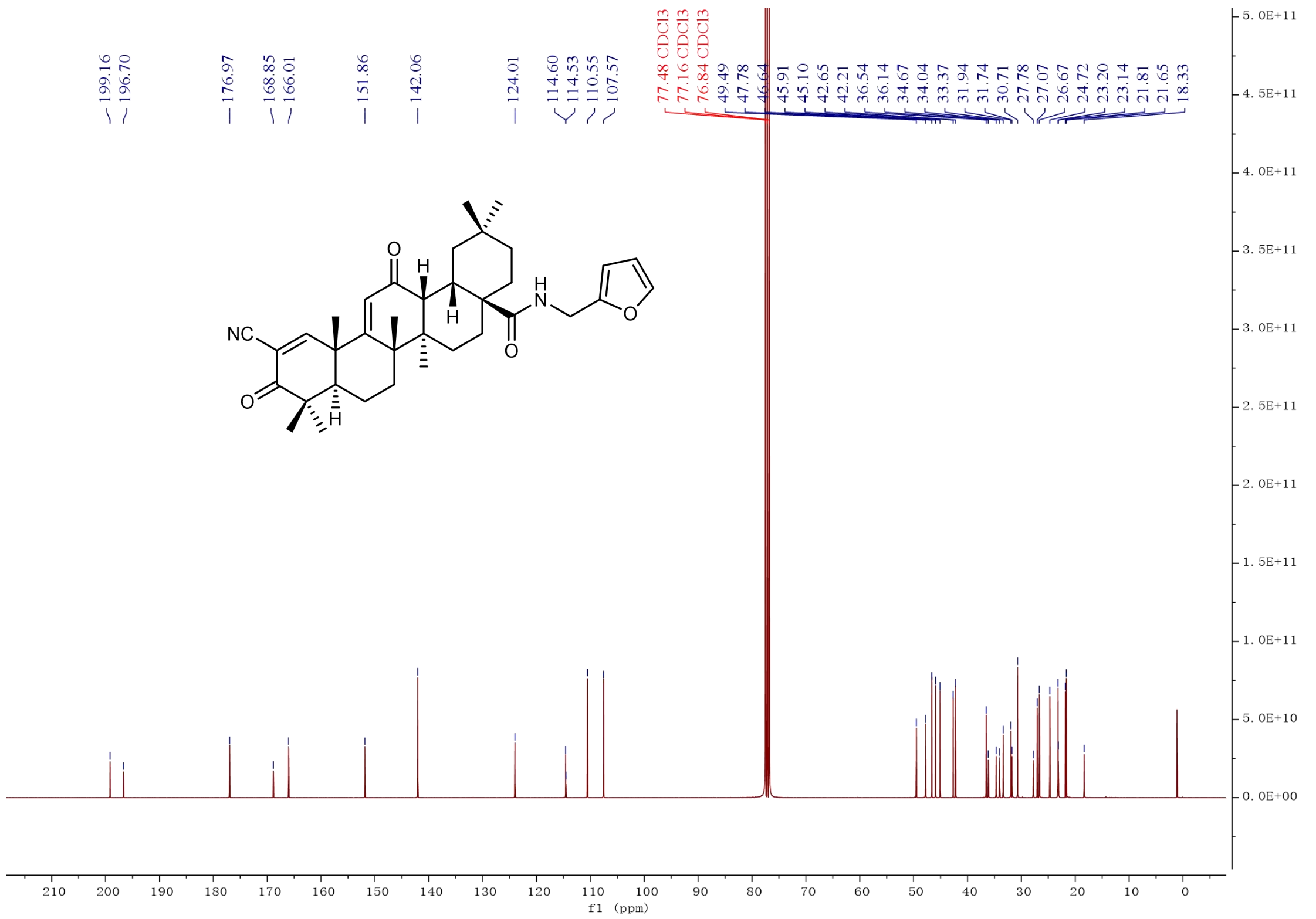

HPLC of LPH-A33

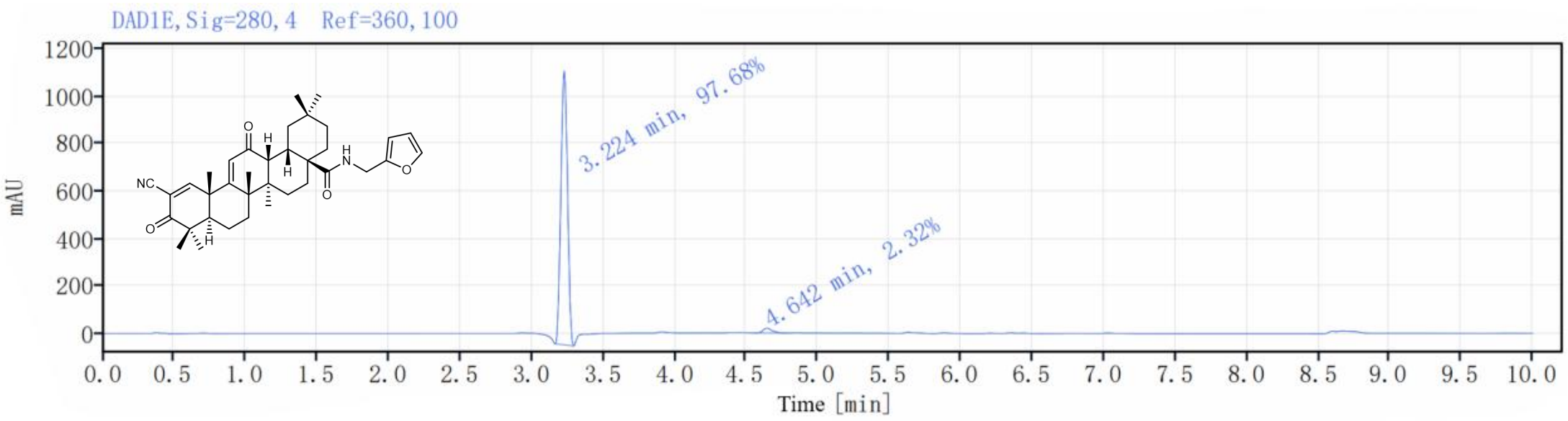

RT [min]	Signal Description	Width [min]	Sum Area	Height	Area%
3.224	DAD1E, Sig=280, 4 Ref=360, 100	0.131	3776.7	1160.6	97.68
4.642	DAD1E, Sig=280, 4 Ref=360, 100	0.227	89.8	20.6	2.32
		Sum	3866.5		

HRMS of LPH-A33

**Data Filename**	ESIH202503601.d	**Sample Name**	F1-LPH-A33
**Sample ID**		**Position**	P1-C1
**Instrument Name**	Agilent 6520 Q-TOF	**Acq Method**	20160322_MS_ESIH_POS_1min.m
**Acquired Time**	6/30/2025 2:31:17 PM	**DA Method**	ESI-HR-20231114.m
**Comment**	ESIH by fangsu		

User Spectra

Fragmentor Voltage	Collision Energy	Ionization Mode
170	0	ESI

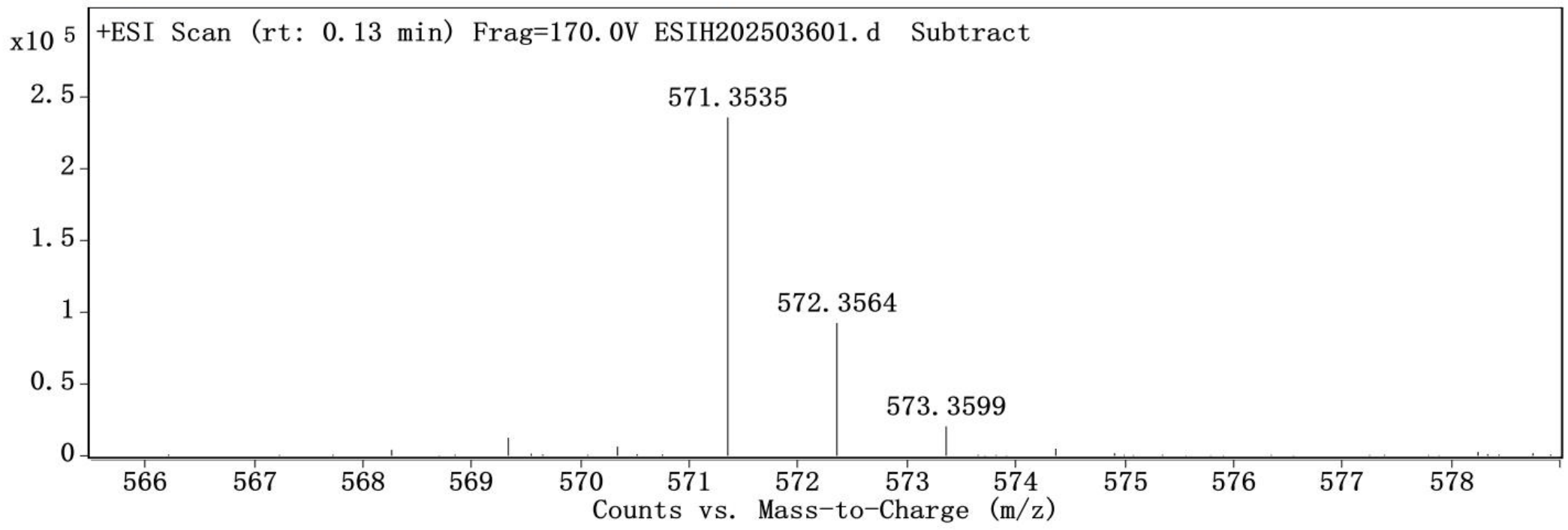

Formula Calculator Results

m/z	Calc m/z	Diff (mDa)	Diff (ppm)	Ion Formula	Ion
571.3535	571.353	-0.45	-0.78	C36 H47 N2 O4	(M+H)+

The synthesis of LPH-A40

The title compound was synthesized with **Method A** from **RTA 401** (50.0 mg, 0.10 mmol) and Cyclopentylamine (12 μL, 0.12 mmol). **LPH-A40** was obtained as a white solid (39.5 mg, 70%). ^{1}H NMR (400 MHz, $CDCl_3$) δ 8.05 (s, 1H), 5.96 (s, 1H), 5.66 (d, J = 7.3 Hz, 1H), 4.25–4.15 (m, 1H), 3.05 (d, J = 4.5 Hz, 1H), 2.85 (d, J = 13.0 Hz, 1H), 2.01-1.90 (m, 3H), 1.85–1.41 (m, 19H), 1.37–1.21 (m, 12H), 1.15 (s, 4H), 0.99 (s, 3H), 0.97 (s, 3H), 0.88 (s, 3H); ^{13}C NMR (101 MHz, $CDCl_3$) δ 199.15, 196.69, 176.65, 168.71, 165.93, 124.09, 114.64, 114.51, 51.34, 49.55, 47.80, 46.31, 46.03, 45.11, 42.65, 42.21, 36.18, 34.69, 34.13, 33.39, 33.26, 33.14, 32.01, 31.75, 30.72, 27.84, 27.08, 26.68, 24.90, 23.86, 23.25, 23.13, 21.84, 21.66, 18.33. HRMS (ESI) m/z: $[M + H]^+$ calcd for $C_{36}H_{51}N_2O_3$ 559.3894, found 559.3899.

^{1}H NMR (400 MHz, CDCl₃) of LPH-A40

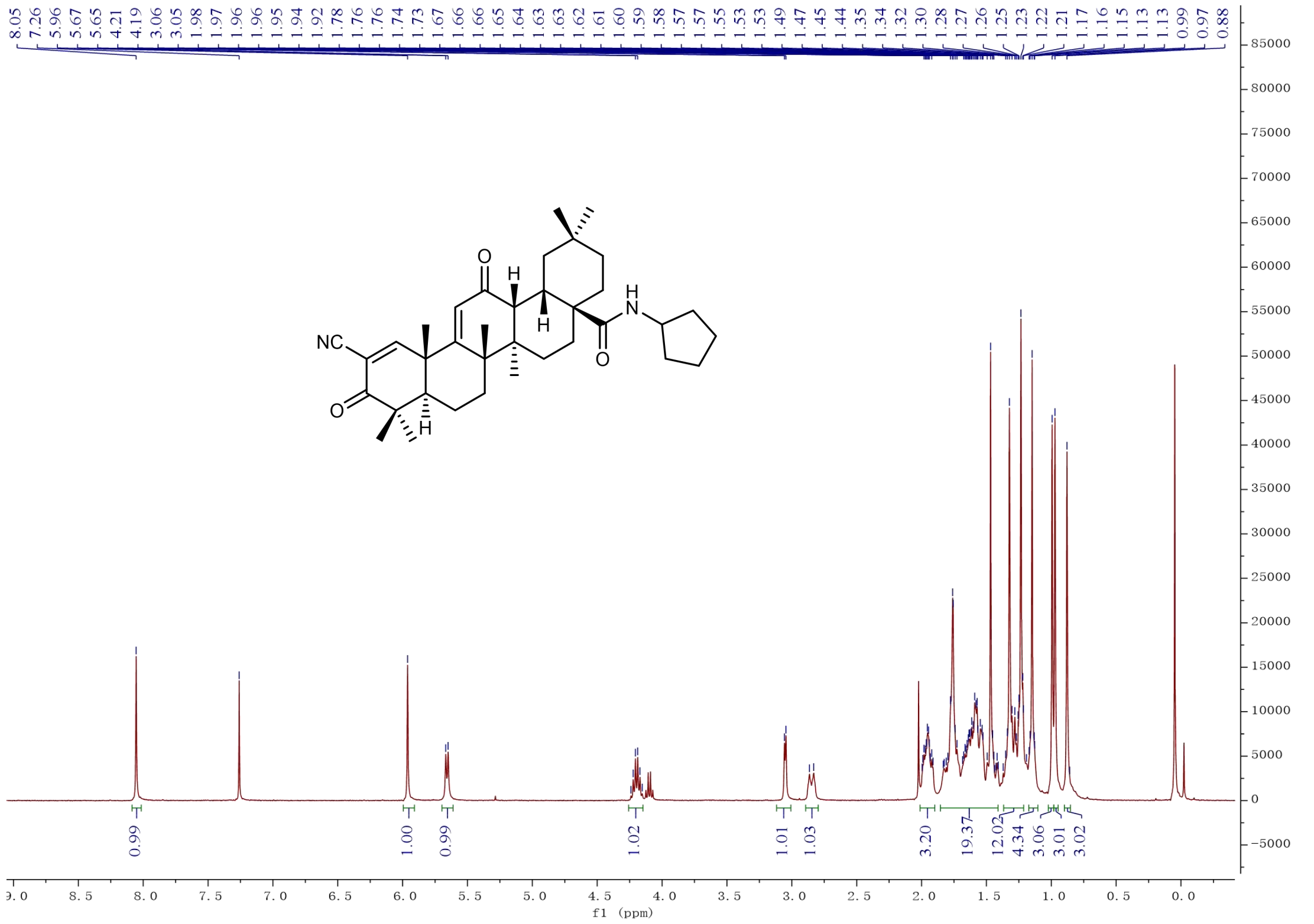

^{13}C NMR (101 MHz, CDCl₃) of LPH-A40

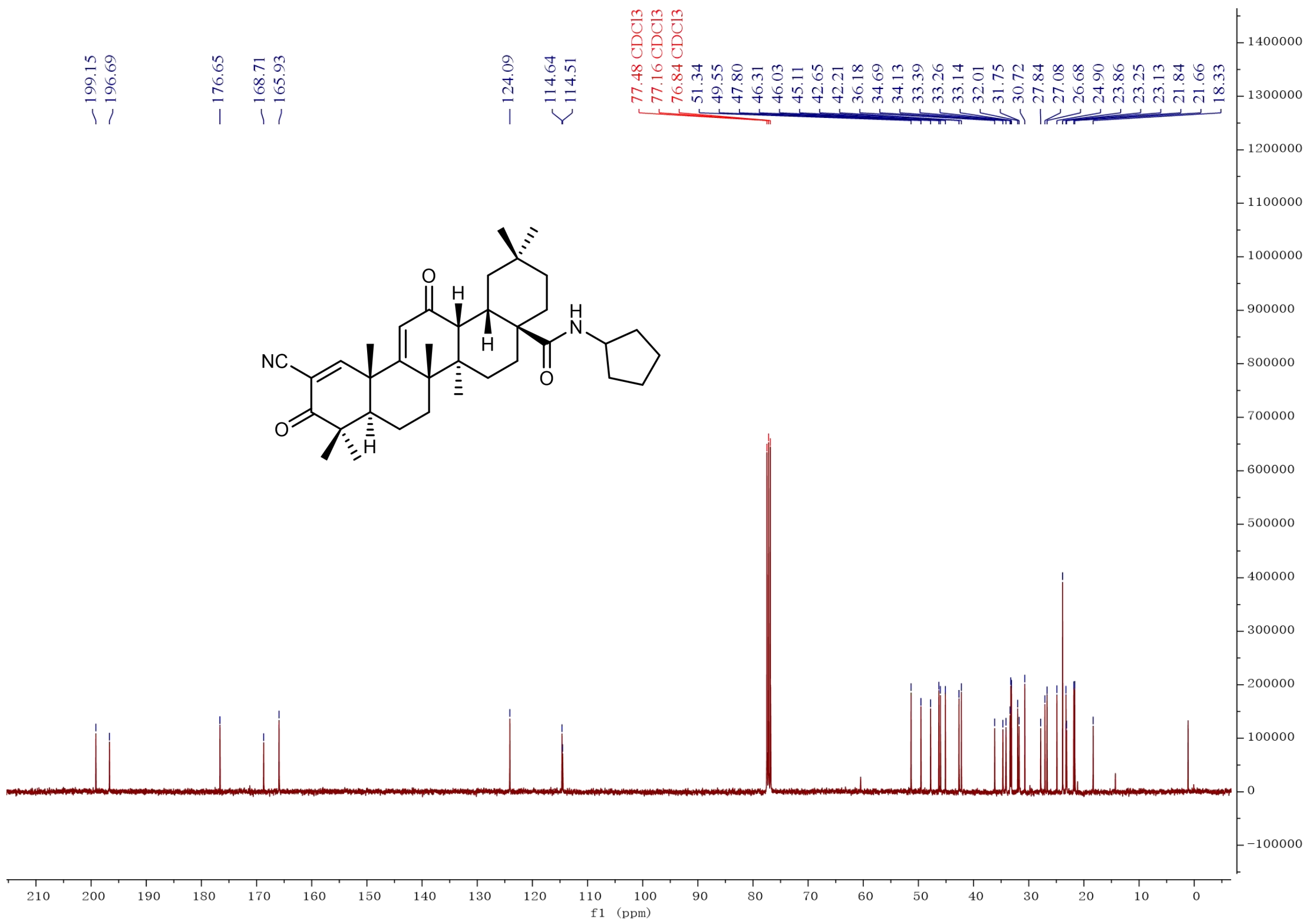

HPLC of LPH-A40

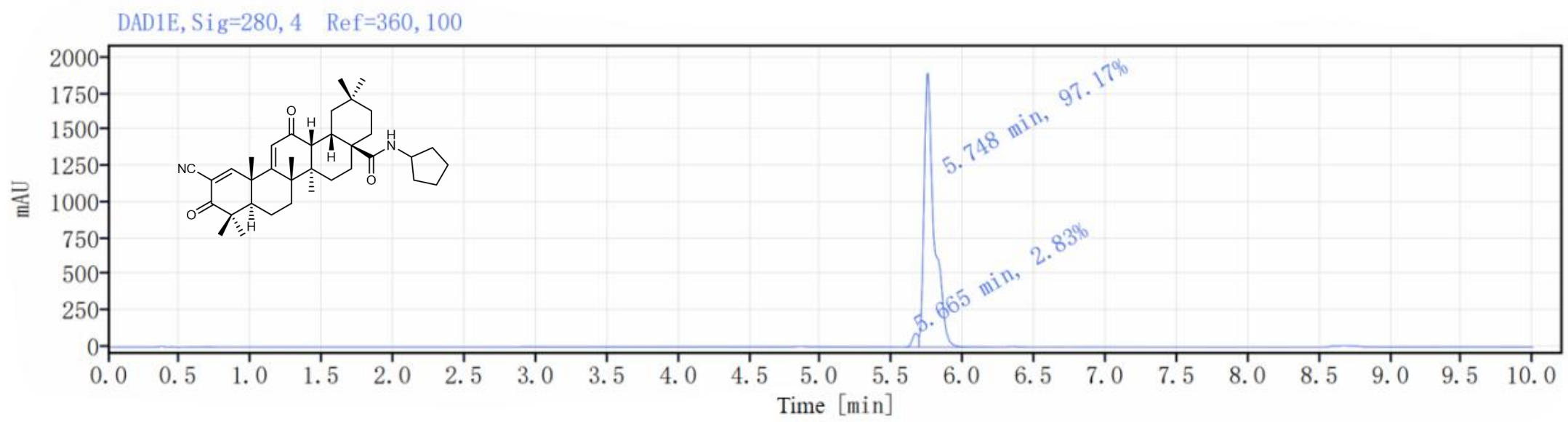

RT [min]	Signal Description	Width [min]	Sum Area	Height	Area%
5.665	DAD1E, Sig=280, 4 Ref=360, 100	0.116	273.7	96.7	2.83
5.748	DAD1E, Sig=280, 4 Ref=360, 100	0.405	9394.4	1907.6	97.17
		Sum	9668.1		

HRMS of LPH-A40

**Data Filename**	ESIH202503602.d	**Sample Name**	F1-LPH-A40
**Sample ID**		**Position**	P1-C2
**Instrument Name**	Agilent 6520 Q-TOF	**Acq Method**	20160322_MS_ESIH_POS_1min.m
**Acquired Time**	6/30/2025 2:32:34 PM	**DA Method**	ESI-HR-20231114.m
**Comment**	ESIH by fangsu		

User Spectra

Fragmentor Voltage	Collision Energy	Ionization Mode
170	0	ESI

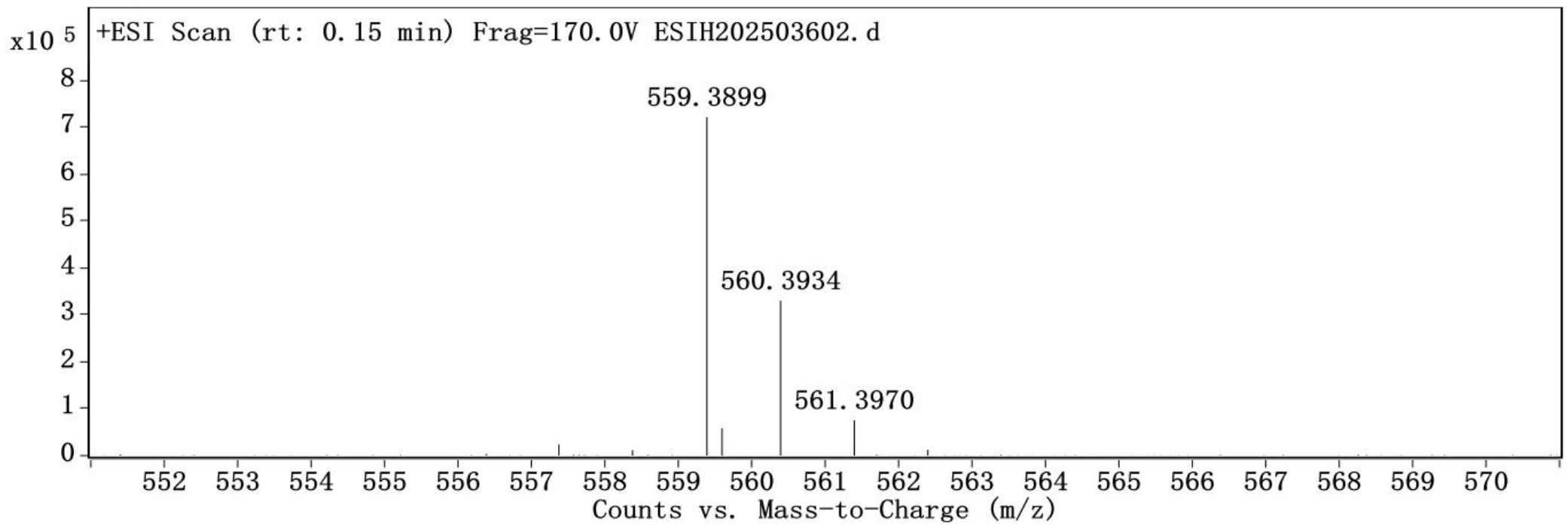

Formula Calculator Results

m/z	Calc m/z	Diff (mDa)	Diff (ppm)	Ion Formula	Ion
559.3899	559.3894	-0.47	-0.83	C36 H51 N2 O3	(M+H)+

The synthesis of LPH-A43

The title compound was synthesized with **Method B** from **RTA 401** (50.0 mg, 0.10 mmol) and *N,O*-Dimethylhydroxylamine hydrochloride (20.0 mg, 0.20 mmol). **LPH-A43** was obtained as a white solid (39.0 mg, 72%). ^{1}H NMR (500 MHz, $CDCl_3$) δ 8.03 (s, 1H), 5.93 (s, 1H), 3.67 (s, 3H), 3.23–3.18 (m, 1H), 3.15 (s, 3H), 3.02 (d, J = 4.2 Hz, 1H), 2.02–1.49 (m, 13H), 1.46 (s, 3H), 1.31 (s, 3H), 1.23 (s, 3H), 1.15 (s, 3H), 1.01 (s, 3H), 0.98 (s, 3H), 0.88 (s, 3H); ^{13}C NMR (125 MHz, $CDCl_3$) δ 199.34, 196.74, 178.60, 168.70, 165.97, 124.06, 114.62, 114.52, 60.58, 50.20, 48.10, 47.80, 45.94, 45.11, 42.63, 42.20, 36.42, 34.57, 34.45, 33.42, 31.70, 31.15, 30.76, 29.17, 28.30, 27.09, 26.70, 24.89, 23.27, 22.39, 22.24, 21.66, 18.34. HRMS (ESI) m/z: $[M + H]^+$ calcd for $C_{33}H_{47}N_2O_4$ 535.3530, found 535.3531.

^{1}H NMR (500 MHz, $CDCl_3$) of LPH-A43

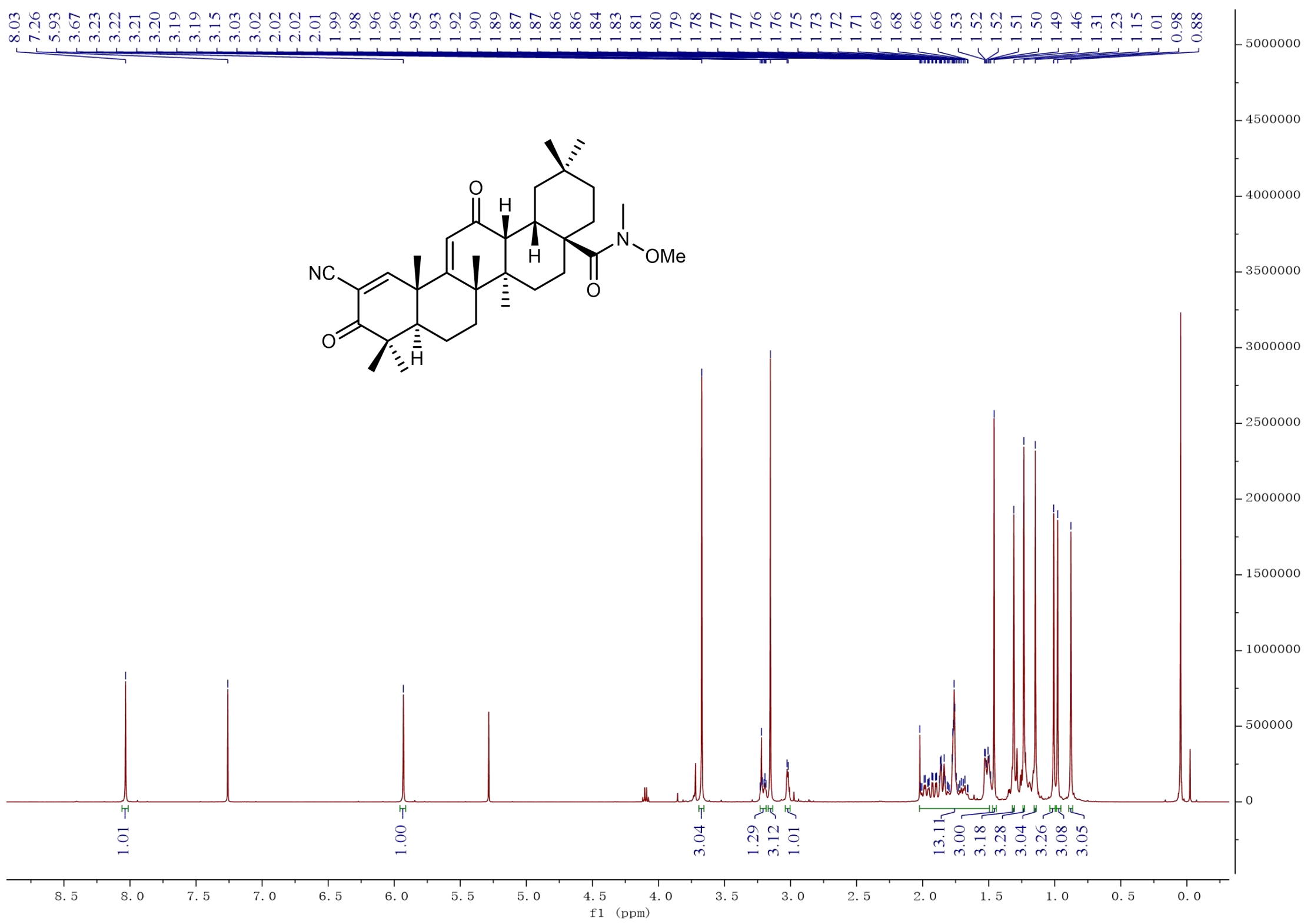

^{13}C NMR (125 MHz, $CDCl_3$) of LPH-A43.

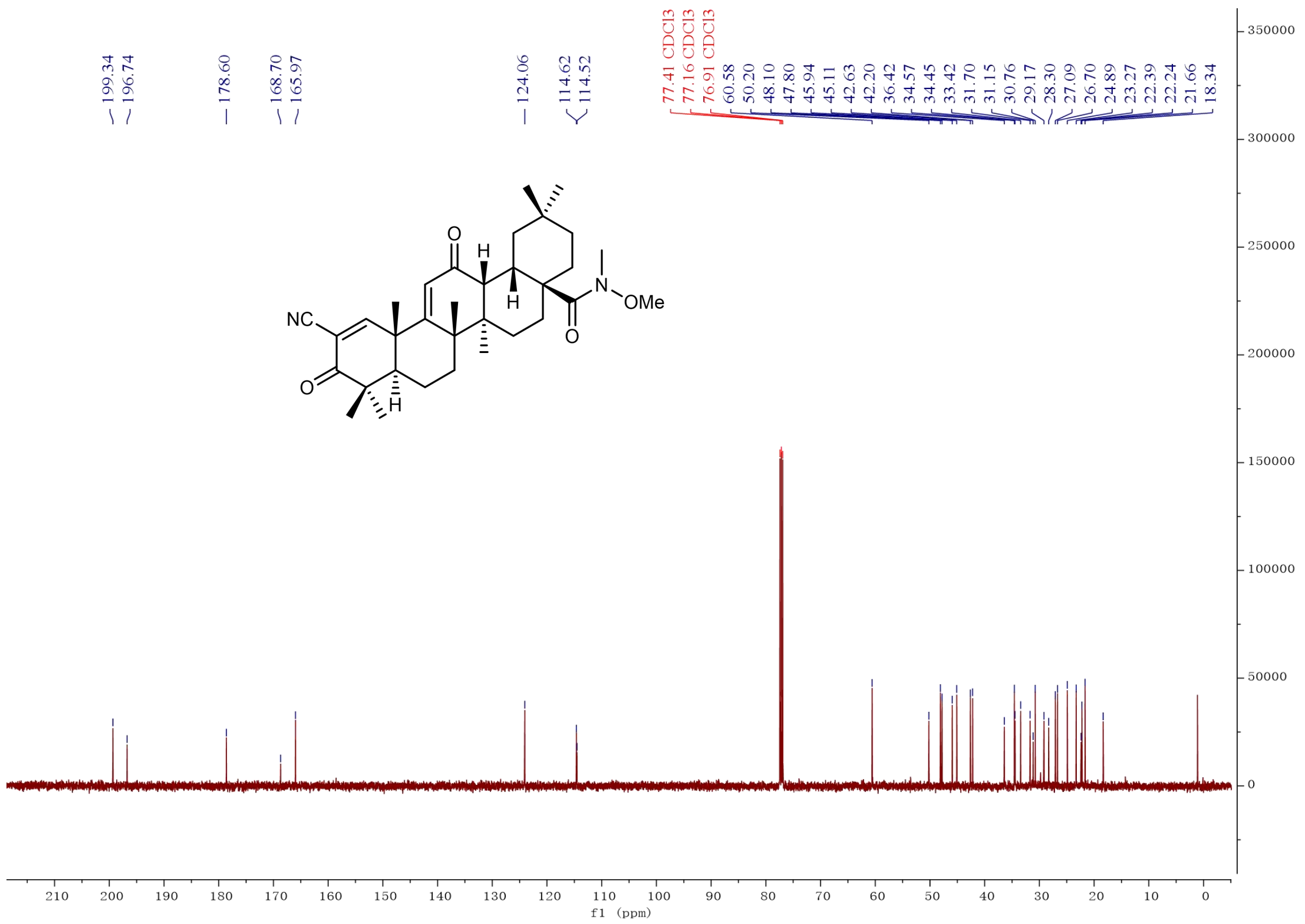

HPLC of LPH-A43

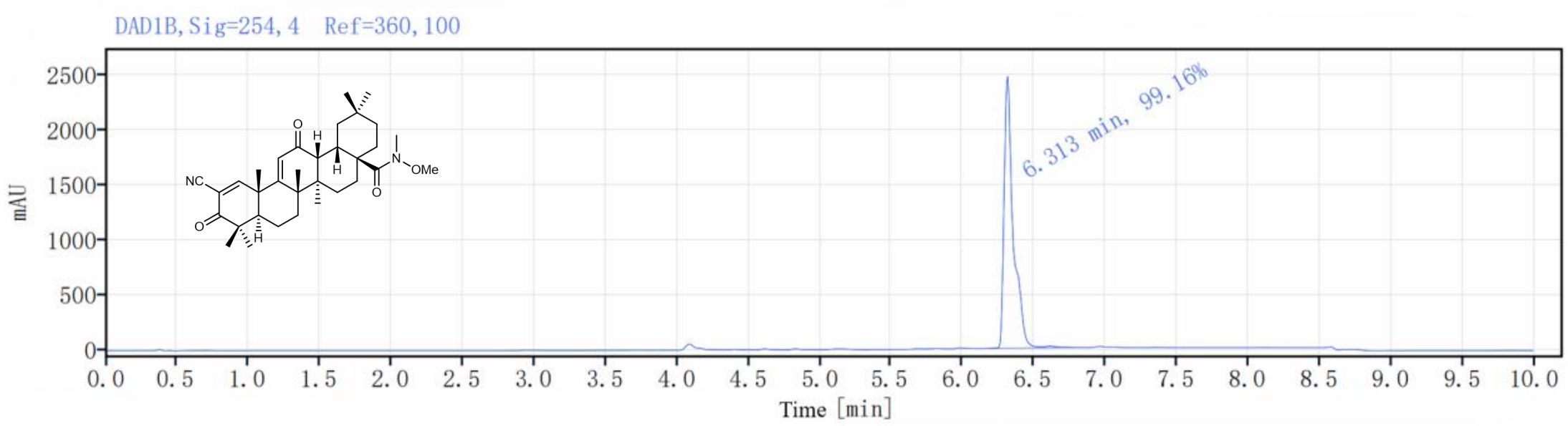

RT [min]	Signal Description	Width [min]	Sum Area	Height	Area%
6.313	DAD1B, Sig=254, 4 Ref=360, 100	0.448	11042.3	2474.4	99.16
		Sum	11042.3		

HRMS of LPH-A43

**Data Filename**	ESIH202503603.d	**Sample Name**	F1-LPH-A43
**Sample ID**		**Position**	P1-C3
**Instrument Name**	Agilent 6520 Q-TOF	**Acq Method**	20160322_MS_ESIH_POS_1min.m
**Acquired Time**	6/30/2025 2:33:51 PM	**DA Method**	ESI-HR-20231114.m
**Comment**	ESIH by fangsu		

User Spectra

Fragmentor Voltage	Collision Energy	Ionization Mode
170	0	ESI

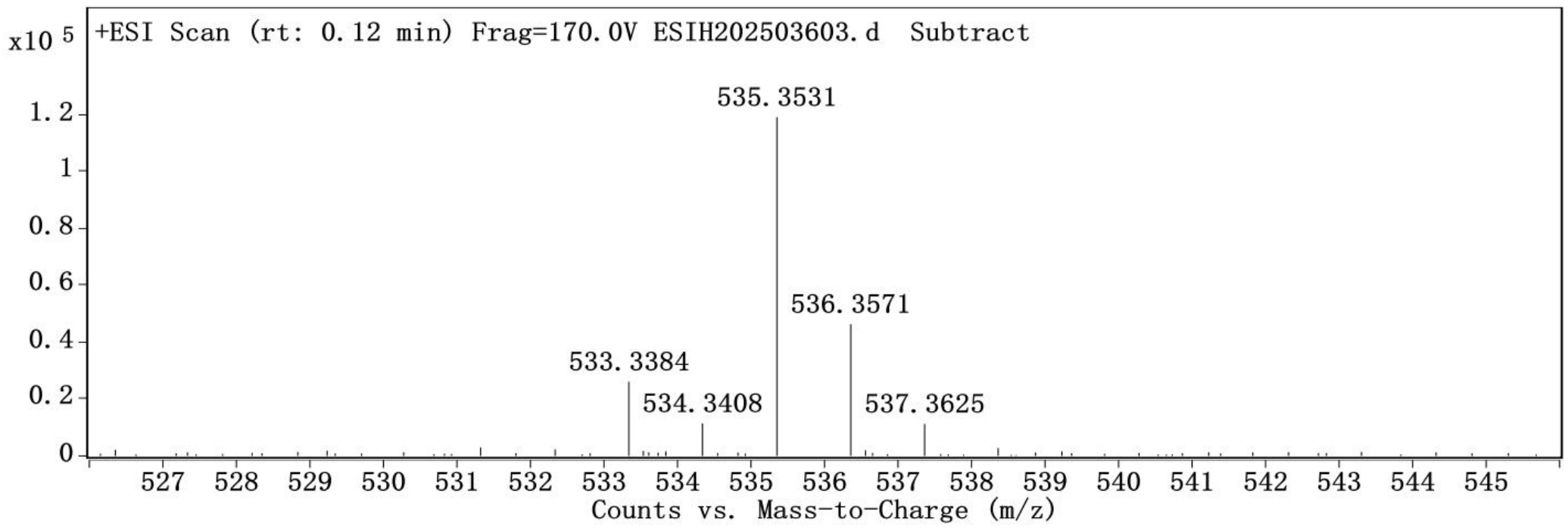

Formula Calculator Results

m/z	Calc m/z	Diff (mDa)	Diff (ppm)	Ion Formula	Ion
535.3531	535.353	-0.1	-0.19	C33 H47 N2 O4	(M+H)+

The synthesis of LPH-A134

Compound **RTA 401** (200.0 mg, 0.41 mmol) was reacted with [2-(2-aminoethoxy)ethyl]carbamic acid tert-butyl ester (100.0 mg, 0.49 mmol) using **Method A** to afford the intermediate (275.0 mg, 99.7%). Subsequently, trifluoroacetic acid (623 μL, 8.14 mmol) was used to remove the Boc and **LPH-A134** was obtained as a white solid (212.0 mg, 90%). ^{1}H NMR (600 MHz, $CDCl_3$) *δ* 8.11 (s, 1H), 6.86 (s, 1H), 5.99 (s, 1H), 4.19–4.03 (m, 2H), 3.67–3.35 (m, 8H), 3.04 (d, *J* = 4.5 Hz, 1H), 2.91 (d, *J* = 12.8 Hz, 1H), 1.46 (s, 3H), 1.31 (s, 3H), 1.22 (s, 3H), 1.13 (s, 3H), 0.98 (s, 3H), 0.95 (s, 3H), 0.85 (s, 3H); ^{13}C NMR (151 MHz, $CDCl_3$) *δ* 199.67, 196.73, 177.66, 169.16, 166.17, 130.94, 124.03, 114.49, 114.49, 69.89, 68.22, 64.92, 49.62, 47.72, 46.57, 46.57, 46.00, 45.08, 42.71, 42.17, 39.32, 36.21, 34.69, 33.94, 33.40, 31.83, 30.71, 29.74, 27.84, 27.00, 24.81, 23.29, 21.82, 21.63, 18.31. HRMS (ESI) m/z: $[M + H]^+$ calcd for $C_{35}H_{52}N_3O_4$ 578.3952, found 578.3953.

^{1}H NMR (600 MHz, $CDCl_3$) of LPH-A134

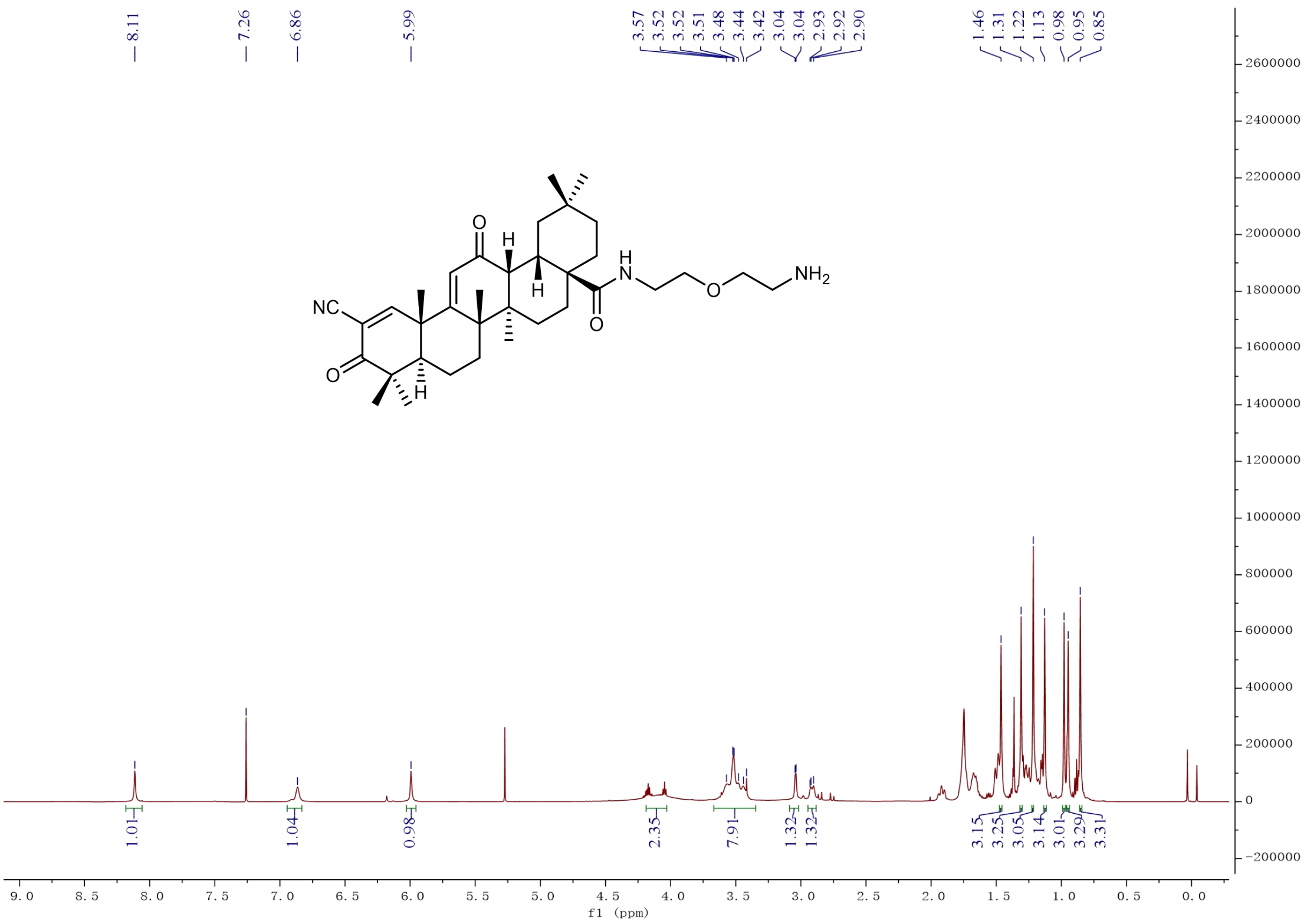

^{13}C NMR (151 MHz, $CDCl_3$) of LPH-A134.

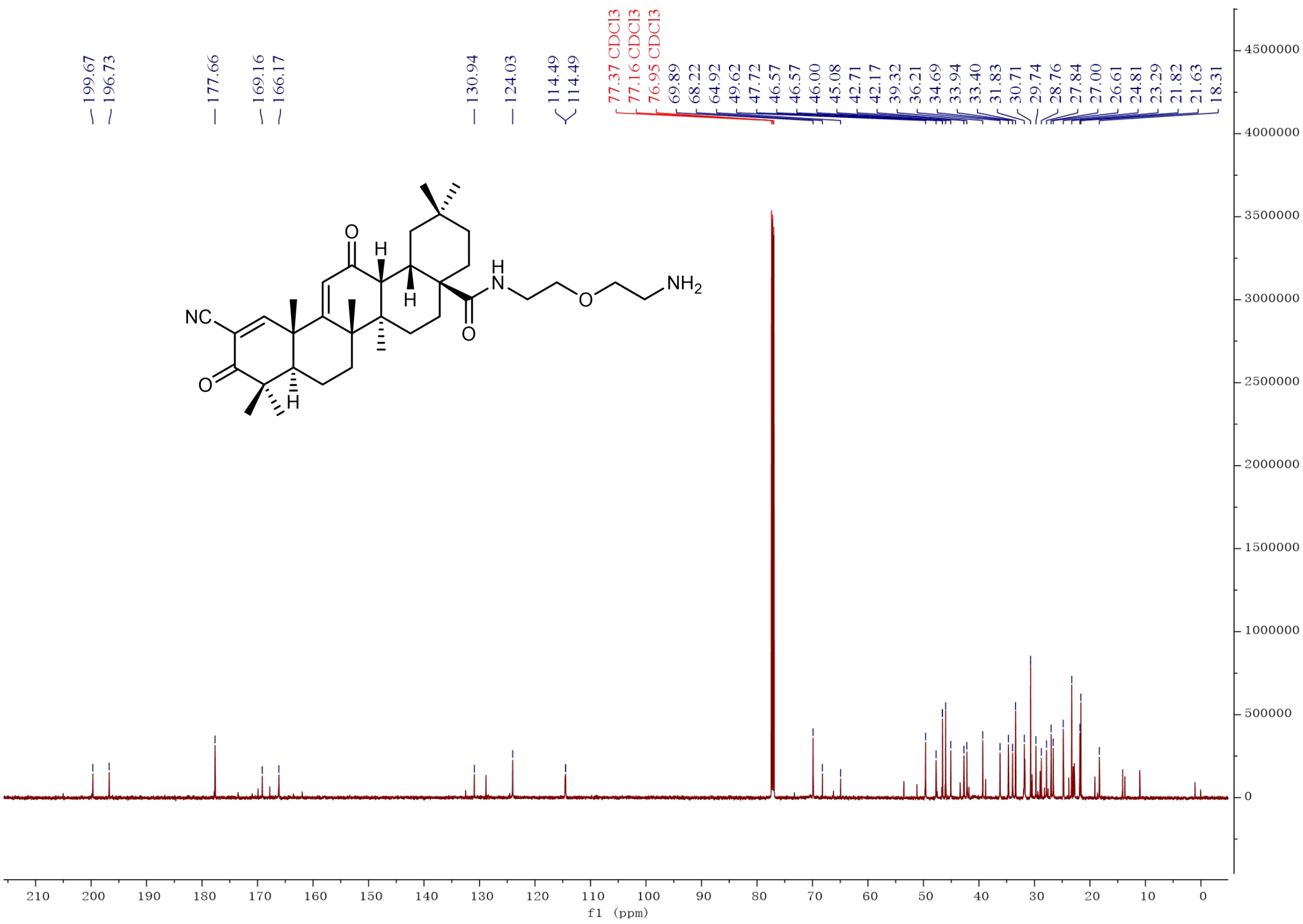

HPLC of LPH-A134

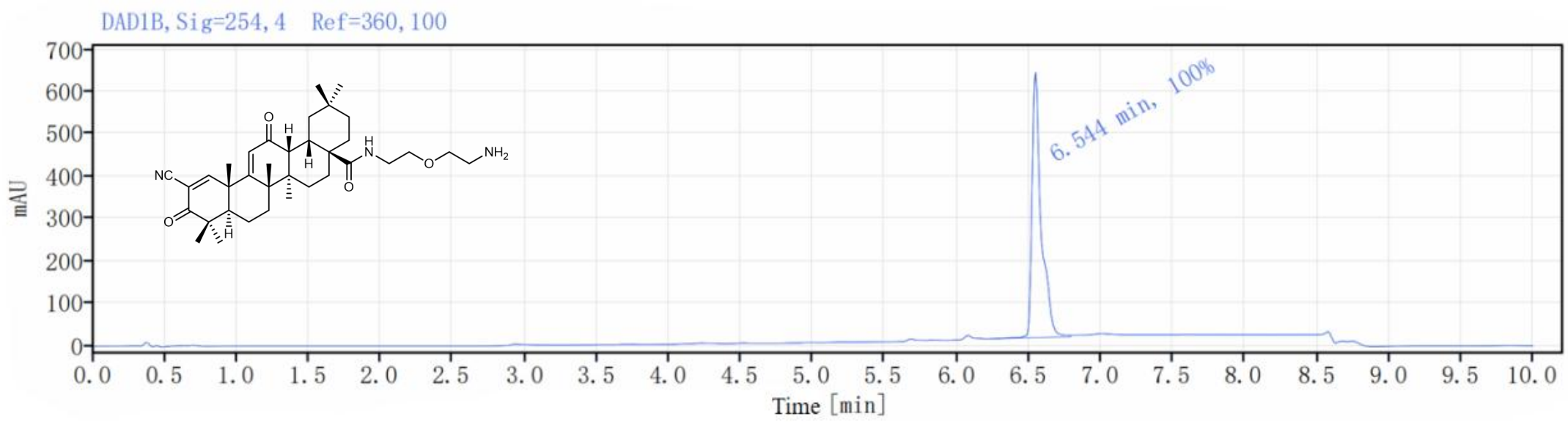

RT [min]	Signal Description	Width [min]	Sum Area	Height	Area%
6.544	DAD1B, Sig=254, 4 Ref=360, 100	0.563	2775.9	629.6	100.00
		Sum	2775.9		

HRMS of LPH-A134

**Data Filename**	ESIH202503609.d	**Sample Name**	F1-LPH-A134
**Sample ID**		**Position**	P1-C9
**Instrument Name**	Agilent 6520 Q-TOF	**Acq Method**	20160322_MS_ESIH_POS_1min.m
**Acquired Time**	6/30/2025 2:41:28 PM	**DA Method**	ESI-HR-20231114.m
**Comment**	ESIH by fangsu		

User Spectra

Fragmentor Voltage	Collision Energy	Ionization Mode
170	0	ESI

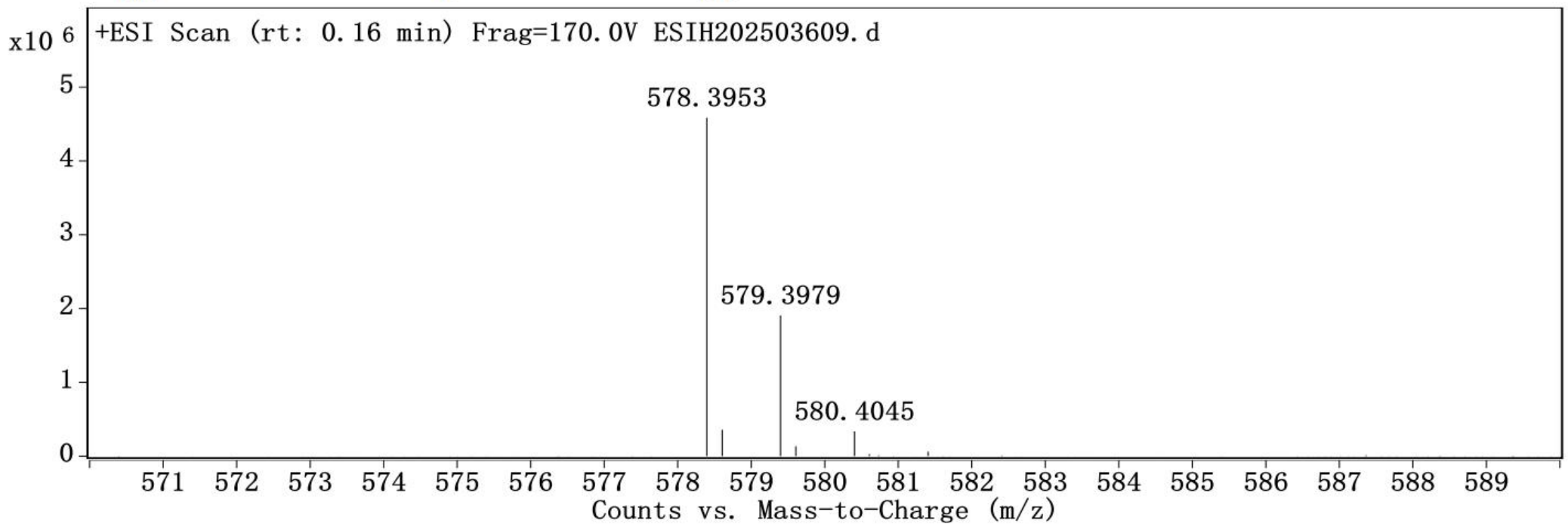

Formula Calculator Results

m/z	Calc m/z	Diff (mDa)	Diff (ppm)	Ion Formula	Ion
578.3953	578.3952	-0.03	-0.05	C35 H52 N3 O4	(M+H)+

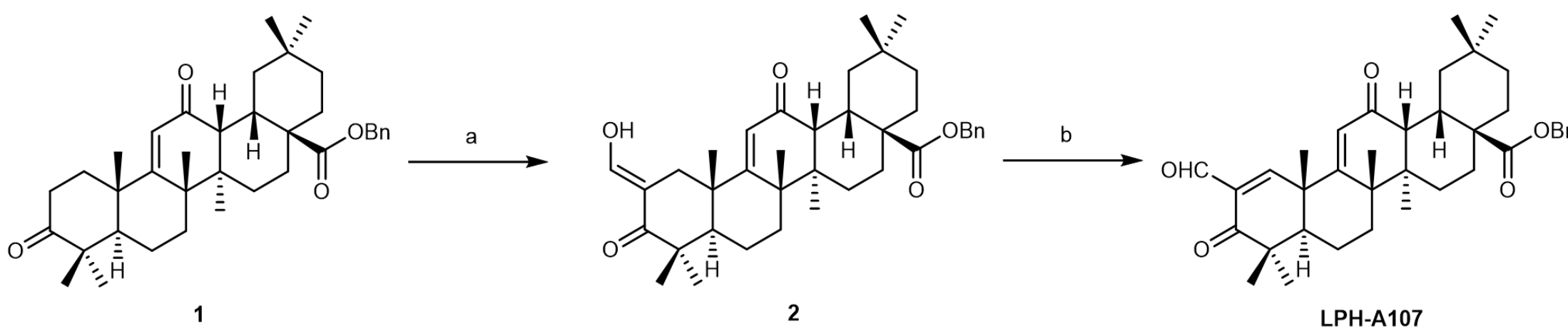

Supplementary Fig. 14 The synthetic route of LPH-A107.

Reagent and conditions: (a) HCO_2Et, MeONa, CH_2Cl_2; (b) PhSeCl/pyr., CH_2Cl_2; 30% H_2O_2, CH_2Cl_2.

Preparation of Benzyl 2-Hydroxymethylene-3,12-dioxoolean-9(11)-en-28-oate (2)[1]. To a solution of **1** (3.5 g, 6.26 mmol) in dry CH_2Cl_2 (12.5 mL) were added ethyl formate (2.0 mL, 25.05 mmol) and NaOMe (2.0 g, 37.58 mmol). The mixture was stirred at room temperature for 5 h. Then the mixture was diluted with a mixture of CH_2Cl_2 and Et_2O (1:2) and washed with 5% aqueous HCl solution (three times). The washings were re-extracted with a mixture of CH_2Cl_2 and Et_2O (1:2), and the combined organic layers were washed with saturated $NaHCO_3$ solution and brine and dried over Na_2SO_4. The solvent was removed and the crude product was purified by column chromatography (hexanes/EtOAc = 5:1) to obtain **2** (3.5 g, 95%) as an amorphous solid.

Preparation of Benzyl 2-Formyl-3,12-dioxooleana-1,9(11)-dien-28-oate (LPH-A107). To a solution of phenylselenenyl chloride (1.3 g, 6.82 mmol) in CH_2Cl_2 (64.0 mL) in an ice bath was added a solution of pyridine (593.0 mg, 7.50 mmol) in CH_2Cl_2 (16.0 mL). After 15 min, a solution of **2** (2.0 g, 3.41 mmol) in CH_2Cl_2 (28.0 mL) was added and the mixture was stirred an additional 1 h. After the mixture was washed with 10% aqueous HCl solution (32.0 mL) twice, 30% H_2O_2 (4.0 mL) was added to the stirred mixture in the ice bath. After an additional 40 min, the mixture was worked up according to the standard method to give a liquid. The liquid was subjected to flash column chromatography (hexanes/EtOAc = 2:1) to give **LPH-A107** as a colorless liquid (1.2 g, 58%). ^{1}H NMR (600 MHz, $CDCl_3$) δ 9.93 (s, 1H), 8.01 (s, 1H), 7.30–7.22 (m, 5H), 6.02 (s, 1H), 5.10 (d, J = 12.2 Hz, 1H), 5.06 (d, J = 12.0 Hz, 1H), 3.02 (d, J = 13.6 Hz, 1H), 2.75 (d, J = 4.4 Hz, 1H), 1.32 (s, 3H), 1.14 (s, 3H), 1.10 (s, 3H), 0.94 (s, 3H), 0.91 (s, 3H), 0.90 (s, 3H), 0.82 (s, 3H); ^{13}C NMR (151 MHz, $CDCl_3$) δ 201.96, 198.87, 189.54, 177.29, 169.65, 161.23, 136.28, 131.25, 128.52, 128.48, 128.17, 124.35, 66.12, 49.61, 47.94, 47.06, 45.69, 45.17, 42.01, 41.85, 34.45, 33.23,

32.72, 31.56, 30.62, 27.85, 27.26, 26.92, 24.63, 24.21, 23.10, 22.66, 21.49, 21.40, 19.86, 18.39.

HRMS (ESI) m/z: $[M + H]^+$ calcd for $C_{38}H_{48}O_5$ 585.3575, found 585.3578.

^{1}H NMR (600 MHz, $CDCl_3$) of LPH-A107

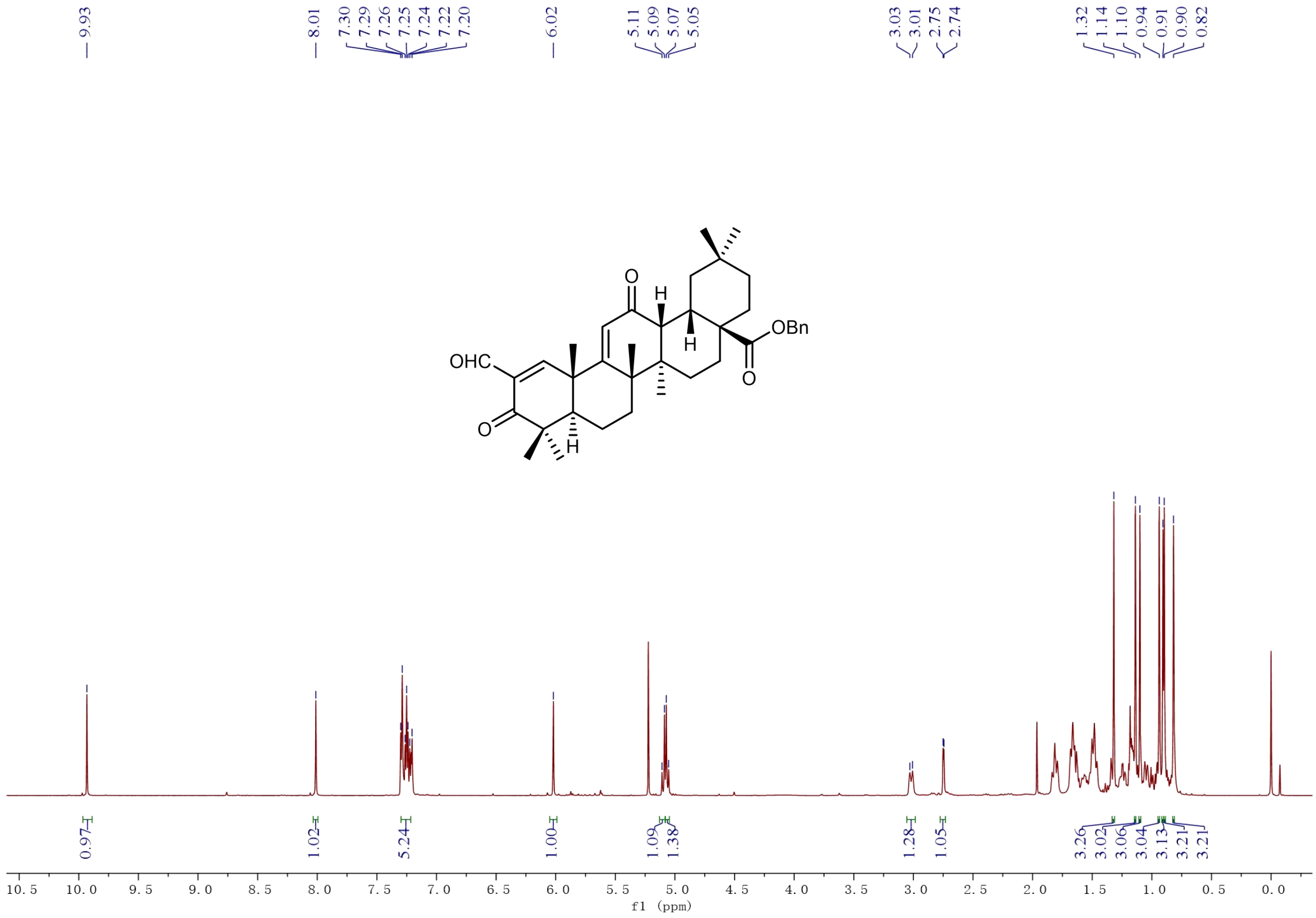

^{13}C NMR (151 MHz, $CDCl_3$) of LPH-A107.

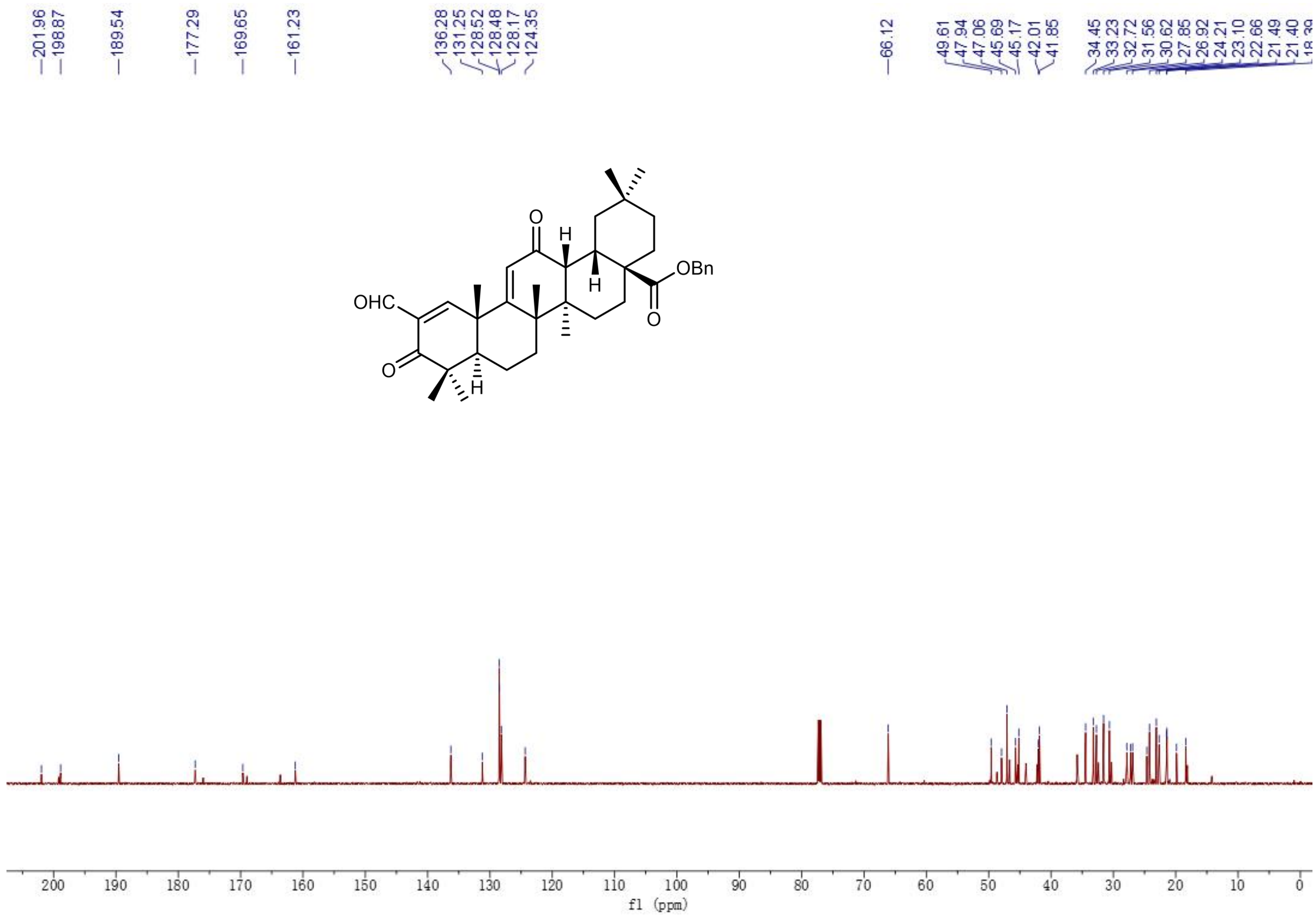

HPLC of LPH-A107

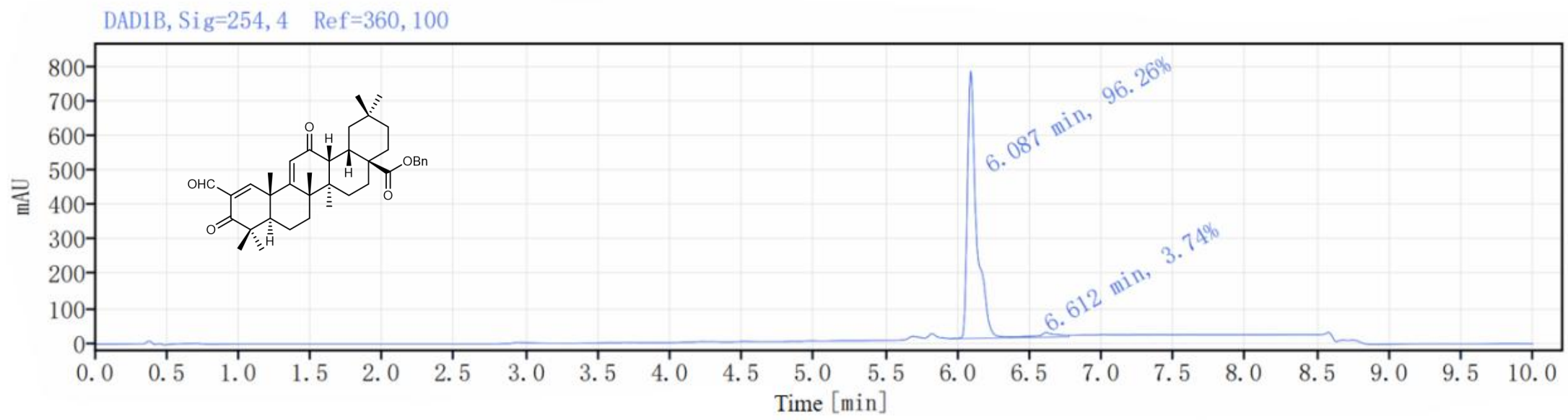

RT [min]	Signal Description	Width [min]	Sum Area	Height	Area%
6.087	DAD1B, Sig=254, 4 Ref=360, 100	0.422	3461.5	775.3	96.26
6.612	DAD1B, Sig=254, 4 Ref=360, 100	0.403	134.5	13.5	3.74
		Sum	3596.1		

HRMS of LPH-A107

**Data Filename**	ESIH202503608.d	**Sample Name**	F1-LPH-A107
**Sample ID**		**Position**	P1-C8
**Instrument Name**	Agilent 6520 Q-TOF	**Acq Method**	20160322_MS_ESIH_POS_1min.m
**Acquired Time**	6/30/2025 2:40:11 PM	**DA Method**	ESI-HR-20231114.m
**Comment**	ESIH by fangsu		

User Spectra

Fragmentor Voltage	Collision Energy	Ionization Mode
170	0	ESI

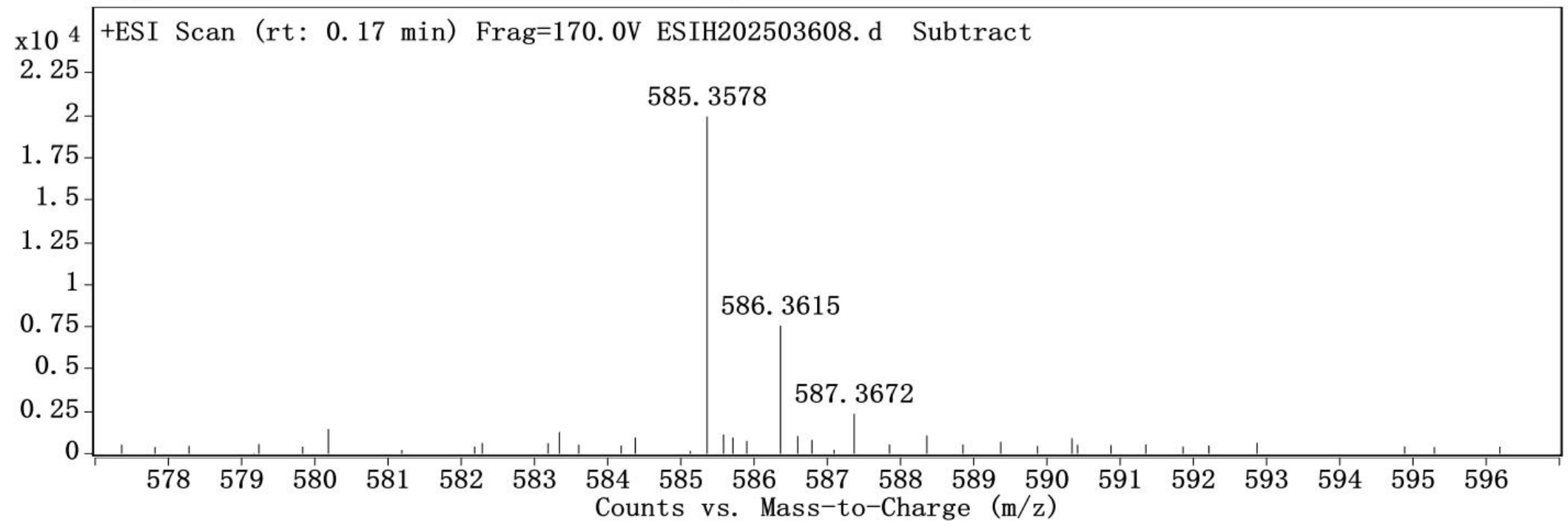

Formula Calculator Results

m/z	Calc m/z	Diff (mDa)	Diff (ppm)	Ion Formula	Ion
585.3578	585.3575	-0.3	-0.52	C38 H49 O5	(M+H)+

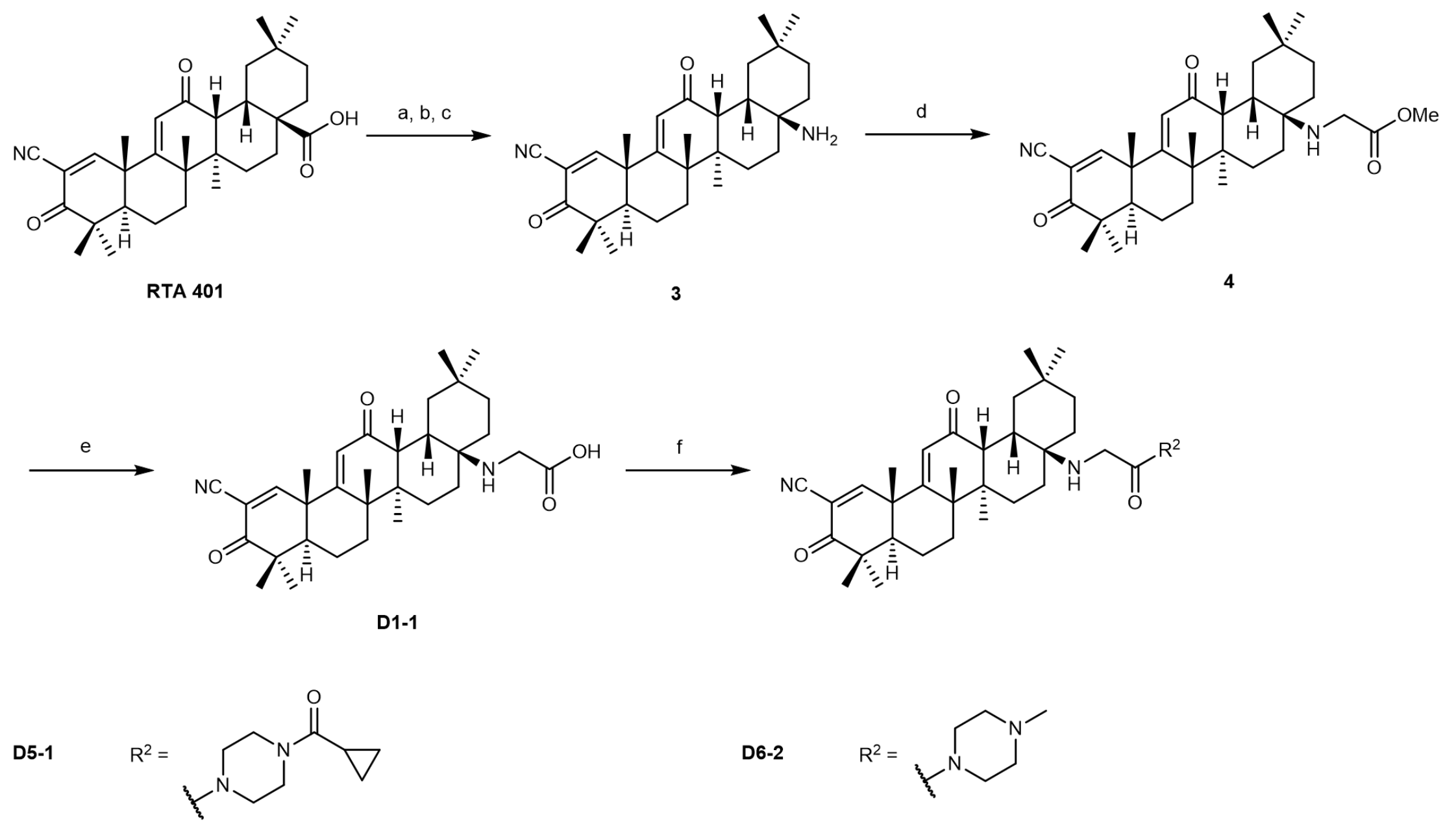

Supplementary Fig. 15 The synthetic route of D1-1, D5-1 and D6-2.

Reagent and conditions: (a) DPPA, Et_3N, Toluene, rt; (b) Benzene, 80 °C; (c) MeCN, HCl, 10% NaOH; (d) Methyl bromoacetate, DIPEA, MeCN, 60 °C; (e) $LiOH \cdot H_2O$, $MeOH/H_2O$, rt; (f) RNH, HATU, DIPEA, DMF, rt.

Preparation of compound 3. RTA 401 (531.0 mg, 1.08 mmol) and triethylamine (0.5 mL, 3.24 mmol) were dissolved in dry toluene (10.8 mL). Diphenyl phosphoryl azide (DPPA) (0.4 mL, 1.62 mmol) was added dropwise at 0 °C. After 15 min, the reaction mixture was warmed to room temperature. The reaction progress was monitored by TLC. Upon completion, purification by flash chromatography (hexanes/EtOAc = 4:1) to obtain a white solid (431.7 mg, 77.4%). The above solid was dissolved in toluene (9.8 mL) and refluxed at 80 °C for 2 h. The solvent was removed under reduced pressure to afford the crude product, which was used directly in the next step. The crude was dissolved in acetonitrile (4.1 mL). Concentrated HCl (12 N, 1.9 mL) was added dropwise at 0 °C. The mixture was then warmed to room temperature and stirred for 1 h. The mixture was cooled again to 0 °C, and 10% NaOH (10.3 mL) was added dropwise with stirring. The mixture was warmed to room temperature and stirred for 1 h. The mixture was then extracted with EtOAc. The organic was washed with water, followed by saturated brine, dried over anhydrous Na_2SO_4, filtered, and concentrated under reduced pressure to afford **3** (345.4 mg, 89%) as a white solid.

Preparation of compound 4. Compound **3** (150.0 mg, 0.32 mmol) was dissolved in dry acetonitrile (5.4 mL). DIPEA (0.5 mL, 3.24 mmol) and methyl bromoacetate (0.3 mL, 2.92 mmol) were added. The reaction mixture was stirred at 60 °C until TLC analysis indicated complete. Upon completion, purification by flash chromatography (hexanes/EtOAc = 3:1) to obtain **4** (159.8 mg, 92%) as a white solid.

The synthesis of D1-1

Compound **4** (50.0 mg, 0.09 mmol) was dissolved in methanol (1.6 mL) and water (0.5 mL). $LiOH\cdot H_2O$ (9.8 mg, 0.23 mmol) was added, and the mixture was stirred at room temperature until TLC analysis indicated complete. After completion, the mixture was cooled to 0 °C and acidified dropwise with dilute HCl under stirring. The mixture was extracted with EtOAc. The organic was washed with water, followed by saturated brine, dried over anhydrous Na_2SO_4, filtered, and concentrated under reduced pressure. Purification by preparative thin-layer chromatography (PTLC) (DCM/MeOH = 30:1) to obtain **D1-1** (47.4 mg, 97%) as a white solid. ^{1}H NMR (600 MHz, $CDCl_3$) δ 8.10 (s, 1H), 6.06 (s, 1H), 3.82 (d, J = 16.4 Hz, 1H), 3.71 (d, J = 16.6 Hz, 1H), 3.16 (d, J = 4.7 Hz, 1H), 2.79 (d, J = 10.8 Hz, 1H), 2.15 (t, J = 12.8 Hz, 1H), 1.96 (t, J = 11.1 Hz, 1H), 1.54 (s, 3H), 1.51 (s, 3H), 1.25 (s, 3H), 1.17 (s, 3H), 1.06 (s, 3H), 0.95 (s, 3H), 0.91 (s, 3H). ^{13}C NMR (151 MHz, $CDCl_3$) δ 197.00, 195.46, 170.86, 168.46, 164.59, 122.94, 113.63, 113.45, 58.24, 52.44, 46.69, 45.76, 45.12, 44.05, 41.59, 40.94, 35.83, 33.87, 32.75, 31.72, 30.70, 29.78, 29.48, 25.95, 25.71, 24.10, 24.01, 22.01, 21.34, 20.78, 20.60, 17.21. HRMS (ESI) m/z: $[M + H]^+$ calcd for $C_{32}H_{45}N_2O_4$ 521.3374, found 521.3375.

^{1}H NMR (600 MHz, $CDCl_3$) of D1-1

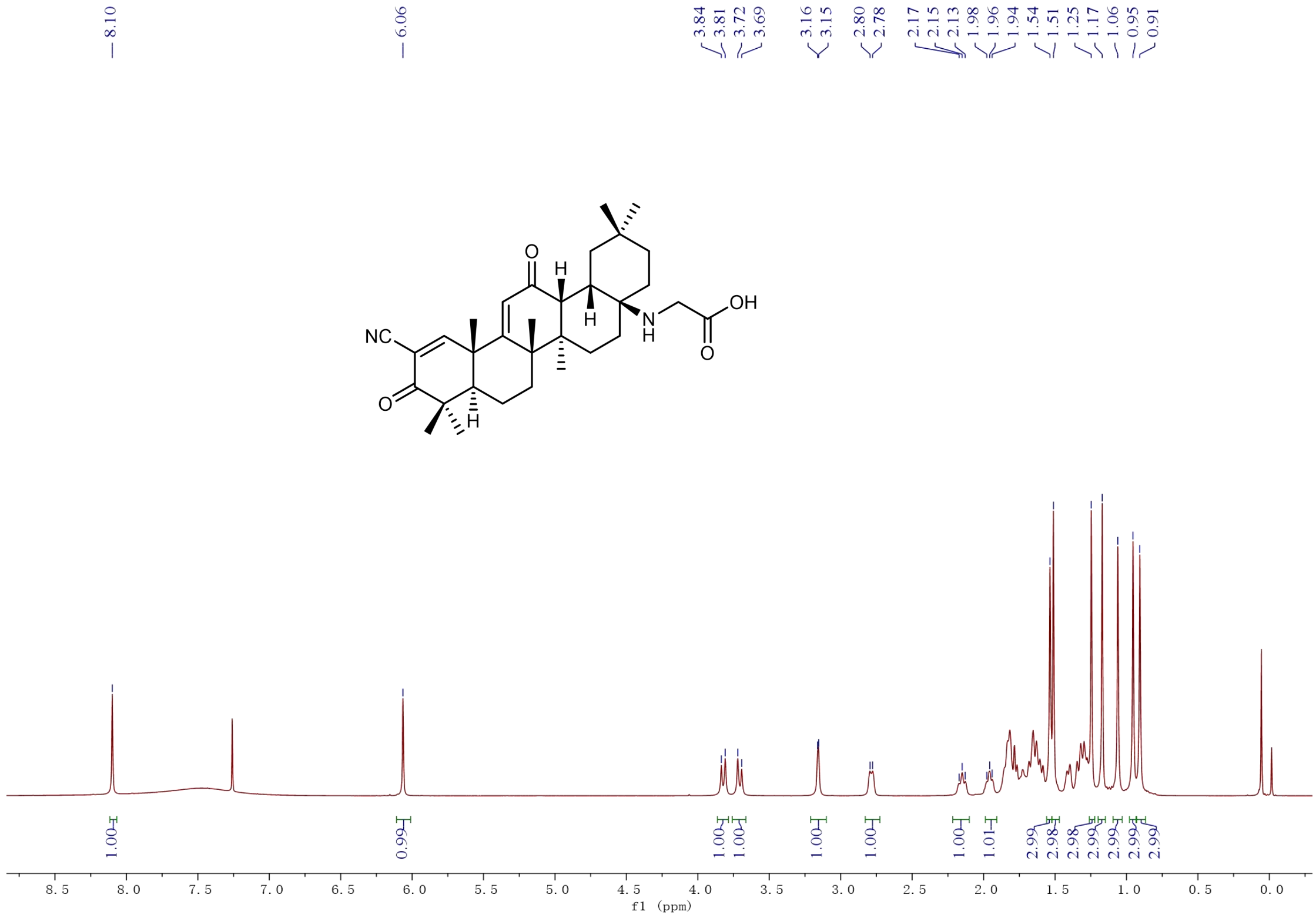

^{13}C NMR (151 MHz, $CDCl_3$) of D1-1

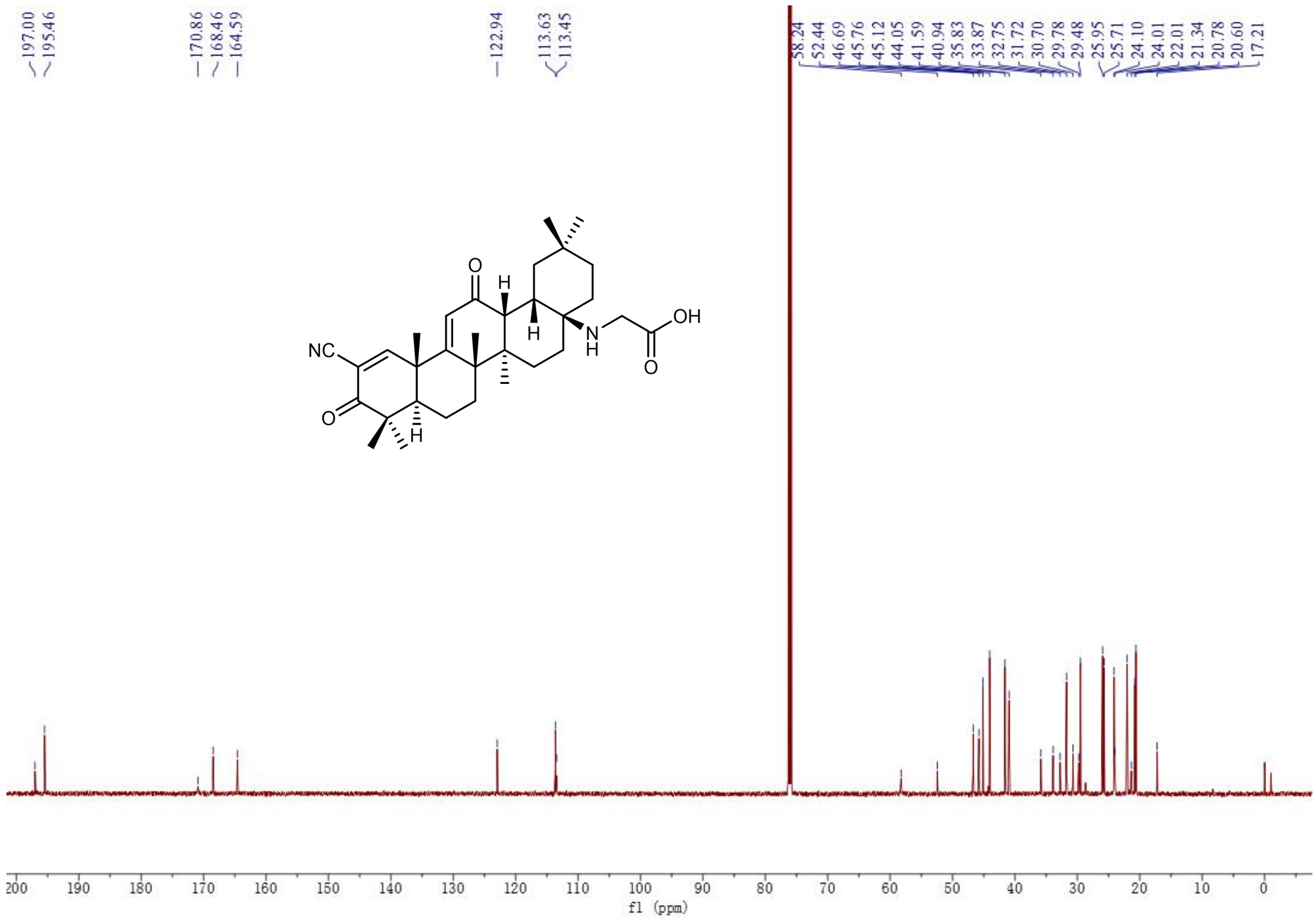

HPLC of D1-1

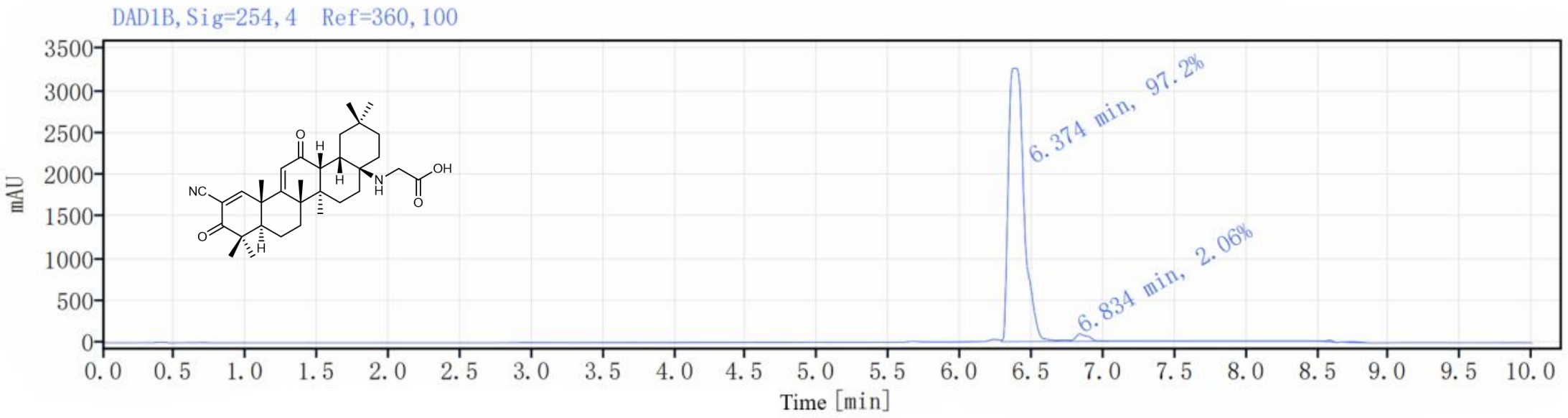

RT [min]	Signal Description	Width [min]	Sum Area	Height	Area%
6.374	DAD1B, Sig=254, 4 Ref=360, 100	0.394	24973.0	3262.3	97.20
6.834	DAD1B, Sig=254, 4 Ref=360, 100	0.274	530.4	87.4	2.06
		Sum	25503.4		

HRMS of D1-1

**Data Filename**	ESIH202503768.d	**Sample Name**	F1-D1-1
**Sample ID**		**Position**	P1-A9
**Instrument Name**	Agilent 6520 Q-TOF	**Acq Method**	20160322_MS_ESIH_POS_1min.m
**Acquired Time**	7/4/2025 2:41:38 PM	**DA Method**	ESI-HR-20231114.m
**Comment**	ESIH by fangsu		

User Spectra

Fragmentor Voltage	Collision Energy	Ionization Mode
170	0	ESI

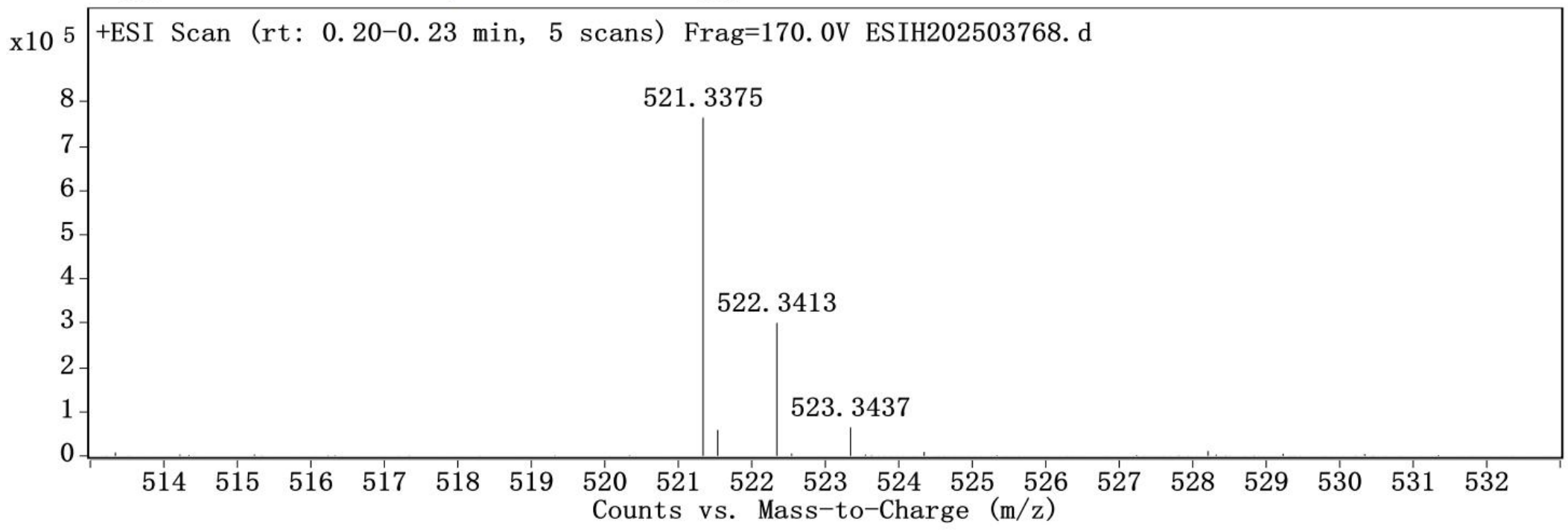

Formula Calculator Results

m/z	Calc m/z	Diff (mDa)	Diff (ppm)	Ion Formula	Ion
521.3375	521.3374	-0.16	-0.31	C32 H45 N2 O4	(M+H)+

The synthesis of D5-1

Compound **D1-1** (50.0 mg, 0.10 mmol) was dissolved in dry DMF (1.9 mL). HATU (66.9 mg, 0.18 mmol) was added, and the mixture was stirred at room temperature for 30 min. DIPEA (34 µL, 0.21 mmol) and 1-(cyclopropanecarbonyl)piperazine (11 µL, 0.08 mmol) were then added to the reaction mixture. The reaction was stirred at room temperature overnight. Upon completion, the mixture was diluted with water and extracted with EtOAc three times. The combined organic layers were washed with saturated brine, dried over anhydrous Na_2SO_4, and filtered. The filtrate was concentrated under reduced pressure to afford the crude product. The crude material was purified by automated flash chromatography (hexanes/EtOAc = 1:1) and semi-preparative HPLC to afford the product **D5-1** (3.8 mg, 7%) as a white solid. ^1H NMR (600 MHz, $CDCl_3$) δ 8.02 (s, 1H), 6.04 (s, 1H), 3.88–3.72 (m, 6H), 3.61–3.49 (m, 4H), 3.08 (d, J = 3.5 Hz, 1H), 2.66 (d, J = 12.6 Hz, 1H), 2.25 (t, J = 13.1 Hz, 1H), 1.62 (s, 3H), 1.53 (s, 2H), 1.27 (s, 3H), 1.20 (s, 2H), 1.08 (s, 3H), 1.00 (s, 3H), 0.94 (s, 3H). ^{13}C NMR (151 MHz, $CDCl_3$) δ 197.22, 196.39, 172.61, 169.96, 165.20, 124.14, 115.01, 114.40, 62.30, 47.94, 46.91, 46.38, 45.24, 43.23, 42.80, 41.93, 36.78, 34.62, 32.66, 31.93, 31.66, 30.50, 30.04, 27.16, 26.94, 25.49, 25.03, 24.05, 22.99, 21.95, 21.71, 18.32, 11.20, 8.03. HRMS (ESI) m/z: $[M + H]^+$ calcd for $C_{40}H_{57}N_4O_4$ 657.4374, found 657.4372.

^{1}H NMR (600 MHz, CDCl$_3$) of D5-1

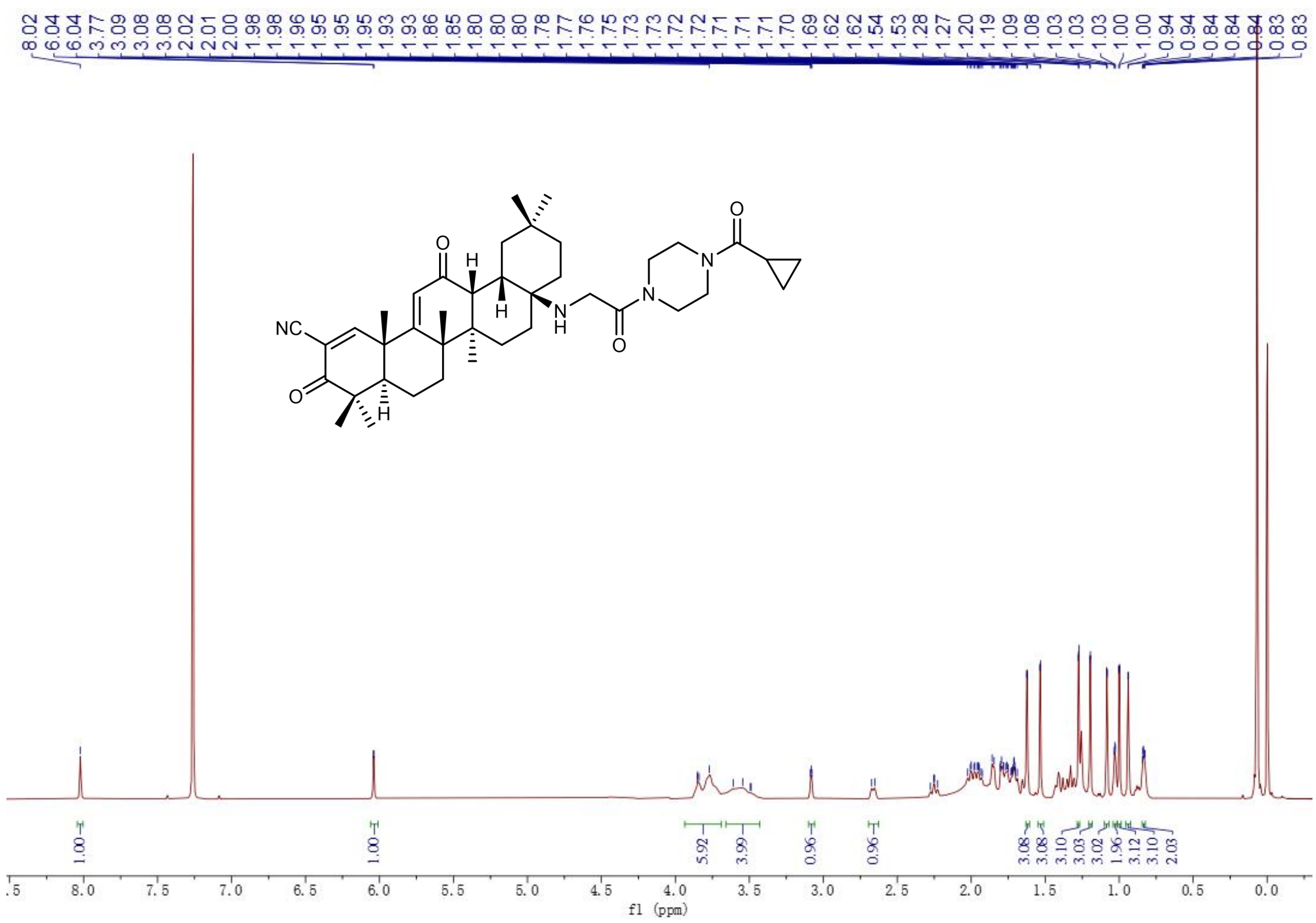

^{13}C NMR (151 MHz, CDCl$_3$) of D5-1

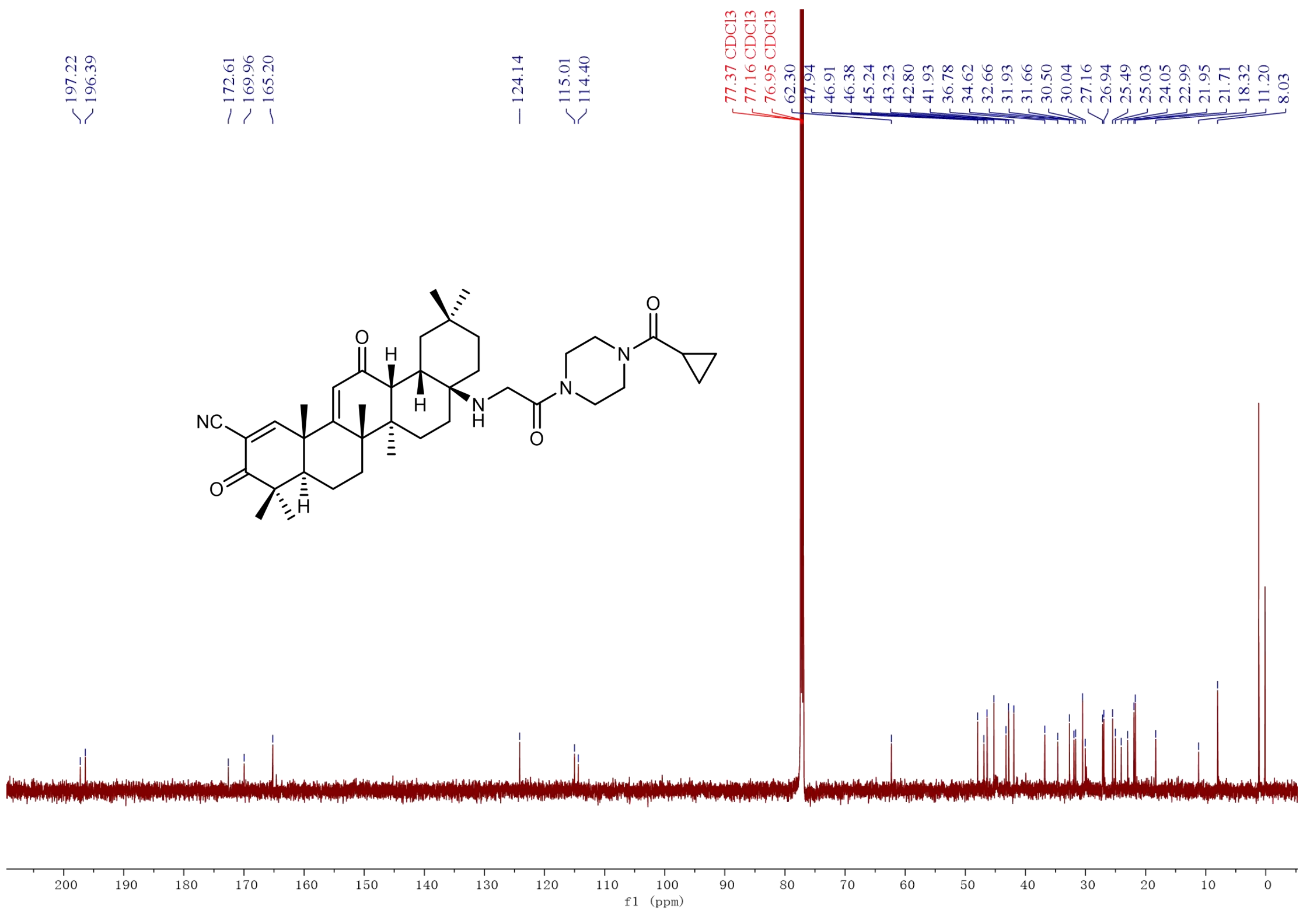

HPLC of D5-1

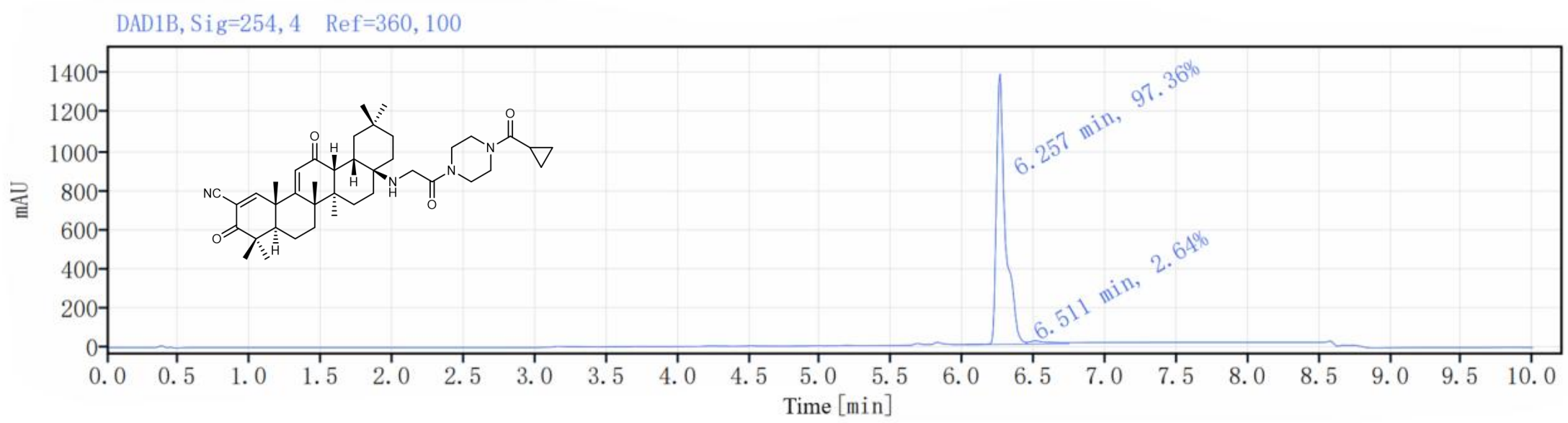

RT [min]	Signal Description	Width [min]	Sum Area	Height	Area%
6.257	DAD1B, Sig=254, 4 Ref=360, 100	0.427	6158.2	1382.3	97.36
6.511	DAD1B, Sig=254, 4 Ref=360, 100	0.299	167.2	15.9	2.64
		**Sum**	6325.4		

HRMS of D5-1

**Data Filename**	ESIH202503604.d	**Sample Name**	F1-D5-1
**Sample ID**		**Position**	P1-C4
**Instrument Name**	Agilent 6520 Q-TOF	**Acq Method**	20160322_MS_ESIH_POS_1min.m
**Acquired Time**	6/30/2025 2:35:07 PM	**DA Method**	ESI-HR-20231114.m
**Comment**	ESIH by fangsu		

User Spectra

Fragmentor Voltage	Collision Energy	Ionization Mode
170	0	ESI

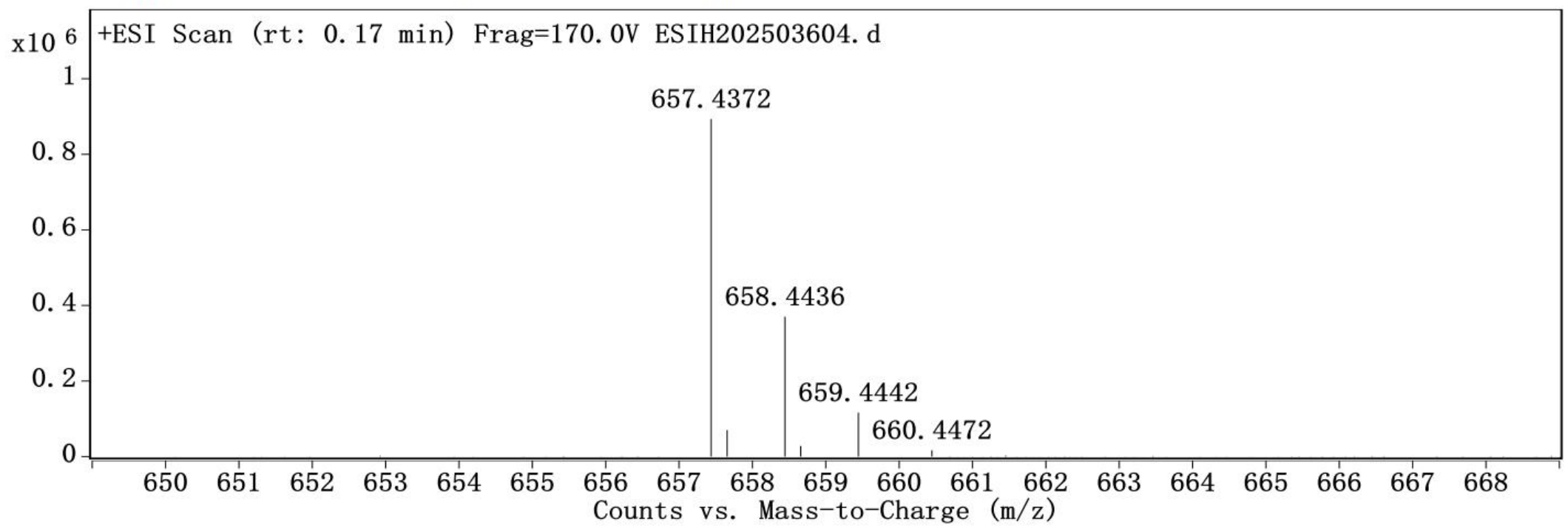

Formula Calculator Results

m/z	Calc m/z	Diff (mDa)	Diff (ppm)	Ion Formula	Ion
657.4372	657.4374	0.24	0.37	C40 H57 N4 O4	(M+H)+

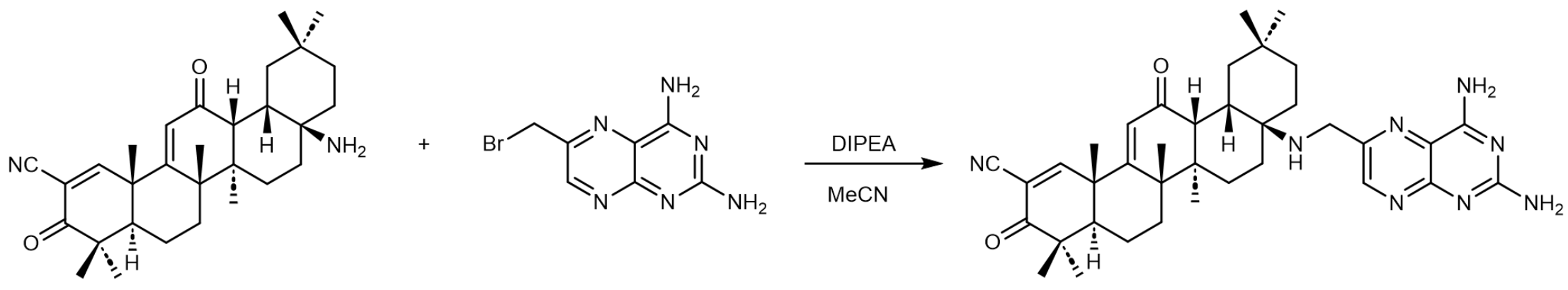

Supplementary Fig. 16 The synthetic route of D6-1.

Compound **3** (100 mg, 0.216 mmol) and 6-(Bromomethyl)-2,4-pteridinediamine (66.2 mg, 0.26 mmol) was dissolved in dry CH_3CN (2.2 mL). DIPEA (150.0 μL, 0.87 mmol) was added, and the mixture was stirred at 85 °C for 5 h. Then the mixture was concentrated under reduced pressure to afford the crude product. The crude material was purified by automated flash chromatography (DCM/MeOH = 10:1) and semi-preparative HPLC to afford the product **D6-1** (15.5 mg, 11%) as a white solid. ^{1}H NMR (600 MHz, CD_3OD) δ 8.95 (s, 1H), 8.42 (s, 1H), 6.24 (s, 1H), 4.70 (s, 2H), 2.87 (d, J = 13.0 Hz, 1H), 2.35 (dt, J = 18.8, 9.3 Hz, 1H), 2.15–2.10 (m, 1H), 1.55 (s, 3H), 1.53 (s, 3H), 1.25 (s, 3H), 1.18 (s, 3H), 1.15 (s, 3H), 1.07 (s, 3H), 0.97 (s, 3H). ^{13}C NMR (151 MHz, CD_3OD) δ 198.70, 172.50, 168.37, 164.51, 158.00, 151.75, 150.19, 147.84, 125.27, 122.84, 115.68, 115.22, 97.21, 47.51, 46.15, 44.18, 43.71, 43.01, 38.26, 35.76, 35.61, 32.85, 31.31, 30.74, 27.19, 26.16, 26.08, 26.05, 23.41, 21.98, 21.74, 19.07, 14.41. HRMS (ESI) m/z: $[M + H]^+$ calcd for $C_{37}H_{49}N_8O_2$ 637.3973, found 637.3971.

^{1}H NMR (600 MHz, CD_3OD) of D6-1

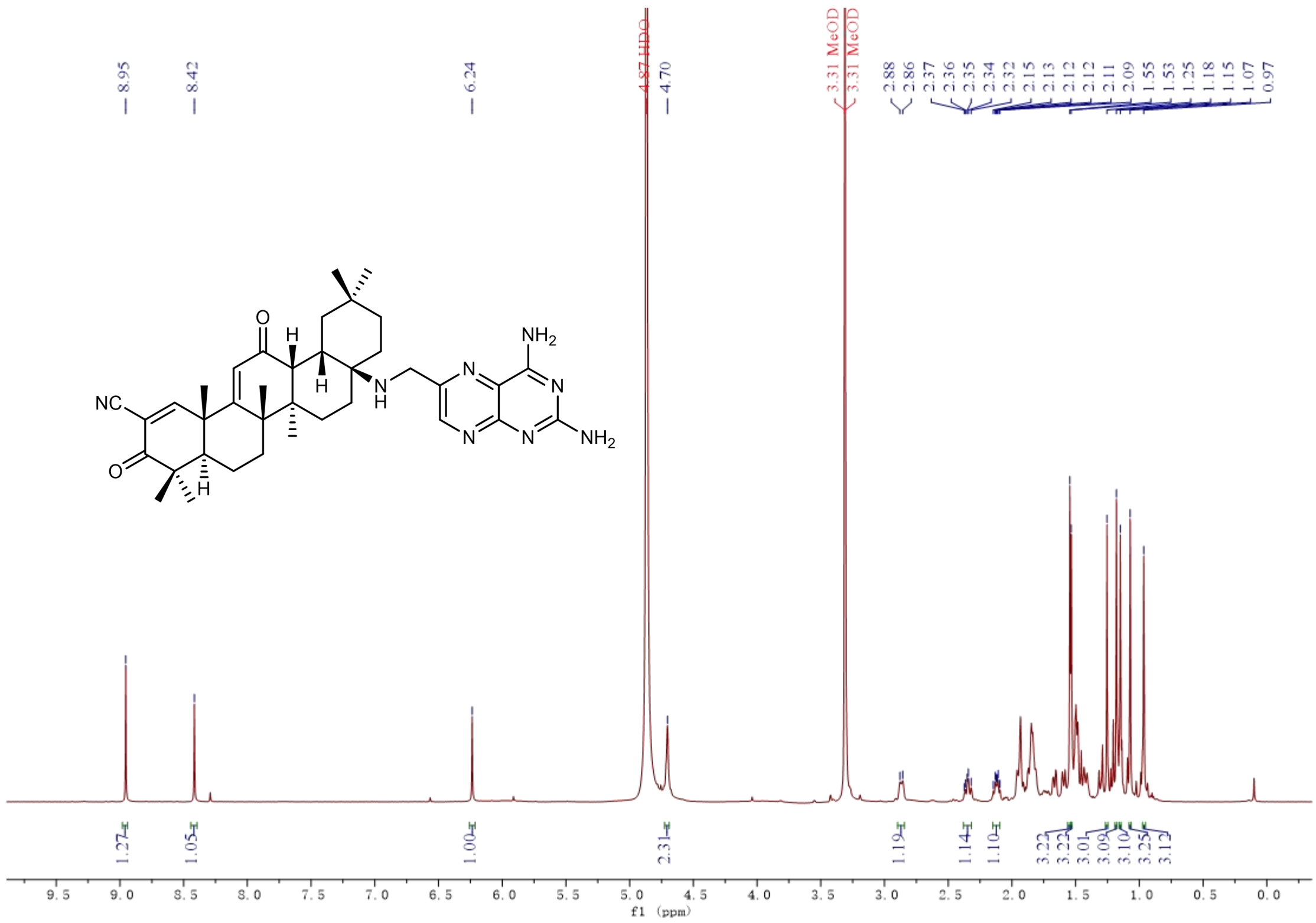

^{13}C NMR (151 MHz, CD_3OD) of D6-1

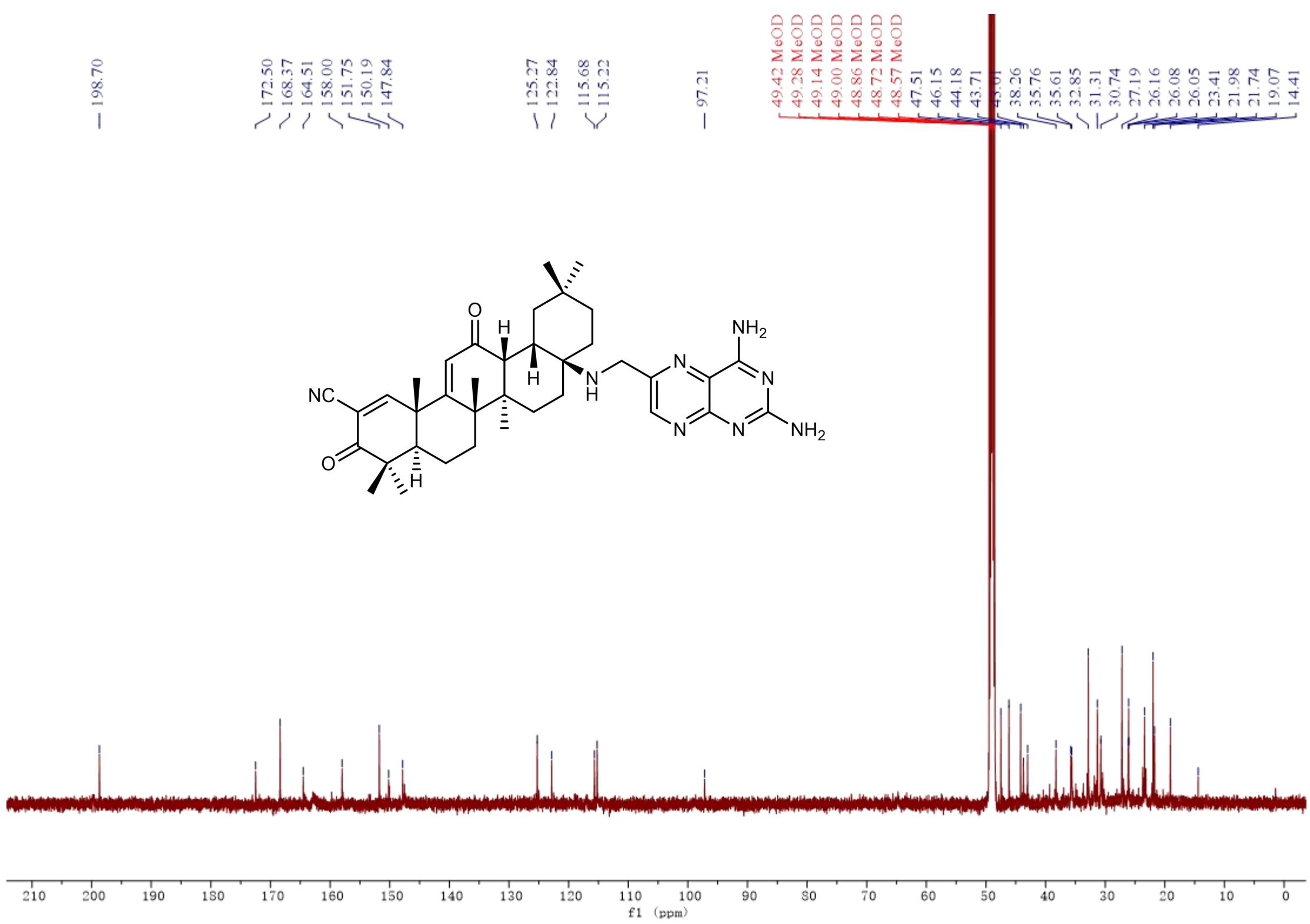

HPLC of D6-1

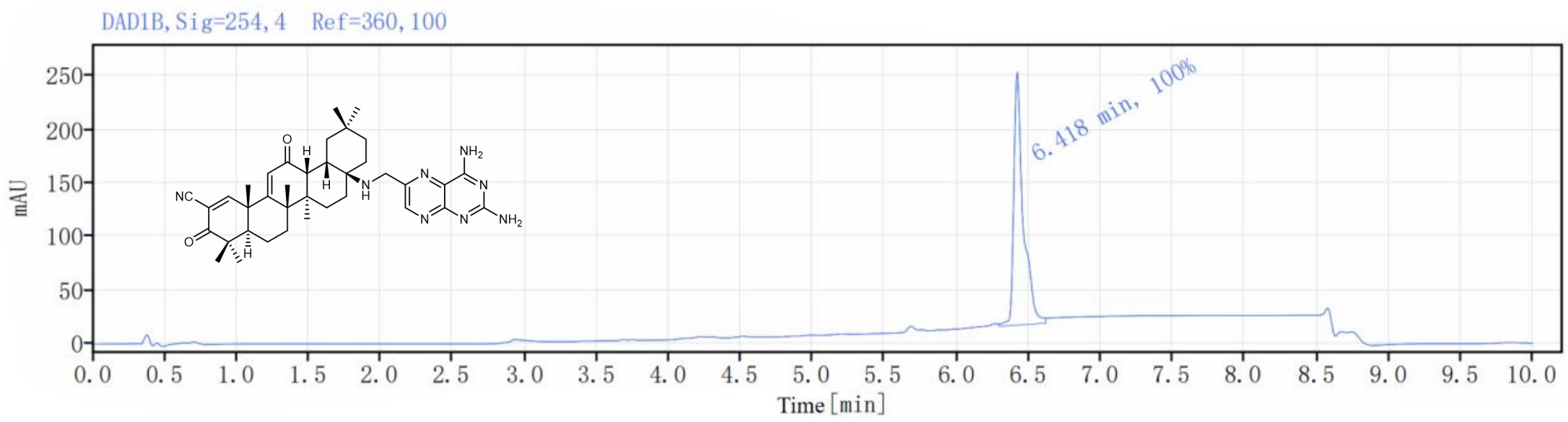

RT [min]	Signal Description	Width [min]	Sum Area	Height	Area%
6. 418	DAD1B, Sig=254, 4 Ref=360, 100	0. 325	1080. 0	236. 9	100. 00
		Sum	1080. 0		

HRMS of D6-1

**Data Filename**	ESIH202503605.d	**Sample Name**	F1-D6-1
**Sample ID**		**Position**	P1-C5
**Instrument Name**	Agilent 6520 Q-TOF	**Acq Method**	20160322_MS_ESIH_POS_1min.m
**Acquired Time**	6/30/2025 2:36:22 PM	**DA Method**	ESI-HR-20231114.m
**Comment**	ESIH by fangsu		

User Spectra

Fragmentor Voltage	Collision Energy	Ionization Mode
170	0	ESI

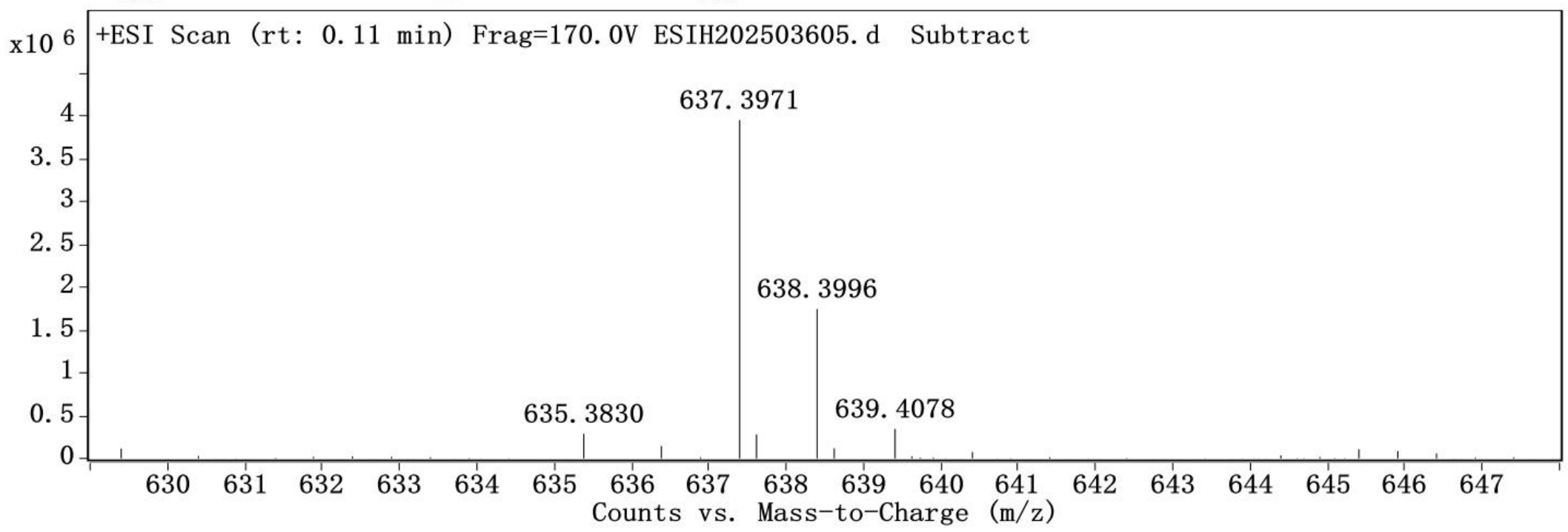

Formula Calculator Results

m/z	Calc m/z	Diff (mDa)	Diff (ppm)	Ion Formula	Ion
637.3971	637.3973	0.15	0.24	C37 H49 N8 O2	(M+H)+

The synthesis of D6-2

Compound **D1-1** (50.0 mg, 0.10 mmol) was dissolved in dry DMF (1.9 mL). HATU (80.3 mg, 0.21 mmol) was added, and the mixture was stirred at room temperature for 30 min. DIPEA (51 μL, 0.29 mmol) and 1-(cyclopropanecarbonyl)piperazine (13 μL, 0.11 mmol) were then added to the reaction mixture. The reaction was stirred at room temperature overnight. Upon completion, the mixture was diluted with water and extracted with EtOAc three times. The combined organic layers were washed with saturated brine, dried over anhydrous Na_2SO_4, and filtered. The filtrate was concentrated under reduced pressure to afford the crude product. The crude material was purified by automated flash chromatography (hexanes/EtOAc = 2:1) and semi-preparative HPLC to afford the product **D6-2** (9.0 mg, 15%) as a white solid. ^1H NMR (600 MHz, $CDCl_3$) δ 8.08 (s, 1H), 6.08 (s, 1H), 3.89 (t, *J* = 5.0 Hz, 2H), 3.48–3.42 (m, 2H), 3.23 (s, 3H), 3.01 (s, 2H), 2.84 (s, 4H), 2.75 (s, 2H), 2.22 (d, *J* = 14.9 Hz, 2H), 1.85–1.82 (m, 2H), 1.59 (s, 3H), 1.52 (s, 3H), 1.26 (s, 3H), 1.18 (s, 3H), 1.08 (s, 4H), 1.00 (s, 3H), 0.93 (s, 3H). ^{13}C NMR (151 MHz, $CDCl_3$) *δ* 196.83, 196.39, 165.21, 165.11, 124.16, 114.98, 114.43, 62.05, 52.51, 51.92, 47.92, 46.77, 46.30, 45.23, 43.67, 42.82, 42.34, 41.93, 40.55, 39.85, 36.82, 34.63, 32.65, 31.92, 30.57, 27.12, 26.89, 25.27, 25.06, 21.96, 21.71, 18.30. HRMS (ESI) m/z: $[M + H]^+$ calcd for $C_{37}H_{55}N_4O_3$ 603.4269, found 603.4272.

^{1}H NMR (600 MHz, $CDCl_3$) of D6-2

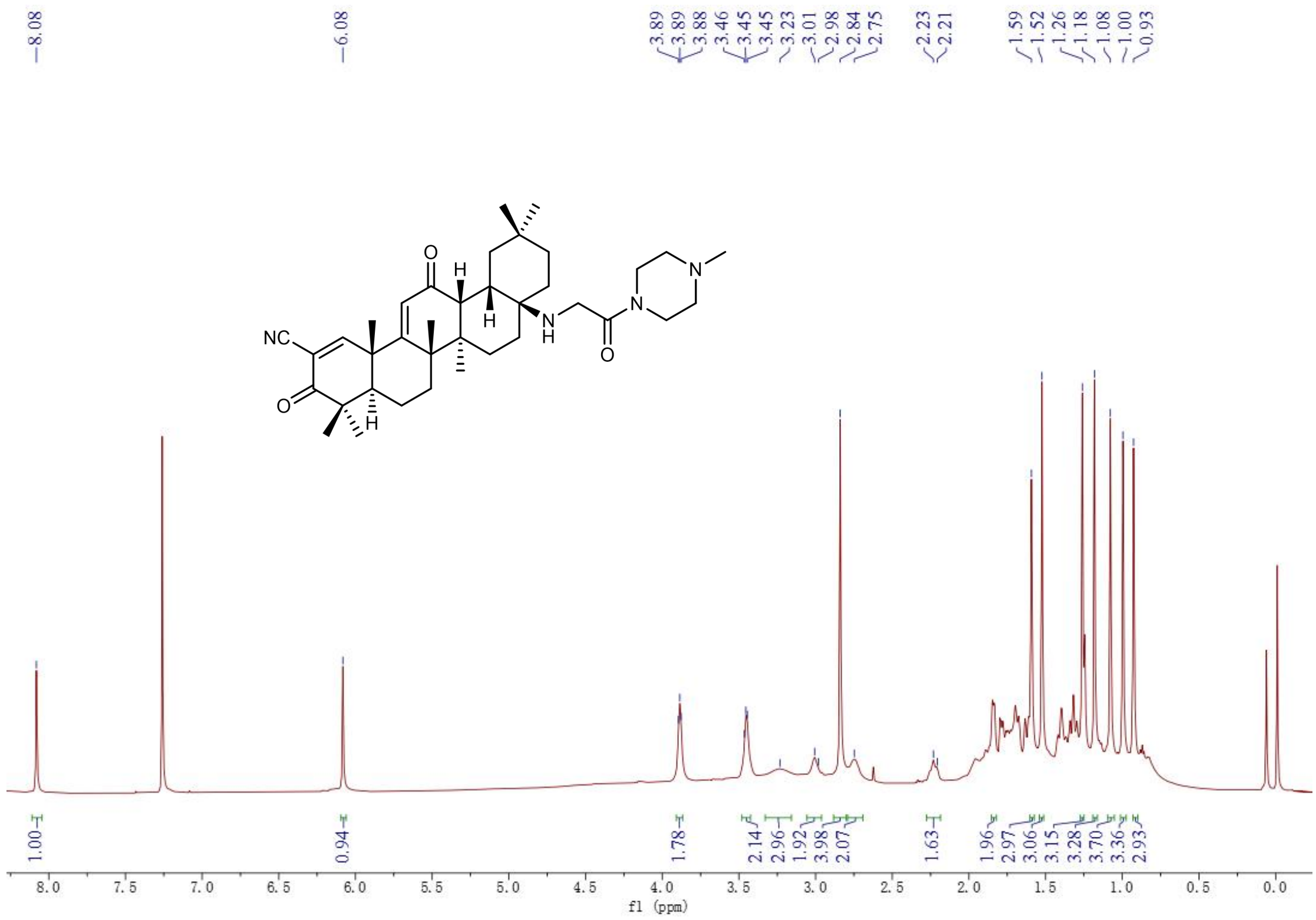

^{13}C NMR (151 MHz, $CDCl_3$) of D6-2

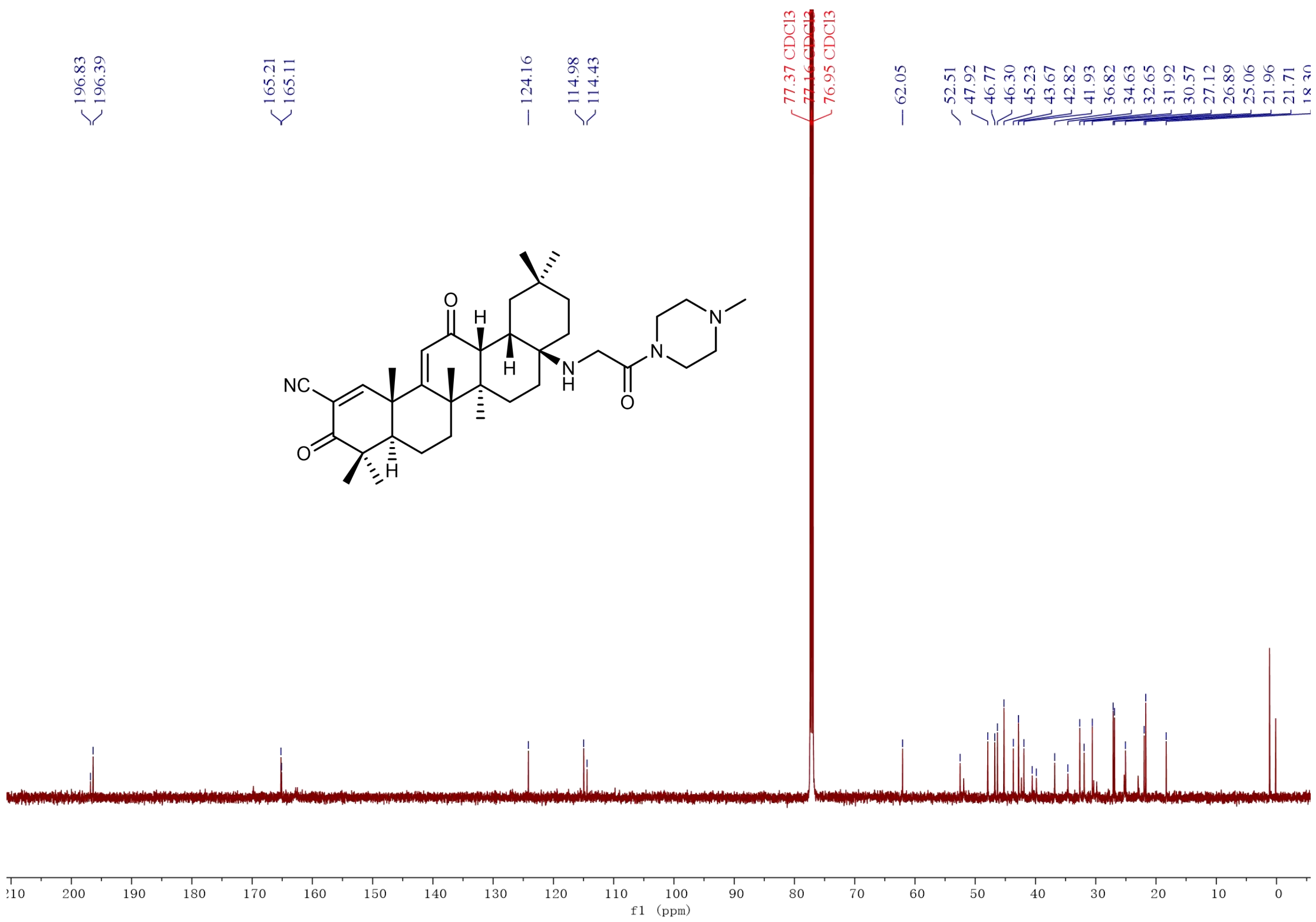

HPLC of D6-2

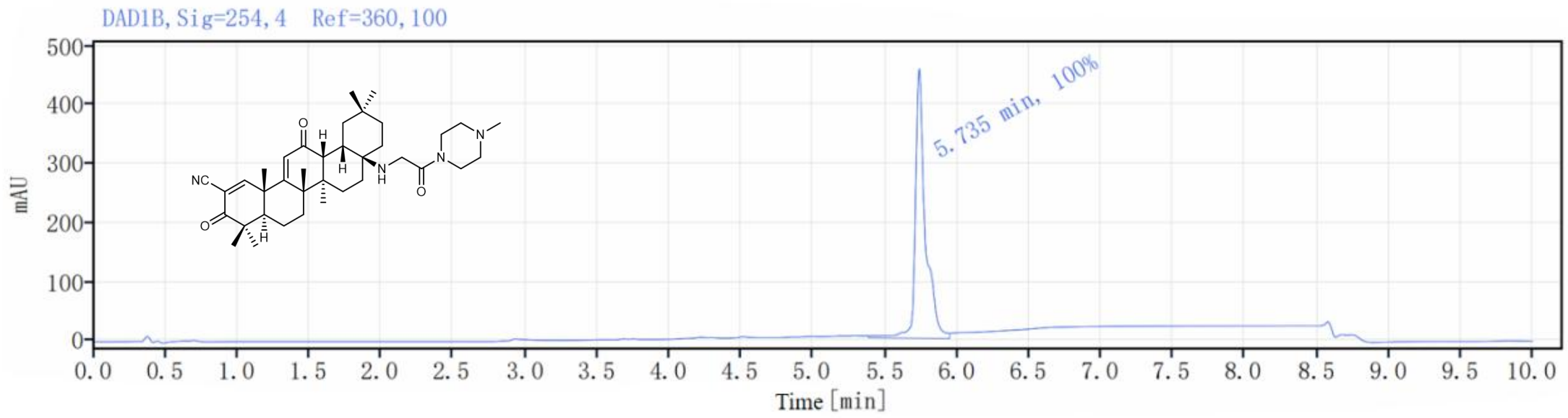

RT [min]	Signal Description	Width [min]	Sum Area	Height	Area%
5.735	DAD1B, Sig=254,4 Ref=360,100	0.565	2242.5	459.3	100.00
		Sum	2242.5		

HRMS of D6-2

**Data Filename**	ESIH202503606.d	**Sample Name**	F1-D6-2
**Sample ID**		**Position**	P1-C6
**Instrument Name**	Agilent 6520 Q-TOF	**Acq Method**	20160322_MS_ESIH_POS_1min.m
**Acquired Time**	6/30/2025 2:37:38 PM	**DA Method**	ESI-HR-20231114.m
**Comment**	ESIH by fangsu		

User Spectra

Fragmentor Voltage	Collision Energy	Ionization Mode
170	0	ESI

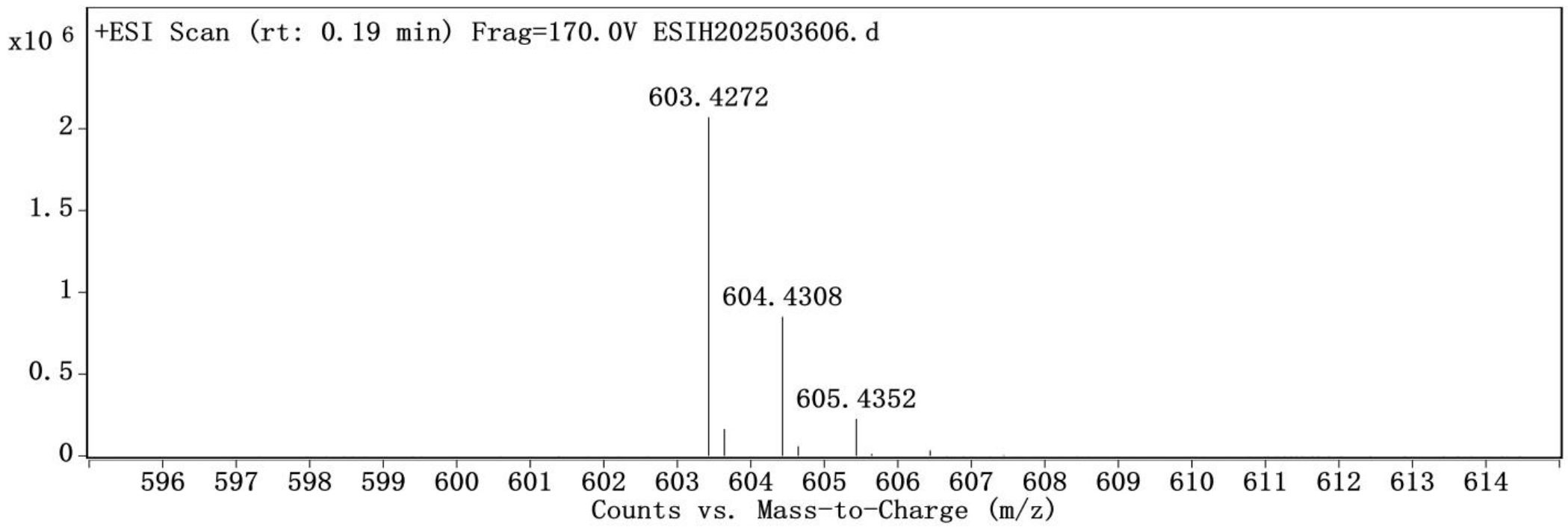

Formula Calculator Results

m/z	Calc m/z	Diff (mDa)	Diff (ppm)	Ion Formula	Ion
603.4272	603.4269	-0.33	-0.55	C37 H55 N4 O3	(M+H)+

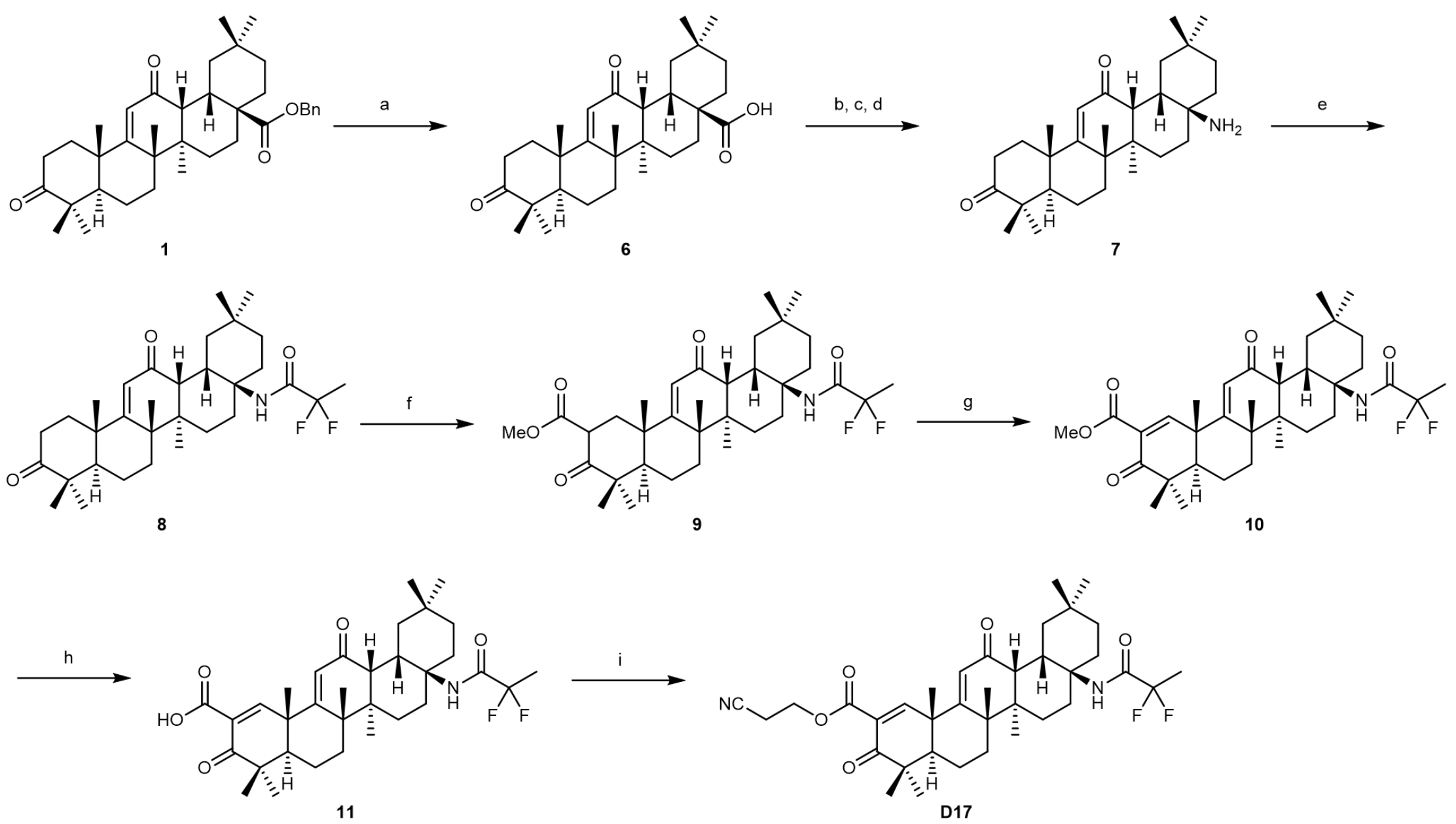

Supplementary Fig. 17 The synthetic route of D17.

Reagent and conditions: (a) H_2, 10% Pd/C, THF; (b) DPPA, Et_3N, Toluene; (c) Benzene, 80 °C; (d) MeCN, HCl, 10% NaOH; (e) CH_3CF_2COOH, DCC, DMAP, CH_2Cl_2; (f) NCCOOMe, LiHMDS, THF; (g) KHMDS, TMSCl, THF; $Pd(OAc)_2$, MeCN; (h) $(CH_3)_3SnOH$, $ClCH_2CH_2Cl$; (i) $NCCH_2CH_2OH$, EDCI, DMAP, DIPEA, CH_2Cl_2.

Preparation of compound 6. Compound **1** (3.3 g, 5.87 mmol) was dissolved in THF (58.7 mL). 10% wet Pd/C (375.0 mg, 3.52 mmol) was added, and the mixture was stirred at room temperature for 45 min. After completion, the mixture was filtered through celite. The filtrate was concentrated under reduced pressure and purified by flash chromatography (hexanes/EtOAc = 2:1) to obtain **6** (2.4 g, 88%) as a white solid.

Preparation of compound 7. Compound **6** (2.4 g, 5.16 mmol) and triethylamine (2.2 mL, 15.49 mmol) were dissolved in dry toluene (51.6 mL). DPPA (1.7 mL, 7.75 mmol) was added dropwise at 0 °C. After 15 min, the reaction mixture was warmed to room temperature and monitored by TLC. After completion, the mixture was purified by flash chromatography (hexanes/EtOAc = 4:1) to afford a white solid (2.3 g, 91%). The above solid (2.3 g) was dissolved in toluene (30.4 mL) and refluxed at 80 °C for 2 h. The solvent was removed by distillation under reduced pressure to afford

the crude product, which was used directly in the next step. The crude was dissolved in acetonitrile (25.7 mL). Concentrated HCl (12 N, 10.4 mL) was added dropwise at 0 ℃. The reaction mixture was then warmed to room temperature and stirred for 1 h. The mixture was cooled again to 0 ℃, and 10% NaOH solution (60.8 mL) was added dropwise with stirring. The mixture was then warmed to room temperature and stirred for 1 h. The reaction mixture was extracted with EtOAc. The organic layer was washed with water and saturated brine, dried over anhydrous Na_2SO_4, filtered, and concentrated under reduced pressure to afford the product **7** (2.0 g, 88%) as a white solid.

Preparation of compound 8. Compound **7** (2.0 g, 4.55 mmol) and 2,2-difluoropropionic acid (600.7 mg, 5.46 mmol) were dissolved in dry CH_2Cl_2 (45.5 mL). DCC (1.4 g, 6.82 mmol) and DMAP (277.9 mg, 2.27 mmol) were added. The reaction mixture was stirred at room temperature overnight. After completion, the mixture was purified by flash chromatography (hexanes/EtOAc = 3:1) to afford compound **8** (1.5 g, 61%) as a white solid.

Preparation of compound 9. Compound **8** (1.5 g, 2.76 mmol) was dissolved in dry THF (55.3 mL). LiHMDS (1.0 M in THF, 2.8 mL, 2.76 mmol) was added dropwise at -78 ℃ under a nitrogen atmosphere. The mixture was stirred at -78 ℃ for 1.5 h. Methyl cyanoformate (0.2 mL, 2.76 mmol) was then added at -78 ℃, and the reaction mixture was stirred at the same temperature for 1 h. After completion, the mixture was quenched with saturated NH_4Cl solution and extracted with CH_2Cl_2. The combined organic layers were washed with saturated brine, dried over anhydrous Na_2SO_4, filtered, and concentrated under reduced pressure. The residue was purified by flash chromatography (hexanes/EtOAc = 4:1) to afford compound **9** (304.0 mg, 19%) as a white solid.

Preparation of compound 10. Compound **9** (304.0 mg, 0.52 mmol) was dissolved in dry THF (5.2 mL). KHMDS (1.0 M in THF, 1.0 mL, 1.03 mmol) was added dropwise at 0 ℃ under a nitrogen atmosphere. The mixture was stirred at 0 ℃ for 10 min. Chlorotrimethylsilane (1.3 mL, 10.31 mmol) was then added at 0 ℃, and stirring was continued at this temperature for 20 min. After completion of the reaction, the mixture was quenched with water and extracted with EtOAc (3 times). The combined organic layers were washed with saturated brine, dried over anhydrous Na_2SO_4, filtered,

and concentrated under reduced pressure to afford the crude product. The crude product was dissolved in dry acetonitrile (5.2 mL). $Pd(OAc)_2$ (347.0 mg, 1.55 mmol) was added, and the mixture was stirred in an oil bath at 60 °C overnight. After completion, the mixture was concentrated under reduced pressure and purified by flash chromatography (hexanes/EtOAc = 3:1) to afford compound **10** (223.2 mg, 74%) as a white solid.

Preparation of compound 11. Compound **10** (223.2 mg, 0.38 mmol) was dissolved in dry 1,2-dichloroethane (3.8 mL). Trimethyltin hydroxide (343.3 mg, 1.90 mmol) was added, and the mixture was stirred in an oil bath at 75 °C for 8 h. After completion of the reaction, the mixture was concentrated under reduced pressure and purified by flash chromatography (hexanes/EtOAc = 1:1) to afford compound **11** (111.6 mg, 51%) as a white solid.

The synthesis of D17

Compound **11** (60.0 mg, 0.10 mmol) was dissolved in dry CH_2Cl_2 (1.1 mL). EDCI (40.1 mg, 0.21 mmol), DMAP (12.8 mg, 0.10 mmol), 3-hydroxypropionitrile (9 μL, 0.13 mmol), and DIPEA (52 μL, 0.31 mmol) were added. The reaction mixture was stirred at room temperature overnight. After completion of the reaction, the solvent was removed under reduced pressure. The residue was purified by flash chromatography (hexanes/EtOAc = 2:1) to afford compound **D17** (12.0 mg, 18%) as a white solid. ^{1}H NMR (600 MHz, $CDCl_3$) δ 8.09 (s, 1H), 6.20 (s, 1H), 5.99 (d, J = 3.0 Hz, 1H), 4.45–4.39 (m, 2H), 3.06 (d, J = 4.7 Hz, 1H), 2.82–2.78 (m, 3H), 2.11 (dt, J = 16.5, 2.9 Hz, 1H), 1.80 (t, J = 19.4 Hz, 3H), 1.42 (s, 3H), 1.41 (s, 3H), 1.21 (s, 3H), 1.20 (s, 3H), 1.07 (s, 3H), 1.05 (s, 3H), 0.91 (s, 3H); ^{13}C NMR (151 MHz, $CDCl_3$) δ 200.26, 199.36, 171.89, 163.98, 161.12, 129.39, 124.96, 117.64, 59.65, 57.68, 48.21, 47.77, 46.10, 46.01, 42.29, 42.10, 36.93, 35.46, 35.28, 33.08, 33.00, 31.61, 30.83, 28.00, 27.19, 26.14, 24.36, 23.40, 22.79, 21.81, 21.35, 20.78, 20.61, 20.44, 18.71, 18.03. HRMS (ESI) m/z: $[M + H]^+$ calcd for $C_{36}H_{49}F_2N_2O_5$ 627.3604, found 627.3604.

^{1}H NMR (600 MHz, $CDCl_3$) of D17

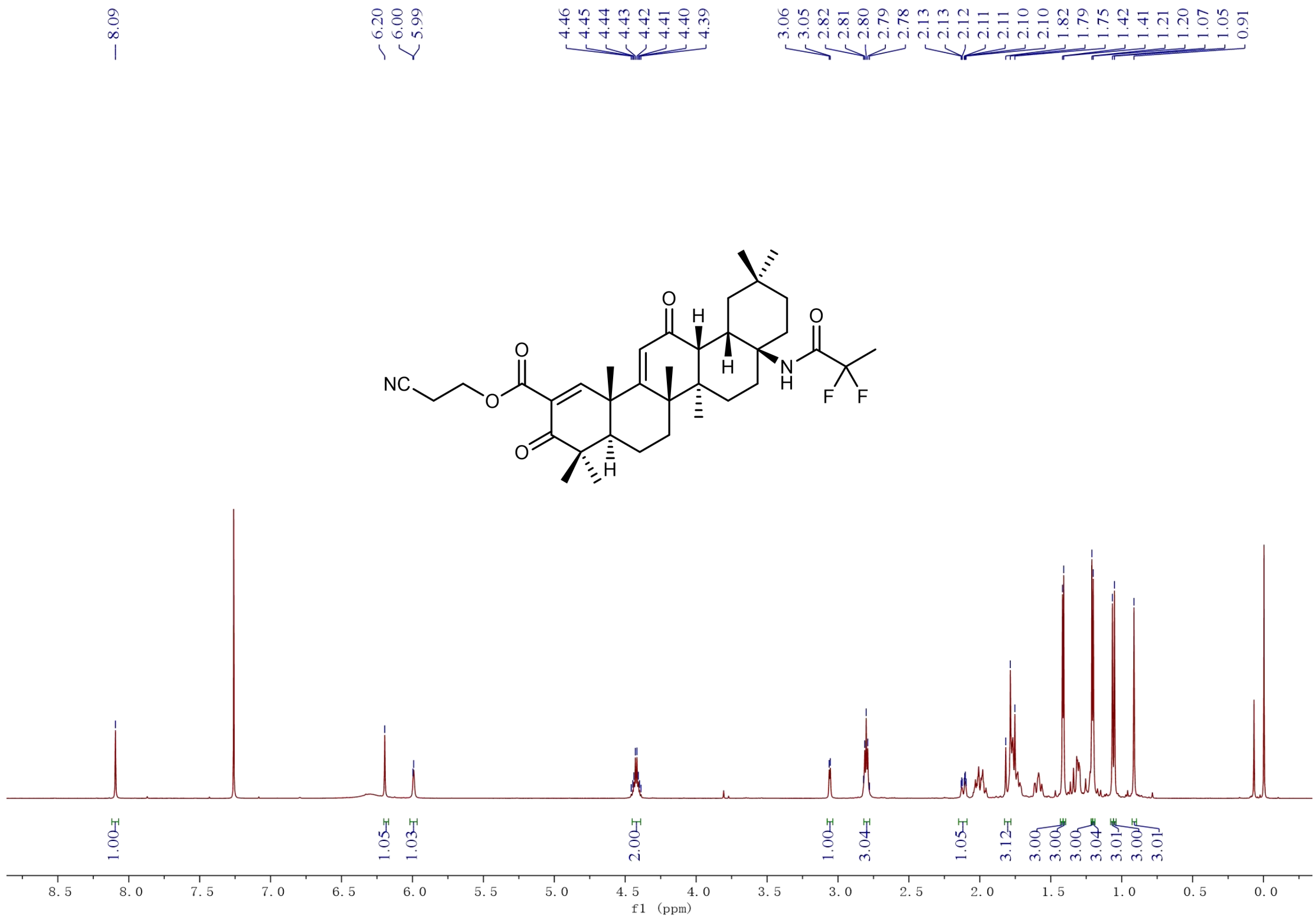

^{13}C NMR (151 MHz, $CDCl_3$) of D17

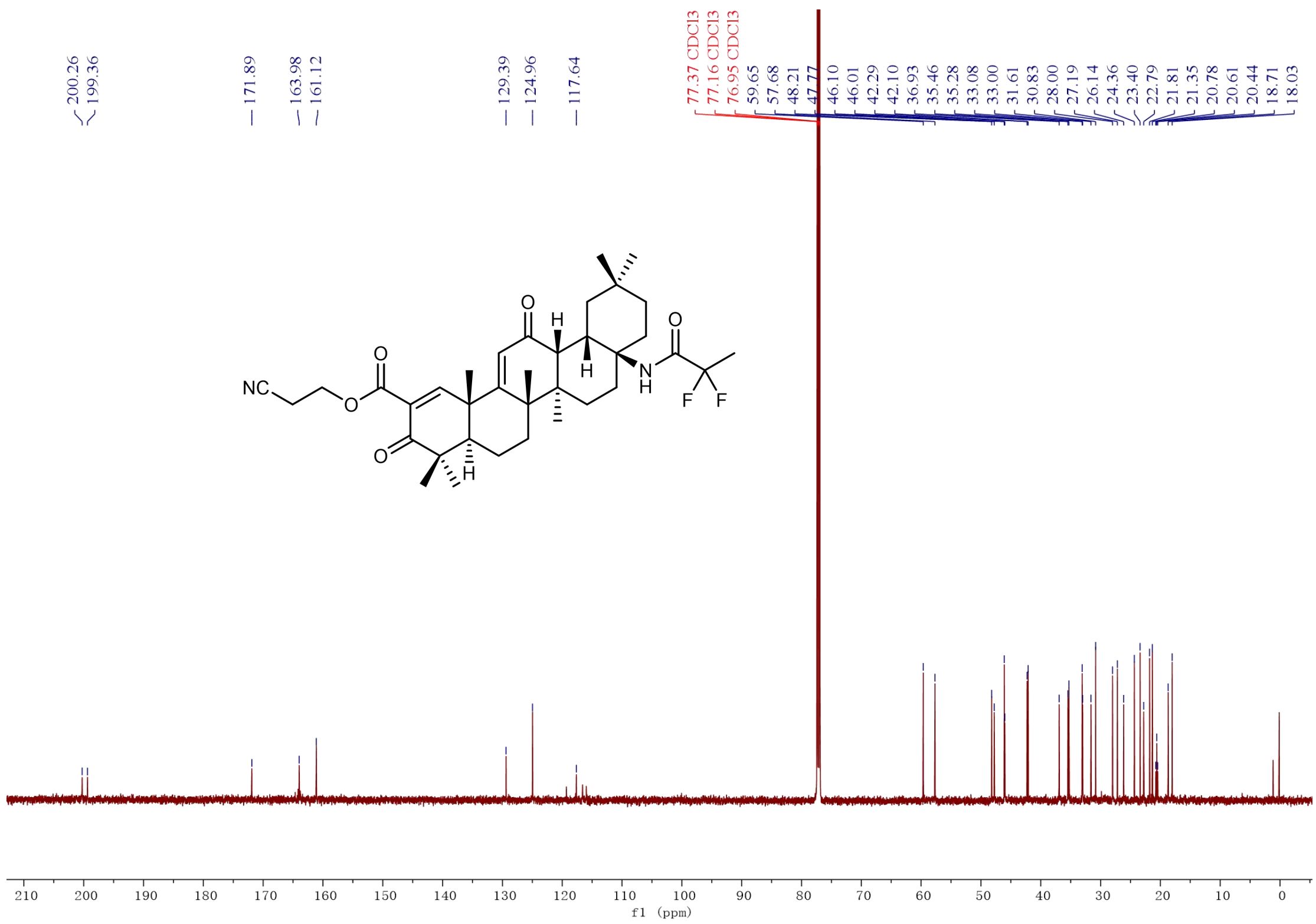

HPLC of D17

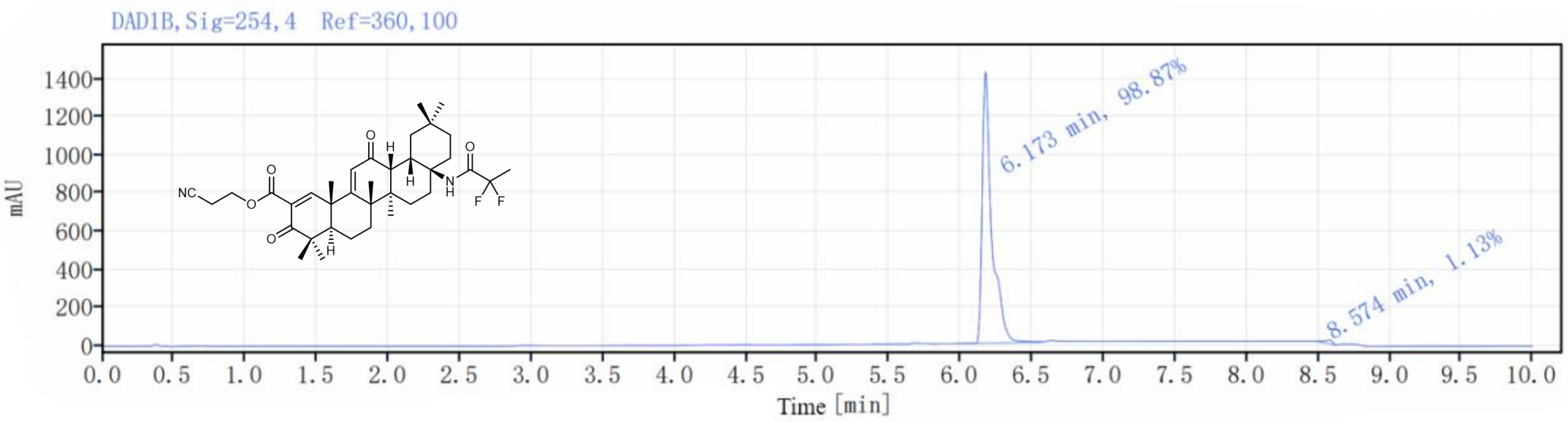

RT [min]	Signal Description	Width [min]	Sum Area	Height	Area%
6.173	DAD1B, Sig=254, 4 Ref=360, 100	0.630	6727.3	1429.2	98.87
8.574	DAD1B, Sig=254, 4 Ref=360, 100	0.147	76.7	19.8	1.13
		Sum	6804.0		

HRMS of D17

**Data Filename**	ESIH202503607.d	**Sample Name**	F1-D17
**Sample ID**		**Position**	P1-C7
**Instrument Name**	Agilent 6520 Q-TOF	**Acq Method**	20160322_MS_ESIH_POS_1min.m
**Acquired Time**	6/30/2025 2:38:54 PM	**DA Method**	ESI-HR-20231114.m
**Comment**	ESIH by fangsu		

User Spectra

Fragmentor Voltage	Collision Energy	Ionization Mode
170	0	ESI

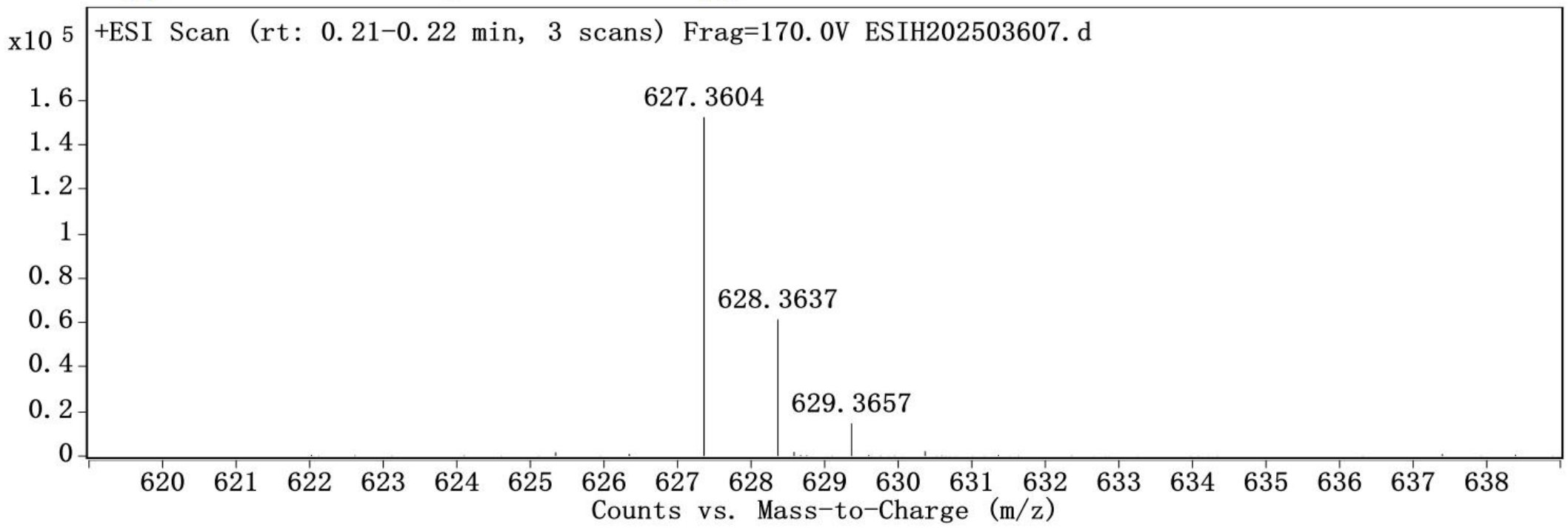

Formula Calculator Results

m/z	Calc m/z	Diff (mDa)	Diff (ppm)	Ion Formula	Ion
627.3604	627.3604	-0.04	-0.07	C36 H49 F2 N2 O5	(M+H)+

Supplementary references

1. Ai, Y. *et al.* Synthesis and Biological Evaluation of Novel Olean-28,13beta-lactams as Potential Antiprostate Cancer Agents. *J Med Chem* **58**, 4506-4520 (2015).
