# Supplementary material for: Omaveloxolone functions as a molecular crowbar-like degrader of STING to alleviate aging-related inflammation
Source: Nat Commun. 2026 Sep 17;17:11202. doi: 10.1038/s41467-026-77760-1 (PMC13719992; doi:10.1038/s41467-026-77760-1)
Supplement: Supplementary file 2 [file 41467_2026_77760_MOESM2_ESM.pdf]

Descriptions of Additional Supplementary Files

Supplementary Data 1

A list of sgRNA sequences and qPCR primers used in the study.

Supplementary Data 2

A list of antibodies used in the study.

Supplementary Data 3

Systems for MD simulation.

Supplementary Data 4

A summary table of HDX-MS experimental parameters.
