# Supplementary material for: Omaveloxolone functions as a molecular crowbar-like degrader of STING to alleviate aging-related inflammation
Source: Nat Commun. 2026 Sep 17;17:11202. doi: 10.1038/s41467-026-77760-1 (PMC13719992; doi:10.1038/s41467-026-77760-1)
Supplement: Supplementary file 3 [file 41467_2026_77760_MOESM7_ESM.pdf]

nature portfolio

Corresponding author(s): Sulin Zhang, Mingyue Zheng, Dan Teng

Last updated by author(s): Jul 31, 2026

Reporting Summary

Nature Portfolio wishes to improve the reproducibility of the work that we publish. This form provides structure for consistency and transparency in reporting. For further information on Nature Portfolio policies, see our Editorial Policies and the Editorial Policy Checklist.

Statistics

For all statistical analyses, confirm that the following items are present in the figure legend, table legend, main text, or Methods section.

n/a	Confirmed	
[ ]	[x]	The exact sample size (*n*) for each experimental group/condition, given as a discrete number and unit of measurement
[ ]	[x]	A statement on whether measurements were taken from distinct samples or whether the same sample was measured repeatedly
[ ]	[x]	The statistical test(s) used AND whether they are one- or two-sided<br>*Only common tests should be described solely by name; describe more complex techniques in the Methods section.*
[x]	[ ]	A description of all covariates tested
[x]	[ ]	A description of any assumptions or corrections, such as tests of normality and adjustment for multiple comparisons
[ ]	[x]	A full description of the statistical parameters including central tendency (e.g. means) or other basic estimates (e.g. regression coefficient) AND variation (e.g. standard deviation) or associated estimates of uncertainty (e.g. confidence intervals)
[ ]	[x]	For null hypothesis testing, the test statistic (e.g. *F*, *t*, *r*) with confidence intervals, effect sizes, degrees of freedom and *P* value noted<br>*Give P values as exact values whenever suitable.*
[x]	[ ]	For Bayesian analysis, information on the choice of priors and Markov chain Monte Carlo settings
[x]	[ ]	For hierarchical and complex designs, identification of the appropriate level for tests and full reporting of outcomes
[x]	[ ]	Estimates of effect sizes (e.g. Cohen's *d*, Pearson's *r*), indicating how they were calculated

Our web collection on statistics for biologists contains articles on many of the points above.

Software and code

Policy information about availability of computer code

Data collection	Experimental data were collected using softwares from the following instruments: NovoCyte 3000 flow cytometer (Agilent); GeneGnome XRQ system (Syngene); Tecan Spark multifuntional microplate reader; PerkinElmer EnVision multifunctional microplate reader; QuantStudio 5 Real-Time PCR System (Applied Biosystems); CFX96 Real-Time PCR Detection System (Bio-Rad); MicroCal PEAQ-ITC (Malvern); Monolith NT.115 instrument (Nano Temper).
Data analysis	GraphPad Prism v8.0.1 was used to perform general statistical analyses. Flow cytometry data were analyzed with FlowJo v10.6.2. The quantitative analysis of western blot images were performed by ImageJ v1.53q. Molecular docking was performed using the Glide module in the Schrödinger 2023 software suite. Molecular dynamics simulations were performed using GROMACS 2022.4. Please see Methods section in manuscript for further details.

For manuscripts utilizing custom algorithms or software that are central to the research but not yet described in published literature, software must be made available to editors and reviewers. We strongly encourage code deposition in a community repository (e.g. GitHub). See the Nature Portfolio guidelines for submitting code & software for further information.

Data

Policy information about availability of data

All manuscripts must include a data availability statement. This statement should provide the following information, where applicable:

- Accession codes, unique identifiers, or web links for publicly available datasets
- A description of any restrictions on data availability
- For clinical datasets or third party data, please ensure that the statement adheres to our policy

All data are available in the main text or the supplementary materials. The RNA-seq data generated in this study have been deposited in the Genome Sequence Archive (GSA) database under accession code HRA015016 (https://ngdc.cncb.ac.cn/search/specific?db=hra&q=HRA015016). The proteomic data generated in this study have been deposited in a ProteomeXchange partner repository under accession code PXD071456 (https://www.iprox.cn//page/SCV017.html?query=PXD071456). Source data are provided with this paper.

Research involving human participants, their data, or biological material

Policy information about studies with human participants or human data. See also policy information about sex, gender (identity/presentation), and sexual orientation and race, ethnicity and racism.

Reporting on sex and gender	n/a
Reporting on race, ethnicity, or other socially relevant groupings	n/a
Population characteristics	n/a
Recruitment	n/a
Ethics oversight	n/a

Note that full information on the approval of the study protocol must also be provided in the manuscript.

Field-specific reporting

Please select the one below that is the best fit for your research. If you are not sure, read the appropriate sections before making your selection.

[x] Life sciences [ ] Behavioural & social sciences [ ] Ecological, evolutionary & environmental sciences

For a reference copy of the document with all sections, see nature.com/documents/nr-reporting-summary-flat.pdf

Life sciences study design

All studies must disclose on these points even when the disclosure is negative.

Sample size	No statistical method was used to predetermine sample size. The sample sizes were determined to ensure experimental reproducibility based on previous experience and published literature. Group sizes for in vivo experiments were selected empirically based upon prior knowledge, with 5-7 mice per group being a commonly used range in similar studies employing the same models.
Data exclusions	No data were excluded from the analyses.
Replication	All experimental findings were reliably reproduced. Details of experimental replicates are provided in methods and figure legends.
Randomization	All experimental samples or models including in vitro and in vivo mice were randomly allocated to each group.
Blinding	Samples were marked by one scientist and were blinded to another scientist for analysis. The investigators were blinded to group allocation during data collection.

Reporting for specific materials, systems and methods

We require information from authors about some types of materials, experimental systems and methods used in many studies. Here, indicate whether each material, system or method listed is relevant to your study. If you are not sure if a list item applies to your research, read the appropriate section before selecting a response.

Materials & experimental systems

n/a	Involved in the study	
	x	Antibodies
	x	Eukaryotic cell lines
x		Palaeontology and archaeology
	x	Animals and other organisms
x		Clinical data
x		Dual use research of concern
x		Plants

Methods

n/a	Involved in the study	
x		ChIP-seq
	x	Flow cytometry
x		MRI-based neuroimaging

Antibodies

Antibodies used

anti-p-STING (Ser366) (Cell Signaling Technology, 50907, Monoclonal (E9A9K), 1:1000);
anti-p-STING (Ser365) (Cell Signaling Technology, 72971,Monoclonal (D8F4W), 1:1000);
anti-STING (Cell Signaling Technology, 13647, Monoclonal (D2P2F), 1:1000);
anti-p-TBK1 (Ser172) (Cell Signaling Technology, 5483, Monoclonal (D52C2), 1:1000);
anti-TBK1 (Cell Signaling Technology, 3504, Monoclonal (D1B4), 1:1000);
anti-p-IRF3 (Ser396) (Cell Signaling Technology, 29047, Monoclonal (D6O1M), 1:1000);
anti-IRF3 (Cell Signaling Technology, 11904, Monoclonal (D6I4C), 1:1000);
anti-NFκB p-p65 (Ser536) (Cell Signaling Technology, 3033, Monoclonal (93H1), 1:1000);
anti-NFκB p65 (Cell Signaling Technology, 8242, Monoclonal (D14E12), 1:1000);
anti-p-STAT3 (Tyr705) (Cell Signaling Technology, 9145, Monoclonal (D3A7), 1:2000);
anti-STAT3 (Cell Signaling Technology, 12640, Monoclonal (D3Z2G), 1:1000);
anti-NRF2 (Cell Signaling Technology, 12721, Monoclonal (D1Z9C), 1:1000);
anti-HA-Tag (Cell Signaling Technology, 3724, Monoclonal (C29F4), 1:1000);
anti-Flag-Tag (Cell Signaling Technology, 2368, Polyclonal, 1:1000);
anti-Myc-Tag (Cell Signaling Technology, 2278, Monoclonal (71D10), 1:1000);
anti-K27-linkage specific ubiquitin (Abcam, ab181537, Monoclonal (EPR17034), 1:5000);
anti-β-Actin (Cell Signaling Technology, 4970, Monoclonal (13E5), 1:1000);
anti-GAPDH (Cell Signaling Technology, 2118, Monoclonal (14C10), 1:1000);
anti-β-Tubulin (Starter, S0B0238, Monoclonal (SDT-312-113), 1:5000);
anti-HUWE1 (Cell Signaling Technology, 5695, Monoclonal (AX8D1), 1:1000);
anti-TRIM25 (Cell Signaling Technology, 13773, Monoclonal (D9T7G), 1:1000);
anti-MID1 (Immunoway, YT2760, Polyclonal, 1:1000);
anti-RNF219 (Proteintech, 24988-1-AP, Polyclonal, 1:1000);
HRP-conjugeted anti-rabbit igG (Promega, W4011, Polyclonal, 1:10000);
HRP-conjugated anti-mouse IgG (Promega, W4021, Polyclonal, 1:10000).

Validation

The antibodies were validated by the manufacturers. Validation data are available on the manufacturers' websites:
https://www.cellsignal.cn/
https://www.abcam.cn/
https://en.starter-bio.com/
https://www.immunoway.com/
https://www.ptgcn.com/
https://www.promega.com.cn/

Eukaryotic cell lines

Policy information about cell lines and Sex and Gender in Research

Cell line source(s)

THP-1 human myeloid leukemia mononuclear cells (TIB-202) were obtained from American Type Culture Collection. RAW 264.7 mouse macrophages cells (SCSP-5036) were obtained from Cell Bank of Chinese Academy of Sciences. HEK293T human embryonic kidney cells (CL-0005) and L929 mouse fibroblast cells (CL-0137) were obtained from Procell Life Science & Technology Co., Ltd. The cell lines THP-1-Blue ISG (thp-isg), THP-1-KO-STING (thpd-kostg), RAW-Lucia ISG (rawl-isg), RAW-KO-STING (rawl-kostg) were purchased from InvivoGen.

Authentication

None of the cell lines used were authenticated.

Mycoplasma contamination

There is no mycoplasma contamination in all cell lines.

Commonly misidentified lines
(See ICLAC register)

None used

Animals and other research organisms

Policy information about studies involving animals; ARRIVE guidelines recommended for reporting animal research, and Sex and Gender in Research

Laboratory animals	Wild-type (WT) C57BL/6 mice were purchased from Beijing Huafukang Biotechnology Co., Ltd. Trex1+/– C57BL/6 mice were kindly provided by Prof. Yichuan Xiao's laboratory at Shanghai Institute of Nutrition and Health, Chinese Academy of Sciences. Trex1–/– C57BL/6 mice were generated by further mating the male and female Trex1+/– mice. Wild-type C57BL/6 mice (6-10 weeks old) and Trex1-/- mice (4-7 weeks old) were used in this study. Aged mice were approximately 25 months old at the time of sample collection.
Wild animals	The study did not involve wild animals.
Reporting on sex	Male mice were selected for aging mouse model study as referenced to previously published aging-related studies. For the Trex1-/- mouse experiments, both male and female 4-7-week-old mice were included; however, sex-based analysis was not conducted, as sex was not incorporated as an experimental variable in the original study design.
Field-collected samples	The study did not involve samples collected from the field.
Ethics oversight	All animal studies were conducted in compliance with the guidelines approved by the Institutional Animal Care and Use Committees (IACUC) of Shanghai Institute of Materia Medica, Chinese Academy of Sciences (IACUC Issue NO. 2025-04-ZMY-12).

Note that full information on the approval of the study protocol must also be provided in the manuscript.

Plants

Seed stocks	n/a
Novel plant genotypes	n/a
Authentication	n/a

Flow Cytometry

Plots

Confirm that:

- [x] The axis labels state the marker and fluorochrome used (e.g. CD4-FITC).
- [x] The axis scales are clearly visible. Include numbers along axes only for bottom left plot of group (a 'group' is an analysis of identical markers).
- [x] All plots are contour plots with outliers or pseudocolor plots.
- [x] A numerical value for number of cells or percentage (with statistics) is provided.

Methodology

Sample preparation	THP-1 cells stably expressing EGFP-STING-IRES-mCherry were treated with indicated compounds for 12 h. After treatment, cells were washed and resuspended with PBS. Prior to analysis, cell suspensions were filtered through a 40 µm nylon mesh.
Instrument	NovoCyte 3000 flow cytometer (Agilent)
Software	FlowJo v10.6.2
Cell population abundance	For more accurate quantification, live single cells were gated based on FSC-A vs. SSC-A and FSC-H vs. FSC-A plot. mCherry-positive cells were then identified via PE-Texas Red-A fluorescence, and the EGFP signal (detected in the FITC-A channel) was quantified to evaluate STING protein abundance.
Gating strategy	Live cells were initially gated using a FSC-A vs. SSC-A plot. From the live cell population, single cells were further selected by gating on FSC-A vs. FSC-H. mCherry-positive cells were subsequently identified based on PE-Texas Red-A fluorescence. Finally, EGFP-STING signal intensity was quantified within the mCherry positive population using fluorescence intensity of FITC-A.

- [x] Tick this box to confirm that a figure exemplifying the gating strategy is provided in the Supplementary Information.
