# Supplementary material for: Omaveloxolone functions as a molecular crowbar-like degrader of STING to alleviate aging-related inflammation
Source: Nat Commun. 2026 Sep 17;17:11202. doi: 10.1038/s41467-026-77760-1 (PMC13719992; doi:10.1038/s41467-026-77760-1)
Supplement: Supplementary file 4 [file 41467_2026_77760_MOESM8_ESM.pdf]

nature portfolio

Peer Review File

Omaveloxolone functions as a molecular crowbar-like degrader of STING to alleviate aging-related inflammation

Corresponding Author: Professor Sulin Zhang

This file contains all reviewer reports in order by version, followed by all author rebuttals in order by version.

Version 0:

Reviewer comments:

Reviewer #1

(Remarks to the Author)
In this study, Zhou et. al identified NRF2 activator omaveloxolone as a strong binder and degrader of hSTING. They use molecular dynamics and biophysical analyses to conclude that omaveloxolone destabilization occurs through a "molecular crowbar" mechanism whereby loosening of the structural integrity increases HUWE1 binding affinity and in turn, degradation by the HUWE1-UbcH5b-proteasome axis. The authors also found degradation of hSTING by omaveloxolone suppresses its downstream inflammatory signaling and ameliorates aging-related inflammatory phenotypes, including in mice. This study will be of great interest to researchers in TPD and aging-related inflammation areas of research and has clinical relevance. This is an outstanding study with some concerns listed below.
It's confusing to mention hSTING LBD and CTD in the "Omaveloxolone binds non-covalently to STING LBD with high affinity and reduces STING protein thermal stability" section. Based on the written method, only the CTD of hSTING (residues 139-379) containing LBD and C-terminal domains was expressed and purified. It'd be better to include a schematic representation of human STING domains and clarify which construct is used for the experiment in the Figure 3, as done for Figure 5g. Otherwise, it's hard to know whether hSTING full length, LBD domain or region including LBD domain and C-terminal domain was affected.

In the "Omaveloxolone acts as a molecular crowbar to destabilize STING conformation" section, it would be interesting to discuss the model for inactive compounds (D5-1 and LPH-A134, Table 1 and Extended Data Fig. 4), why don't these compounds change in binding affinity, despite being derived from the critical anchoring region at the LBD base?

HUWE1 mediated K27-linked ubiquitination has been reported to promote inflammasome activation (for example NLRP3, AIM2, and NLRC4), but not degradation. Although omaveloxolonecan could cause hSTING K27-linked ubiquitination in the assay of Figure 5i, it's not necessarily that homotypic K27-linked ubiquitination is used in cells to promote the degradation of hSTING. Is ubiquitinated hSTING observed in omaveloxolone-treated or omaveloxolone and PR171-cotreated cells? IP-hSTING should be probed for specific ubiquitin chains using chain-specific antibodies and/or subjected to mass spectrometry to distinguish whether homotypic K27-linked ubiquitin chains are added to hSTING. To support the experiment in Figure 5i, it would be good to include a control sample extracted from cells transfected with HA-Ub (plus parallel mutant experiments), Flag-STING and treated with omaveloxolone to whether the exogenous protein gets degraded differently depending on the ubiquitin linkage.

It's better to include experiments to dissect whether omaveloxolone's contribution to aging-related inflammation acts mainly through hSTING or hSTING as well as other downregulated proteins. And it would be good to discuss any connections existing among these proteins.

Comparing Fig 3h with 3b, why did the melting temperature increase in hSTING S162A and T263A? Could this stabilizing effect prevent the destabilizing effect of Omaveloxolone? Also for S162A, T267A or T263A, T267 the y-axis value is decreased with Omaveloxolone; why is this?

An alternative model could be that Omaveloxolone binds to HUWE-1 and promotes its interaction with STING. There doesn't seem to be any data precluding this possibility.

In Discussion the authors should connect their findings to the knowledge that Omaveloxolone is an NRF2 activator. Is it

simply serendipity that both proteins are targeted by this compound or is there some shared attribute?

Minor comments
Figure 3a, it's better to plot FP values as y axis.
The labeling in the Figures is not consistent.
Line 121 'serves' should be 'serving'

Reviewer #2

(Remarks to the Author)
The manuscript investigates Omaveloxolone as a non-covalent STING degrader and proposes that it acts through a "molecular crowbar" mechanism that destabilizes the STING ligand-binding domain. The authors use a screening approach followed by biochemical, biophysical, and cellular assays—including FP, thermal shift, mutagenesis, ITC, computational modeling, and proteomics—to build a mechanistic framework for how the compound promotes HUWE1-dependent ubiquitination and degradation of STING. The work extends into in vivo models, showing effects in both TREX1–/– mice and naturally aged animals, and demonstrates degradation of both wild-type and gain-of-function STING variants by a drug that is already clinically approved and capable of brain penetration. Overall, the manuscript is of high quality, with strong data and a compelling story. The mechanistic claims are mostly well supported, and the therapeutic argument is well situated within current interest in STING biology and inflammation. With that said, there are areas where additional nuance and caution would improve both the scientific rigor and the credibility of the central claims.
The claim that Omaveloxolone functions as a molecular crowbar is plausible, but the field's definition of "crowbar" is still in its infancy, and the authors may be overextending the strength of the inference. At present, the evidence for destabilization is indirect—protein thermal shift and MD simulations—and while these are certainly suggestive, they do not unequivocally demonstrate the specific conformational mechanism proposed. A more measured tone, or ideally a complementary experimental assay (HDX-MS, limited proteolysis, backbone protection analyses), would go a long way toward solidifying the mechanistic argument.
The discrepancy between the MD simulations (one molecule bound) and ITC (two binding events) is brushed aside a bit too quickly. Readers will immediately notice this inconsistency, and at minimum a thoughtful explanation or hypothesis is needed. Even if the authors cannot resolve it experimentally, acknowledging the tension would show intellectual honesty.
The identification of HUWE1 as the responsible E3 ligase is convincing, but the mechanistic model (that HUWE1 engages STING specifically because the crowbar effect exposes hydrophobic regions) remains speculative. Recruitment could just as well be downstream of early ubiquitination events or altered trafficking. The authors are close to a strong mechanistic claim here, and a few additional controls—particularly proteasome blockade followed by interaction assays—would help distinguish cause from consequence.
The proteomic data revealing downregulation of other proteins is addressed somewhat superficially. Several of these proteins have meaningful roles in immune regulation or nucleic acid metabolism, and dismissing them as irrelevant is too quick. It is admittedly difficult to know what is direct and what is downstream of STING suppression, but the manuscript would benefit from a simple experiment in STING-KO cells to determine whether these effects are STING-dependent.
The in vivo section is strong in terms of efficacy but limited in terms of safety or tolerability. Since Omaveloxolone is used chronically in these mice, even limited indicators—body weight trajectories, gross pathology, or clinical chemistry—would strengthen the translational argument. Without such data, the reader is left to assume tolerability rather than being shown it.
Because Omaveloxolone is a known NRF2 activator, the anti-inflammatory effects observed in aged animals likely represent a mixture of STING suppression and NRF2-driven cytoprotective gene expression. The manuscript acknowledges the NRF2 connection only briefly, but reviewers will certainly raise it. Addressing this more directly—either by adding data or by discussing limitations—will significantly improve the manuscript's scientific balance.

Reviewer #3

(Remarks to the Author)
The manuscript titled "Omaveloxolone functions as a molecular crowbar degrader of STING to alleviate aging-related inflammation" presents an intriguing and compelling study. The authors propose an innovative mechanism for Omaveloxolone, identifying it as a "molecular crowbar" that directly destabilizes and degrades STING. Overall, the experimental design is rigorous, and the data appear robust and well-controlled. The authors successfully demonstrate the therapeutic potential of this mechanism in the context of aging-related inflammation.
However, despite the methodological rigor, further clarification is required regarding the structural basis of the drug-target interaction and respective contributions of its dual mechanisms.
Major Comments:
1. Despite extensive MD analyses, empirical structural evidence is necessary to fully elucidate the Omaveloxolone-STING interaction. To validate the proposed "molecular crowbar" mechanism, the authors should provide a crystal structure or Cryo-EM analysis of the complex.
2. While the authors demonstrated that STING degradation occurs independently of NRF2, the study does not clearly differentiate the therapeutic contribution of Omaveloxolone's NRF2 activation from its STING-degrading activity in the disease model. The authors should either dissect the relative impact of these two mechanisms on the observed phenotype or explicitly characterize Omaveloxolone as a dual-function modulator that targets both pathways synergistically.

Reviewer #4

(Remarks to the Author)
I co-reviewed this manuscript with one of the reviewers who provided the listed reports. This is part of the Nature Communications initiative to facilitate training in peer review and to provide appropriate recognition for Early Career Researchers who co-review manuscripts.

Version 1:

Reviewer comments:

Reviewer #1

(Remarks to the Author)
All concerns have been addressed; congratulations on your exciting results!

Reviewer #2

(Remarks to the Author)
The reviewers have replied to my concerns in a satisfactory manner.

Reviewer #3

(Remarks to the Author)
I have carefully evaluated the revised manuscript NCOMMS-25-93443A, "Omaveloxolone functions as a molecular crowbar-like degrader of STING to alleviate aging-related inflammation," along with your point-by-point response letter. I am pleased to note that you have addressed all major concerns raised in the previous round of review in a thorough and satisfactory manner.
In particular, the following additions substantially strengthen the manuscript:
1) The HDX-MS analysis provides compelling orthogonal experimental evidence for Omaveloxolone-induced conformational destabilization of the STING LBD, complementing the existing thermal shift and MD simulation data.
2) The K27 linkage-specific endogenous ubiquitination assays, combined with the ubiquitin K-only mutant experiments, convincingly demonstrate that Omaveloxolone promotes homotypic K27-linked polyubiquitination of STING in a cellular context.
3) The STING-KO proteomic profiling clarifies the STING-dependence of downstream protein changes and provides useful mechanistic context for the off-target observations.
4) The HUWE1 recruitment controls (proteasome inhibition Co-IP and STING LBD-based NanoBiT assays) effectively address the alternative model in which HUWE1 engagement could be secondary to prior ubiquitination or altered STING trafficking.
5) Your decision to adopt more measured language—describing Omaveloxolone as exhibiting "molecular crowbar-like behavior" rather than definitively classifying it as a molecular crowbar—is scientifically responsible and appropriately reflects the current state of the field.
The mechanistic framework is now well-supported by multiple independent experimental approaches, and the therapeutic rationale in the context of aging-related inflammation is clearly and compellingly articulated.

Reviewer #4

(Remarks to the Author)
I co-reviewed this manuscript with one of the reviewers who provided the listed reports. This is part of the Nature Communications initiative to facilitate training in peer review and to provide appropriate recognition for Early Career Researchers who co-review manuscripts.

Open Access This Peer Review File is licensed under a Creative Commons Attribution 4.0 International License, which permits use, sharing, adaptation, distribution and reproduction in any medium or format, as long as you give appropriate credit to the original author(s) and the source, provide a link to the Creative Commons license, and indicate if changes were made.
In cases where reviewers are anonymous, credit should be given to 'Anonymous Referee' and the source.
The images or other third party material in this Peer Review File are included in the article's Creative Commons license, unless indicated otherwise in a credit line to the material. If material is not included in the article's Creative Commons license and your intended use is not permitted by statutory regulation or exceeds the permitted use, you will need to obtain permission directly from the copyright holder.
To view a copy of this license, visit https://creativecommons.org/licenses/by/4.0/

Thank you for the thoughtful feedback from the reviewers. We sincerely appreciate the time and expertise dedicated to evaluating our manuscript, which have guided substantial improvements to our work. In response, we have carefully revised the manuscript to address all raised concerns and implement all reviewer suggestions. Our point-by-point responses are detailed below. Related modifications have been highlighted in red in the revised manuscript.

Reviewer #1 (Remarks to the Author)

In this study, Zhou et. al identified NRF2 activator omaveloxolone as a strong binder and degrader of hSTING. They use molecular dynamics and biophysical analyses to conclude that omaveloxolone destabilization occurs through a "molecular crowbar" mechanism whereby loosening of the structural integrity increases HUWE1 binding affinity and in turn, degradation by the HUWE1-UbcH5b-proteasome axis. The authors also found degradation of hSTING by omaveloxolone suppresses its downstream inflammatory signaling and ameliorates aging-related inflammatory phenotypes, including in mice. This study will be of great interest to researchers in TPD and aging-related inflammation areas of research and has clinical relevance. This is an outstanding study with some concerns listed below.

Reply: We sincerely appreciate the reviewer's summary and positive feedback. Our detailed responses are provided below.

1. It's confusing to mention hSTING LBD and CTD in the "Omaveloxolone binds non-covalently to STING LBD with high affinity and reduces STING protein thermal stability" section. Based on the written method, only the CTD of hSTING (residues 139-379) containing LBD and C-terminal domains was expressed and purified. It'd be better to include a schematic representation of human STING domains and clarify which construct is used for the experiment in the Figure 3, as done for Figure 5g. Otherwise, it's hard to know whether hSTING full length, LBD domain or region including LBD domain and C-terminal domain was affected.

Reply: We greatly appreciate the reviewer's important suggestion, which is critical for eliminating terminology ambiguity and improving the clarity of our result description.

To unify the terminology and avoid confusion, we have revised all relevant domain descriptions of

hSTING to “hSTING LBD” throughout this section. Meanwhile, a schematic representation of human STING domains is now provided in Fig. 3, and the construct used in this study has been explicitly indicated in the revised manuscript.

As noted by the reviewer, the hSTING C-terminal domain (CTD, residues 139–379), which contains the ligand-binding domain (LBD) and the C-terminal tail (CTT), was expressed and purified in this study. The LBD serves as the core functional region for STING’s ligand recognition and binding. It is also the main focus of our fluorescence polarization (FP) binding assays and molecular dynamics (MD) simulations, given that both the Omaveloxolone binding site and its induced conformational perturbation are predominantly confined to the LBD. Accordingly, we consistently use “LBD” throughout this section to emphasize that the affected structural region corresponds to the hSTING LBD.

2. In the “Omaveloxolone acts as a molecular crowbar to destabilize STING conformation” section, it would be interesting to discuss the model for inactive compounds (D5-1 and LPH-A134, Table 1 and Extended Data Fig. 4), why don’t these compounds change in binding affinity, despite being derived from the critical anchoring region at the LBD base?

Reply: We greatly appreciate the reviewer’s insightful suggestion. In the original manuscript, to dissect why analogs that bind to STING but lack destabilizing effects are deficient in degrading activity, we performed MD simulations using the STING-Omaveloxolone complex as a reference, focusing on two representative inactive compounds, D5-1 and LPH-A134. Simulations revealed that these analogs bind at positions shifted upward relative to Omaveloxolone, deviating from the critical anchoring region at the LBD base (Extended Data Fig. 4d, Fig. R1).

A possible explanation for why D5-1 and LPH-A134 retain comparable binding affinity despite deviating from the critical anchoring region is that the upward-shifted binding poses may still allow engagement within the STING LBD pocket. Binding-mode analysis revealed that, compared with Omaveloxolone, both D5-1 and LPH-A134 adopt upward-shifted poses that enable hydrogen bonding with D237, a residue located in the upper β-sheet lid region of the STING LBD (Fig. R1). These additional interactions, together with retained contacts mediated by the shared core scaffold, may compensate for reduced engagement with the deeper LBD-base anchoring region and thereby help preserve their overall binding affinity.

However, this altered binding geometry may compromise their ability to transmit the pose-dependent conformational perturbation required for molecular crowbar-like destabilization. In particular, engagement with the upper lid region and reduced deep anchoring may favor LBD reclosure rather than sustaining the open, destabilized conformation induced by Omaveloxolone. Thus, D5-1 and LPH-A134 appear to behave as non-productive binders: they retain binding to STING, but fail to induce the open conformation, reduced thermal stability, and subsequent degradation observed with Omaveloxolone.

We have incorporated this mechanistic discussion and the additional analysis into the revised manuscript.

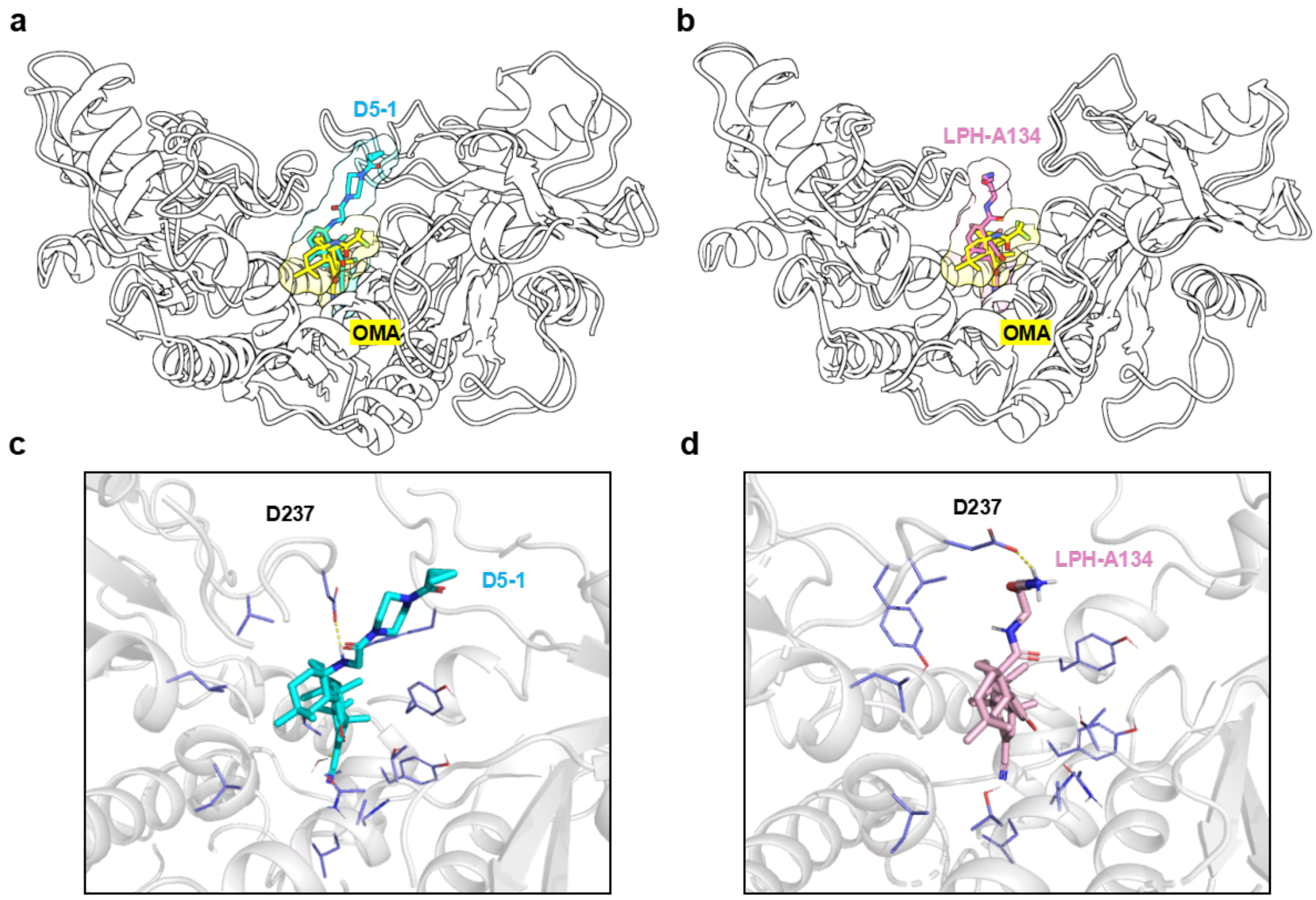

Fig. R1. a, Superposition of representative ligand poses from the stable MD simulation stage of the D5-1-STING complex (blue) and the Omaveloxolone-STING complex (yellow). **b,** Superposition of representative ligand poses from the stable MD simulation stage of the LPH-A134-STING complex (magenta) and the Omaveloxolone-STING complex (yellow). **c,d,** Binding mode analysis of D5-1 (**c**) and LPH-A134 (**d**) in the STING LBD. OMA denotes Omaveloxolone.

3. HUWE1 mediated K27-linked ubiquitination has been reported to promote inflammasome activation (for example NLRP3, AIM2, and NLRC4), but not degradation. Although omaveloxolone can could cause hSTING K27-linked ubiquitination in the assay of Figure 5i, it's not necessarily

that homotypic K27-linked ubiquitination is used in cells to promote the degradation of hSTING. Is ubiquitinated hSTING observed in omaveloxolone-treated or omaveloxolone and PR171-cotreated cells? IP-hSTING should be probed for specific ubiquitin chains using chain-specific antibodies and/or subjected to mass spectrometry to distinguish whether homotypic K27-linked ubiquitin chains are added to hSTING. To support the experiment in Figure 5i, it would be good to include a control sample extracted from cells transfected with HA-Ub (plus parallel mutant experiments), Flag-STING and treated with omaveloxolone to whether the exogenous protein gets degraded differently depending on the ubiquitin linkage.

Reply: We thank the reviewer for this insightful suggestion, which has been instrumental in strengthening our mechanistic evidence chain and improving the scientific rigor of our manuscript. To determine whether homotypic K27-linked ubiquitin chains are added to hSTING, we performed additional endogenous ubiquitination assays as suggested by the reviewer: THP-1 cells were treated with Omaveloxolone alone, or co-treated with Omaveloxolone and the proteasome inhibitor PR171. Endogenous STING was immunoprecipitated using an anti-STING antibody, followed by immunoblotting with a K27 linkage-specific ubiquitin antibody. As shown in Fig. R2a, a clear K27-linked ubiquitination signal was detected upon Omaveloxolone and PR171 co-treatment, supporting that Omaveloxolone induces K27-linked polyubiquitination of endogenous STING and suggesting that intracellular homotypic K27-linked ubiquitin chains participate in the OMA-mediated degradation of STING.

To determine whether exogenous STING gets degraded differently depending on the ubiquitin linkage, we co-transfected HEK293T cells with Flag-STING together with HA-tagged ubiquitin single-lysine retention mutants (K-only mutants) as suggested. Upon Omaveloxolone treatment, STING degradation exhibited a clear ubiquitin linkage type dependence, with the K27-linked chain being the most effective in driving STING degradation (Fig. R2b). These results provide further support for the conclusions drawn from Figures 5i and 5j.

As noted by the reviewer, existing studies have reported that HUWE1-mediated K27-linked ubiquitination regulates the activation of inflammasomes such as NLRP3, AIM2, and NLRC4, whereas no previous work has documented a role for this modification in mediating substrate protein degradation. Our study suggests that proteasomal degradation of substrate proteins may also be a functional outcome of HUWE1-catalyzed K27-linked ubiquitination, thereby expanding the

understanding of the functional diversity of HUWE1-mediated ubiquitination modifications.

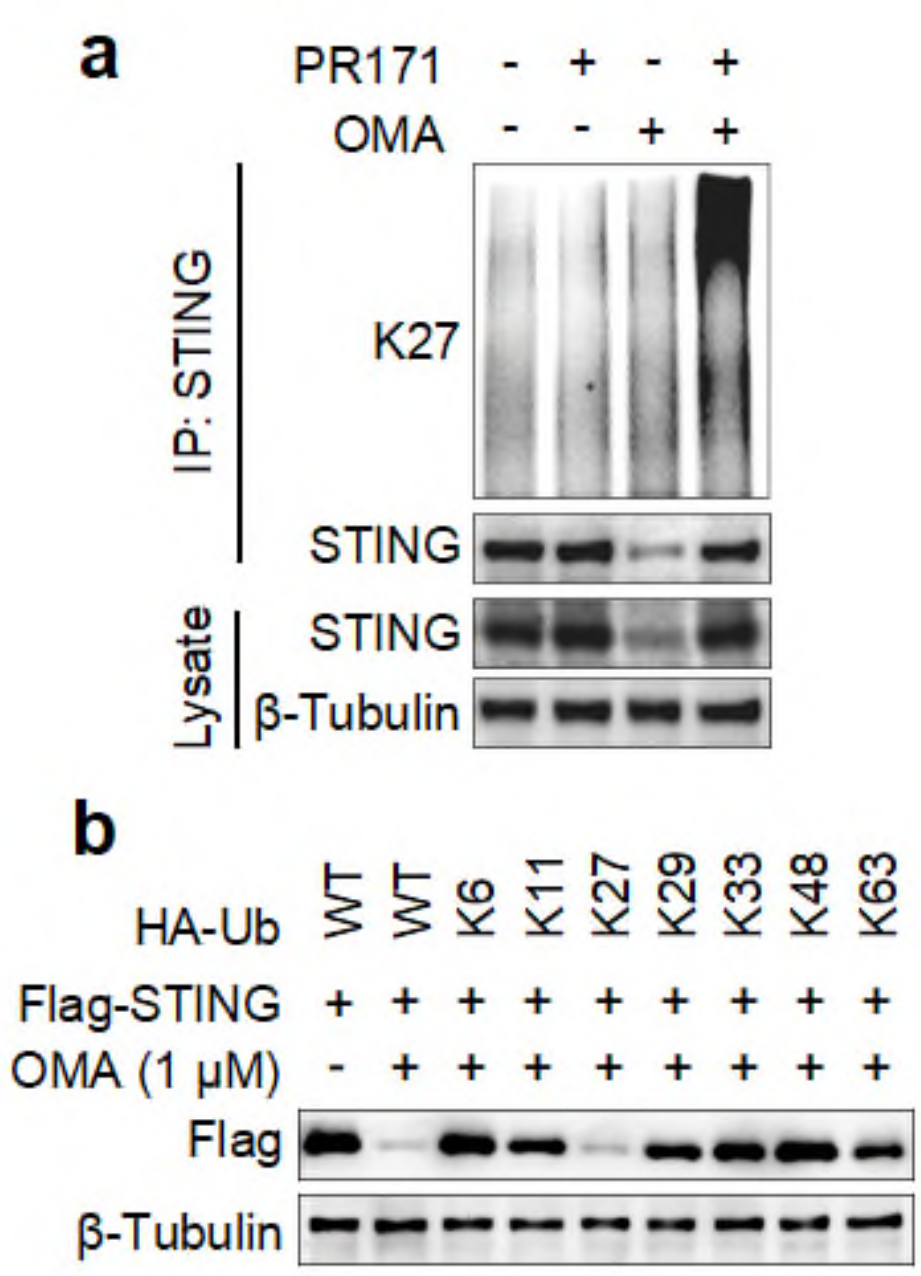

Fig. R2. a, Omaveloxolone induces K27-linked polyubiquitination of endogenous STING. THP-1 cells were pretreated with PR171 (2 μM) for 1 h followed by OMA (1 μM) for 8 h. Endogenous STING was immunoprecipitated and probed with K27 linkage-specific ubiquitin antibodies. **b,** Effects of Ub mutants on OMA-induced exogenous STING degradation. HEK293T cells expressing Flag-STING together with HA-tagged ubiquitin mutants were treated with OMA (1 μM) for 12 h. OMA denotes Omaveloxolone.

4. It's better to include experiments to dissect whether omaveloxolone's contribution to aging-related inflammation acts mainly through hSTING or hSTING as well as other downregulated proteins. And it would be good to discuss any connections existing among these proteins.

Reply: We thank the reviewer for this valuable suggestion. To determine whether Omaveloxolone's contribution to aging-related inflammation acts mainly through STING, we established cellular senescence models induced by Doxorubicin (Dox) and ionizing radiation (IR) in wild-type (WT) and STING-KO THP-1/RAW 264.7 cells (Fig. R3, Fig. R4), as Dox and IR are well-established inducers of cellular senescence that recapitulate key features of aging-related inflammation, including senescence-associated secretory phenotype (SASP) factor upregulation and pro-inflammatory cytokine secretion[1–3]. In WT cells, Omaveloxolone treatment significantly alleviated senescence-related inflammation: it reduced the mRNA levels of senescence markers (*P16* and *P21*)

and pro-inflammatory SASP factors (*IL6* and *IL1B*), while also decreasing the proportion of senescence-associated β-galactosidase (SA-β-Gal)-positive cells (Fig. R4a–d). In contrast, such effects were substantially diminished in STING-KO THP-1 and RAW 264.7 cells (Fig. R4e–h), supporting that the ability of Omaveloxolone to ameliorate cellular senescence-associated inflammation is STING-dependent. Although our *in vitro* senescence models clearly show that Omaveloxolone modulates senescence-associated inflammation in a largely STING-dependent manner (Fig. R4), the *in vivo* aging microenvironment is far more complex than cell culture systems. Our existing *in vivo* data cannot definitively confirm that the ability of Omaveloxolone to ameliorate systemic inflammation in aged mice is also primarily mediated by STING, which represents a major limitation of our *in vivo* mechanistic characterization.

To investigate the connections between STING and the other downregulated proteins revealed by our proteomic data in THP-1 cells, we performed additional proteomic analysis on STING-KO THP-1 cells. As shown in Fig. R5, among the downregulated proteins in WT THP-1 cells, the Omaveloxolone-mediated downregulation of ARMC6, SETX, and USP3 remained significant in STING-KO THP-1 cells, indicating that their downregulation occurs largely through STING-independent mechanisms. In contrast, the downregulation of IL1B and TDO2—two proteins known to be functionally interconnected in inflammatory regulation—was abolished upon STING deletion, demonstrating that their suppression is STING-dependent. IL1B serves as a canonical pro-inflammatory downstream effector of NF-κB signaling[4], while TDO2 functions as a key immunomodulatory enzyme induced by inflammatory stress[5]. Importantly, pro-inflammatory cytokines including IL1B can induce TDO2 expression[6], and TDO2-derived metabolites can in turn modulate inflammatory signaling[7]. The coordinated STING-dependent downregulation of these two factors may collectively contribute to the protective effects of Omaveloxolone against aging-related inflammation.

We have incorporated these findings and discussions into the revised manuscript.

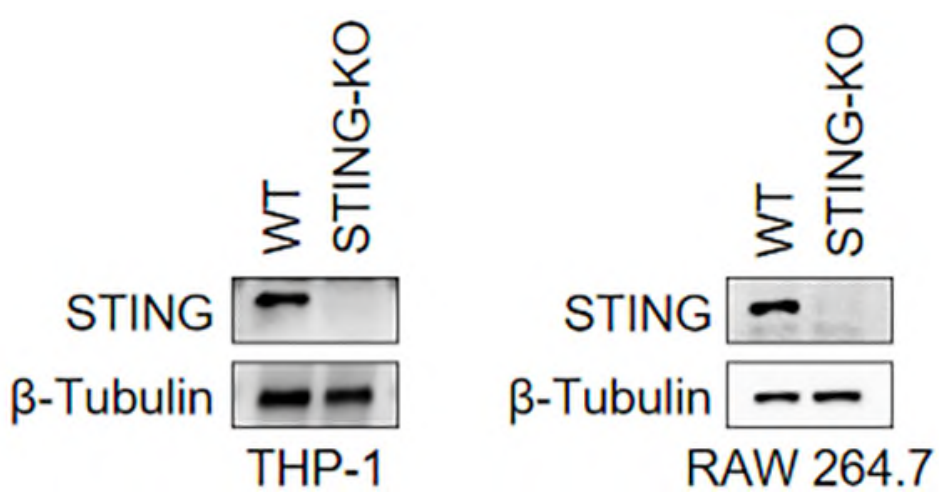

Fig. R3. Western blot analysis of STING protein levels in WT and STING-KO THP-1/RAW

264.7 cells.

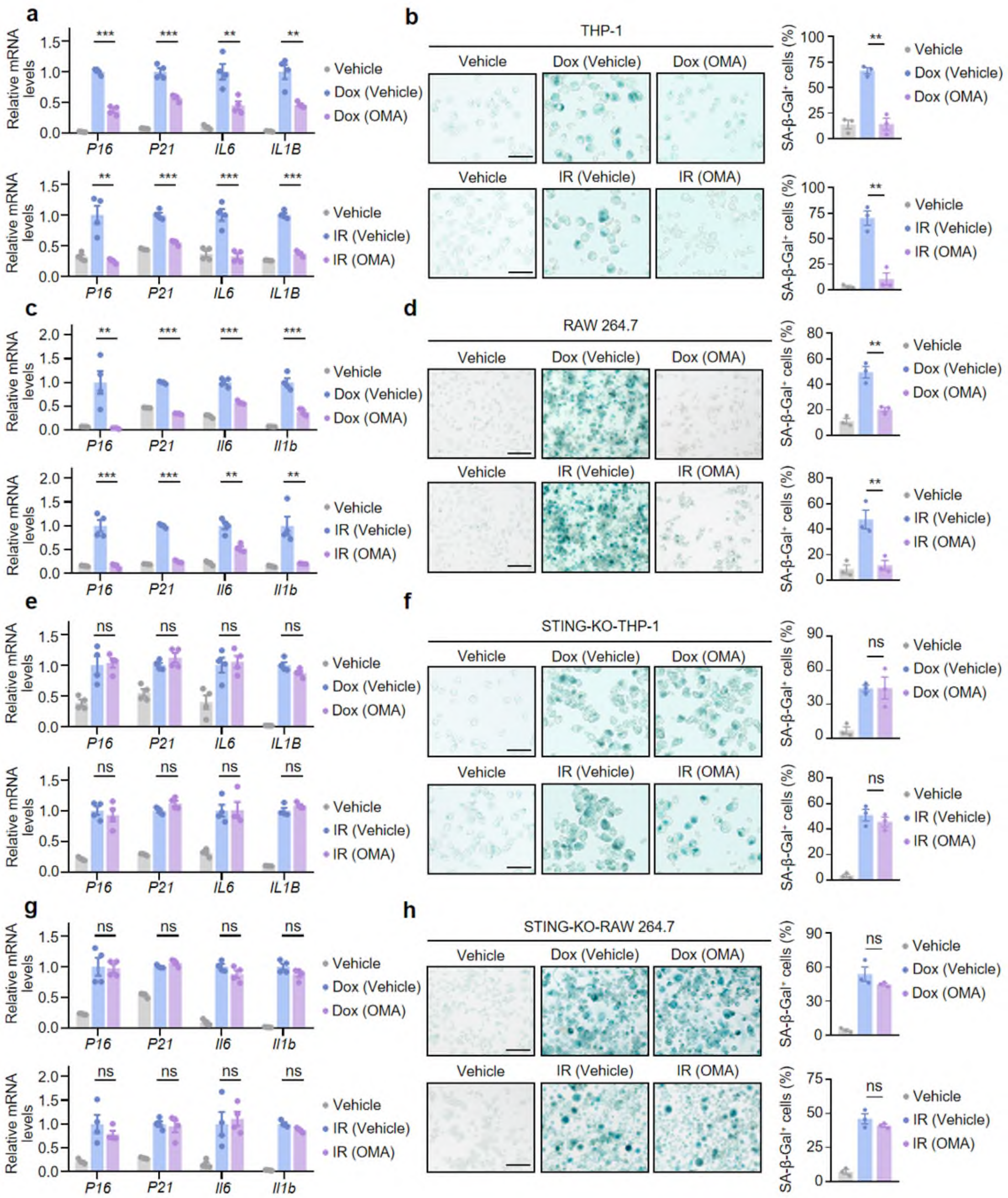

Fig. R4. Omaveloxolone alleviates cellular senescence and senescence-related inflammation in a STING-dependent manner. a,b, THP-1 cells were treated with 200 nM Dox for 24 h followed by 4 days of culture, or irradiated with 7.5 Gy X-ray and cultured for 7-9 days, to induce cellular senescence. Cells were then treated with 500 nM OMA for 24 h. The mRNA levels of senescence markers (*P16*, *P21*) and pro-inflammatory SASP factors (*IL6*, *IL1B*) were measured by RT-qPCR (n = 4, two technical replicates from two independent experiments, mean ± SEM) (**a**). Cellular senescence was assessed by SA-β-Gal staining; representative images and quantifications are shown (scale bar, 50 μm; n = 3 independent biological replicates, mean ± SEM) (**b**). **c,d,** RAW 264.7 cells

were treated with 200 nM Dox for 24 h followed by 2 days of culture, or irradiated with 6 Gy X-ray and cultured for 4-6 days, to induce cellular senescence. Cells were then treated with 1 µM OMA for 24 h. The mRNA levels of *P16*, *P21*, *Il6*, and *Il1b* were measured by RT-qPCR (*n* = 4, two technical replicates from two independent experiments, mean ± SEM) (**c**). Representative SA-β-Gal staining images and quantifications are presented (scale bar, 100 µm; *n* = 3 independent biological replicates, mean ± SEM) (**d**). **e,f,** STING-KO-THP-1 cells were subjected to senescence induction as in **a,b**, followed by 500 nM OMA treatment for 24 h. The mRNA levels of *P16*, *P21*, *IL6*, and *IL1B* were measured by RT-qPCR (*n* = 4, two technical replicates from two independent experiments, mean ± SEM) (**e**). Representative SA-β-Gal staining images and quantifications are presented (scale bar, 50 µm; *n* = 3 independent biological replicates, mean ± SEM) (**f**). **g,h,** STING-KO-RAW 264.7 cells were subjected to senescence induction as in **c,d**, followed by 1 µM OMA treatment for 24 h. The mRNA levels of *P16*, *P21*, *Il6*, and *Il1b* were measured by RT-qPCR (*n* = 4, two technical replicates from two independent experiments, mean ± SEM) (**g**). Representative SA-β-Gal staining images and quantifications are presented (scale bar, 100 µm; *n* = 3 independent biological replicates, mean ± SEM) (**h**). OMA denotes Omaveloxolone. ns, not significant; $P > 0.05$; $^{**}P < 0.01$; $^{***}P < 0.001$ by two-tailed unpaired *t*-test.

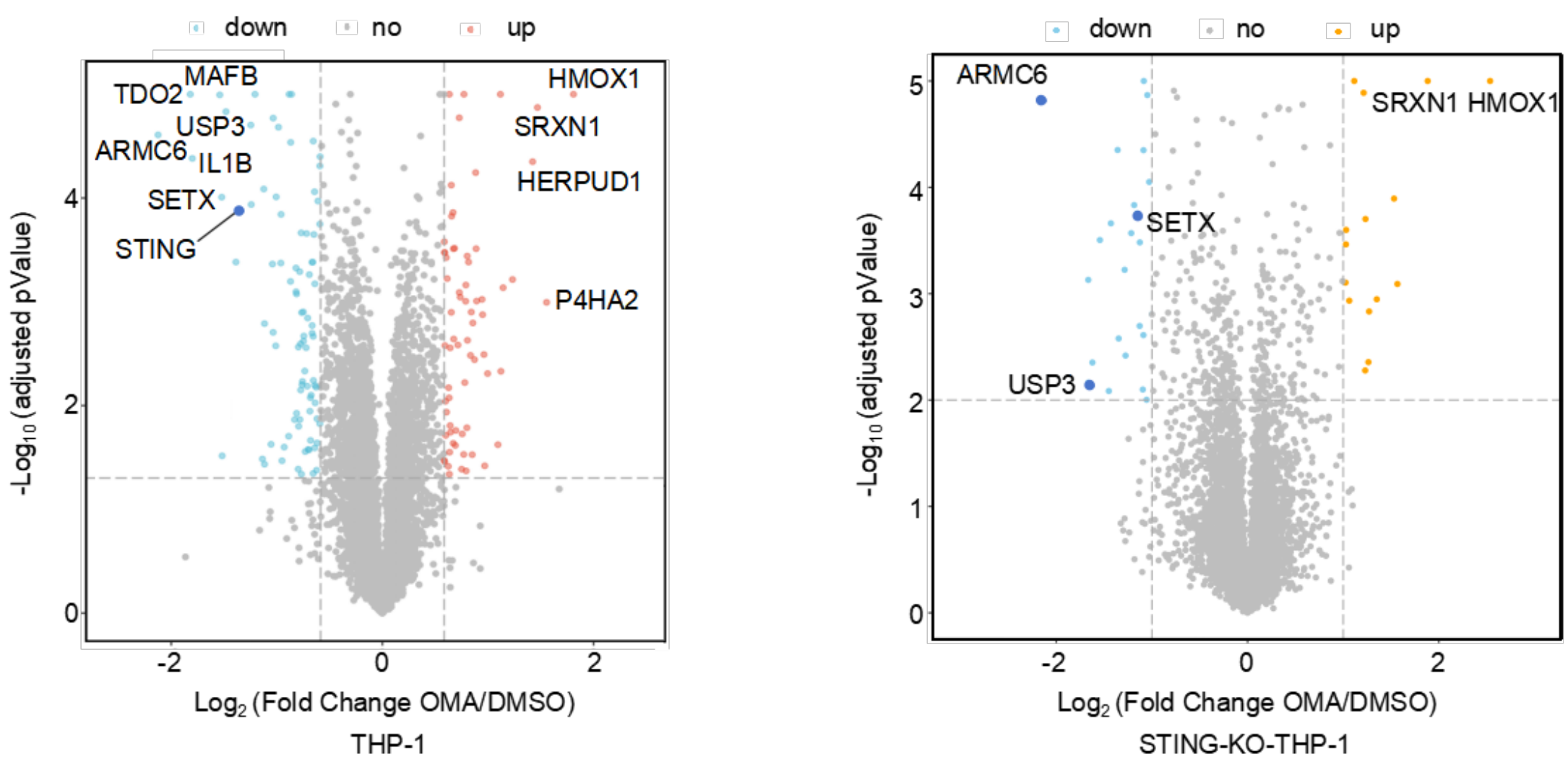

Fig. R5. Proteomic analysis of THP-1 and STING-KO-THP-1 cells treated with 500 nM Omaveloxolone (OMA) for 12 h. The volcano plot displays differentially expressed proteins compared to the DMSO control.

References:

[1] Rodier F, Coppé JP, Patil CK, Hoeijmakers WA, Muñoz DP, Raza SR, Freund A, Campeau E, Davalos AR, Campisi J. Persistent DNA damage signalling triggers senescence-associated inflammatory cytokine secretion. Nat Cell Biol. 2009, 11(8):973-9.

[2] Liu Y, Ye J, Fan Z, Wu X, Zhang Y, Yang R, Jiang B, Wang Y, Wu M, Zhou J, Meng J, Ge Z, Zhou G, Zhu Y, Xiao Y, Zheng M, Zhang S. Ginkgetin Alleviates Inflammation and Senescence by

Targeting STING. Adv Sci (Weinh). 2025, 12(2):e2407222.
[3] Marques M, Hayashide LS, Amorim P, Fernandes BM, Araujo APB, Messor DF, Leocadio VE, Pessoa B, Corrêa JBLP, Villablanca C, Vidal RL, González-Billault C, Matias I, Gomes FCA, Diniz LP. Doxorubicin Induces a Senescent Phenotype in Murine and Human Astrocytes. J Neurochem. 2025, 169(8):e70177.
[4] Bleve A, Motta F, Durante B, Pandolfo C, Selmi C, Sica A. Immunosenescence, Inflammaging, and Frailty: Role of Myeloid Cells in Age-Related Diseases. Clin Rev Allergy Immunol. 2023, 64(2):123-144.
[5] Platten M, Nollen EAA, Röhrig UF, Fallarino F, Opitz CA. Tryptophan metabolism as a common therapeutic target in cancer, neurodegeneration and beyond. Nat Rev Drug Discov. 2019, 18(5):379-401.
[6] Rong G, Zhang T, Xu Y, Zhang Z, Gui B, Hu K, Zhang J, Tang Z, Shen C. High levels of TDO2 in relation to pro-inflammatory cytokines in synovium and synovial fluid of patients with osteoarthritis. BMC Musculoskelet Disord. 2022, 23(1):604.
[7] Wawrzak-Pienkowska K, Golonko A, Ignatiuk D, Swidnicka-Siergiejko A, Pienkowski T, Kurek K. Kynurenine pathway impact on immune evasion and inflammation in acute myeloid leukemia. Life Sci. 2026, 400:124474.

5. Comparing Fig 3h with 3b, why did the melting temperature increase in hSTING S162A and T263A? Could this stabilizing effect prevent the destabilizing effect of Omaveloxolone? Also for S162A, T267A or T263A, T267 the y-axis value is decreased with Omaveloxolone; why is this?

Reply: We appreciate the reviewer's careful observation and insightful questions. The increased melting temperature of hSTING S162A and T263A mutants reflects intrinsic changes in protein stability. Mutating serine/threonine residues to alanine may eliminate the hydroxyl side chain, potentially reducing local structural flexibility and restricting conformational disorder[1–3], thereby likely enhancing the basal thermostability of STING and resulting in a higher T_m value.

We reasoned that this stabilizing effect caused by these mutations is unlikely to counteract the destabilizing effect of Omaveloxolone. Instead, the loss of Omaveloxolone's destabilizing effect more likely results from the inability of the mutant proteins to bind the compound. To verify this, we performed ITC measurements. As shown in Fig. R6a–b, no measurable binding was detected between Omaveloxolone and either S162A or T263A. This indicates that the loss of Omaveloxolone-mediated STING destabilization in these mutants is directly attributed to the abolished compound-protein interaction, rather than the altered basal thermostability caused by

these mutations.

The reviewer also observed a minor reduction in y-axis signal for the S162A, T267A, and T263A/T267A mutants after Omaveloxolone treatment. Upon careful review of all data, we attribute this signal variation to normal experimental error inherent to the protein shift assay, caused by unavoidable technical factors including pipetting errors and well-to-well differences. We have included the complete results of three independent biological replicates of the protein shift assay (Fig. R6c–f), which clearly show minor signal variations between technical replicates, indicating that the phenomenon observed by the reviewer is attributable to the normal range of experimental error.

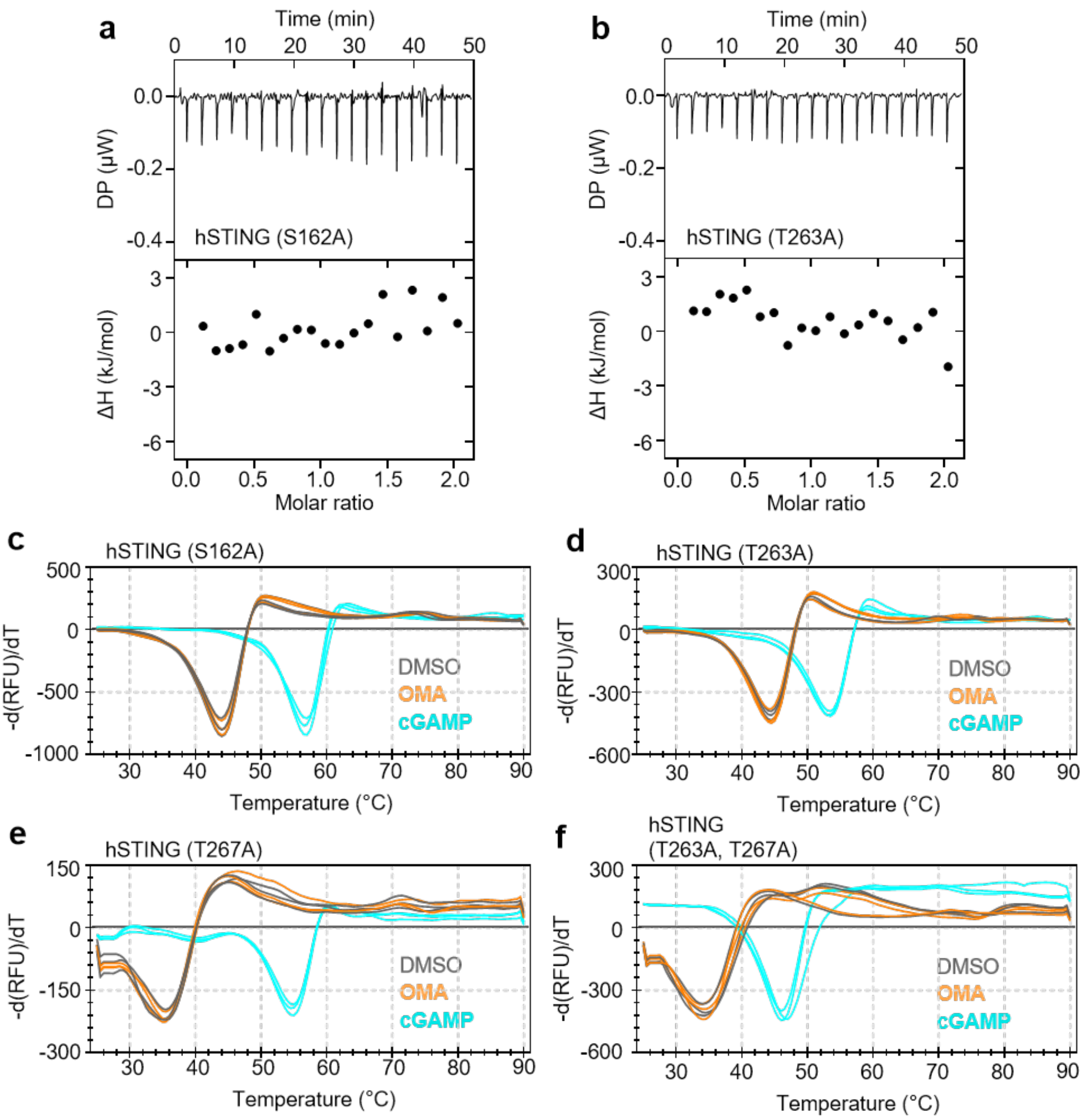

Fig. R6. ITC and PTS measurements for the binding between Omaveloxolone and STING mutants. a–b, ITC measurements confirm no detectable binding of OMA to hSTING S162A, T263A. **c–f**, PTS assays were performed in three independent biological replicates for the indicated STING mutants. OMA denotes Omaveloxolone.

References:
[1] Hoya M, Matsunaga R, Nagatoishi S, Ide T, Kuroda D, Tsumoto K. Impact of single-residue mutations on protein thermal stability: The case of threonine 83 of BC2L-CN lectin. Int J Biol Macromol. 2024, 272(Pt 1):132682.
[2] Heinz DW, Baase WA, Matthews BW. Folding and function of a T4 lysozyme containing 10 consecutive alanines illustrate the redundancy of information in an amino acid sequence. Proc Natl Acad Sci U S A. 1992, 89(9):3751-5.
[3] Hou XN, Song B, Zhao C, Chu WT, Ruan MX, Dong X, Meng LS, Gong Z, Weng YX, Zheng J, Wang J, Tang C. Connecting Protein Millisecond Conformational Dynamics to Protein Thermal Stability. JACS Au. 2024, 4(8):3310-3320.

6. An alternative model could be that Omaveloxolone binds to HUWE-1 and promotes its interaction with STING. There doesn't seem to be any data precluding this possibility.

Reply: We thank the reviewer for this alternative possibility. To test whether Omaveloxolone interacts with HUWE1, we performed microscale thermophoresis (MST) binding assays. As shown in Fig. R7, no detectable binding was observed between Omaveloxolone and HUWE1. These data thus do not support the alternative model in which Omaveloxolone acts by directly binding to HUWE1. We have incorporated the data into the revised manuscript.

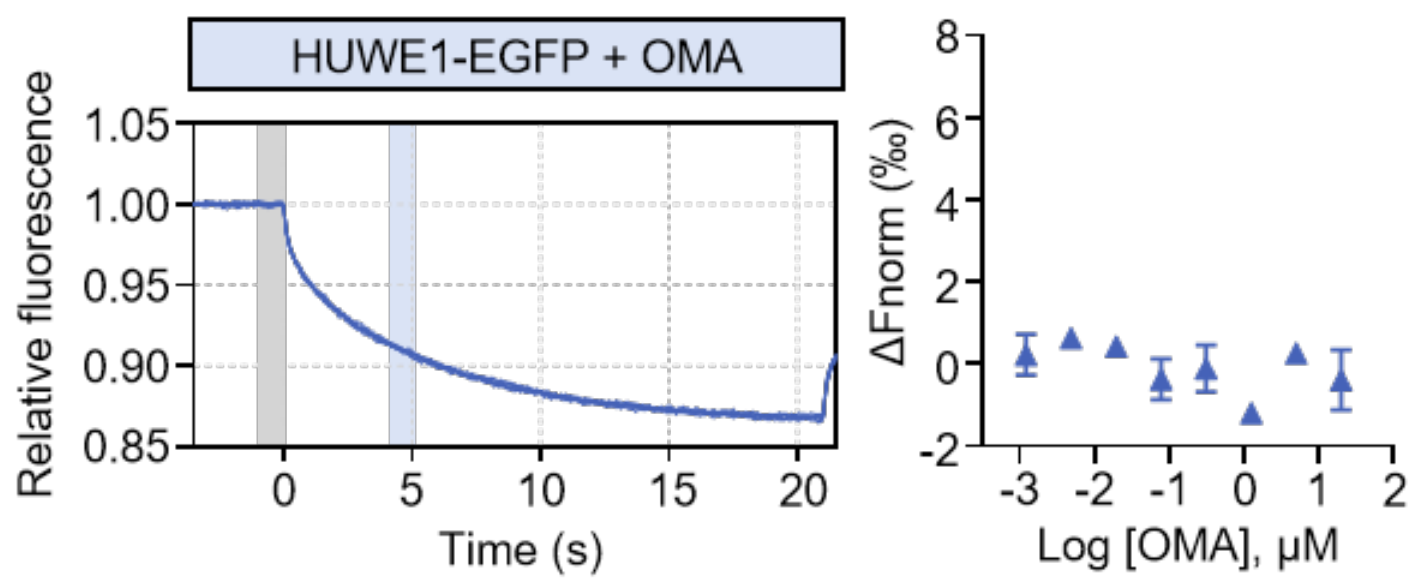

Fig. R7. MST analysis reveals no direct binding between Omaveloxolone and HUWE1. Representative MST traces (left panel) and dose-response curve (right panel) reveal no detectable binding (n = 3 independent biological replicates, mean ± SEM). OMA denotes Omaveloxolone.

7. In Discussion the authors should connect their findings to the knowledge that Omaveloxolone is an NRF2 activator. Is it simply serendipity that both proteins are targeted by this compound or is there some shared attribute?

Reply: We thank the reviewer for this insightful suggestion and comment. Combining the experimental results with the existing literature, we have added relevant discussions as follows:

Omaveloxolone targets STING degradation and NRF2 activation through mechanistically independent pathways. Our data demonstrate that Omaveloxolone-induced STING degradation occurs at the proteasomal level without altering STING mRNA expression, and this degradation is independent of NRF2 (Fig. 2e). In contrast, Omaveloxolone activates NRF2 via covalent modification of KEAP1, a well-documented mechanism of Omaveloxolone action. Importantly, proteomic analysis in STING-KO-THP-1 cells revealed that Omaveloxolone still robustly upregulates the canonical NRF2 downstream targets HMOX1 and SRXN1 to levels comparable to those in WT cells (Fig. R5), confirming that NRF2 activation does not rely on STING expression or degradation. These results exclude the possibility that one function is a byproduct of the other, and demonstrate that Omaveloxolone engages the two pathways independently.

While Omaveloxolone's dual targeting of STING and NRF2 may initially appear serendipitous, we propose this reflects a biologically meaningful convergence on two complementary pillars of cellular homeostasis: inflammation and oxidative stress. STING activation drives pro-inflammatory signaling and acts as a key contributor to aging-associated inflammation, while NRF2 serves as the master regulator of the antioxidant response, counteracting oxidative damage and maintaining redox balance. Importantly, both pathways are concurrently dysregulated during aging. Aging is characterized by chronic low-grade inflammation ("inflammaging"), to which STING contributes substantially[1, 2], as well as by impaired antioxidant capacity, which is largely governed by NRF2[3-5]. A therapeutic agent that simultaneously suppresses the inflammatory arm (via STING degradation) and bolsters the protective antioxidant arm (via NRF2 activation) therefore possesses strong mechanistic rationale. Omaveloxolone's dual modulation of these two functionally synergistic nodes may make it an ideal therapeutic candidate for the treatment of aging-related inflammation.

Although our cellular senescence models indicate that Omaveloxolone modulates cellular senescence-related inflammation primarily in a STING-dependent manner (Fig. R4), naturally aged mice exhibit a complex *in vivo* microenvironment in which STING-driven hyperinflammation coexists with impaired NRF2-mediated antioxidant capacity. Therefore, we cannot rule out the possibility that the *in vivo* protective effects of Omaveloxolone against aging-related inflammation may also be partially attributed to NRF2 activation. This represents a limitation of the present study regarding the *in vivo* pharmacological mechanism.

References:
[1] Gulen MF, Samson N, Keller A, Schwabenland M, Liu C, Glück S, Thacker VV, Favre L, Mangeat B, Kroese LJ, Krimpenfort P, Prinz M, Ablasser A. cGAS-STING drives ageing-related inflammation and neurodegeneration. Nature. 2023, 620(7973):374-380.
[2] Shang Z, Qin D, Liu X, Li H, Liu C, Zhang R, Sun T, Pan Z, Feng W, You X. The cGAS-STING pathway in age-related ocular diseases: Mechanisms and therapeutic opportunities. Cell Signal. 2025, 135:112069.
[3] Suh JH, Shenvi SV, Dixon BM, Liu H, Jaiswal AK, Liu RM, Hagen TM. Decline in transcriptional activity of Nrf2 causes age-related loss of glutathione synthesis, which is reversible with lipoic acid. Proc Natl Acad Sci U S A. 2004, 101(10):3381-6.
[4] Safdar A, deBeer J, Tarnopolsky MA. Dysfunctional Nrf2-Keap1 redox signaling in skeletal muscle of the sedentary old. Free Radic Biol Med. 2010, 49(10):1487-93.
[5] Zhang H, Davies KJA, Forman HJ. Oxidative stress response and Nrf2 signaling in aging. Free Radic Biol Med. 2015, 88(Pt B):314-336.

Minor comments

1. Figure 3a, it's better to plot FP values as y axis.

Reply: Thanks for your suggestion. Our FP assay was designed as a competitive binding system, in which Omaveloxolone competes with the fluorescent diABZI probe for binding to the STING LBD. Therefore, plotting the competition rate on the y-axis allows a more intuitive and straightforward evaluation of compound binding potency.

We acknowledge that raw FP values provide additional reference information. We have now included the raw FP values across different compound concentrations in Fig. R8, and these data have also been incorporated to the revised Extended Data Fig. 2.

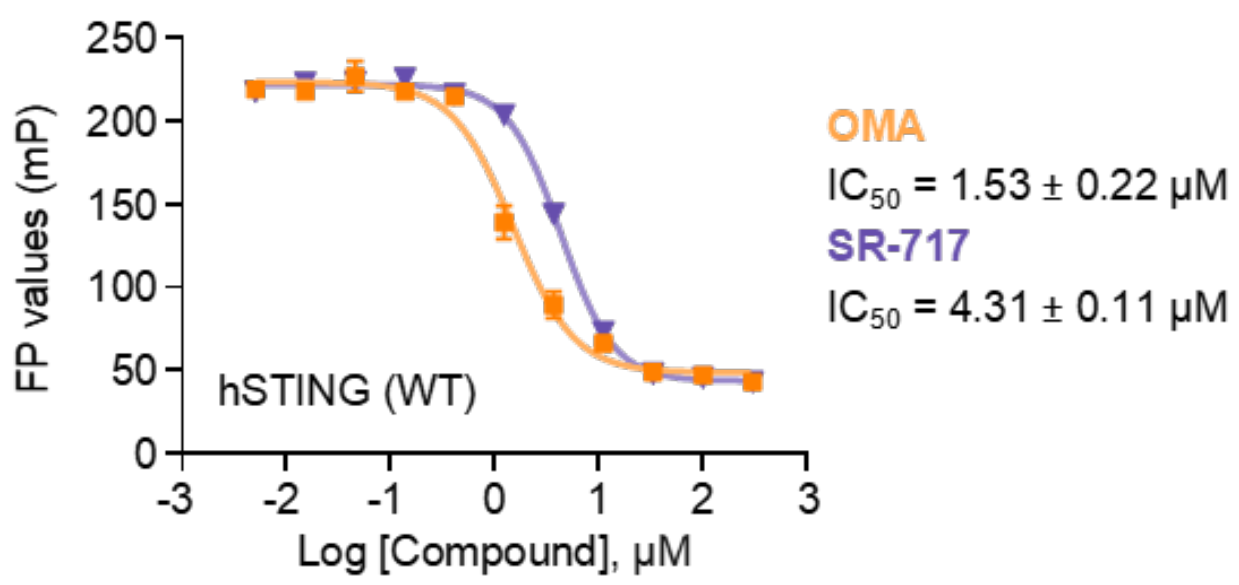

Fig. R8. FP values from competitive binding assays of Omaveloxolone and SR-717 to hSTING^{WT}. Data are presented as mean ± SD from four independent biological replicates, with FP values plotted against concentrations to reflect the competitive binding of compounds. OMA

denotes Omaveloxolone.

2. The labeling in the Figures is not consistent.

Reply: Thank you for your kind reminder. We have carefully checked all figure labeling across all figures to maintain consistent presentation.

3. Line 121 'serves' should be 'serving'

Reply: Thank you for your careful review. We have made revisions accordingly.

Reviewer #2 (Remarks to the Author)

The manuscript investigates Omaveloxolone as a non-covalent STING degrader and proposes that it acts through a "molecular crowbar" mechanism that destabilizes the STING ligand-binding domain. The authors use a screening approach followed by biochemical, biophysical, and cellular assays—including FP, thermal shift, mutagenesis, ITC, computational modeling, and proteomics—to build a mechanistic framework for how the compound promotes HUWE1-dependent ubiquitination and degradation of STING. The work extends into in vivo models, showing effects in both TREX1–/– mice and naturally aged animals, and demonstrates degradation of both wild-type and gain-of-function STING variants by a drug that is already clinically approved and capable of brain penetration. Overall, the manuscript is of high quality, with strong data and a compelling story. The mechanistic claims are mostly well supported, and the therapeutic argument is well situated within current interest in STING biology and inflammation. With that said, there are areas where additional nuance and caution would improve both the scientific rigor and the credibility of the central claims.

Reply: We sincerely thank the reviewer's summary and positive feedback. Our detailed responses are provided below.

1. The claim that Omaveloxolone functions as a molecular crowbar is plausible, but the field's definition of "crowbar" is still in its infancy, and the authors may be overextending the strength of the inference. At present, the evidence for destabilization is indirect—protein thermal shift and MD simulations—and while these are certainly suggestive, they do not unequivocally demonstrate the

specific conformational mechanism proposed. A more measured tone, or ideally a complementary experimental assay (HDX-MS, limited proteolysis, backbone protection analyses), would go a long way toward solidifying the mechanistic argument.

Reply: We appreciate the reviewer's recognition of our proposed mechanism that Omaveloxolone acts as a molecular crowbar. As the reviewer pointed out, the definition of "molecular crowbar" is still in the early stage of development in this research field. Following the reviewer's suggestion, we have revised the manuscript with a more measured tone. We now describe Omaveloxolone as exhibiting "molecular crowbar-like behavior" or being "consistent with a molecular crowbar mechanism".

In addition, we also performed hydrogen-deuterium exchange mass spectrometry (HDX-MS) analysis as recommended by the reviewer. The HDX-MS results revealed region-specific increases in deuterium uptake within the STING LBD, mainly covering peptides spanning residues 154–162, 176–181, 213–221, and 324–337. These segments are located within α-helical regions of the LBD, indicating reduced backbone protection and increased local solvent accessibility, flexibility, and conformational dynamics upon Omaveloxolone binding (Fig. R9). These findings demonstrate that Omaveloxolone induces local conformational loosening and structural destabilization within specific regions of the STING LBD, which is highly consistent with our PTS results and MD simulation-predicted conformational changes (Fig. 4b–g). This provides further experimental evidence supporting the molecular crowbar-like mechanism of Omaveloxolone and refines our understanding of Omaveloxolone's mechanism of action. We have incorporated the data into the revised manuscript.

Once again, we sincerely thank the reviewer for the valuable comment and suggestion, which have been essential for strengthening our mechanistic evidence chain and improving the scientific rigor of our manuscript.

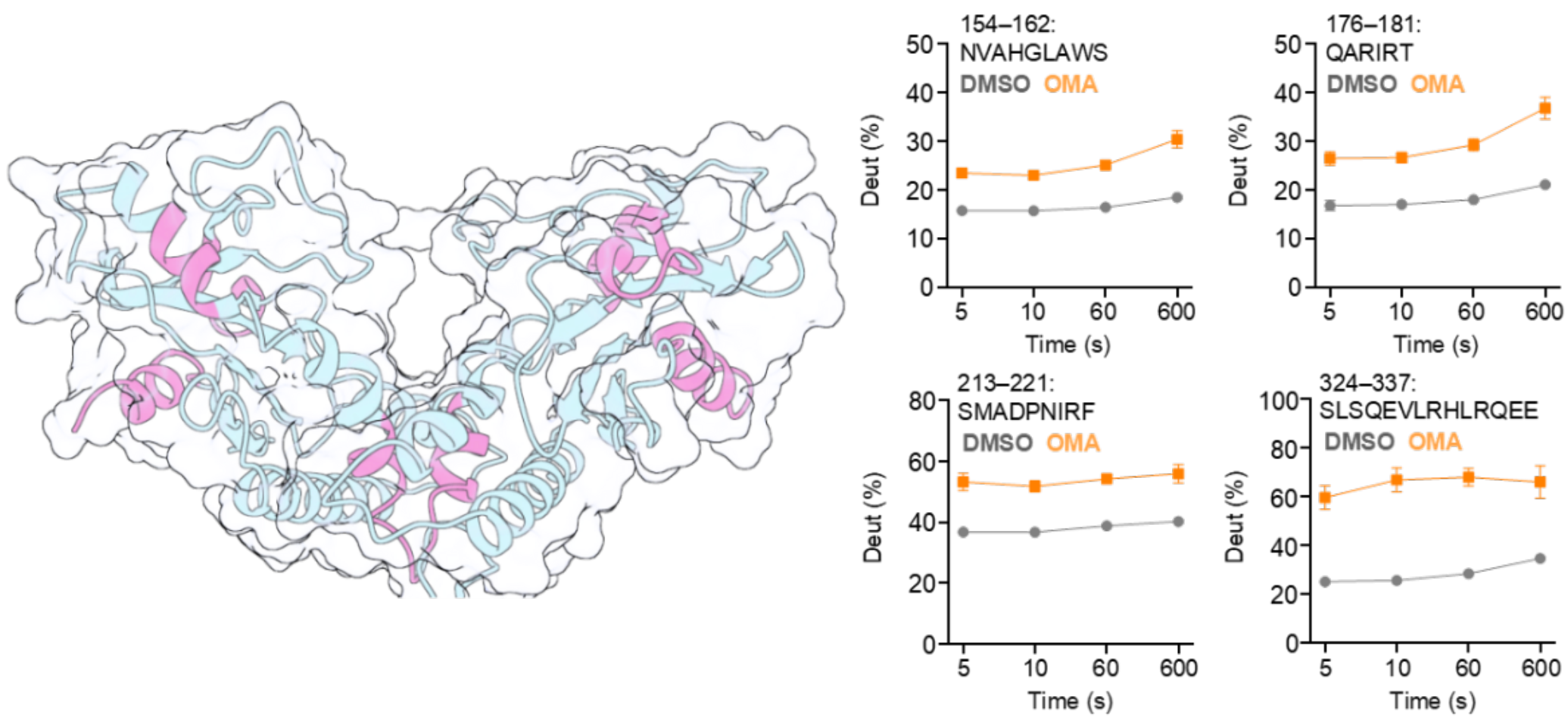

Fig. R9. Structural mapping of STING LBD regions showing increased deuterium uptake upon Omaveloxolone binding revealed by HDX-MS.

Structural mapping (left) and deuterium uptake plots (right) showing peptide regions in the STING LBD dimer with increased deuterium uptake upon Omaveloxolone (OMA) binding. The regions with increased deuterium uptake are highlighted in magenta on the STING LBD dimer structure, while the remaining structure is shown in light cyan. The HDX-MS data are presented as mean ± SD (n = 4 independent biological replicates).

2. The discrepancy between the MD simulations (one molecule bound) and ITC (two binding events) is brushed aside a bit too quickly. Readers will immediately notice this inconsistency, and at minimum a thoughtful explanation or hypothesis is needed. Even if the authors cannot resolve it experimentally, acknowledging the tension would show intellectual honesty.

Reply: Thank you for your insightful comment. In our ITC assay, STING protein was loaded in the titration syringe and Omaveloxolone in the sample cell, with STING protein sequentially titrated into Omaveloxolone solution. Under this setup, the fitted N value represents the binding stoichiometry of the titrant (STING protein) to the analyte (Omaveloxolone) in the sample cell (i.e., STING:Omaveloxolone). The N value of approximately 2 measured in this experiment indicates that two STING monomers bind to one Omaveloxolone molecule, which translates biologically to one STING dimer binding one molecule of OMA. This binding mode is consistent with our MD simulation results.

The stoichiometry we observed (one STING dimer binding one ligand) is consistent with previously reported ITC findings[1-2]. Co-crystal structures of STING in complex with the canonical ligand c-di-GMP have clearly confirmed that one molecule of c-di-GMP binds to the ligand binding pocket

of STING dimer[1-3]. In the work from Yin *et al.*, the reverse titration scheme was adopted (c-di-GMP titrated into STING solution), yielding an N value of approximately 0.5, which equivalently corresponds to one c-di-GMP bound per two STING monomers (i.e., one STING dimer)[1]. The reciprocal numerical relationship between these two N values (2 vs 0.5) originates essentially from reversed titration orientations yet describes identical intrinsic binding stoichiometry. Moreover, the STING protein construct utilized in our study is identical to those employed by Yin *et al*. Collectively, these results confirm that one STING dimer binds one molecule of Omaveloxolone, consistent with our MD simulation results.

References:

[1] Yin Q, Tian Y, Kabaleeswaran V, Jiang X, Tu D, Eck MJ, Chen ZJ, Wu H. Cyclic di-GMP sensing via the innate immune signaling protein STING. Mol Cell. 2012, 46(6):735-45.

[2] Shu C, Yi G, Watts T, Kao CC, Li P. Structure of STING bound to cyclic di-GMP reveals the mechanism of cyclic dinucleotide recognition by the immune system. Nat Struct Mol Biol. 2012, 19(7):722-4.

[3] Ouyang S, Song X, Wang Y, Ru H, Shaw N, Jiang Y, Niu F, Zhu Y, Qiu W, Parvatiyar K, Li Y, Zhang R, Cheng G, Liu ZJ. Structural analysis of the STING adaptor protein reveals a hydrophobic dimer interface and mode of cyclic di-GMP binding. Immunity. 2012, 36(6):1073-86.

3. The identification of HUWE1 as the responsible E3 ligase is convincing, but the mechanistic model (that HUWE1 engages STING specifically because the crowbar effect exposes hydrophobic regions) remains speculative. Recruitment could just as well be downstream of early ubiquitination events or altered trafficking. The authors are close to a strong mechanistic claim here, and a few additional controls—particularly proteasome blockade followed by interaction assays—would help distinguish cause from consequence.

Reply: We thank the reviewer for recognizing that our identification of HUWE1 as the responsible E3 ligase is convincing. The reviewer raises the possibility that HUWE1 recruitment to STING could be downstream of early ubiquitination events. We agree that this is a plausible alternative, as HUWE1 contains canonical ubiquitin-binding domains, including UBA, UIM, and UBM, which are capable of recognizing pre-formed ubiquitin chains generated by other E3 ligases[1-2]. Thus, it remains possible that Omaveloxolone binding to STING induces early ubiquitination events, which

in turn recruit HUWE1.

Following the reviewer's suggestion, we performed additional control experiments using proteasome inhibition to test this possibility. Cells were pretreated with PR171 to block STING degradation, followed by Omaveloxolone treatment, and the HUWE1-STING interaction was assessed via Co-IP. If HUWE1 recruitment relied on preceding ubiquitination, accumulated ubiquitin chains upon PR171 treatment would be expected to enhance their interaction. However, as shown in Fig. R10, PR171 treatment did not increase the HUWE1-STING interaction compared to control conditions. This result argues against the possibility that HUWE1 recruitment to STING is simply downstream of early ubiquitination events.

In addition, the recruitment of HUWE1 to STING is not downstream of altered STING trafficking. In the NanoBiT experiment shown in Fig. 5g of our manuscript, the STING LBD truncation we employed lacks the transmembrane (TM) domain—a domain essential for STING's ER anchoring and subsequent ER-to-Golgi trafficking[3]. Consequently, the STING LBD exists as a soluble cytosolic protein and is incapable of subcellular vesicular trafficking. The robust interaction observed between HUWE1 and STING LBD in this membrane-independent, cytosolic context therefore argues against the possibility that STING trafficking is a prerequisite for HUWE1 recruitment.

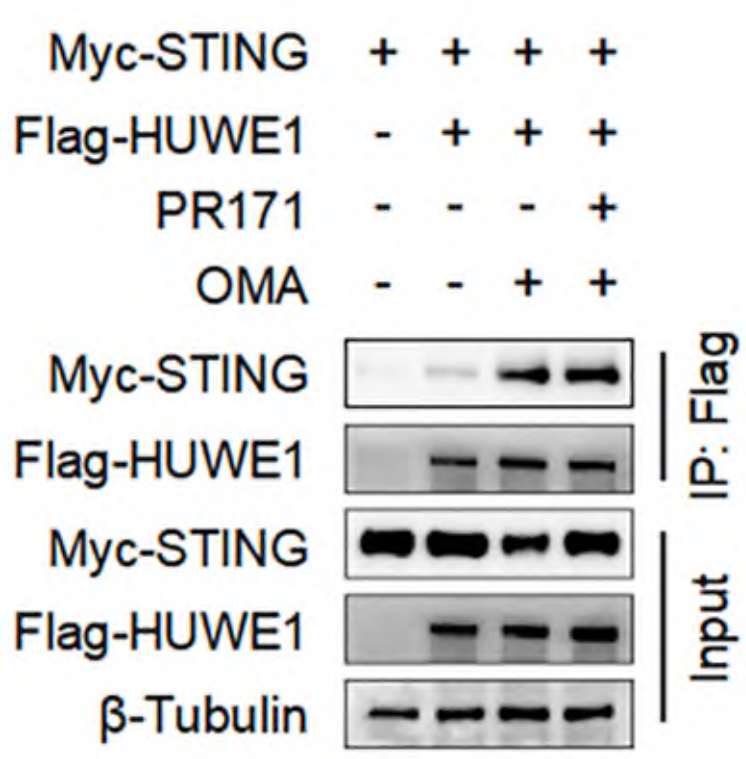

Fig. R10. Co-IP assay detecting the interaction between STING and HUWE1. HEK293T cells were transfected with Myc-STING and Flag-HUWE1 plasmids for 24 h. After pretreatment with PR171 (2 μM) for 1 h, cells were treated with OMA (350 nM) for 12 h. OMA denotes Omaveloxolone.

References:

[1] Hunkeler M, Jin CY, Ma MW, Monda JK, Overwijn D, Bennett EJ, Fischer ES. Solenoid

architecture of HUWE1 contributes to ligase activity and substrate recognition. Mol Cell. 2021, 81(17):3468-3480.e7.
[2] Buchberger A. From UBA to UBX: new words in the ubiquitin vocabulary. Trends Cell Biol. 2002, 12(5):216-21.
[3] Hopfner KP, Hornung V. Molecular mechanisms and cellular functions of cGAS-STING signalling. Nat Rev Mol Cell Biol. 2020, 21(9):501-521.

4. The proteomic data revealing downregulation of other proteins is addressed somewhat superficially. Several of these proteins have meaningful roles in immune regulation or nucleic acid metabolism, and dismissing them as irrelevant is too quick. It is admittedly difficult to know what is direct and what is downstream of STING suppression, but the manuscript would benefit from a simple experiment in STING-KO cells to determine whether these effects are STING-dependent.

Reply: We thank the reviewer for this constructive suggestion. To clarify whether Omaveloxolone-mediated downregulation of the proteomically identified proteins is STING-dependent, we performed additional proteomic analysis on STING-KO-THP-1 cells. As shown in Fig. R5, Omaveloxolone markedly downregulated a panel of proteins with established functions in immune regulation and nucleic acid metabolism in WT THP-1 cells, including, TDO2[1], USP3[2], ARMC6[3], IL1B[4], SETX[5], and MAFB[6-7]. In STING-KO THP-1 cells, the Omaveloxolone-mediated downregulation of ARMC6, SETX and USP3 was still significant, indicating that the modulation of these proteins occurs largely through STING-independent mechanisms. In contrast, the downregulation of MAFB, TDO2 and IL1B was abolished upon STING deletion, demonstrating that the suppression of these three proteins is STING-dependent. Notably, IL1B serves as a canonical pro-inflammatory downstream effector of NF-κB signaling[8], while TDO2 functions as a key immunomodulatory enzyme induced by inflammatory stress[9]. Importantly, pro-inflammatory cytokines including IL1B can induce TDO2 expression[10], and TDO2-derived metabolites can in turn modulate inflammatory signaling[11]. The coordinated STING-dependent downregulation of these two factors may collectively contribute to the protective effects of Omaveloxolone against aging-related inflammation.

We have incorporated these findings and discussions into the revised manuscript.

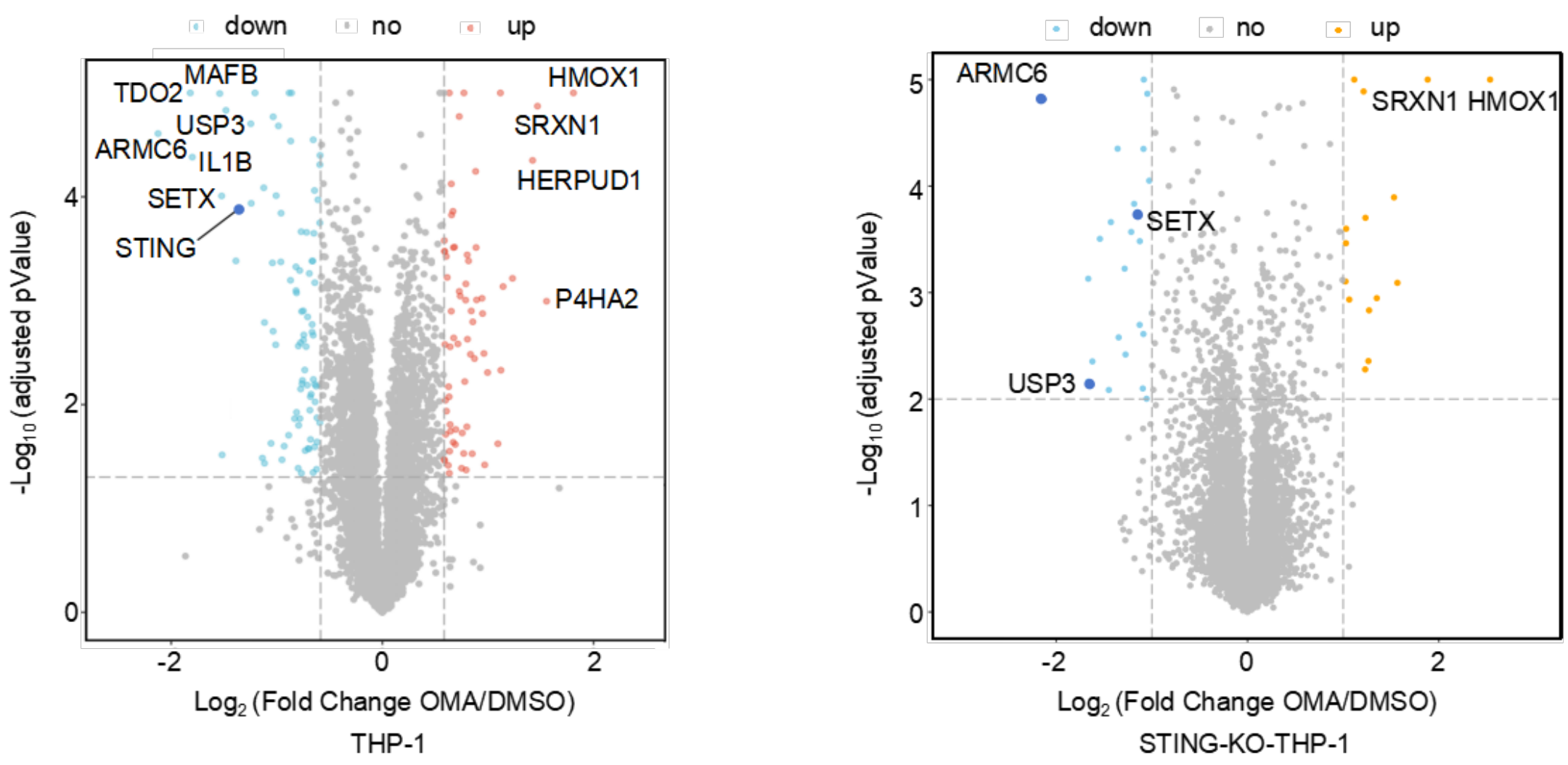

Fig. R5. Proteomic analysis of THP-1 and STING-KO-THP-1 cells treated with 500 nM Omaveloxolone (OMA) for 12 h. The volcano plot displays differentially expressed proteins compared to the DMSO control.

References:

[1] Platten M, Nollen EAA, Röhrig UF, Fallarino F, Opitz CA. Tryptophan metabolism as a common therapeutic target in cancer, neurodegeneration and beyond. Nat Rev Drug Discov. 2019, 18(5):379-401.

[2] Li S, Xiong S, Li Z, Yang L, Yang H, Xiong J, Pan W, Guo J, Xu S, Fu B. USP3 promotes DNA damage response and chemotherapy resistance through stabilizing and deubiquitinating SMARCA5 in prostate cancer. Cell Death Dis. 2024, 15(11):790.

[3] Adámik M, Soldánová Z, Drotárová M, Brečková K, Petr M, Helma R, Jenner LP, Vorlíčková M, Sýkorová E, Brázdová M. Human ARMC6 binds in vitro to both cancer genes and telomeric RNA, favoring G-quadruplex structure recognition. Biochim Biophys Acta Gene Regul Mech. 2024, 1867(3):195050.

[4] Bleve A, Motta F, Durante B, Pandolfo C, Selmi C, Sica A. Immunosenescence, Inflammaging, and Frailty: Role of Myeloid Cells in Age-Related Diseases. Clin Rev Allergy Immunol. 2023, 64(2):123-144.

[5] Bennett CL, Dastidar SG, Ling SC, Malik B, Ashe T, Wadhwa M, Miller DB, Lee C, Mitchell MB, van Es MA, Grunseich C, Chen Y, Sopher BL, Greensmith L, Cleveland DW, La Spada AR. Senataxin mutations elicit motor neuron degeneration phenotypes and yield TDP-43 mislocalization in ALS4 mice and human patients. Acta Neuropathol. 2018, 136(3):425-443.

[6] Vanneste D, Bai Q, Hasan S, Peng W, Pirottin D, Schyns J, Maréchal P, Ruscitti C, Meunier M, Liu Z, Legrand C, Fievez L, Ginhoux F, Radermecker C, Bureau F, Marichal T. MafB-restricted local monocyte proliferation precedes lung interstitial macrophage differentiation. Nat Immunol. 2023, 24(5):827-840.

[7] Ventura AB, Loconte T, Negri A, Viggiano L, Fiermonte G, Ciavarella S, Guarini A, Volpe G. MAFB: a key regulator of myeloid commitment involved in hematological diseases. Cell Death Discov. 2025, 11(1):276.
[8] Bleve A, Motta F, Durante B, Pandolfo C, Selmi C, Sica A. Immunosenescence, Inflammaging, and Frailty: Role of Myeloid Cells in Age-Related Diseases. Clin Rev Allergy Immunol. 2023, 64(2):123-144.
[9] Platten M, Nollen EAA, Röhrig UF, Fallarino F, Opitz CA. Tryptophan metabolism as a common therapeutic target in cancer, neurodegeneration and beyond. Nat Rev Drug Discov. 2019, 18(5):379-401.
[10] Rong G, Zhang T, Xu Y, Zhang Z, Gui B, Hu K, Zhang J, Tang Z, Shen C. High levels of TDO2 in relation to pro-inflammatory cytokines in synovium and synovial fluid of patients with osteoarthritis. BMC Musculoskelet Disord. 2022, 23(1):604.
[11] Wawrzak-Pienkowska K, Golonko A, Ignatiuk D, Swidnicka-Siergiejko A, Pienkowski T, Kurek K. Kynurenine pathway impact on immune evasion and inflammation in acute myeloid leukemia. Life Sci. 2026, 400:124474.

5. The in vivo section is strong in terms of efficacy but limited in terms of safety or tolerability. Since Omaveloxolone is used chronically in these mice, even limited indicators—body weight trajectories, gross pathology, or clinical chemistry—would strengthen the translational argument. Without such data, the reader is left to assume tolerability rather than being shown it.

Reply: We thank the reviewer for raising this important point regarding safety and tolerability, which is essential to support the translational implication of our study.

We have monitored body weight changes of aged mice throughout the chronic treatment course. No significant differences in body weight trajectories were observed between the Omaveloxolone-treated and vehicle control groups, indicating favorable tolerability at the therapeutic dose based on this limited readout (Fig. R11).

Beyond our own body weight results, robust clinical and preclinical reports further substantiate Omaveloxolone's favorable safety profile. Omaveloxolone is an FDA-approved therapeutic agent whose long-term safety and tolerability have been rigorously verified in clinical trials[1-2]. Consistently, multiple independent chronic-dosing preclinical investigations across distinct mouse disease models confirm that prolonged Omaveloxolone treatment (ranging from 4 to 9 weeks) does not trigger overt systemic toxic effects or body weight loss at effective therapeutic dosages[3-6]. Collectively, existing clinical and multi-model preclinical evidence supports the chronic tolerability

of Omaveloxolone, reinforcing the translational potential of our pharmacological observations. We also acknowledge that our own study did not include a full clinical chemistry panel, which represents a limitation of the current tolerability assessment.

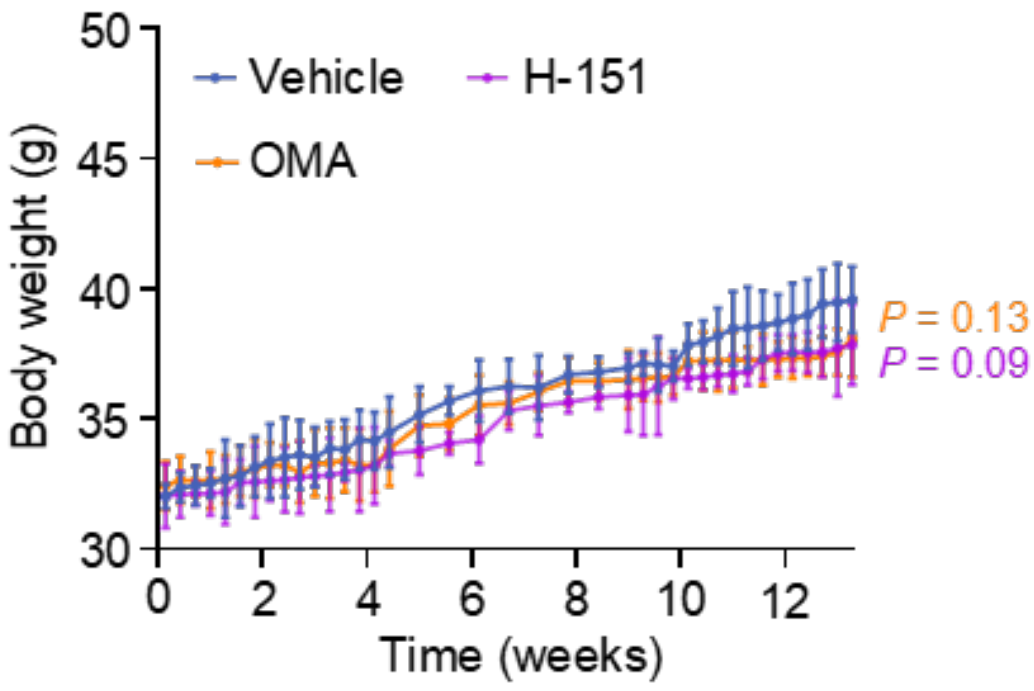

Fig. R11. Body weight trajectories during chronic OMA treatment in aged mice. 22-month-old wild-type C57BL/6 mice were injected with OMA (i.p. 10 mg kg^{-1}) once every two days or H-151 (i.p. 10 mg kg^{-1}) daily for one month. The treatment regimen consisted of one month of dosing, followed by a one-month treatment-free interval, and then a second month of the same dosing schedule. Body weights were recorded every two days during treatment periods and every four days during the interval (n = 5 mice/group, mean ± SD). OMA denotes Omaveloxolone. P values were calculated by two-tailed unpaired t-test.

References:

[1] Lynch DR, Chin MP, Delatycki MB, Subramony SH, Corti M, Hoyle JC, Boesch S, Nachbauer W, Mariotti C, Mathews KD, Giunti P, Wilmot G, Zesiewicz T, Perlman S, Goldsberry A, O'Grady M, Meyer CJ. Safety and Efficacy of Omaveloxolone in Friedreich Ataxia (MOXIe Study). Ann Neurol. 2021, 89(2):212-225.

[2] Gunther K, Profeta V, Keita M, Park C, Wells M, Sharma S, Schadt K, Lynch DR. Safety Monitoring of Omaveloxolone in Friedreich Ataxia: Results from One Year of Clinical Treatment. Neurol Ther. 2025, 14(3):1105-1114.

[3] Salinas L, Figueroa F, Montgomery CB, Thai PN, Chiamvimonvat N, Cortopassi G, Dedkova EN. Omaveloxolone, But Not Dimethyl Fumarate, Improves Cardiac Function in Friedreich's Ataxia Mice With Severe Cardiomyopathy. J Am Heart Assoc. 2025, 14(12):e038505.

[4] Wang X, Liu T, Fei Y, Zhang S, Yang Y, Chen Z, Zhu R, Deng S, Zhang T, Wu D, Xu Y. RTA-408 overcomes cisplatin-resistant lung cancer by inhibiting WWP1-mediated NCOA4 ubiquitination to induce ferritinophagy and ferroptosis. Free Radic Biol Med. 2025, 238:595-610.

[5] Lin X, Wang H, Zou L, Yang B, Chen W, Rong X, Zhang X, He L, Li X, Peng Y. The NRF2 activator RTA-408 ameliorates chronic alcohol exposure-induced cognitive impairment and NLRP3 inflammasome activation by modulating impaired mitophagy initiation. Free Radic Biol Med. 2024,

220:15-27.
[6] Yang B, Pan J, Zhang XN, Wang H, He L, Rong X, Li X, Peng Y. NRF2 activation suppresses motor neuron ferroptosis induced by the SOD1G93A mutation and exerts neuroprotection in amyotrophic lateral sclerosis. Neurobiol Dis. 2023, 184:106210.

6. Because Omaveloxolone is a known NRF2 activator, the anti-inflammatory effects observed in aged animals likely represent a mixture of STING suppression and NRF2-driven cytoprotective gene expression. The manuscript acknowledges the NRF2 connection only briefly, but reviewers will certainly raise it. Addressing this more directly—either by adding data or by discussing limitations—will significantly improve the manuscript's scientific balance.

Reply: We appreciate the reviewer's valuable and constructive comment pointing out the insufficient discussion of the NRF2 pathway in our original manuscript. We agree with the reviewer that the anti-inflammatory effects observed in aged animals likely represent a combined outcome of Omaveloxolone-mediated STING suppression and NRF2-driven cytoprotective gene expression. As suggested by the reviewer, comprehensive elaboration on the functional contribution of the NRF2 pathway to Omaveloxolone-mediated anti-inflammatory benefits in aged animals, together with explicit discussion of limitations, substantially improves the scientific rigor and balanced interpretation of our findings.

Our *in vivo* data demonstrate that Omaveloxolone facilitates STING protein degradation in tissues from aged mice and effectively represses inflammatory signaling mediated by the STING pathway (Fig. 7i). Meanwhile, as a well-established NRF2 activator, Omaveloxolone also upregulates a panel of antioxidant and detoxification enzymes including NQO1, HO-1, and GCLC to alleviate cellular oxidative stress and further exerts anti-inflammatory effects[1-2].

A core pathological feature of the aging organism is the concurrent occurrence of chronic low-grade inflammation (inflammaging) and impaired antioxidant capacity[3-4], two pathophysiological phenotypes predominantly governed by the STING and NRF2 signaling axes, respectively[5-9]. During aging, progressive decline in NRF2 transcriptional activity compromises endogenous antioxidant defense; concurrently, the STING pathway becomes aberrantly and persistently activated due to the accumulation of cytosolic DNA caused by multiple factors. Omaveloxolone has the potential to simultaneously rectify these two aging-associated pathway perturbations: on one

hand, it interrupts pro-inflammatory signaling via direct STING degradation; on the other hand, it reinforces cellular antioxidant defense through NRF2 activation. Based on this mechanistic rationale, the overall anti-inflammatory phenotype observed in aged mice is plausibly attributable to synergistic actions of the two pathways. However, our current experimental dataset cannot quantitatively dissect the individual contribution of each mechanism.

The inability to separate the respective contributions of STING degradation and NRF2 activation toward Omaveloxolone's anti-inflammatory efficacy in aged animals constitutes a limitation of this work. Following the reviewer's suggestion, we have provided a more comprehensive discussion and clearly outlined this limitation within the revised Discussion section.

References:

[1] Salinas L, Figueroa F, Montgomery CB, Thai PN, Chiamvimonvat N, Cortopassi G, Dedkova EN. Omaveloxolone, But Not Dimethyl Fumarate, Improves Cardiac Function in Friedreich's Ataxia Mice With Severe Cardiomyopathy. J Am Heart Assoc. 2025, 14(12):e038505.

[2] Reisman SA, Gahir SS, Lee CI, Proksch JW, Sakamoto M, Ward KW. Pharmacokinetics and pharmacodynamics of the novel Nrf2 activator omaveloxolone in primates. Drug Des Devel Ther. 2019, 13:1259-1270.

[3] López-Otín C, Blasco MA, Partridge L, Serrano M, Kroemer G. Hallmarks of aging: An expanding universe. Cell. 2023, 186(2):243-278.

[4] Fang S, Jiang M, Jiao J, Zhao H, Liu D, Gao D, Wang T, Yang Z, Yuan H. Unraveling the ROS-Inflammation-Immune Balance: A New Perspective on Aging and Disease. Aging Dis. 2025, 17(1):305-325.

[5] Gulen MF, Samson N, Keller A, Schwabenland M, Liu C, Glück S, Thacker VV, Favre L, Mangeat B, Kroese LJ, Krimpenfort P, Prinz M, Ablasser A. cGAS-STING drives ageing-related inflammation and neurodegeneration. Nature. 2023, 620(7973):374-380.

[6] Shang Z, Qin D, Liu X, Li H, Liu C, Zhang R, Sun T, Pan Z, Feng W, You X. The cGAS-STING pathway in age-related ocular diseases: Mechanisms and therapeutic opportunities. Cell Signal. 2025, 135:112069.

[7] Suh JH, Shenvi SV, Dixon BM, Liu H, Jaiswal AK, Liu RM, Hagen TM. Decline in transcriptional activity of Nrf2 causes age-related loss of glutathione synthesis, which is reversible with lipoic acid. Proc Natl Acad Sci U S A. 2004, 101(10):3381-6.

[8] Safdar A, deBeer J, Tarnopolsky MA. Dysfunctional Nrf2-Keap1 redox signaling in skeletal muscle of the sedentary old. Free Radic Biol Med. 2010, 49(10):1487-93.

[9] Zhang H, Davies KJA, Forman HJ. Oxidative stress response and Nrf2 signaling in aging. Free

Radic Biol Med. 2015, 88(Pt B):314-336.

Reviewer #3 (Remarks to the Author):

The manuscript titled "Omaveloxolone functions as a molecular crowbar degrader of STING to alleviate aging-related inflammation" presents an intriguing and compelling study. The authors propose an innovative mechanism for Omaveloxolone, identifying it as a "molecular crowbar" that directly destabilizes and degrades STING. Overall, the experimental design is rigorous, and the data appear robust and well-controlled. The authors successfully demonstrate the therapeutic potential of this mechanism in the context of aging-related inflammation.

However, despite the methodological rigor, further clarification is required regarding the structural basis of the drug-target interaction and respective contributions of its dual mechanisms.

Reply: We sincerely appreciate the reviewer’s summary and positive feedback. Our detailed responses are provided below.

Major Comments:

1.Despite extensive MD analyses, empirical structural evidence is necessary to fully elucidate the Omaveloxolone-STING interaction. To validate the proposed "molecular crowbar" mechanism, the authors should provide a crystal structure or Cryo-EM analysis of the complex.

Reply: We sincerely thank the reviewer for this important suggestion. We fully agree that a high-resolution structure of the Omaveloxolone-STING complex would provide more direct structural evidence for defining its binding mode and conformational mechanism.

Following the reviewer’s recommendation, we performed extensive co-crystallization screenings under various conditions, including different buffer systems and temperatures. We also tested multiple protein batches to rule out batch-specific issues. Despite these extensive efforts, we regret that we were unable to obtain diffraction-quality co-crystals for structural determination.

We speculate that this difficulty may be related to the conformational destabilization induced by Omaveloxolone binding. Our PTS assays showed that recombinant wild type STING protein has relatively low intrinsic thermal stability, with a T_m of approximately 38°C. Upon Omaveloxolone

binding, the T_m further decreased to approximately 34°C (Fig. 4b). These results suggest that Omaveloxolone binding might increase the conformational flexibility and heterogeneity of the STING protein, which could disfavor the formation of a well-ordered crystal lattice for the Omaveloxolone-STING complex. We speculate that this may be one important factor contributing to the difficulty in obtaining high-quality co-crystals of the complex.

We fully recognize the reviewer's concern regarding high-resolution structural evidence. Accordingly, we have tempered our mechanistic claims, revising the more definitive "molecular crowbar" mechanism description to the more cautious phrasing "exhibiting molecular crowbar-like behavior" or "being consistent with a molecular crowbar-like mechanism". We have also added a statement in the Discussion acknowledging the current lack of a high-resolution Omaveloxolone-STING complex structure as a limitation of this study, and that future structural biology efforts will be required to further define the precise binding mode.

Although we were unable to obtain the complex structure at this stage, we consider that the current combination of multiple independent lines of evidence—including ITC, key residue mutations, PTS assays, SAR of analogs, and microsecond-scale MD simulations—provides a coherent mechanistic framework supporting Omaveloxolone-induced conformational perturbation of STING and its subsequent degradation.

We again thank the reviewer for this constructive suggestion.

2. While the authors demonstrated that STING degradation occurs independently of NRF2, the study does not clearly differentiate the therapeutic contribution of Omaveloxolone's NRF2 activation from its STING-degrading activity in the disease model. The authors should either dissect the relative impact of these two mechanisms on the observed phenotype or explicitly characterize Omaveloxolone as a dual-function modulator that targets both pathways synergistically.

Reply: We greatly appreciate the reviewer for this insightful and constructive comment pointing out the insufficient discussion of the NRF2 pathway in our original manuscript. We agree that thorough discussion dissecting the individual therapeutic contributions of STING suppression and NRF2 activation within our disease model would substantially improve the scientific rigor of the manuscript.

In terms of functional linkage, Omaveloxolone drives STING degradation and NRF2 activation through independent molecular pathways, with no upstream/downstream or causal relationship

between the two events. On one hand, Omaveloxolone-mediated STING degradation proceeds at the proteasomal level without altering STING mRNA expression, and this degradation process is NRF2-independent (Fig. 2e). On the other hand, Omaveloxolone activates NRF2 through covalent modification of KEAP1, a well-established mechanism validated in the field. In addition, proteomic profiling in STING-KO-THP-1 cells reveals that Omaveloxolone still robustly upregulates the canonical NRF2 downstream targets including HMOX1 and SRXN1 to levels comparable to those in WT cells (Fig. R5). These findings collectively rule out the possibility that one pharmacological outcome arises as a secondary byproduct of the other and establish Omaveloxolone as a dual-function modulator capable of independently regulating the two signaling axes simultaneously.

Further, this dual-target property endows Omaveloxolone with profound pharmacological complementarity, precisely addressing the two core pathological drivers of aging-related inflammation. A hallmark feature of the aging organism is the coexistence of chronic low-grade inflammation (inflammaging) and declining antioxidant capacity[1-2]: the STING pathway becomes aberrantly and persistently activated due to the accumulation of cytosolic DNA and is a core driver of inflammaging[3-4], whereas NRF2 transcriptional activity progressively declines with age, directly compromising endogenous antioxidant defense[5-7], and the two processes reinforce each other to form a vicious cycle. Omaveloxolone can simultaneously rectify both aging-associated pathway dysregulations: on one hand, it directly degrades STING, blocking inflammatory signaling cascades at their source; on the other hand, it activates NRF2, enhancing cellular antioxidant defense. Such dual regulatory strategy combining anti-inflammation and antioxidant effects directly addresses two interconnected pathogenic pillars of inflammaging, enabling natural functional synergy between the two pathways.

Notably, our cellular assays have preliminarily dissected their relative functional contributions (Fig. R4). In WT cells, Omaveloxolone treatment significantly alleviated senescence-related inflammation, as evidenced by reduced the mRNA levels of senescence markers (*P16* and *P21*) and pro-inflammatory SASP factors (*IL6* and *IL1B*), as well as decreased proportion of SA-β-Gal-positive cells (Fig. R4a–d). In contrast, the anti-inflammatory efficacy of Omaveloxolone was nearly abolished in STING-KO senescent cell models (Fig. R4e–h). These results support that STING degradation constitutes the predominant route mediating Omaveloxolone's anti-inflammaging activity *in vitro*. Nevertheless, we acknowledge that the *in vivo* microenvironment of naturally aged mice is far more sophisticated than simplified cellular systems, wherein STING-

driven hyperinflammation and NRF2-mediated impaired antioxidant capacity coexist and intertwine over the long term. Our current *in vivo* data cannot quantitatively dissect the individual contribution of each mechanism. Therefore, we cannot completely rule out the possibility that NRF2 activation may partially contribute to the *in vivo* protective effects, and we recognize this as a limitation of the present study regarding the *in vivo* pharmacological mechanism

In accordance with the reviewer's suggestions, we have made comprehensive additions to the Discussion section of the revised manuscript: we now explicitly characterize Omaveloxolone as a dual-function modulator that independently targets both STING and NRF2, elaborate on the mechanistic independence and functional synergy of the two pathways, and objectively state the limitations of the current study. These revisions have substantially improved the scientific balance and rigor of the manuscript. Once again, we sincerely thank the reviewer for the expert guidance.

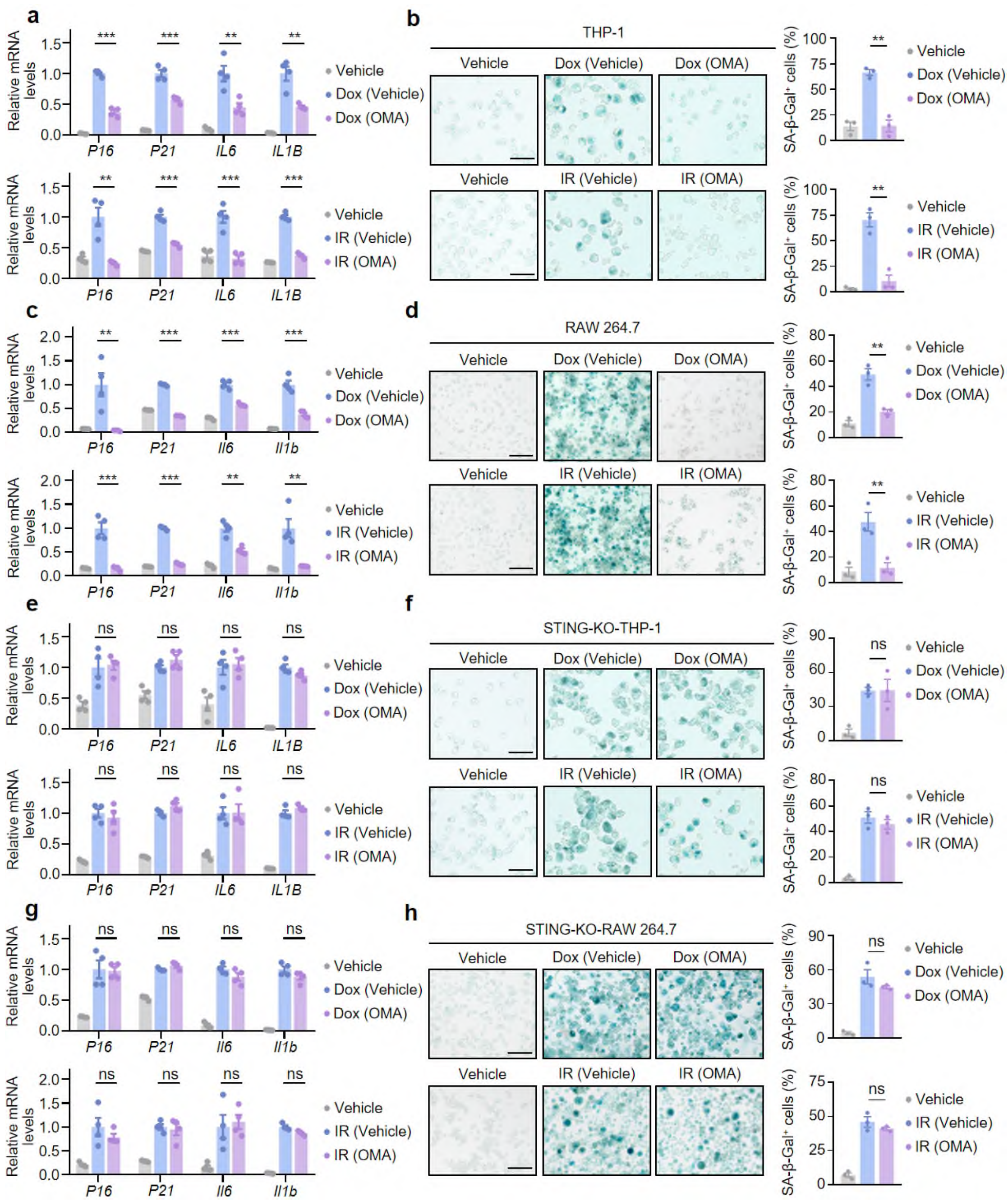

Fig. R4. Omaveloxolone alleviates cellular senescence and senescence-related inflammation in a STING-dependent manner. a,b, THP-1 cells were treated with 200 nM Dox for 24 h followed by 4 days of culture, or irradiated with 7.5 Gy X-ray and cultured for 7-9 days, to induce cellular senescence. Cells were then treated with 500 nM OMA for 24 h. The mRNA levels of senescence markers (*P16*, *P21*) and pro-inflammatory SASP factors (*IL6*, *IL1B*) were measured by RT-qPCR (n = 4, two technical replicates from two independent experiments, mean ± SEM) (**a**). Cellular senescence was assessed by SA-β-Gal staining; representative images and quantifications are shown (scale bar, 50 μm; n = 3 independent biological replicates, mean ± SEM) (**b**). **c,d,** RAW 264.7 cells were treated with 200 nM Dox for 24 h followed by 2 days of culture, or irradiated with 6 Gy X-ray and cultured for 4-6 days, to induce cellular senescence. Cells were then treated with 1 μM OMA

for 24 h. The mRNA levels of *P16*, *P21*, *Il6*, and *Il1b* were measured by RT-qPCR (*n* = 4, two technical replicates from two independent experiments, mean ± SEM) (**c**). Representative SA-β-Gal staining images and quantifications are presented (scale bar, 100 μm; *n* = 3 independent biological replicates, mean ± SEM) (**d**). **e,f,** STING-KO-THP-1 cells were subjected to senescence induction as in **a,b**, followed by 500 nM OMA treatment for 24 h. The mRNA levels of *P16*, *P21*, *IL6*, and *IL1B* were measured by RT-qPCR (*n* = 4, two technical replicates from two independent experiments, mean ± SEM) (**e**). Representative SA-β-Gal staining images and quantifications are presented (scale bar, 50 μm; *n* = 3 independent biological replicates, mean ± SEM) (**f**). **g,h,** STING-KO-RAW 264.7 cells were subjected to senescence induction as in **c,d**, followed by 1 μM OMA treatment for 24 h. The mRNA levels of *P16*, *P21*, *Il6*, and *Il1b* were measured by RT-qPCR (*n* = 4, two technical replicates from two independent experiments, mean ± SEM) (**g**). Representative SA-β-Gal staining images and quantifications are presented (scale bar, 100 μm; *n* = 3 independent biological replicates, mean ± SEM) (**h**). OMA denotes Omaveloxolone. ns, not significant; $P > 0.05$; $^{**}P < 0.01$; $^{***}P < 0.001$ by two-tailed unpaired *t*-test.

References:

[1] López-Otín C, Blasco MA, Partridge L, Serrano M, Kroemer G. Hallmarks of aging: An expanding universe. Cell. 2023, 186(2):243-278.

[2] Fang S, Jiang M, Jiao J, Zhao H, Liu D, Gao D, Wang T, Yang Z, Yuan H. Unraveling the ROS-Inflammation-Immune Balance: A New Perspective on Aging and Disease. Aging Dis. 2025, 17(1):305-325.

[3] Gulen MF, Samson N, Keller A, Schwabenland M, Liu C, Glück S, Thacker VV, Favre L, Mangeat B, Kroese LJ, Krimpenfort P, Prinz M, Ablasser A. cGAS-STING drives ageing-related inflammation and neurodegeneration. Nature. 2023, 620(7973):374-380.

[4] Shang Z, Qin D, Liu X, Li H, Liu C, Zhang R, Sun T, Pan Z, Feng W, You X. The cGAS-STING pathway in age-related ocular diseases: Mechanisms and therapeutic opportunities. Cell Signal. 2025, 135:112069.

[5] Suh JH, Shenvi SV, Dixon BM, Liu H, Jaiswal AK, Liu RM, Hagen TM. Decline in transcriptional activity of Nrf2 causes age-related loss of glutathione synthesis, which is reversible with lipoic acid. Proc Natl Acad Sci U S A. 2004, 101(10):3381-6.

[6] Safdar A, deBeer J, Tarnopolsky MA. Dysfunctional Nrf2-Keap1 redox signaling in skeletal muscle of the sedentary old. Free Radic Biol Med. 2010, 49(10):1487-93.

[7] Zhang H, Davies KJA, Forman HJ. Oxidative stress response and Nrf2 signaling in aging. Free Radic Biol Med. 2015, 88(Pt B):314-336.

Reviewer #4 (Remarks to the Author)

I co-reviewed this manuscript with one of the reviewers who provided the listed reports. This is part of the Nature Communications initiative to facilitate training in peer review and to provide appropriate recognition for Early Career Researchers who co-review manuscripts.

Reply: We sincerely thank the reviewer and the co-reviewer for their constructive comments.

Reviewer #1 (Remarks to the Author):

All concerns have been addressed; congratulations on your exciting results!

Reply: Thank you very much for your positive feedback. We appreciate your time and effort in reviewing our manuscript, which have helped us improve the clarity and rigor of our study.

Reviewer #2 (Remarks to the Author):

The reviewers have replied to my concerns in a satisfactory manner.

Reply: We are glad that our responses have satisfactorily addressed your concerns. Thank you for your constructive input throughout the review process, which has helped us improve the quality of our work.

Reviewer #3 (Remarks to the Author):

I have carefully evaluated the revised manuscript NCOMMS-25-93443A, "Omaveloxolone functions as a molecular crowbar-like degrader of STING to alleviate aging-related inflammation," along with your point-by-point response letter. I am pleased to note that you have addressed all major concerns raised in the previous round of review in a thorough and satisfactory manner.

In particular, the following additions substantially strengthen the manuscript:

1) The HDX-MS analysis provides compelling orthogonal experimental evidence for Omaveloxolone-induced conformational destabilization of the STING LBD, complementing the existing thermal shift and MD simulation data.

2) The K27 linkage-specific endogenous ubiquitination assays, combined with the ubiquitin K-only mutant experiments, convincingly demonstrate that Omaveloxolone promotes homotypic K27-linked polyubiquitination of STING in a cellular context.

3) The STING-KO proteomic profiling clarifies the STING-dependence of downstream protein changes and provides useful mechanistic context for the off-target observations.

4) The HUWE1 recruitment controls (proteasome inhibition Co-IP and STING LBD-based NanoBiT assays) effectively address the alternative model in which HUWE1 engagement could be secondary to prior ubiquitination or altered STING trafficking.

5) Your decision to adopt more measured language—describing Omaveloxolone as exhibiting "molecular crowbar-like behavior" rather than definitively classifying it as a molecular crowbar—

is scientifically responsible and appropriately reflects the current state of the field.

The mechanistic framework is now well-supported by multiple independent experimental approaches, and the therapeutic rationale in the context of aging-related inflammation is clearly and compellingly articulated.

Reply: Thank you for your positive feedback on our revised manuscript. We appreciate your thorough evaluation and constructive suggestions throughout the review process, which have helped us improve the quality of our study.

Reviewer #4 (Remarks to the Author):

I co-reviewed this manuscript with one of the reviewers who provided the listed reports. This is part of the Nature Communications initiative to facilitate training in peer review and to provide appropriate recognition for Early Career Researchers who co-review manuscripts.

Reply: Thank you for your participation in the co-review process and for your time and effort in reviewing our manuscript. We appreciate your valuable contribution to improving our work.
